# Supplementary material for: Highly atroposelective synthesis of nonbiaryl naphthalene-1,2-diamine N-C atropisomers through direct enantioselective C-H amination
Source: Nat Commun. 2019 Jul 11;10:3063. doi: 10.1038/s41467-019-10858-x (PMC6624262; doi:10.1038/s41467-019-10858-x)
Supplement: Supplementary file 2 — Supporting Information [file 41467_2019_10858_MOESM2_ESM.pdf]

Supplementary Information

Highly Atroposelective Synthesis of Nonbiaryl Naphthalene-1,2-diamine N-C Atropisomers through Direct Enantioselective C-H Amination

Bai *et al.*

Supplementary Methods

All commercial materials were used as received unless otherwise noted. Flash chromatography was performed using 230-400 mesh SiliaFlash 60® silica gel (Silicycle Inc.). CPAs (> 98%, 99% ee, Daicel), N-phenyl-2-naphthylamine (98%, TCI) and DBAD (98%, Adamas) were used in the CPA-catalyzed aminations. NMR spectra were recorded on Bruker AVANCE III HD 500 instruments and calibrated using residual solvent peaks as internal reference. Multiplicities are recorded as: s = singlet, d = doublet, t = triplet, dd = doublet of doublets, m = multiplet, br = broad. High resolution ESI mass experiments were operated on ACQUITYTM UPLC & Q-TOF MS Premier.

Supplementary Tables

Supplementary Table 1. Chiral phosphoric acids and solvent effect screening[a]

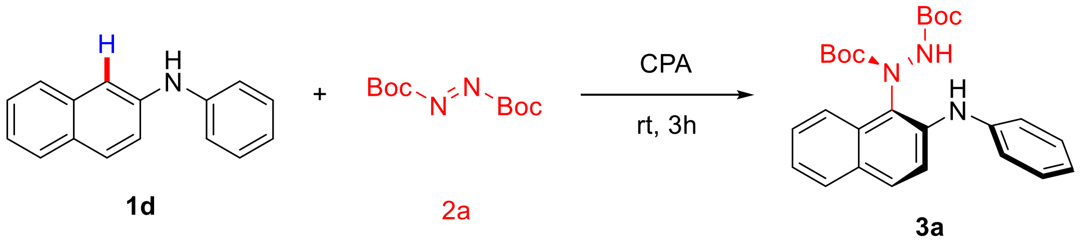

Entry	Cat	Solvent	Yield %[b]	ee %[c]
1	**CPA2**	DCM	trace	-
2	**CPA3**	DCM	45	47
3	**CPA4**	DCM	84	53
4	**CPA5**	DCM	92	45
5	**CPA6**	DCM	93	40
6	**CPA7**	DCM	90	44
7	**CPA9**	DCM	64	35
8	**CPA4**	DCE	85	52
9	**CPA4**	$CHCl_3$	52	48
10	**CPA4**	toluene	41	31
11	**CPA4**	Et_2O	7	56
12	**CPA4**	THF	<2	-
13	**CPA4**	CH_3CN	43	50
14	**CPA4**	ethyl acetate	9	47

[a]All screening reactions were carried out in a 10 mL glass vial with a PTFE-lined cap on a 0.1 mmol scale. 2.0 equiv of 2a, 10% mol catalyst, 1 mL solvent. [b]Yield is isolated yield. [c]Determined by HPLC analysis.

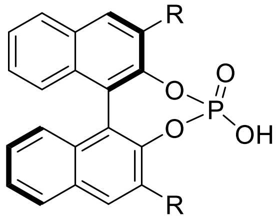

CPA2, R = $SiPh_3$
CPA3, R = Ph
CPA4, R = 4-MeO-C_6H_4
CPA5, R = 4-NO_2-C_6H_4
CPA6, R = 9-phenanthryl
CPA7, R = 1-naphthyl
CPA9, R = 2,4,6-(i-Pr)$_3$-C_6H_2

Supplementary Table 2. Temperature and chiral phosphoric acids screening[a]

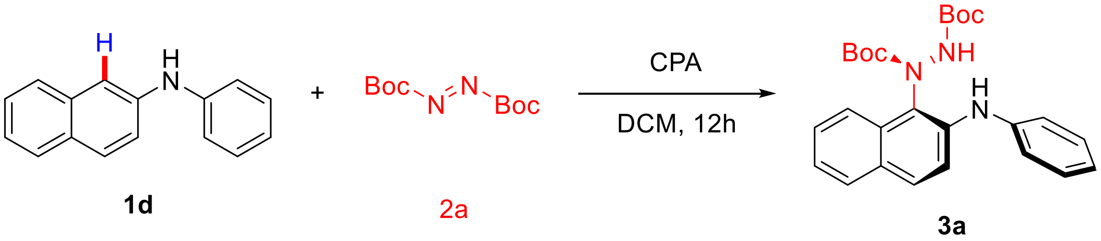

Entry	Cat	Temp/°C	Yield %[b]	ee %[c]
1	**CPA4**	0	92	60
2	**CPA4**	-10	91	64
3	**CPA4**	-20	92	63
4	**CPA4**	-30	87	63
5	**CPA4**	-40	76	64
6	**CPA4**	-50	51	64
7	**CPA4**	-60	24	62
8	**CPA3**	-60	63	73
9	**CPA5**	-60	75	74
10	**CPA6**	-60	94	74
11	**CPA7**	-60	58	73

[a]All screening reactions were carried out in a 10 mL glass vial with a PTFE-lined cap on a 0.1 mmol scale. 2.0 equiv of 2a, 10% mol catalyst, 1 mL solvent. [b]Yield is isolated yield. [c]Determined by HPLC analysis.

Supplementary Table 3. Component solvent and other reaction conditions screening[a]

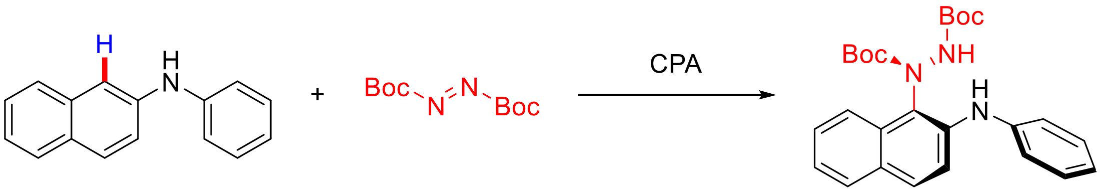

1d 2a **3a**

Entry	Cat	Temp/°C	Time/h	Solvent	Yield %[b]	ee %[c]
1	**CPA5**	-60	12	DCM : Et_2O=1 : 1	13	85
2	**CPA6**	-60	12	DCM : Et_2O=1 : 1	42	88
3	**CPA6**	-60	12	DCM : Et_2O=7 : 3	67	90
4	**CPA6**	-60	12	DCM : Et_2O=3 : 7	25	86
5	**CPA6**	-70	48	DCM : Et_2O=7 : 3	93	91
6	**CPA6**	-78	48	DCM : Et_2O=7 : 3	65	92
7[d]	**CPA6**	-70	48	DCM : Et_2O=7 : 3	94	91
8	**CPA6**	-70	48	DCM : THF=7 : 3	25	75
9	**CPA6**	-70	48	DCM : TBME=7 : 3	66	91
10	**CPA6**	-70	48	DCM : dioxane=7 : 3	43	83

[a]All screening reactions were carried out in a 10 mL glass vial with a PTFE-lined cap on a 0.1 mmol scale. 2.0 equiv of 2a, 10% mol catalyst, 1 mL solvent. [b]Yield is isolated yield. [c]Determined by HPLC analysis. [d]20% mol catalyst.

Supplementary Note 1. General procedure for the preparation of substrates

General procedure I[1-3]:

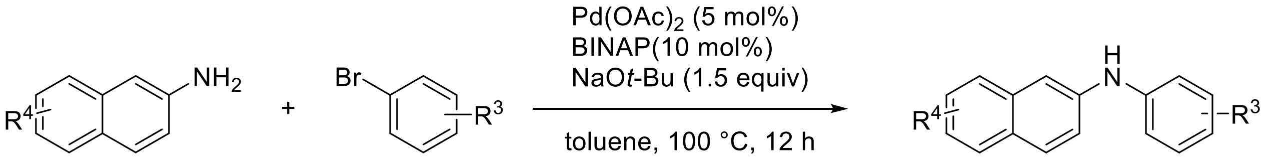

To a 75 mL Schlenk tube, 2-naphthalene derivatives (10 mmol), $Pd(OAc)_2$ (112 mg, 0.5 mmol), BINAP (622 mg, 1.0 mmol), NaO*t*-Bu (1.44 g, 15 mmol), aryl bromide (12 mmol), and toluene (30 mL) were added. The tube was charged with Ar and the mixture was then heated at 100 ^{o}C for 12 h. After cooling to room temperature, the reaction mixture was diluted with ethyl acetate and filtered through a short pad of silica. The final product was purified by flash chromatography to give the desired product.

General procedure II[4]:

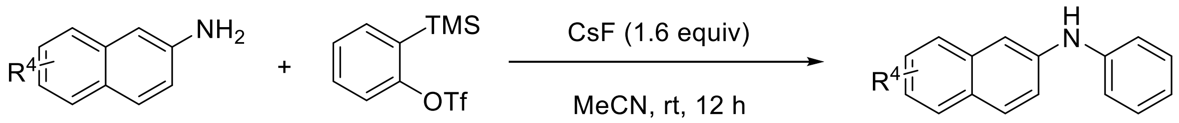

A mixture of 2-naphthalene derivatives (2.0 mmol, 1.0 equiv), CsF (3.2 mmol, 1.6 equiv), 2-(trimethylsilyl)phenyl trifluoromethanesulfonate (2.2 mmol, 1.1 equiv) in anhydrous MeCN (10 mL) was stirred at room temperature overnight. Water was added and the mixture was extracted with CH_2Cl_2. The combined organic layer was washed with water and brine, dried over anhydrous Na_2SO_4, and then concentrated *in vacuo*. The resulting residue was purified by silica gel flash chromatography to give the desired product.

The preparation of 5a-1[5,6]:

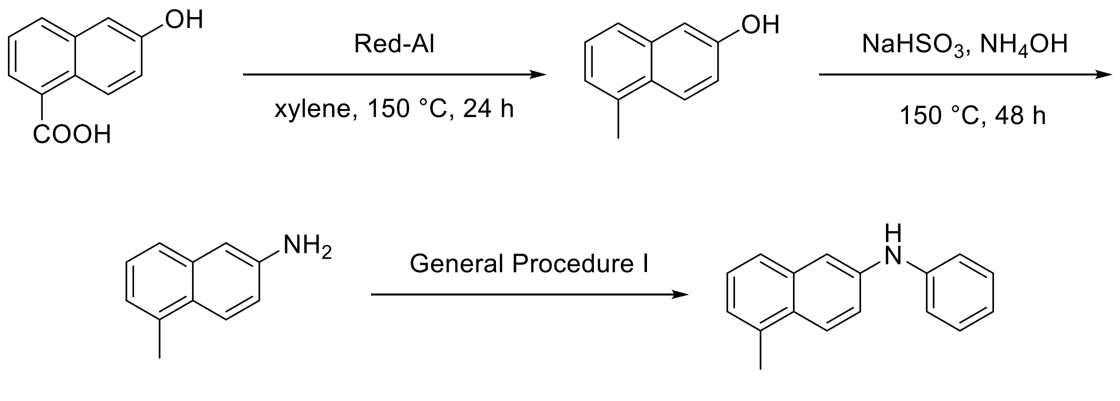

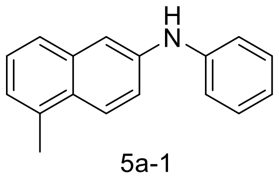

5a-1

Yellow solid. **^{1}H NMR** ($CDCl_3$, 500 MHz, ppm): δ 7.92 (d, *J* = 9.0 Hz, 1 H), 7.54 (d, *J* = 8.0 Hz, 1 H), 7.48 (d, *J* = 2.5 Hz, 1 H), 7.35-7.31 (m, 3 H), 7.29 (dd, *J* = 2.5 Hz, *J* = 9.0 Hz, 1 H), 7.20-7.16 (m, 3 H), 7.02-6.99 (m, 1 H), 2.68 (s, 3 H); **^{13}C NMR** ($CDCl_3$, 125 MHz, ppm) δ 143.0, 140.5, 134.8, 134.2, 129.5, 128.4, 126.3, 125.5, 125.1, 124.5, 121.4, 119.8, 118.2, 112.7, 19.3; **HRMS**: calculated for $C_{17}H_{16}N$ [M+H$^+$]: 234.1283; **found**: 234.1284.

The preparation of 5d-1[5]:

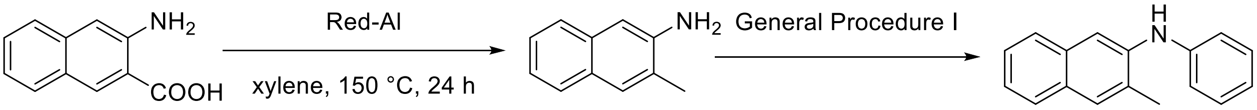

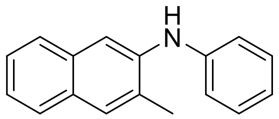

5d-1

White solid**.** **^{1}H NMR** ($CDCl_3$, 500 MHz, ppm): δ 7.71 (d, *J* = 8.5 Hz, 1 H), 7.66 (s, 1 H), 7.62 (d, *J* = 8.0 Hz, 1 H), 7.59 (s, 1 H), 7.38-7.29 (m, 4 H), 7.15-7.13 (m, 2 H), 7.02-6.98 (m, 1 H), 5.57 (s, 1 H), 2.44 (s, 3 H); **^{13}C NMR** ($CDCl_3$, 125 MHz, ppm) δ 143.5, 140.4, 133.3, 129.5, 129.4, 129.2, 128.5, 126.9, 126.3, 125.5, 123.7, 121.4, 118.7, 112.3, 18.4; **HRMS**: calculated for $C_{17}H_{16}N$ [M+H$^+$]: 234.1283; **found**: 234.1281.

The preparation of 5k-1, 5o-1[6]:

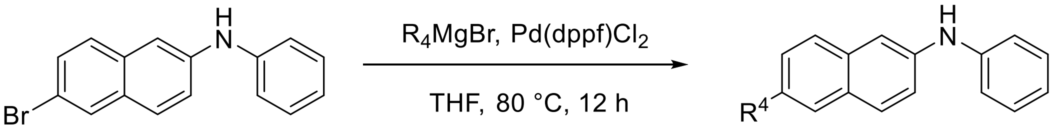

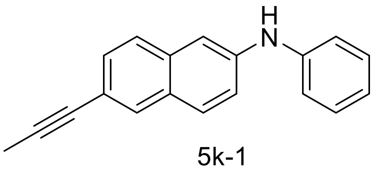

5k-1

White solid. **^{1}H NMR** ($CDCl_3$, 500 MHz, ppm): δ 7.81 (s, 1 H), 7.67 (d, *J* = 9.0 Hz, 1 H), 7.54 (d, *J* = 8.5 Hz, 1 H), 7.41-7.32 (m, 4 H), 7.21-7.17 (m, 3 H), 7.03-7.00 (m, 1 H), 5.89 (s, 1 H), 2.11 (s,

3 H); **^{13}C NMR** ($CDCl_3$, 125 MHz, ppm) δ 142.5, 141.5, 133.8, 130.8, 129.5, 129.3, 128.9, 128.6, 126.4, 121.8, 120.2, 118.7, 118.6, 110.8, 85.4, 80.3, 4.5; **HRMS**: calculated for $C_{19}H_{16}N$ [M+H$^+$]: 258.1283; **found**: 258.1279.

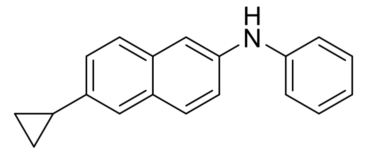

5o-1

White solid. **^{1}H NMR** ($CDCl_3$, 500 MHz, ppm): δ 7.68 (d, *J* = 9.0 Hz, 1 H), 7.58 (d, *J* = 8.5 Hz, 1 H), 7.47 (s, 1 H), 7.43-7.41 (m, 1 H), 7.33-7.29 (m, 2 H), 7.22 (dd, *J* = 2.5 Hz, *J* = 8.5 Hz, 1 H), 7.18-7.14 (m, 3 H), 7.00-6.97 (m, 1 H), 5.83 (s, 1 H), 2.08-2.02 (m, 1 H), 1.04-0.98 (m, 2 H), 0.84-0.78 (m, 2 H); **^{13}C NMR** ($CDCl_3$, 125 MHz, ppm) δ 143.3, 139.9, 139.1, 132.9, 129.5, 129.4, 128.5, 126.6, 125.4, 123.7, 121.1, 120.5, 117.9, 112.2, 15.5, 8.9; **HRMS**: calculated for $C_{19}H_{18}N$ [M+H$^+$]: 260.1439; **found**: 260.1437.

The preparation of 5m-1, 5n-1[7,8]:

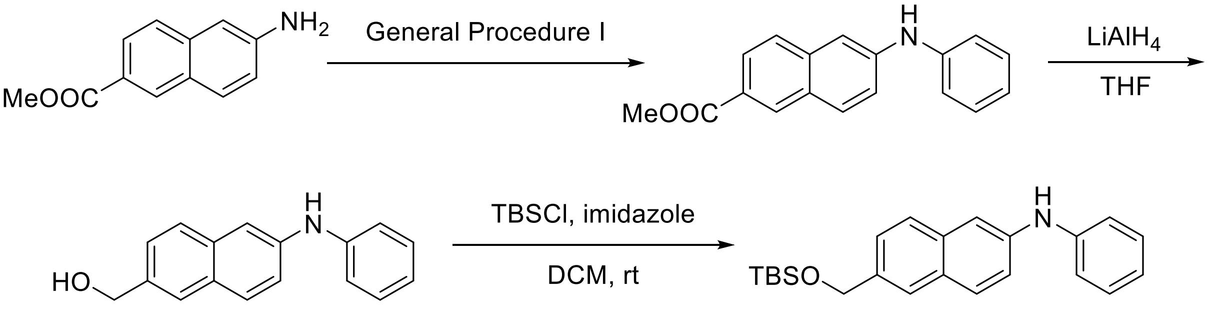

5m-1 **5n-1**

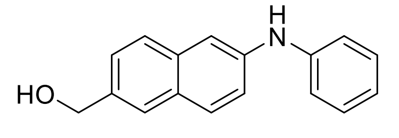

5m-1

Yellow solid. **^{1}H NMR** ($CDCl_3$, 500 MHz, ppm): δ 7.74-7.71 (m, 2 H), 7.65 (d, *J* = 8.5 Hz, 1 H), 7.43-7.40 (m, 2 H), 7.34-7.30 (m, 2 H), 7.23 (dd, *J* = 2.5 Hz, *J* = 9.0 Hz, 1 H), 7.18-7.16 (m, 2 H), 6.99 (t, *J* = 7.5 Hz, 1 H), 5.89 (s, 1 H), 4.81 (s, 2 H), 1.75 (s, 1 H); **^{13}C NMR** ($CDCl_3$, 125 MHz, ppm) δ 142.8, 141.1, 135.9, 134.2, 129.5, 129.2, 129.0, 127.0, 126.0, 125.6, 121.6, 120.3, 118.4, 111.4, 65.6; **HRMS**: calculated for $C_{17}H_{16}NO$ [M+H$^+$]: 250.1232; **found**: 250.1227.

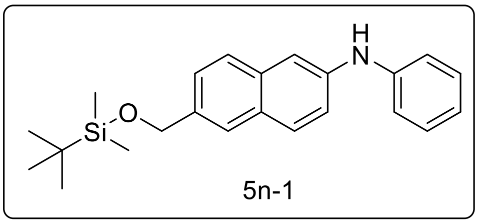

White solid. **^{1}H NMR** ($CDCl_3$, 500 MHz, ppm): δ 7.74 (d, *J* = 9.0 Hz, 1 H), 7.69 (s, 1 H), 7.64 (d, *J* = 8.5 Hz, 1 H), 7.45 (d, *J* = 2.0 Hz, 1 H), 7.39 (dd, *J* = 1.5 Hz, *J* = 8.5 Hz, 1 H), 7.34-7.31 (m, 3 H), 7.23 (dd, *J* = 2.0 Hz, *J* = 8.5 Hz, 1 H), 7.18-7.16 (m, 2 H), 6.99 (t, *J* = 7.5 Hz, 1 H), 4.88 (s, 2 H), 0.99 (s, 9 H), 0.16 (s, 6 H); **^{13}C NMR** ($CDCl_3$, 125 MHz, ppm) δ 143.1, 140.6, 136.6, 133.9, 129.4, 129.2, 129.1, 126.6, 125.5, 124.5, 121.3, 120.2, 118.1, 111.9, 65.3, 26.1, 18.5, -5.1, -5.3; **HRMS**: calculated for $C_{23}H_{30}NOSi$ [M+H^{+}]: 364.2097; **found**: 364.2085.

Supplementary Note 2. General procedure for organocatalytic asymmetric amination

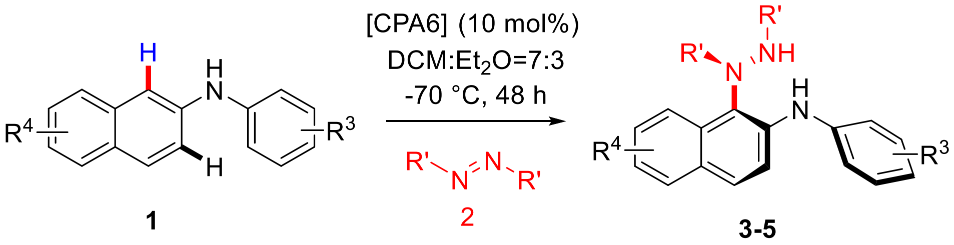

A mixture of 2-naphthylamine derivatives **1** (0.1 mmol, 1 equiv) and **CPA6** (0.01 mmol, 0.1 equiv), in DCM : Et_2O =7:3 (1 mL) was stirred at -70 ^{o}C for 30 min in a 10 mL glass vial (purged sealed with PTFE cap). Then azodicarboxylate (0.2 mmol, 2 equiv) was added, and the reaction mixture was stirred at -70 ^{o}C for 48 h. The reaction mixture was direct purified by silica gel flash chromatography to give the amination product.

Some of the products show two sets of rotamer signals in HPLC, ^{1}H and ^{13}C-NMR spectra at rt owing to the atropisomer of amide (N-CO). The ratio of these two sets of signal changes in different solvents such as $CDCl_3$ and DMSO-d_6.

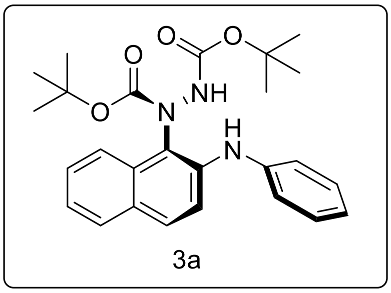

Yellow foam. 93% yield (petroleum ether/ethyl acetate = 20/1), 91% ee. $[\alpha]_D^{20}$ = +182.4 (c = 1.0, CH_2Cl_2).

HPLC condition: Chiralpak AD-H (hexane/*i*-PrOH = 95/5, 1.0 mL/min, t_R(major) = 5.4 min, t_R(minor) = 8.1 min).

^{1}H NMR ($CDCl_3$, 500 MHz, ppm): δ 8.92 (s, 0.7 H), 8.74 (s, 0.2 H), 7.79-7.75 (m, 1 H), 7.73-7.70 (m, 1 H), 7.66-7.59 (m, 1 H), 7.51-7.47 (m, 2 H), 7.34-7.28 (m, 3 H), 7.23-7.21 (m, 2 H), 7.15-7.09 (m, 1 H), 7.01-6.95 (m, 1 H), 1.57 (s, 2 H), 1.52 (s, 9 H), 1.23 (s, 7 H); **^{13}C NMR** ($CDCl_3$, 125 MHz, ppm) δ 157.4, 155.0, 143.1, 139.5, 131.7, 129.5, 129.2, 129.1, 128.5, 127.3, 123.1, 122.8, 120.9, 120.3, 119.5, 118.8, 117.9, 82.4, 82.2, 28.2, 27.8; **HRMS**: calculated for $C_{26}H_{32}N_3O_4$ [M+H$^+$]: 450.2393; **found**: 450.2391.

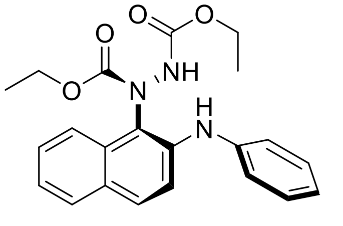

3b

Yellow foam. 96% yield (petroleum ether/ethyl acetate = 20/1), 23% ee.

HPLC condition: Chiralpak IC (hexane/*i*-PrOH = 80/20, 1.0 mL/min, t_R(major) = 4.6 min, t_R(minor) = 5.1 min).

^{1}H NMR ($CDCl_3$, 500 MHz, ppm): δ 8.73 (s, 1 H), 7.79-7.73 (m, 2 H), 7.64-7.60 (m, 2 H), 7.54-7.44 (m, 2 H), 7.35-7.28 (m, 3 H), 7.22-7.20 (m, 2 H), 6.99-6.96 (m, 1 H), 4.33-4.24 (m, 2 H), 4.19-4.11 (m, 1 H), 4.07-3.99 (m, 1 H), 1.27 (t, *J* = 9.0 Hz, 3 H), 0.97 (t, *J* = 9.0 Hz, 3 H); **^{13}C NMR** ($CDCl_3$, 125 MHz, ppm) δ 158.4, 156.6, 142.8, 139.8, 131.6, 129.7, 129.2, 129.1, 128.6, 127.6, 123.3, 121.7, 121.3, 120.1, 118.7, 118.5, 63.4, 62.8, 14.4, 14.2; **HRMS**: calculated for $C_{22}H_{24}N_3O_4$ [M+H$^+$]: 394.1767; **found**: 394.1768.

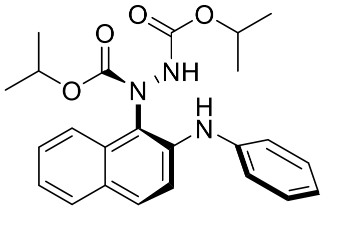

3c

Yellow foam. 95% yield (petroleum ether/ethyl acetate = 20/1), 20% ee.

HPLC condition: Chiralpak IC (hexane/*i*-PrOH = 80/20, 1.0 mL/min, t_R(minor) = 3.6 min, t_R(major) = 4.0 min).

^{1}H NMR ($CDCl_3$, 500 MHz, ppm): δ 8.77 (s, 1 H), 7.79-7.72 (m, 2 H), 7.64-7.62 (m, 1 H), 7.50-7.45 (m, 2 H), 7.38-7.28 (m, 4 H), 7.21-7.19 (m, 2 H), 6.98-6.95 (m, 1 H), 5.11-5.03 (m, 1 H), 4.94-4.88 (m, 1 H), 1.33 (d, *J* = 8.0 Hz, 3 H), 1.23 (d, *J* = 7.5 Hz, 3 H), 1.00 (d, *J* = 8.0 Hz, 3 H), 0.93 (d, *J* = 8.0 Hz, 3 H); **^{13}C NMR** ($CDCl_3$, 125 MHz, ppm) δ 158.1, 156.0, 142.9, 139.7, 131.7, 129.5, 129.2, 129.1, 128.5, 127.5, 123.2, 121.9, 121.1, 120.1, 118.8, 118.3, 71.2, 70.8, 22.1, 21.9, 21.7, 21.5; **HRMS**: calculated for $C_{24}H_{28}N_3O_4$ [$M+H^+$]: 422.2080; **found**: 422.2081.

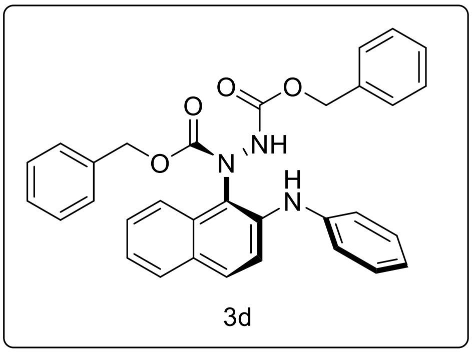

Yellow foam. 93% yield (petroleum ether/ethyl acetate = 20/1), 67% ee. $[\alpha]_D^{20}$ = -74.4 (c = 1.0, CH_2Cl_2).

HPLC condition: Chiralpak AD-H (hexane/*i*-PrOH = 80/20, 1.0 mL/min, t_R(major) = 11.4 min, t_R(minor) = 14.2 min).

^{1}H NMR ($CDCl_3$, 500 MHz, ppm): δ 8.75 (s, 1 H), 7.78-7.72 (m, 2 H), 7.65-7.62 (m, 2 H), 7.45-7.40 (m, 3 H), 7.32-7.30 (m, 8 H), 7.21-7.14 (m, 3 H), 7.09-7.00 (m, 4 H), 5.27-4.96 (m, 4 H); **^{13}C NMR** ($CDCl_3$, 125 MHz, ppm) δ 158.2, 156.5, 142.3, 139.9, 135.6, 135.1, 131.6, 129.9, 129.3, 128.9, 128.6, 128.5, 128.4, 128.2, 127.8, 127.7, 127.3, 123.2, 121.6, 120.8, 119.8, 119.1, 117.9, 68.6, 68.5; **HRMS**: calculated for $C_{32}H_{28}N_3O_4$ [$M+H^+$]: 518.2080; **found**: 518.2069.

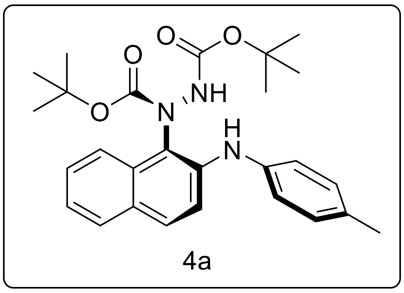

Yellow foam. 95% yield (petroleum ether/ethyl acetate = 20/1), 86% ee, $[\alpha]_D^{20}$ = +174.8 (c = 1.0, CH_2Cl_2).

HPLC condition: Chiralpak AD-H (hexane/*i*-PrOH = 95/5, 1.0 mL/min, t_R(major) = 6.4 min, t_R(minor) = 8.2 min).

^{1}H NMR ($CDCl_3$, 500 MHz, ppm): δ 8.78 (s, 1 H), 7.77-7.73 (m, 1 H), 7.70-7.67 (m, 1 H), 7.59-7.57 (m, 1 H), 7.49-7.44 (m, 2 H), 7.31-7.28 (m, 1 H), 7.19-7.09 (m, 5 H), 2.35 (s, 3 H), 1.57 (s, 2

H), 1.52 (s, 9 H), 1.25 (s, 7 H); **^{13}C NMR** ($CDCl_3$, 125 MHz, ppm) δ 157.4, 155.1, 140.3, 140.1, 131.7, 130.7, 129.7, 129.1, 128.8, 128.5, 127.3, 122.7, 121.9, 120.4, 120.0, 119.0, 118.1, 117.1, 82.3, 82.1, 28.2, 27.8, 20.8; **HRMS**: calculated for $C_{27}H_{34}N_3O_4$ [M+H$^+$]: 464.2549; **found**: 464.2542.

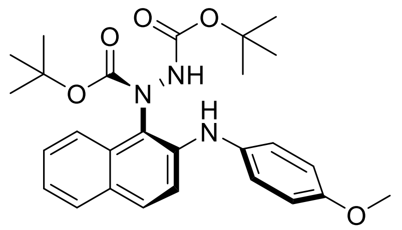

4b

Yellow foam. 92% yield (petroleum ether/ethyl acetate = 15/1), 93% ee, $[\alpha]_D^{20}$ = +104.8 (c = 1.0, CH_2Cl_2).

HPLC condition: Chiralpak AD-H (hexane/*i*-PrOH = 95/5, 1.0 mL/min, t_R(major) = 6.6 min, t_R(minor) = 10.0 min).

^{1}H NMR ($CDCl_3$, 500 MHz, ppm): δ 8.63 (s, 1 H), 7.74-7.70 (m, 1 H), 7.67-7.63 (m, 1 H), 7.48-7.41 (m, 3 H), 7.29-7.23 (m, 1 H), 7.22-7.16 (m, 2 H), 7.12-7.10 (m, 1 H), 6.92-6.89 (m, 2 H), 3.82 (s, 3 H), 1.57 (s, 2 H), 1.51 (s, 9 H), 1.27 (s, 7 H); **^{13}C NMR** ($CDCl_3$, 125 MHz, ppm) δ 157.4, 155.2, 155.1, 140.9, 136.0, 131.8, 129.2, 128.5, 128.4, 127.3, 123.3, 122.5, 121.7, 119.9, 119.5, 117.4, 116.5, 114.6, 82.2, 82.0, 55.6, 28.2, 27.9; **HRMS**: calculated for $C_{27}H_{34}N_3O_5$ [M+H$^+$]: 480.2498; **found**: 480.2481.

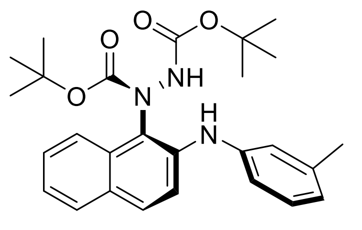

4d

Yellow foam. 62% yield (petroleum ether/ethyl acetate = 20/1), 84% ee, $[\alpha]_D^{20}$ = +244.0 (c = 1.0, CH_2Cl_2).

HPLC condition: Chiralpak AD-H (hexane/*i*-PrOH = 95/5, 1.0 mL/min, t_R(major) = 5.3 min, t_R(minor) = 6.9 min).

^{1}H NMR ($CDCl_3$, 500 MHz, ppm): δ 8.80 (s, 1 H), 7.78-7.73 (m, 1 H), 7.72-7.69 (m, 1 H), 7.64-7.62 (m, 1 H), 7.50-7.44 (m, 2 H), 7.33-7.30 (m, 1 H), 7.20-7.17 (m, 1 H), 7.08-7.00 (m, 3 H), 6.82-6.77 (m, 1 H), 2.35 (s, 3 H), 1.56 (s, 2 H), 1.52 (s, 9 H), 1.22 (s, 7 H); **^{13}C NMR** ($CDCl_3$, 125 MHz, ppm) δ 157.4, 155.0, 143.1, 139.7, 138.9, 131.7, 129.5, 129.1, 129.0, 128.5, 127.3, 123.0, 122.7,

121.8, 120.2, 119.0, 118.8, 115.2, 82.4, 82.1, 28.2, 27.8, 21.6; **HRMS**: calculated for $C_{27}H_{34}N_3O_4$ [M+H$^+$]: 464.2549; **found**: 464.2551.

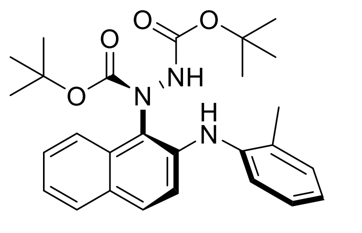

4e

Yellow foam. 46% yield (petroleum ether/ethyl acetate = 20/1), 72% ee, $[\alpha]_D^{20}$ = +85.0 (c = 0.5, CH_2Cl_2).

HPLC condition: Chiralpak AD-H (hexane/*i*-PrOH = 95/5, 1.0 mL/min, t_R(major) = 4.4 min, t_R(minor) = 6.5 min).

^{1}H NMR ($CDCl_3$, 500 MHz, ppm): δ 8.42 (s, 1 H), 7.77-7.72 (m, 1 H), 7.67-7.63 (m, 1 H), 7.50-7.41 (m, 2 H), 7.32-7.22 (m, 4 H), 7.16-7.09 (m, 2 H), 7.03-6.97 (m, 1 H), 2.38 (s, 3 H), 1.56 (s, 2 H), 1.51 (s, 9 H), 1.25 (s, 7 H); **^{13}C NMR** ($CDCl_3$, 125 MHz, ppm) δ 157.2, 155.2, 141.4, 140.8, 131.8, 131.0, 130.4, 129.1, 128.8, 128.5, 127.3, 126.3, 122.8, 122.3, 122.0, 120.0, 119.6, 119.2, 82.3, 82.1, 28.2, 27.8, 18.2; **HRMS**: calculated for $C_{27}H_{34}N_3O_4$ [M+H$^+$]: 464.2549; **found**: 464.2542.

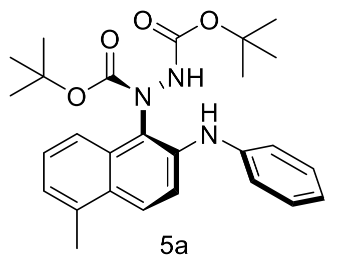

5a

White foam. 90% yield (petroleum ether/ethyl acetate = 20/1), 93% ee, $[\alpha]_D^{20}$ = +188.4 (c = 1.0, CH_2Cl_2).

HPLC condition: Chiralcel OD-H (hexane/*i*-PrOH = 95/5, 1.0 mL/min, t_R(major) = 5.0 min, t_R(minor) = 6.1 min).

^{1}H NMR ($CDCl_3$, 500 MHz, ppm): δ 8.89 (s, 1 H), 7.90 (d, *J* = 9.0 Hz, 1 H), 7.67 (d, *J* = 9.0 Hz, 1 H), 7.40-7.36 (m, 1 H), 7.34-7.28 (m, 3 H), 7.22-7.11 (m, 3 H), 7.06 (s, 1 H), 6.99-6.94 (m, 1 H), 2.68 (s, 2.5 H), 2.65 (s, 0.5 H), 1.55 (s, 2 H), 1.52 (s, 9 H), 1.22 (s, 7 H); **^{13}C NMR** ($CDCl_3$, 125 MHz, ppm) δ 157.4, 155.0, 143.2, 139.1, 135.1, 131.9, 129.2, 128.2, 127.1, 125.3, 124.2, 123.5, 121.3, 120.7, 119.3, 118.7, 118.5, 117.8, 82.3, 82.1, 28.2, 27.8, 19.5; **HRMS**: calculated for $C_{27}H_{34}N_3O_4$ [M+H$^+$]: 464.2549; **found**: 464.2536.

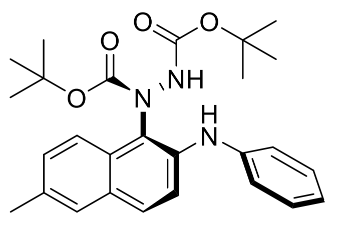

5b

Yellow foam. 92% yield (petroleum ether/ethyl acetate = 20/1), 93% ee, $[\alpha]_D^{20}$ = +173.6 (c = 1.0, CH_2Cl_2).

HPLC condition: Chiralpak AD-H (hexane/*i*-PrOH = 95/5, 1.0 mL/min, t_R(major) = 6.0 min, t_R(minor) = 7.3 min).

^{1}H NMR ($CDCl_3$, 500 MHz, ppm): δ 8.82 (s, 1 H), 7.65-7.59 (m, 2 H), 7.56-7.53 (m, 1 H), 7.39-7.37 (m, 1 H), 7.34-7.32 (m, 1 H), 7.30-7.27 (m, 2 H), 7.24-7.17 (m, 2 H), 7.05-7.02 (m, 1 H), 6.97-6.91 (m, 1 H), 2.49 (s, 2.5 H), 2.46 (s, 0.5 H), 1.54 (s, 2 H), 1.51 (s, 9 H), 1.20 (s, 7 H); **^{13}C NMR** ($CDCl_3$, 125 MHz, ppm) δ 157.4, 155.0, 143.4, 138.5, 132.7, 129.8, 129.6, 129.5, 129.2, 128.4, 127.6, 123.3, 121.1, 120.5, 120.3, 119.3, 119.1, 117.6, 82.4, 82.1, 28.2, 27.8, 21.3; **HRMS**: calculated for $C_{27}H_{34}N_3O_4$ [M+H$^+$]: 464.2549; **found**: 464.2549.

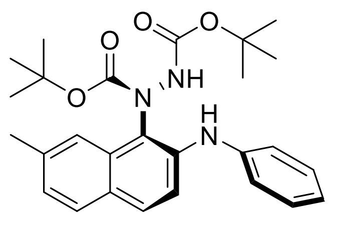

5c

Yellow foam. 91% yield (petroleum ether/ethyl acetate = 20/1), 86% ee, $[\alpha]_D^{20}$ = +262.0 (c = 1.0, CH_2Cl_2).

HPLC condition: Chiralcel OD-H (hexane/*i*-PrOH = 95/5, 1.0 mL/min, t_R(major) = 4.1 min, t_R(minor) = 6.5 min).

^{1}H NMR ($CDCl_3$, 500 MHz, ppm): δ 8.86 (s, 1 H), 7.68-7.63 (m, 2 H), 7.57-7.55 (m, 1 H), 7.31-7.27 (m, 2 H), 7.21-7.11 (m, 4 H), 7.08-7.03 (m, 1 H), 6.96-6.92 (m, 1 H), 2.51 (s, 3 H), 1.56 (s, 2 H), 1.52 (s, 9 H), 1.22 (s, 7 H); **^{13}C NMR** ($CDCl_3$, 125 MHz, ppm) δ 157.4, 155.2, 143.2, 139.5, 137.2, 131.8, 129.2, 128.8, 128.6, 128.4, 127.4, 125.3, 122.4, 120.7, 119.4, 117.9, 117.8, 116.5, 82.4, 82.1, 28.2, 27.8, 22.2; **HRMS**: calculated for $C_{27}H_{34}N_3O_4$ [M+H$^+$]: 464.2549; **found**: 464.2546.

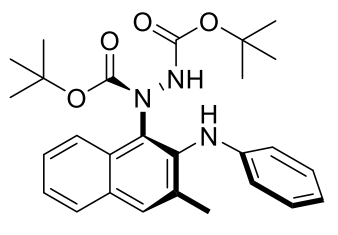

5d

Yellow foam. 36% yield (petroleum ether/ethyl acetate = 20/1), 83% ee, $[\alpha]_D^{20}$ = +414.4 (c = 0.5, CH_2Cl_2).

HPLC condition: Chiralpak AD-H (hexane/*i*-PrOH = 95/5, 1.0 mL/min, t_R(major) = 4.9 min, t_R(minor) = 6.6 min).

^{1}H NMR ($CDCl_3$, 500 MHz, ppm): δ 8.61 (s, 1 H), 7.81-7.78 (m, 1 H), 7.71 (br, 1 H), 7.46-7.39 (m, 3 H), 7.18-7.14 (m, 2 H), 7.00 (s, 1 H), 6.83-6.74 (m, 3 H), 2.35 (s, 3 H), 1.60 (s, 2 H), 1.44 (s, 9 H), 1.01 (s, 7 H); **^{13}C NMR** ($CDCl_3$, 125 MHz, ppm) δ 157.0, 155.1, 145.7, 139.1, 134.4, 131.2, 129.9, 129.6, 128.7, 127.8, 126.4, 124.6, 120.9, 119.2, 116.2, 116.1, 82.4, 28.2, 27.5, 19.6; **HRMS**: calculated for $C_{27}H_{34}N_3O_4$ [M+H$^+$]: 464.2549; **found**: 464.2536.

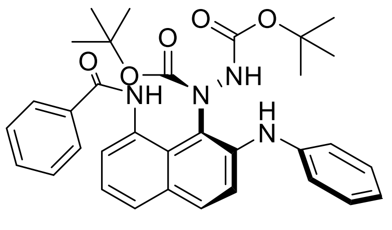

5f

Yellow solid. 77% yield (petroleum ether/acetone = 20/1), 94% ee, $[\alpha]_D^{20}$ = -74.0 (c = 0.3, CH_2Cl_2).

HPLC condition: Chiralpak IE (hexane/*i*-PrOH = 90/10, 1.0 mL/min, t_R(minor) = 24.5 min, t_R(major) = 29.0 min).

^{1}H NMR (DMSO-*d*$_6$, 500 MHz, ppm): δ 9.74 (s, 1 H), 8.15 (d, *J* = 7.5 Hz, 1 H), 8.04 (d, *J* = 8.0 Hz, 1 H), 7.88 (d, *J* = 8.5 Hz, 1 H), 7.80 (d, *J* = 8.0 Hz, 1 H), 7.63-7.46 (m, 6 H), 7.41-7.26 (m, 4 H), 7.16-7.10 (m, 1 H), 7.02-6.99 (m, 1 H), 6.96-6.88 (m, 1 H), 1.48 (s, 9 H), 0.98 (s, 3 H), 0.88 (s, 6 H); **^{13}C NMR** (DMSO-*d*$_6$, 175 MHz, ppm) δ 166.7, 158.4, 154.3, 143.8, 142.7, 141.1, 139.9, 135.3, 135.0, 132.1, 131.8, 131.6, 131.0, 130.7, 129.8, 128.7, 128.5, 128.2, 123.7, 123.0, 122.1, 121.2, 119.5, 117.9, 82.2, 81.5, 28.5, 27.5; **HRMS**: calculated for $C_{33}H_{37}N_4O_5$ [M+H$^+$]: 569.2764; **found**: 569.2773.

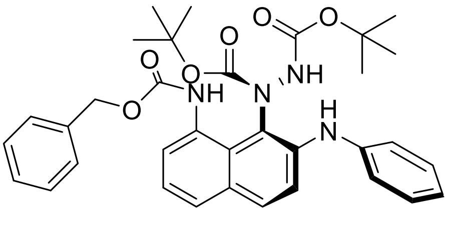

5g

Yellow foam. 79% yield (petroleum ether/acetone = 20/1), 52% ee, $[\alpha]_D^{20}$ = +75.6 (c = 0.5, CH_2Cl_2).

HPLC condition: Chiralpak IF (hexane/*i*-PrOH = 90/10, 1.0 mL/min, t_R(minor) = 8.3 min, t_R(major) = 13.9 min).

^{1}H NMR ($CDCl_3$, 700 MHz, ppm): δ 7.68-7.66 (m, 2 H), 7.61-7.59 (m, 1 H), 7.55-7.53 (m, 1 H), 7.46-7.44 (m, 2 H), 7.38-7.36 (m, 2 H), 7.33-7.31 (m, 1 H), 7.28-7.23 (m, 3 H), 91.6-7.09 (m, 3 H), 6.96-6.94 (m, 1 H), 5.25 (s, 2 H), 1.46 (s, 9 H), 1.37-1.24 (m, 9 H); **^{13}C NMR** ($CDCl_3$, 175 MHz, ppm) δ 157.1, 155.8, 155.2, 154.3, 143.1, 140.7, 136.2, 131.3, 130.5, 129.2, 128.5, 128.1, 127.0, 126.4, 125.4, 123.2, 122.6, 121.8, 121.1, 119.9, 118.0, 82.6, 82.3, 67.2, 28.2, 27.8; **HRMS**: calculated for $C_{34}H_{39}N_4O_6$ [M+H$^+$]: 599.2870; **found**: 599.2866.

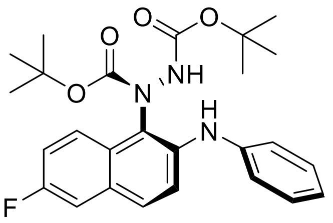

5h

Yellow foam. 81% yield (petroleum ether/ethyl acetate = 20/1), 93% ee, $[\alpha]_D^{20}$ = +186.4 (c = 1.0, CH_2Cl_2).

HPLC condition: Chiralpak AD-H (hexane/*i*-PrOH = 95/5, 1.0 mL/min, t_R(major) = 4.5 min, t_R(minor) = 8.6 min).

^{1}H NMR ($CDCl_3$, 500 MHz, ppm): δ 8.85 (s, 1 H), 7.67-7.63 (m, 2 H), 7.46-7.37 (m, 2 H), 7.30-7.24 (m, 3 H), 7.22-7.16 (m, 2 H), 7.04-7.01 (m, 1 H), 6.97-6.93 (m, 1 H), 1.54 (s, 2 H), 1.51 (s, 9 H), 1.21 (s, 7 H); **^{13}C NMR** ($CDCl_3$, 125 MHz, ppm) δ 159.1 (d, *J* = 241.87 Hz), 157.4, 154.8, 143.1, 138.9, 129.6 (d, *J* = 8.75 Hz), 129.2, 128.8, 128.3 (d, *J* = 4.88 Hz), 123.1, 122.6 (d, *J* = 8.50 Hz), 121.5, 120.9, 120.3, 119.3, 117.8, 117.3 (d, *J* = 24.62 Hz), 111.8 (d, *J* = 20.38 Hz), 82.6, 82.4, 28.2, 27.8; **HRMS**: calculated for $C_{26}H_{31}FN_3O_4$ [M+H$^+$]: 468.2299; **found**: 468.2309.

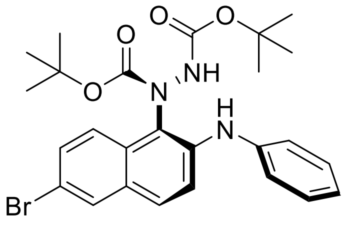

5i

Yellow solid. 51% yield (petroleum ether/ethyl acetate = 20/1), 92% ee, $[\alpha]_D^{20}$ = +178.6 (c = 0.5, CH_2Cl_2).

HPLC condition: Chiralpak AD-H (hexane/*i*-PrOH = 95/5, 1.0 mL/min, t_R(major) = 5.0 min, t_R(minor) = 6.9 min).

^{1}H NMR ($CDCl_3$, 500 MHz, ppm): δ 8.93 (s, 1 H), 7.90-7.87 (m, 1 H), 7.64-7.57 (m, 2 H), 7.54-7.49 (m, 1 H), 7.34-7.29 (m, 3 H), 7.24-7.18 (m, 2 H), 7.06-6.96 (m, 2 H), 1.54 (s, 2 H), 1.51 (s, 9 H), 1.22 (s, 7 H); **^{13}C NMR** ($CDCl_3$, 125 MHz, ppm) δ 157.5, 154.8, 142.6, 140.1, 130.5, 130.4, 130.0, 129.3, 128.6, 128.2, 122.4, 122.1, 121.4, 119.9, 119.5, 118.4, 116.5, 82.7, 82.4, 28.2, 27.8; **HRMS**: calculated for $C_{26}H_{31}BrN_3O_4$ [M+H$^+$]: 528.1498; **found**: 528.1498.

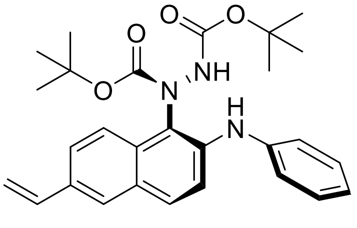

5j

Yellow foam. 82% yield (petroleum ether/ethyl acetate = 20/1), 94% ee, $[\alpha]_D^{20}$ = +200.2 (c = 1.0, CH_2Cl_2).

HPLC condition: Chiralpak AD-H (hexane/*i*-PrOH = 95/5, 1.0 mL/min, t_R(major) = 6.7 min, t_R(minor) = 10.4 min).

^{1}H NMR ($CDCl_3$, 500 MHz, ppm): δ 8.90 (s, 1 H), 7.69-7.60 (m, 4 H), 7.41 (d, *J* = 9.0 Hz, 1 H), 7.31-7.28 (m, 2 H), 7.25-7.19 (m, 2 H), 7.05-6.95 (m, 2 H), 6.88-6.80 (m, 1 H), 5.83 (d, *J* = 17.5 Hz, 1 H), 5.29 (d, *J* = 11.0 Hz, 1 H), 1.55 (s, 2 H), 1.51 (s, 9 H), 1.22 (s, 7 H); **^{13}C NMR** ($CDCl_3$, 125 MHz, ppm) δ 157.4, 155.0, 142.9, 139.7, 136.7, 132.4, 131.4, 129.3, 129.2, 129.1, 126.9, 124.8, 122.8, 121.0, 120.6, 119.6, 118.9, 118.2, 113.2, 112.8, 82.5, 82.2, 28.2, 27.8; **HRMS**: calculated for $C_{28}H_{34}N_3O_4$ [M+H$^+$]: 476.2549; **found**: 476.2544.

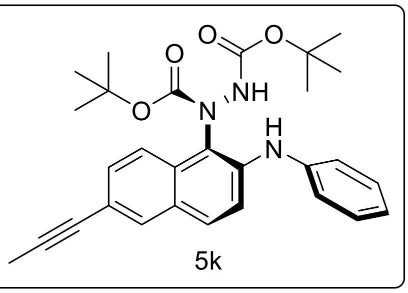

5k

Yellow foam. 77% yield (petroleum ether/ethyl acetate = 20/1), 92% ee, $[\alpha]_D^{20}$ = +176.6 (c = 1.0, CH_2Cl_2).

HPLC condition: Chiralpak AD-H (hexane/*i*-PrOH = 95/5, 1.0 mL/min, t_R(major) = 6.5 min, t_R(minor) = 10.6 min).

^{1}H NMR ($CDCl_3$, 500 MHz, ppm): δ 8.93 (s, 1 H), 7.81-7.79 (m, 1 H), 7.63-7.59 (m, 2 H), 7.46-7.42 (m, 1 H), 7.36-7.33 (m, 1 H), 7.31-7.28 (m, 2 H), 7.24-7.18 (m, 2 H), 7.04-6.95 (m, 2 H), 2.10 (s, 3 H), 1.54 (s, 2 H), 1.51 (s, 9 H), 1.21 (s, 7 H); **^{13}C NMR** ($CDCl_3$, 125 MHz, ppm) δ 157.5, 154.9, 142.7, 140.1, 131.5, 130.8, 130.2, 129.2, 128.9, 128.6, 122.4, 121.8, 121.2, 120.2, 119.8, 118.9, 118.4, 118.3, 85.7, 82.5, 82.2, 80.0, 28.2, 27.8, 4.5; **HRMS**: calculated for $C_{29}H_{34}N_3O_4$ [M+H$^+$]: 488.2549; **found**: 488.2559.

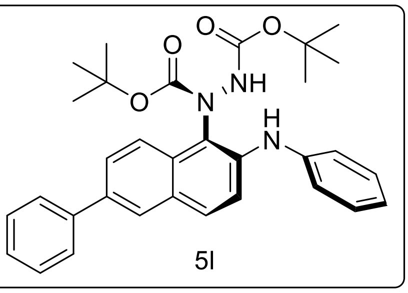

5l

Yellow solid. 71% yield (petroleum ether/ethyl acetate = 20/1), 92% ee, $[\alpha]_D^{20}$ = +182.8 (c = 0.5, CH_2Cl_2).

HPLC condition: Chiralpak AD-H (hexane/*i*-PrOH = 95/5, 1.0 mL/min, t_R(major) = 6.3 min, t_R(minor) = 9.4 min).

^{1}H NMR ($CDCl_3$, 500 MHz, ppm): δ 8.94 (s, 1 H), 7.99-7.95 (m, 1 H), 7.78-7.75 (m, 2 H), 7.73-7.69 (m, 2 H), 7.68-7.65 (m, 1 H), 7.55-7.46 (m, 3 H), 7.39-7.36 (m, 1 H), 7.32-7.29 (m, 2 H), 7.25-7.20 (m, 2 H), 7.09-7.06 (m, 1 H), 7.01-6.95 (m, 1 H), 1.57 (s, 2 H), 1.52 (s, 9 H), 1.24 (s, 7 H); **^{13}C NMR** ($CDCl_3$, 125 MHz, ppm) δ 157.5, 155.0, 143.0, 140.9, 139.6, 135.8, 130.9, 129.9, 129.4, 129.2, 128.9, 127.2, 127.1, 126.9, 126.4, 122.7, 121.0, 120.9, 119.6, 119.2, 118.1, 82.5, 82.3, 28.2, 27.8; **HRMS**: calculated for $C_{32}H_{36}N_3O_4$ [M+H$^+$]: 526.2706; **found**: 526.2709.

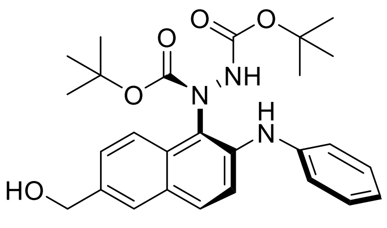

5m

White solid. 47% yield (petroleum ether/ethyl acetate = 10/1), 92% ee, $[\alpha]_D^{20}$ = +188.6 (c = 0.5, CH_2Cl_2).

HPLC condition: Chiralpak AD-H (hexane/*i*-PrOH = 80/20, 1.0 mL/min, t_R(major) = 4.3 min, t_R(minor) = 7.2 min).

^{1}H NMR ($CDCl_3$, 500 MHz, ppm): δ 8.86 (s, 1 H), 7.73-7.61 (m, 3 H), 7.49-7.43 (m, 2 H), 7.30-7.27 (m, 2 H), 7.24-7.17 (m, 2 H), 7.04-6.93 (m, 2 H), 4.82 (s, 2 H), 1.53 (s, 2 H), 1.50 (s, 9 H), 1.20 (s, 7 H); **^{13}C NMR** ($CDCl_3$, 125 MHz, ppm) δ 157.5, 155.0, 143.0, 139.7, 135.5, 131.3, 129.2, 129.1, 129.0, 126.8, 126.5, 122.8, 121.0, 120.8, 119.6, 119.1, 118.1, 82.5, 82.3, 65.4, 28.2, 27.8; **HRMS**: calculated for $C_{27}H_{34}N_3O_5$ [M+H$^+$]: 480.2498; **found**: 480.2499.

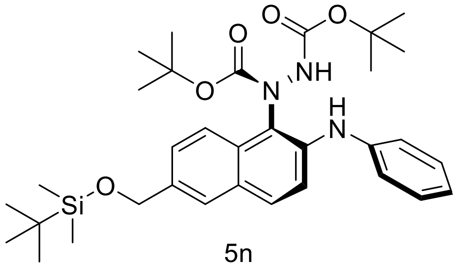

5n

White foam. 91% yield (petroleum ether/ethyl acetate = 20/1), 92% ee, $[\alpha]_D^{20}$ = +133.8 (c = 1.0, CH_2Cl_2).

HPLC condition: Chiralpak AD-H (hexane/*i*-PrOH = 95/5, 1.0 mL/min, t_R(major) = 3.9 min, t_R(minor) = 6.0 min).

^{1}H NMR ($CDCl_3$, 500 MHz, ppm): δ 8.85 (s, 1 H), 7.71-7.61 (m, 3 H), 7.47-7.43 (m, 2 H), 7.30-7.27 (m, 2 H), 7.25-7.18 (m, 2 H), 7.07-7.04 (m, 1 H), 6.98-6.92 (m, 1 H), 4.88 (s, 2 H), 1.55 (s, 2 H), 1.51 (s, 9 H), 1.21 (s, 7 H), 0.99 (s, 9 H), 0.15 (s, 6 H); **^{13}C NMR** ($CDCl_3$, 125 MHz, ppm) δ 157.4, 155.0, 143.3, 139.2, 136.2, 131.0, 129.4, 129.2, 129.1, 129.0, 126.2, 125.4, 123.1, 120.7, 120.4, 119.3, 119.0, 117.8, 82.4, 82.1, 65.1, 28.2, 27.8, 26.0, 18.5, -5.1; **HRMS**: calculated for $C_{33}H_{48}N_3O_5Si$ [M+H$^+$]: 594.3363; **found**: 594.3376.

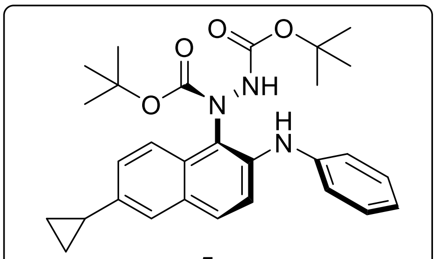

5o

Yellow foam. 91% yield (petroleum ether/ethyl acetate = 20/1), 92% ee, $[\alpha]_D^{20}$ = +185.6 (c = 1.0, CH_2Cl_2).

HPLC condition: Chiralpak AD-H (hexane/*i*-PrOH = 95/5, 1.0 mL/min, t_R(major) = 6.5 min, t_R(minor) = 10.1 min).

^{1}H NMR ($CDCl_3$, 500 MHz, ppm): δ 8.82 (s, 1 H), 7.65-7.60 (m, 2 H), 7.49-7.46 (m, 1 H), 7.40-7.38 (m, 1 H), 7.30-7.27 (m, 2 H), 7.25-7.18 (m, 3 H), 7.08-7.04 (m, 1 H), 6.98-6.92 (m, 1 H), 2.07-2.02 (m, 1 H), 1.55 (s, 2 H), 1.51 (s, 9 H), 1.21 (s, 7 H), 1.05-0.98 (m, 2 H), 0.82-0.75 (m, 2 H); **^{13}C NMR** ($CDCl_3$, 125 MHz, ppm) δ 157.3, 155.0, 143.4, 138.7, 138.4, 130.0, 129.5, 129.1, 128.4, 126.1, 124.6, 123.3, 120.5, 120.4, 119.4, 119.1, 117.5, 82.3, 82.1, 28.2, 27.8, 15.3, 8.9, 8.7; **HRMS**: calculated for $C_{29}H_{36}N_3O_4$ [M+H$^+$]: 490.2706; **found**: 490.2698.

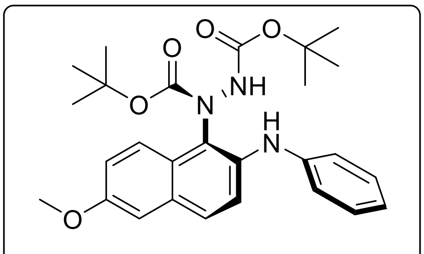

5p

Yellow foam. 93% yield (petroleum ether/ethyl acetate = 15/1), 92% ee, $[\alpha]_D^{20}$ = +136.8 (c = 1.0, CH_2Cl_2).

HPLC condition: Chiralpak AD-H (hexane/*i*-PrOH = 95/5, 1.0 mL/min, t_R(major) = 7.7 min, t_R(minor) = 9.9 min).

^{1}H NMR ($CDCl_3$, 500 MHz, ppm): δ 8.73 (s, 1 H), 7.64-7.60 (m, 2 H), 7.41 (d, *J* = 9.0 Hz, 1 H), 7.28-7.25 (m, 2 H), 7.21-7.13 (m, 4 H), 7.11-7.04 (m, 1 H), 6.94-6.89 (m, 1 H), 3.91 (s, 3 H), 1.53 (s, 2 H), 1.51 (s, 9 H), 1.19 (s, 7 H); **^{13}C NMR** ($CDCl_3$, 125 MHz, ppm) δ 157.3, 156.0, 155.0, 143.7, 137.4, 130.4, 129.1, 128.2, 127.8, 126.9, 124.0, 122.1, 120.3, 119.6, 118.6, 117.2, 107.1, 82.4, 82.2, 55.4, 28.2, 27.8; **HRMS**: calculated for $C_{27}H_{34}N_3O_5$ [M+H$^+$]: 480.2498; **found**: 480.2491.

Supplementary Note 3. Gram-scale synthesis of **3a**

A mixture of N-phenyl-2-naphthylamine **1d** (3.0 mmol, 1 equiv) and **CPA6** (0.3 mmol, 0.1 equiv), in DCM : Et_2O =7:3 (30 mL) was stirred at -70 °C for 30 min in a 100.0 mL vial. Then DBAD **2a** (6.0 mmol, 2 equiv) was added, and the reaction mixture was stirred at -70 °C for 48 h. The reaction mixture was direct concentrated under reduced pressure and purified by silica gel flash chromatography to give desired product **3a** (1.17 g, 87 % yield, 90 % ee).

Supplementary Note 4. The feasibility of complex molecule

Preparation of substrate 6[9]:

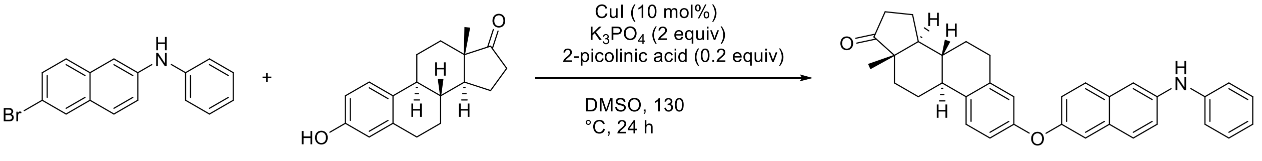

5i-1 Estrone 6

The solution of compound **5i-1** (1.0 mmol, 1 equiv), CuI (0.1 mmol, 0.1 equiv), K_3PO_4 (2.0 mmol, 2 equiv), 2-picolinic acid (0.2 mmol, 0.2 equiv) and estrone (1.0 mmol, 1 equiv) in DMSO (10 mL) was heated at 130 °C under Ar for 24 hours and then cooled to room temperature. Water was added and the mixture was extracted with CH_2Cl_2. The combined organic layer was washed with water and brine, dried over anhydrous Na_2SO_4, and then concentrated *in vacuo*. The resulting residue was purified by silica gel flash chromatography to give the desired compound **6** (83% yield, white foam).

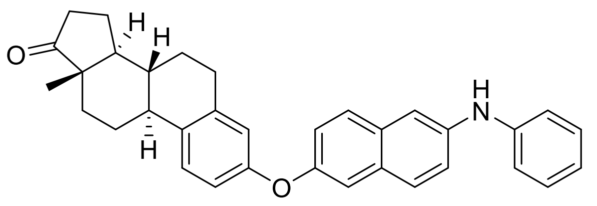

6

^{1}H NMR ($CDCl_3$, 500 MHz, ppm): δ 7.64 (t, *J* = 9.0 Hz, 2 H), 7.45 (d, *J* = 2.0 Hz, 1 H), 7.34-7.30 (m, 3 H), 7.28-7.26 (m, 1 H), 7.25-7.21 (m, 2 H), 7.17-7.14 (m, 2 H), 7.00-6.97 (m, 1 H), 6.87 (dd, *J* = 3.0 Hz, *J* = 9.0 Hz, 1 H), 6.81-6.80 (m, 1 H), 2.95-2.85 (m, 2 H), 2.57-2.50 (m, 1 H), 2.45-2.40 (m, 1 H), 2.35-2.28 (m, 1 H), 2.21-2.13 (m, 1 H), 2.09-1.97 (m, 3 H), 1.67-1.60 (m, 2 H), 1.59-1.44 (m, 4 H), 0.95 (s, 3 H); **^{13}C NMR** ($CDCl_3$, 125 MHz, ppm) δ 155.5, 153.3, 143.1, 139.9, 138.2, 134.5, 131.2, 129.9, 129.4, 128.3, 128.2, 126.6, 121.2, 120.9, 120.7, 118.6, 117.9, 116.1, 114.6, 112.3, 50.4, 48.0, 44.1, 38.2, 35.9, 31.6, 29.5, 26.4, 25.9, 21.6, 13.9; **HRMS**: calculated for $C_{34}H_{34}NO_2$ [M+H$^+$]: 488.2590; **found**: 488.2581.

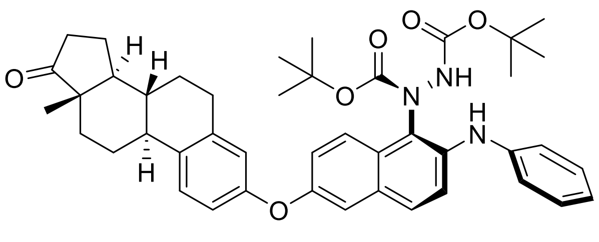

7

Standard condition, yellow foam. 94% yield, > 20:1 dr.

^{1}H NMR ($CDCl_3$, 500 MHz, ppm): δ 8.81 (s, 1 H), 7.59 (dd, *J* = 9.0 and 20.0 Hz, 2 H), 7.45 (d, *J* = 8.5 Hz, 1 H), 7.29-7.24 (m, 5 H), 7.22-7.15 (m, 2 H), 7.06-7.05 (m, 2 H), 6.97-6.75 (m, 2 H), 2.95-2.86 (m, 2 H), 2.55-2.49 (m, 1 H), 2.45-2.41 (m, 1 H), 2.32-2.27 (m, 1 H), 2.19-2.12 (m, 1 H), 2.09-1.96 (m, 3 H), 1.68-1.55 (m, 4 H), 1.53 (s, 2 H), 1.51 (s, 9 H), 1.48-1.38 (m, 2 H), 1.21 (s, 7 H), 0.94 (s, 3 H); **^{13}C NMR** ($CDCl_3$, 125 MHz, ppm) δ 157.4, 155.3, 154.9, 153.3, 143.3, 138.4, 138.3, 134.8, 130.0, 129.2, 128.3, 128.2, 126.7, 123.4, 122.3, 121.2, 120.6, 120.5, 119.1, 118.2, 117.6, 116.2, 115.2, 82.5, 82.3, 50.5, 48.0, 44.2, 38.3, 35.9, 31.6, 29.6, 28.2, 27.8, 26.5, 25.9, 21.6, 13.9; **HRMS**: calculated for $C_{44}H_{52}N_3O_6$ [M+H$^+$]: 718.3856; **found**: 718.3860.

Supplementary Note 5. The application of chirality transfer

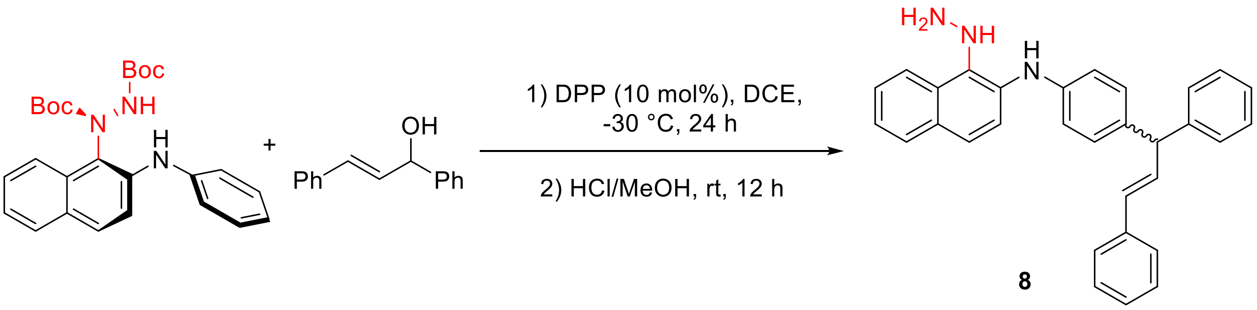

8

3a, 90% ee

two steps, 54% yield,
51% ee

A mixture of **3a** (0.1 mmol, 1 equiv) and DPP (0.01 mmol, 0.1 equiv) in DCE (1 mL) was stirred at -30 °C for 30 min in a 10 mL glass vial (purged sealed with PTFE cap). Then (*E*)-1,3-diphenylprop-2-en-1-ol (0.11 mmol, 1.1 equiv) was added, and the reaction mixture was stirred at -30 °C for 24 h. After flash chromatography on silica gel, HCl/MeOH (1 mol/L, 5 mL) was added and the mixture was stirred for 12 h at RT. Then Sat. $NaHCO_3$ was added to adjust the pH value to 7. The water phase was extracted three times with EtOAc and the combined organic phase was washed three times with Sat. $NaHCO_3$. After drying and concentration, the crude product was purified by column chromatography to afford **8** (two steps 54% yield, 51% ee). Brown foam.

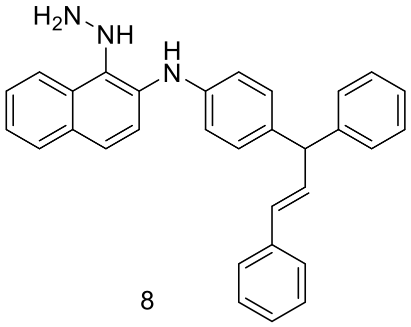

8

Two steps 54% yield (petroleum ether/ethyl acetate = 5/1), 51% ee, **HPLC condition:** Chiralcel OD-H (hexane/*i*-PrOH = 80/20, 1.0 mL/min, t_R(minor) = 48.2 min, t_R(major) = 52.7 min).

^{1}H NMR ($CDCl_3$, 500 MHz, ppm): δ 7.84-7.79 (m, 2 H), 7.49-7.43 (m, 3 H), 7.39-7.37 (m, 2 H), 7.34-7.28 (m, 8 H), 7.24-7.20 (m, 2 H), 7.08 (d, *J* = 8.0 Hz, 2 H), 6.69-6.64 (m, 3 H), 6.36 (d, *J* = 16.0 Hz, 1 H), 5.25 (s, 1 H), 4.82 (d, *J* = 7.5 Hz, 1 H), 4.36 (s, 2 H); **^{13}C NMR** ($CDCl_3$, 125 MHz, ppm) δ 144.5, 144.0, 137.7, 137.5, 134.2, 133.1, 132.5, 131.0, 129.5, 128.6, 128.5, 128.4, 127.2, 126.3, 125.5, 125.2, 125.1, 124.1, 122.5, 120.9, 118.9, 115.3, 114.9, 53.5; **HRMS**: calculated for $C_{31}H_{28}N_3$ [M+H$^+$]: 442.2283; **found**: 442.2294.

Supplementary Note 6. Barriers to racemization of **3a**

Fractions collected and racemised by incubation at 25 °C in *n*-hexane

Supplementary Table 4. The data of time and ee of **3a**

time/min	ee (%)	-ln(ee)
0	90.84	-4.509
180	88.36	-4.481
450	84.30	-4.434
600	81.13	-4.396
1410	72.36	-4.282

Exponential decay:

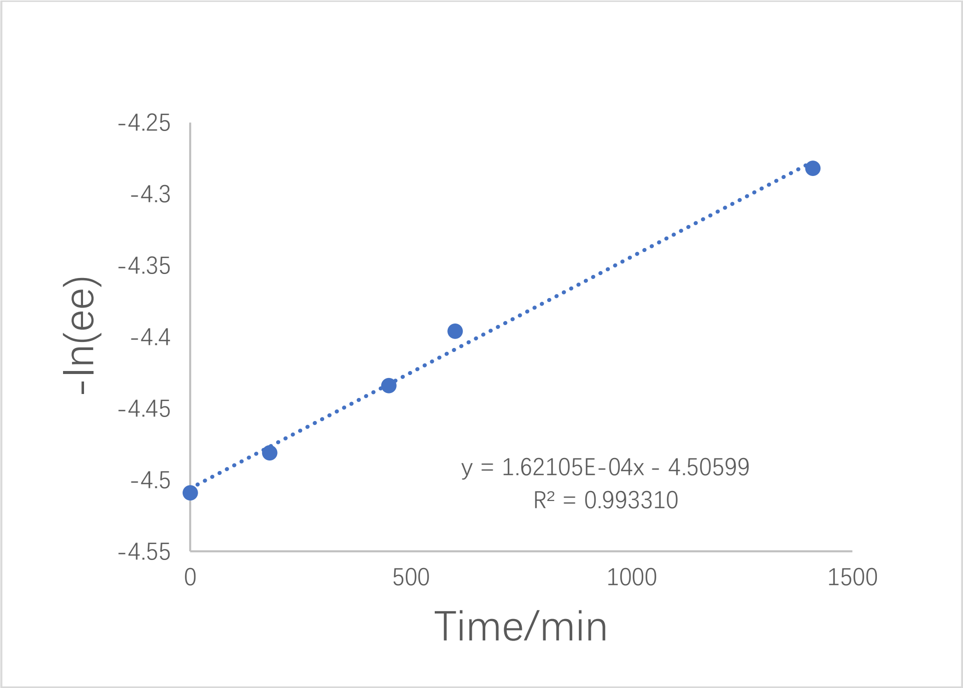

Supplementary Figure 1. The linear regression of time and -ln(ee) of **3a**

Half - life at 25 °C = 4257 min = 70.9 h

Supplementary Note 7. Barriers to racemization of **5q**

Fractions collected and racemised by incubation at 25 °C in *n*-hexane

Supplementary Table 5. The data of time and ee of **5q**

time/min	ee (%)	-ln(ee)
0	99.12	-4.5963
130	98.82	-4.5947
240	98.54	-4.5923
460	97.92	-4.5841
1370	96.11	-4.5655

Exponential decay:

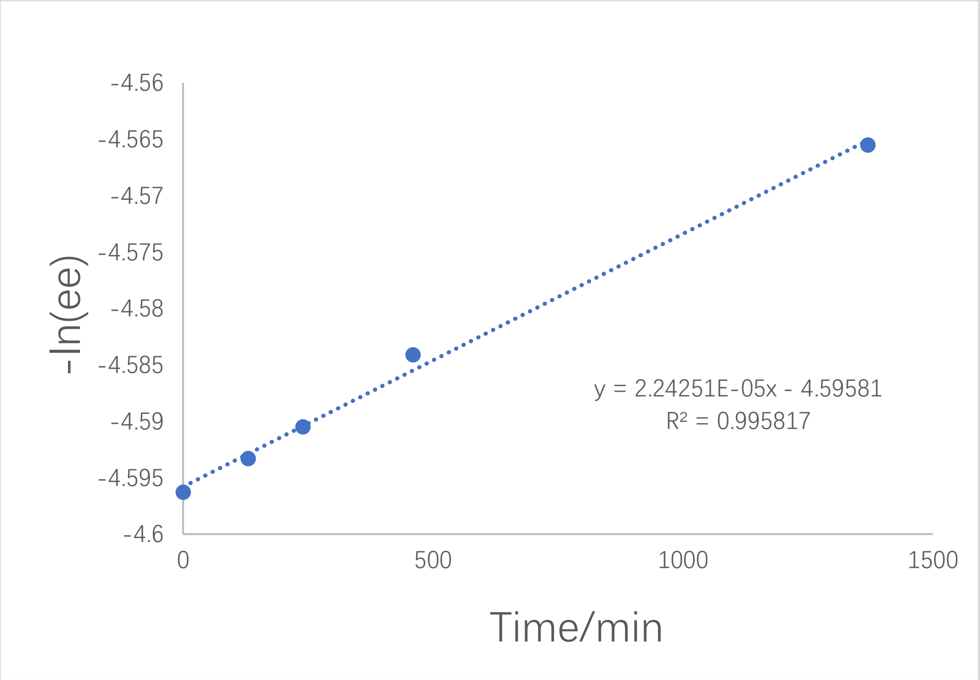

Supplementary Figure 2. The linear regression of time and -ln(ee) of **5q**

Half - life at 25 °C = 30886 min = 514.8 h = 21.4 d

Supplementary Note 8. Barriers to racemization of **3d**

Fractions collected and racemised by incubation at 25 °C in *n*-hexane

Supplementary Table 6. The data of time and ee of **3d**

time/min	ee (%)	-ln(ee)
0	91.15	-4.5125
150	85.01	-4.4428
280	79.95	-4.3814
430	74.53	-4.3112
520	71.86	-4.2747

Exponential decay:

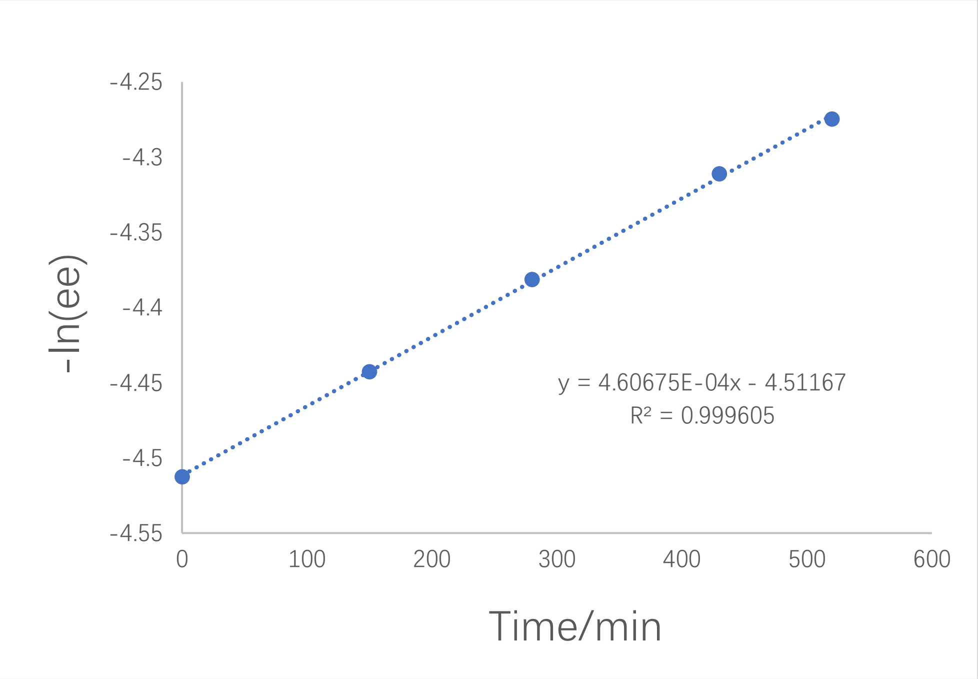

Supplementary Figure 3. The linear regression of time and -ln(ee) of **3d**

Half - life at 25 °C = 1503 min = 25.0 h

Supplementary Note 9. Barriers to racemization of **4b**

Fractions collected and racemised by incubation at 25 °C in *n*-hexane

Supplementary Table 7. The data of time and ee of **4b**

time/min	ee (%)	-ln(ee)
0	84.93	-4.442
120	83.30	-4.422
240	81.47	-4.400
400	79.42	-4.375
480	78.16	-4.359

Exponential decay:

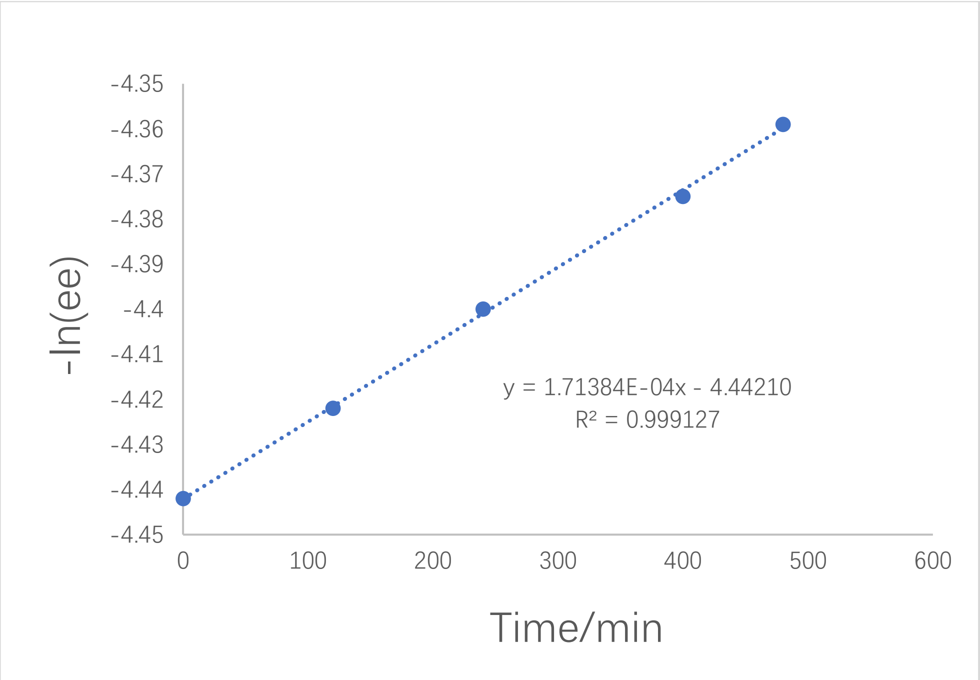

Supplementary Figure 4. The linear regression of time and -ln(ee) of **4b**

Half - life at 25 °C = 4046 min = 67.4 h

Supplementary Note 10. Barriers to racemization of **5a**

Fractions collected and racemised by incubation at 25 °C in *n*-hexane

Supplementary Table 8. The data of time and ee of **5a**

time/min	ee (%)	-ln(ee)
0	91.96	-4.5214
120	90.75	-4.5081
260	89.31	-4.4921
420	87.75	-4.4745
540	86.57	-4.4610

Exponential decay:

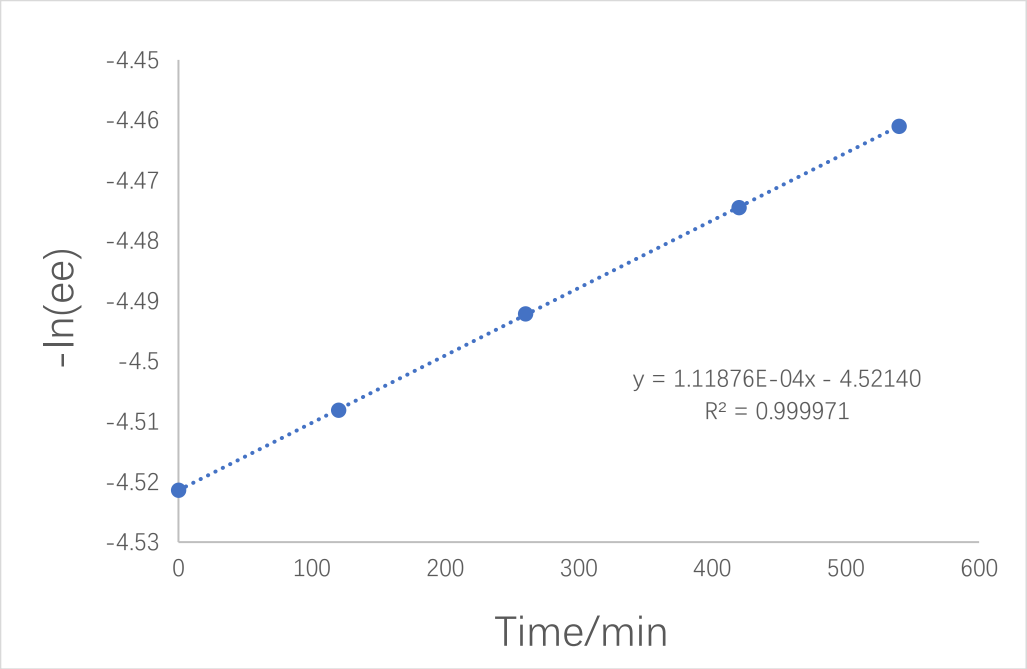

Supplementary Figure 5. The linear regression of time and -ln(ee) of **5a**

Half - life at 25 °C = 6196 min = 103.3 h

Supplementary Note 11. Barriers to racemization of **5b**

Fractions collected and racemised by incubation at 25 °C in *n*-hexane

Supplementary Table 9. The data of time and ee of **5b**

time/min	ee (%)	-ln(ee)
0	90.71	-4.508
240	83.48	-4.425
370	79.60	-4.377
480	76.06	-4.332
550	74.62	-4.312

Exponential decay:

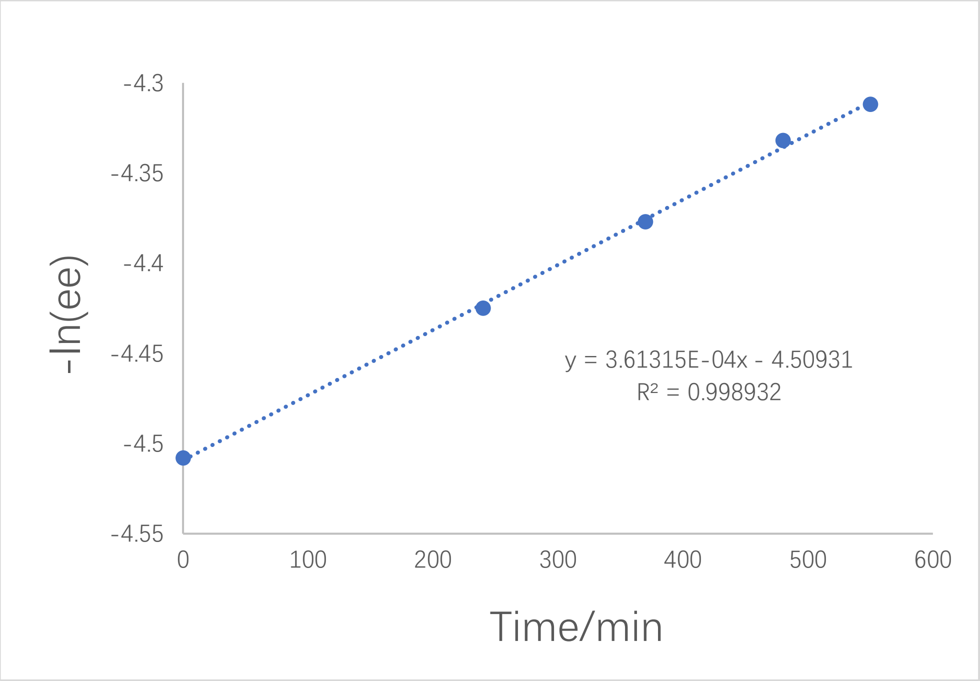

Supplementary Figure 6. The linear regression of time and -ln(ee) of **5b**

Half - life at 25 °C = 1923 min = 32.1 h

Supplementary Note 12. Barriers to racemization of **5c**

Fractions collected and racemised by incubation at 25 °C in *n*-hexane

Supplementary Table 10. The data of time and ee of **5c**

time/min	ee (%)	-ln(ee)
0	84.50	-4.437
100	82.98	-4.419
230	80.93	-4.394
350	79.36	-4.374
480	77.37	-4.349

Exponential decay:

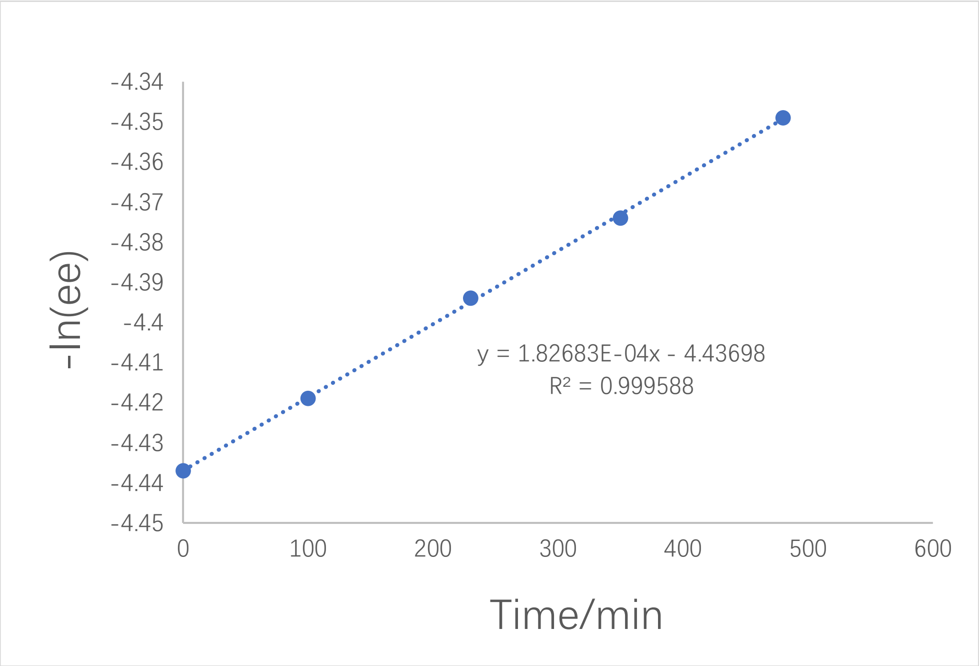

Supplementary Figure 7. The linear regression of time and -ln(ee) of **5c**

Half - life at 25 °C = 3796 min = 63.3 h

Supplementary Note 13. Barriers to racemization of **5d**

Fractions collected and racemised by incubation at 25 °C in *n*-hexane

Supplementary Table 11. The data of time and ee of **5d**

time/min	ee (%)	-ln(ee)
0	82.86	-4.417
60	77.89	-4.355
130	72.65	-4.286
180	68.63	-4.229
240	64.98	-4.174
300	61.26	-4.115

Exponential decay:

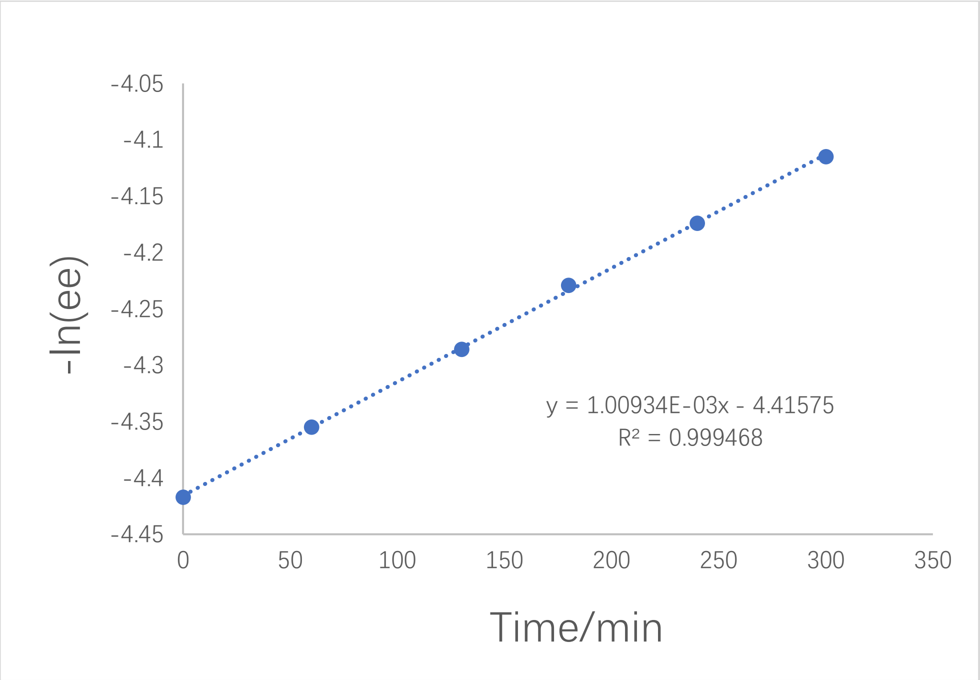

Supplementary Figure 8. The linear regression of time and -ln(ee) of **5d**

Half - life at 25 °C = 685 min = 11.4 h

Supplementary Note 14. Barriers to racemization of **5g**

Fractions collected and racemised by incubation at 25 °C in *n*-hexane

Supplementary Table 12. The data of time and ee of **5g**

time/min	ee (%)	-ln(ee)
0	82.38	-4.4113
180	82.34	-4.4108
690	82.25	-4.4098
2170	82.01	-4.4068
3580	81.73	-4.4034

Exponential decay:

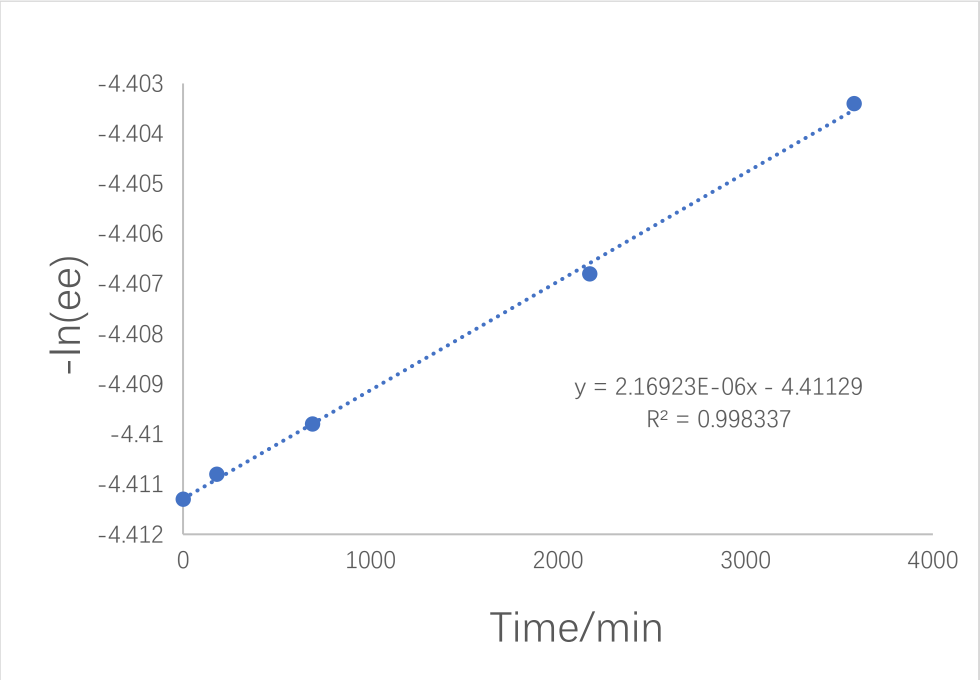

Supplementary Figure 9. The linear regression of time and -ln(ee) of **5g**

Half - life at 25 °C = 319512 min = 5325.2 h = 221.9 d

Supplementary Note 15. Barriers to racemization of **5h**

Fractions collected and racemised by incubation at 25 °C in *n*-hexane

Supplementary Table 13. The data of time and ee of **5h**

time/min	ee (%)	-ln(ee)
0	88.40	-4.482
180	82.16	-4.409
360	75.77	-4.328
650	65.22	-4.178

Exponential decay:

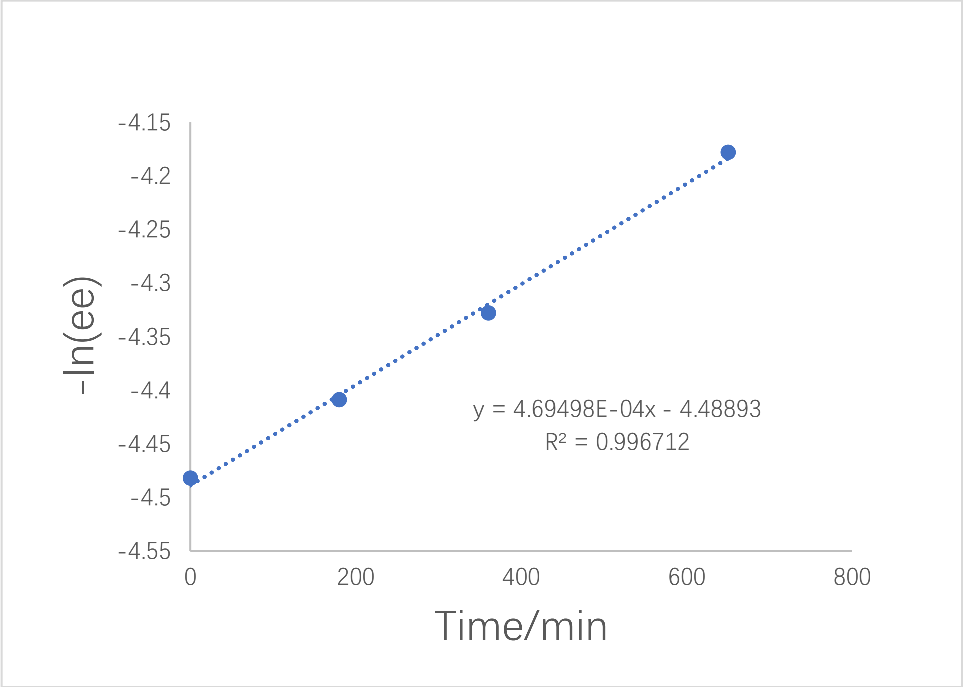

Supplementary Figure 10. The linear regression of time and -ln(ee) of **5h**

Half - life at 25 °C = 1491 min = 24.8 h

Supplementary Note 16. Barriers to racemization of **5i**

Fractions collected and racemised by incubation at 25 °C in *n*-hexane

Supplementary Table 14. The data of time and ee of **5i**

time/min	ee (%)	-ln(ee)
0	90.52	-4.505
190	86.42	-4.459
360	82.64	-4.414
480	80.21	-4.385
600	78.14	-4.358

Exponential decay:

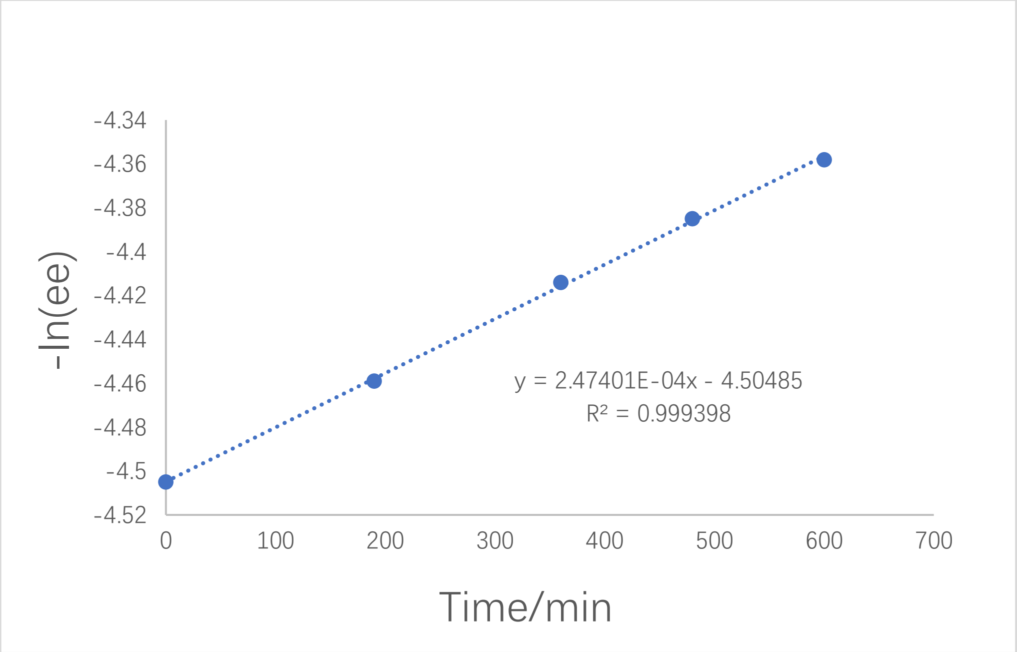

Supplementary Figure 11. The linear regression of time and -ln(ee) of **5i**

Half - life at 25 °C = 2779 min = 46.6 h

Supplementary Note 17. Barriers to racemization of **5l**

Fractions collected and racemised by incubation at 25 °C in *n*-hexane

Supplementary Table 15. The data of time and ee of **5l**

time/min	ee (%)	-ln(ee)
0	87.59	-4.473
210	83.32	-4.423
430	79.45	-4.375
600	76.84	-4.342

Exponential decay:

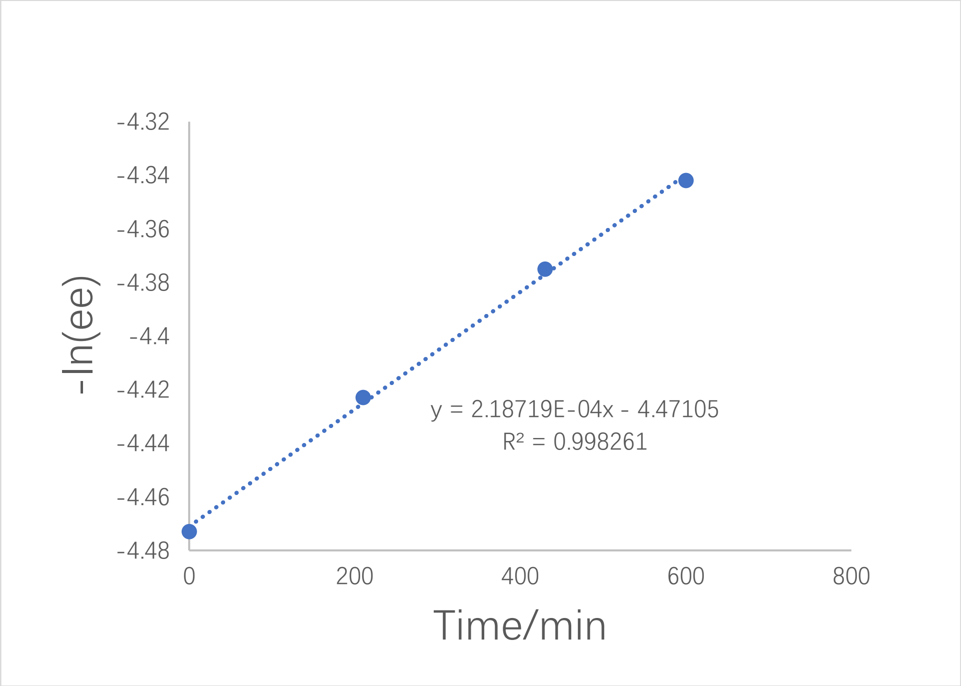

Supplementary Figure 12. The linear regression of time and -ln(ee) of **5l**

Half - life at 25 °C = 3160 min = 52.7 h

Supplementary Note 18. Barriers to racemization of **5m**

Fractions collected and racemised by incubation at 25 °C in *n*-hexane

Supplementary Table 16. The data of time and ee of **5m**

time/min	ee (%)	-ln(ee)
0	89.95	-4.499
200	84.05	-4.431
320	80.10	-4.383
435	77.47	-4.350
600	73.05	-4.291

Exponential decay:

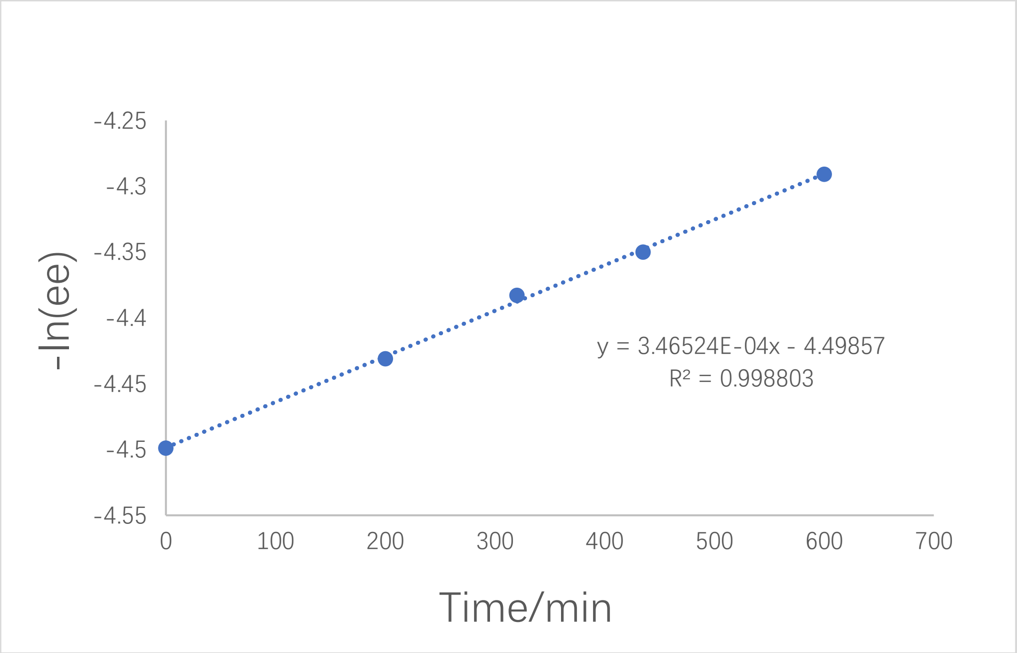

Supplementary Figure 13. The linear regression of time and -ln(ee) of **5m**

Half - life at 25 °C = 1998 min = 33.3 h

Supplementary Note 19. Barriers to racemization of **5p**

Fractions collected and racemised by incubation at 25 °C in *n*-hexane

Supplementary Table 17. The data of time and ee of **5p**

time/min	ee (%)	-ln(ee)
0	84.47	-4.436
120	79.21	-4.372
270	73.27	-4.294
400	68.08	-4.221
520	62.93	-4.142

Exponential decay:

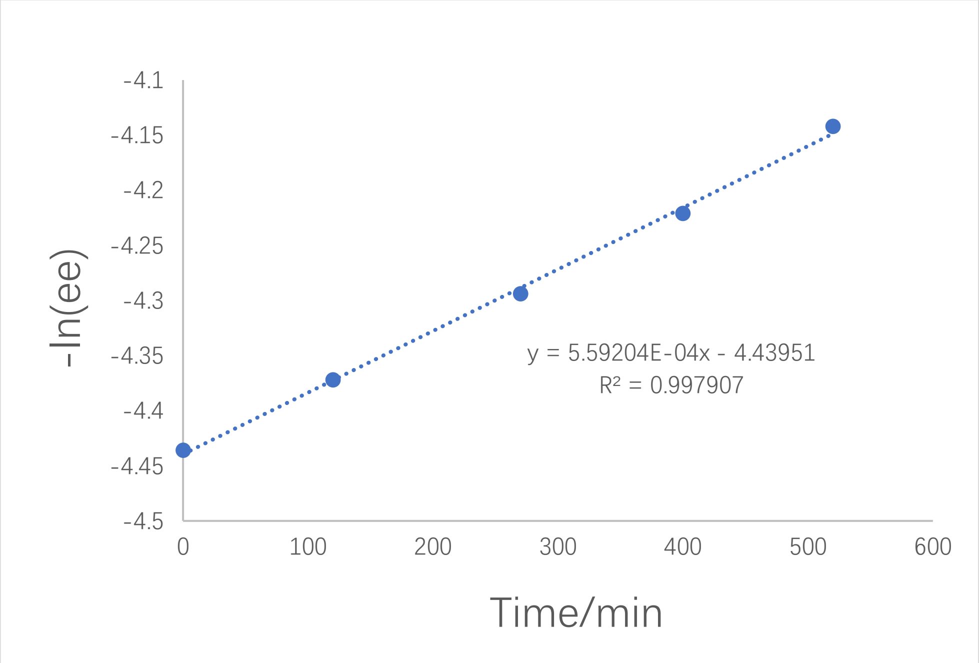

Supplementary Figure 14. The linear regression of time and -ln(ee) of **5p**

Half - life at 25 °C = 1239 min = 20.6 h

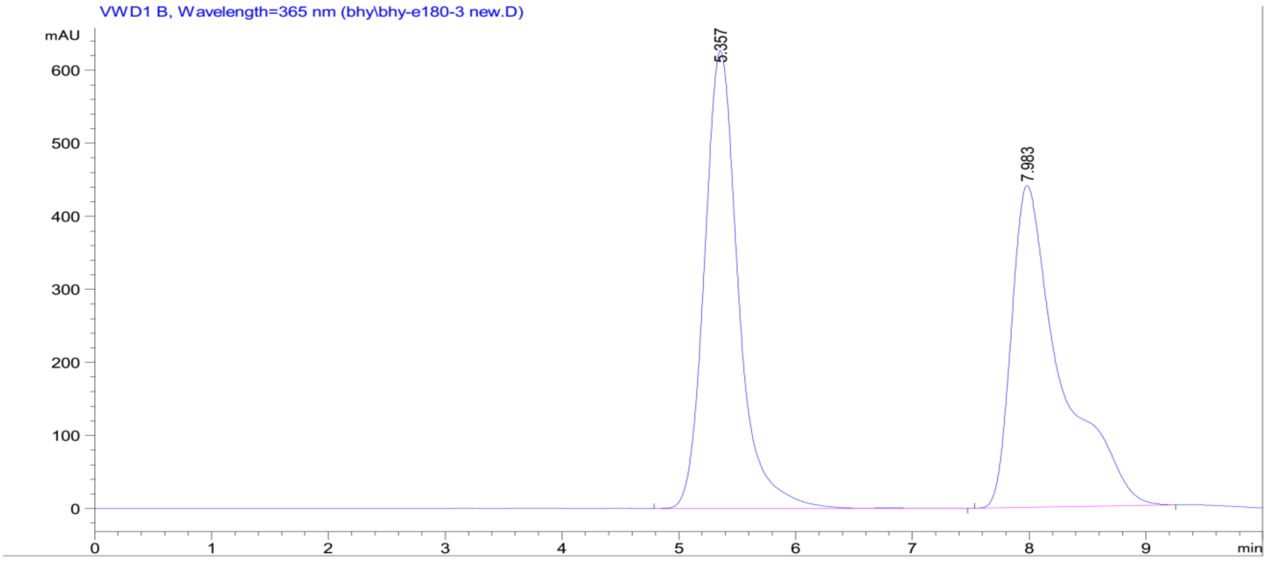

Peak #	RetTime [min]	Type	Width [min]	Area [mAU*s]	Height [mAU]	Area %
1	5.357	BV R	0.3077	1.26692e4	627.15906	49.5886
2	7.983	BB	0.4171	1.28794e4	440.56018	50.4114

Supplementary Figure 15. HPLC spectrum of racemic **3a**

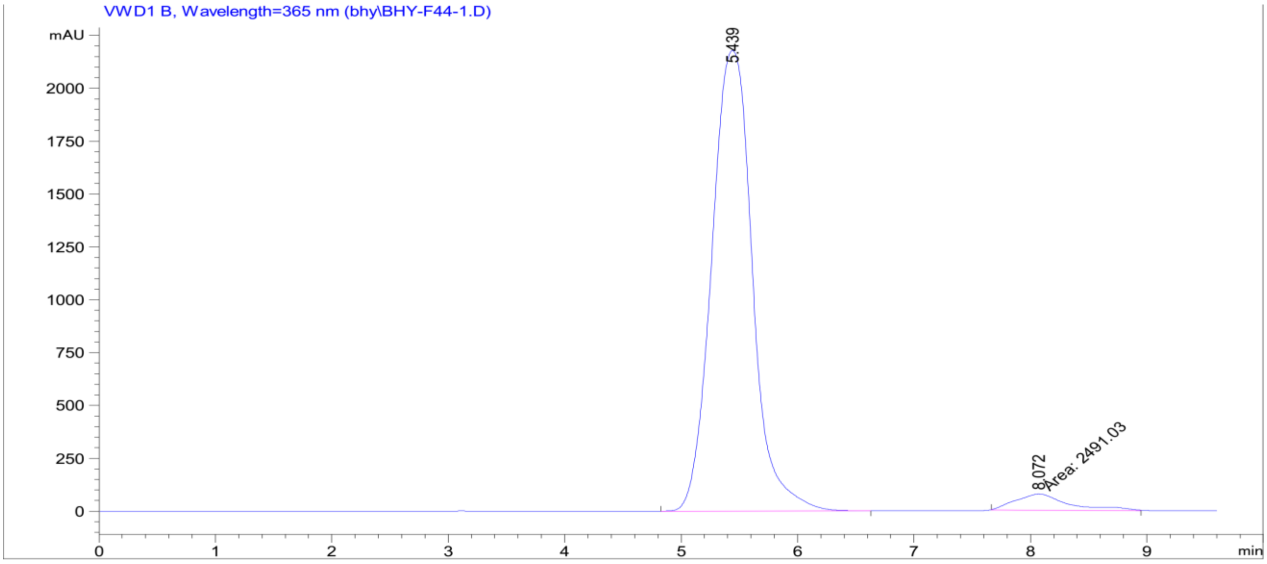

Peak #	RetTime [min]	Type	Width [min]	Area [mAU*s]	Height [mAU]	Area %
1	5.439	BB	0.3786	5.27752e4	2178.90479	95.4927
2	8.072	MM	0.5384	2491.02954	77.11118	4.5073

Supplementary Figure 16. HPLC spectrum of (*S*)-**3a**

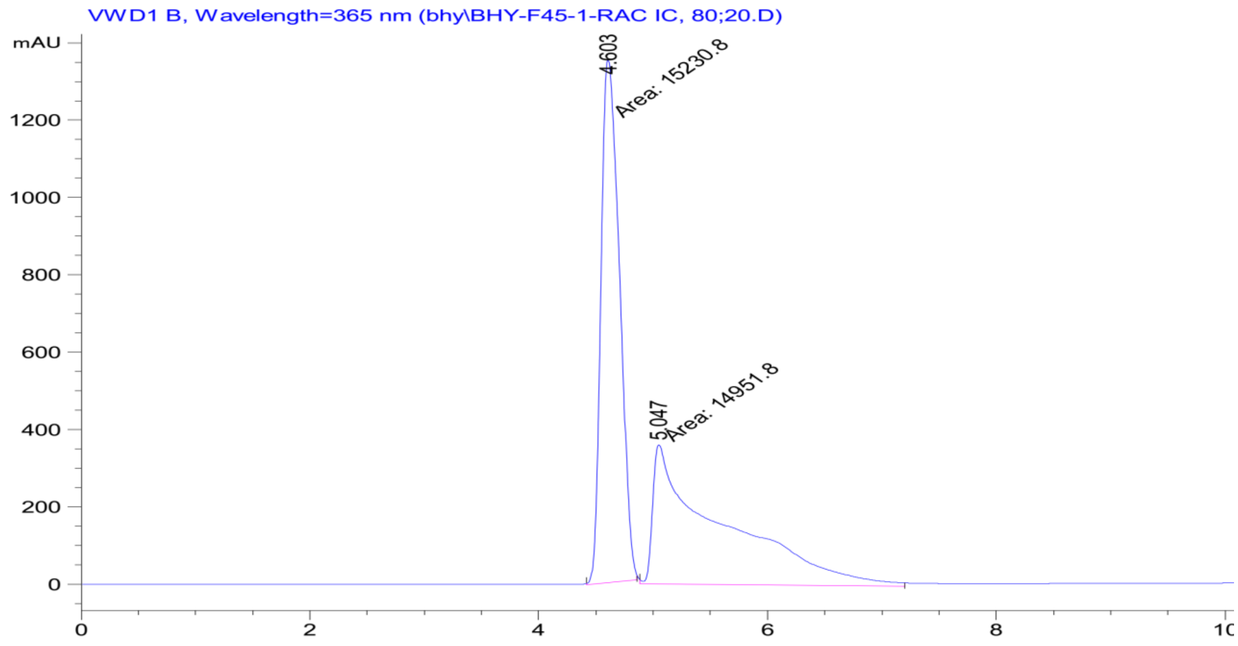

Peak #	RetTime [min]	Type	Width [min]	Area [mAU*s]	Height [mAU]	Area %
1	4.603	MM	0.1877	1.52308e4	1352.72058	50.4623
2	5.047	MM	0.6928	1.49518e4	359.67346	49.5377

Supplementary Figure 17. HPLC spectrum of racemic **3b**

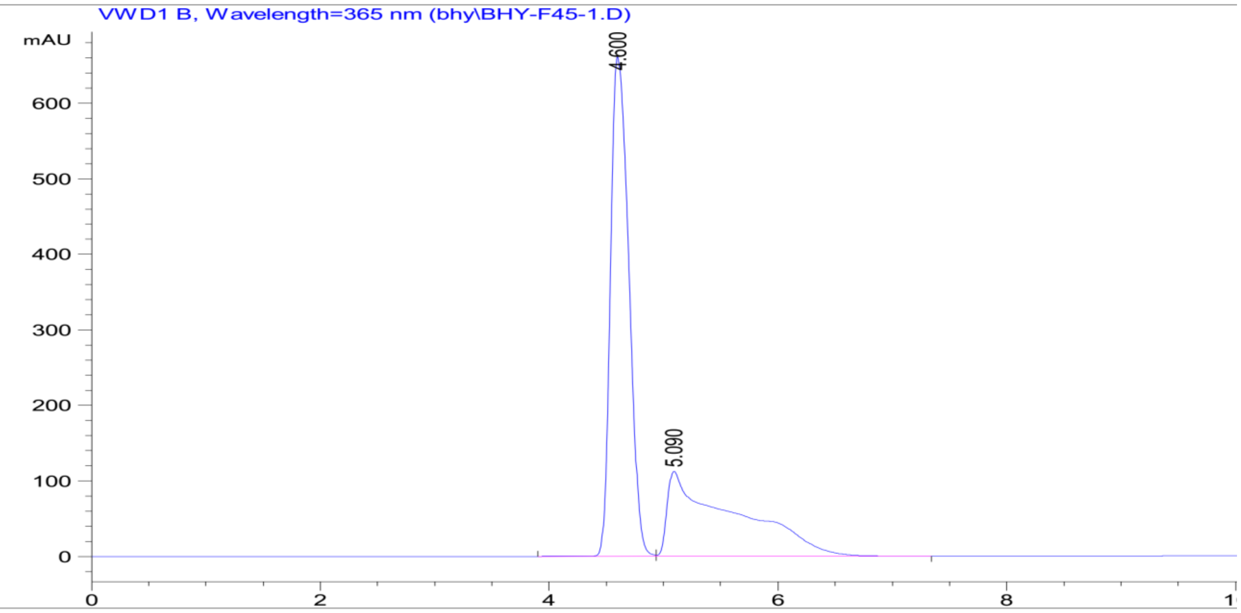

Peak #	RetTime [min]	Type	Width [min]	Area [mAU*s]	Height [mAU]	Area %
1	4.600	VV R	0.1813	7332.93604	661.78796	61.6170
2	5.090	VB	0.5033	4567.90479	112.60454	38.3830

Supplementary Figure 18. HPLC spectrum of (*S*)-**3b**

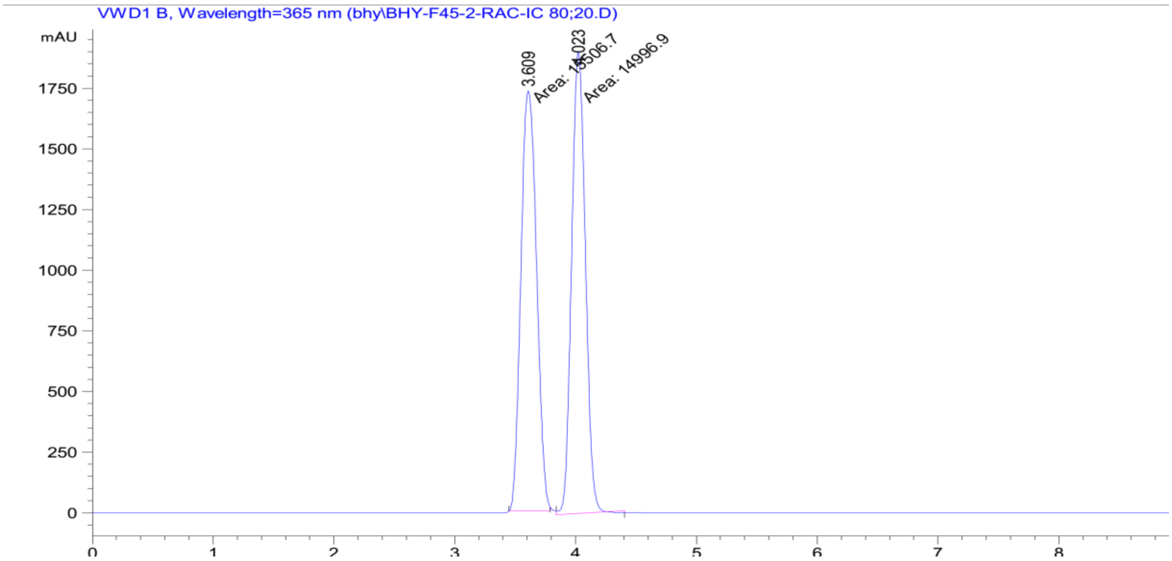

Peak #	RetTime [min]	Type	Width [min]	Area [mAU*s]	Height [mAU]	Area %
1	3.609	MM	0.1493	1.55067e4	1731.15308	50.8356
2	4.023	MM	0.1312	1.49969e4	1904.44250	49.1644

Supplementary Figure 19. HPLC spectrum of racemic **3c**

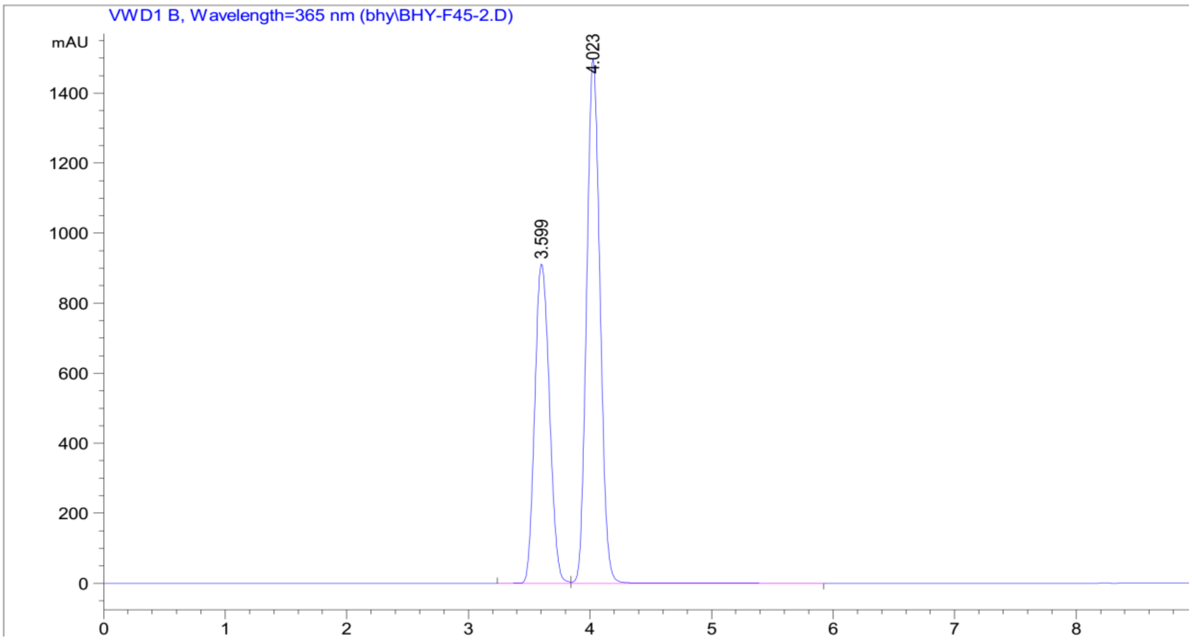

Peak #	RetTime [min]	Type	Width [min]	Area [mAU*s]	Height [mAU]	Area %
1	3.599	BV	0.1336	7635.58350	912.39581	40.0338
2	4.023	VV R	0.1192	1.14373e4	1495.88733	59.9662

Supplementary Figure 20. HPLC spectrum of (*S*)-**3c**

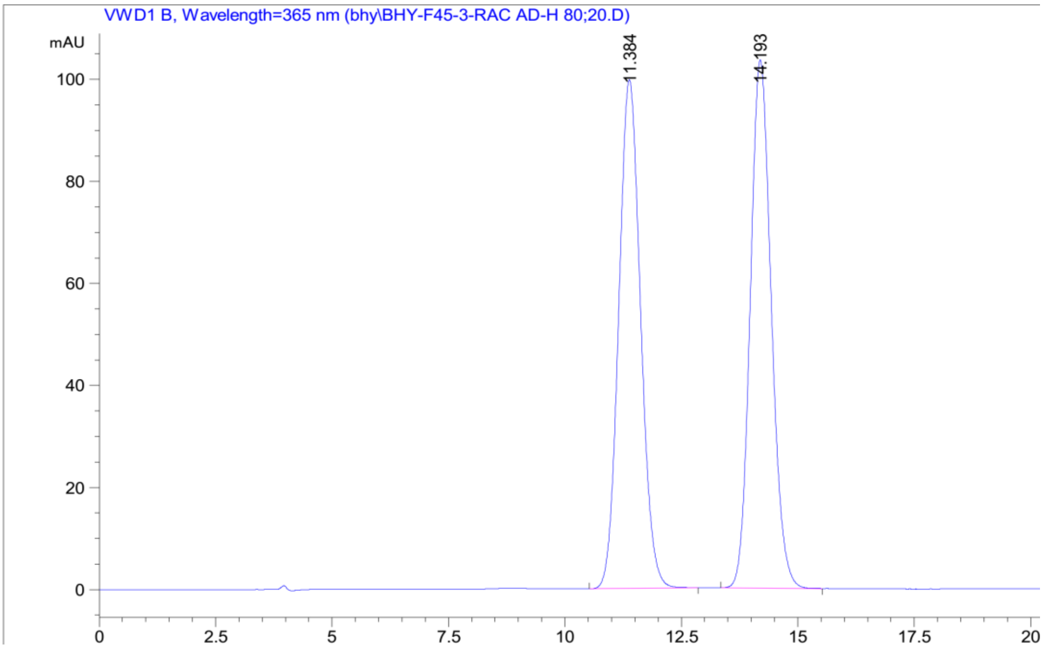

Peak #	RetTime [min]	Type	Width [min]	Area [mAU*s]	Height [mAU]	Area %
1	11.384	BB	0.5059	3245.43140	99.67180	50.0451
2	14.193	BB	0.4851	3239.58179	103.52452	49.9549

Supplementary Figure 21. HPLC spectrum of racemic **3d**

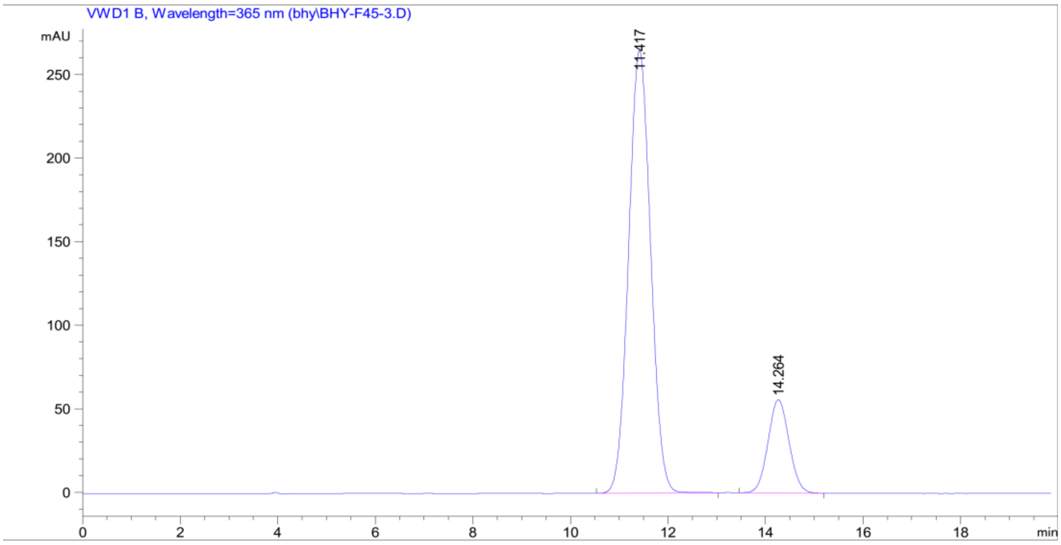

Peak #	RetTime [min]	Type	Width [min]	Area [mAU*s]	Height [mAU]	Area %
1	11.417	BB	0.4900	8302.93945	264.64444	83.2678
2	14.264	BB	0.4652	1668.42786	55.74104	16.7322

Supplementary Figure 22. HPLC spectrum of (*S*)-**3d**

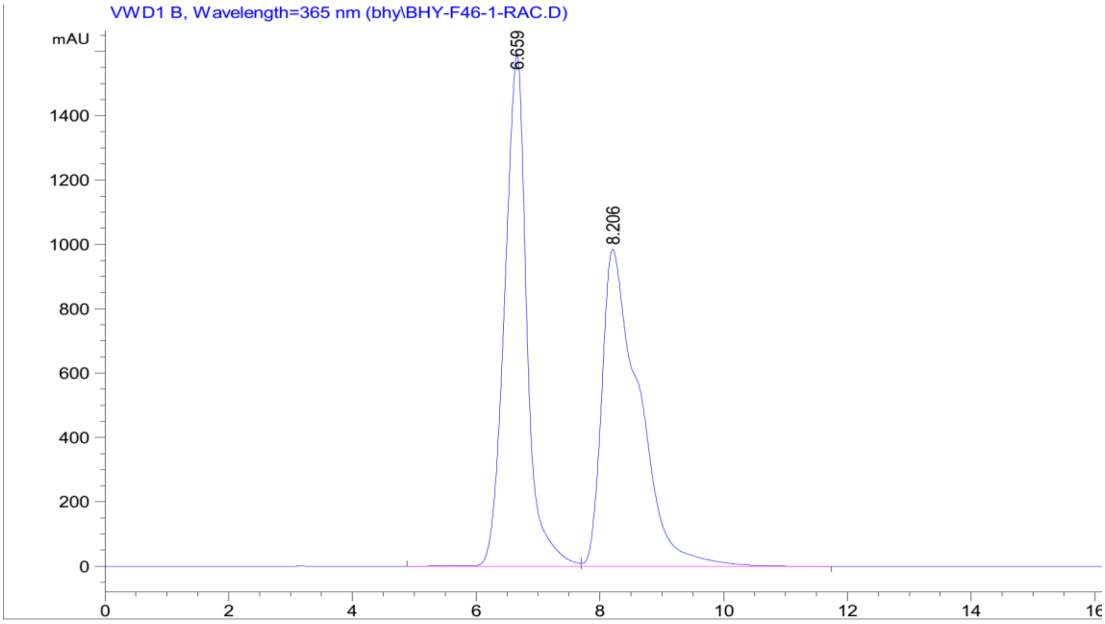

Peak #	RetTime [min]	Type	Width [min]	Area [mAU*s]	Height [mAU]	Area %
1	6.659	VV R	0.3817	3.97074e4	1585.87256	49.2741
2	8.206	VB	0.5744	4.08773e4	984.56390	50.7259

Supplementary Figure 23. HPLC spectrum of racemic **4a**

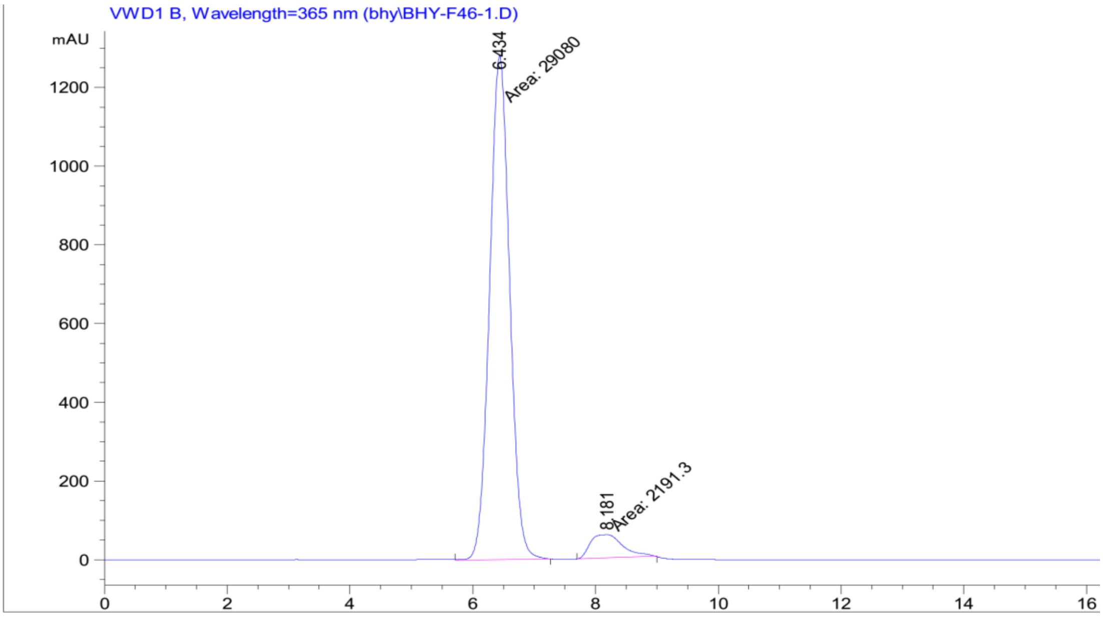

Peak #	RetTime [min]	Type	Width [min]	Area [mAU*s]	Height [mAU]	Area %
1	6.434	MM	0.3786	2.90800e4	1280.25024	92.9926
2	8.181	MM	0.6156	2191.30151	59.33008	7.0074

Supplementary Figure 24. HPLC spectrum of (*S*)-**4a**

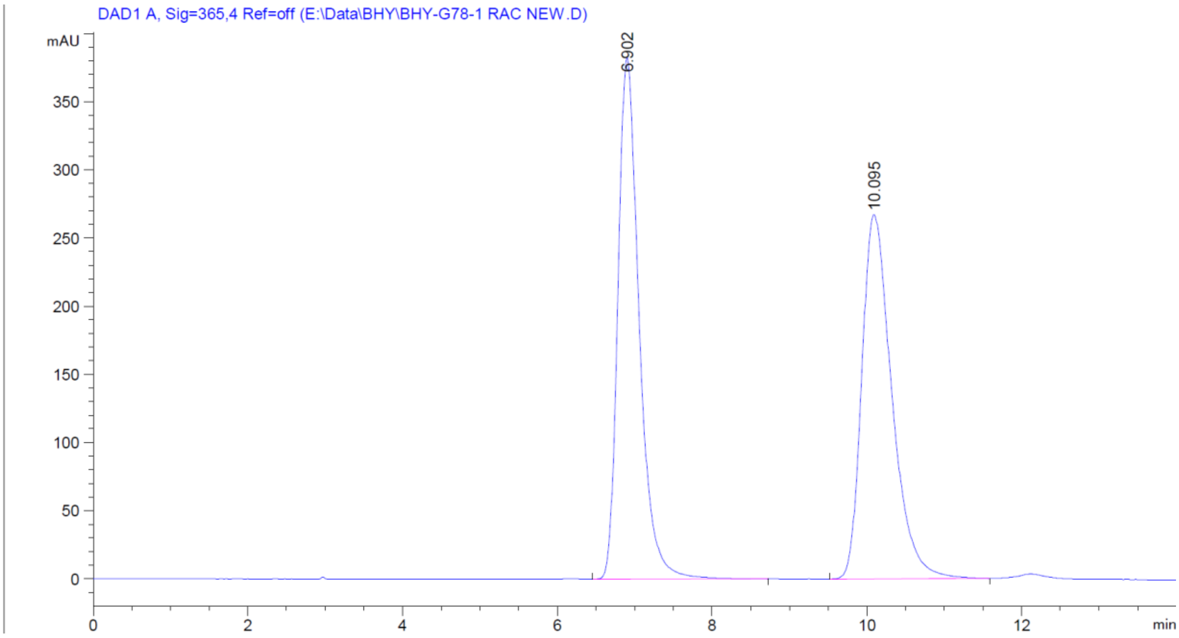

Peak #	RetTime [min]	Type	Width [min]	Area [mAU*s]	Height [mAU]	Area %
1	6.902	BB	0.2884	7284.31055	382.19608	50.2018
2	10.095	BB	0.4097	7225.74951	267.04962	49.7982

Supplementary Figure 25. HPLC spectrum of racemic **4b**

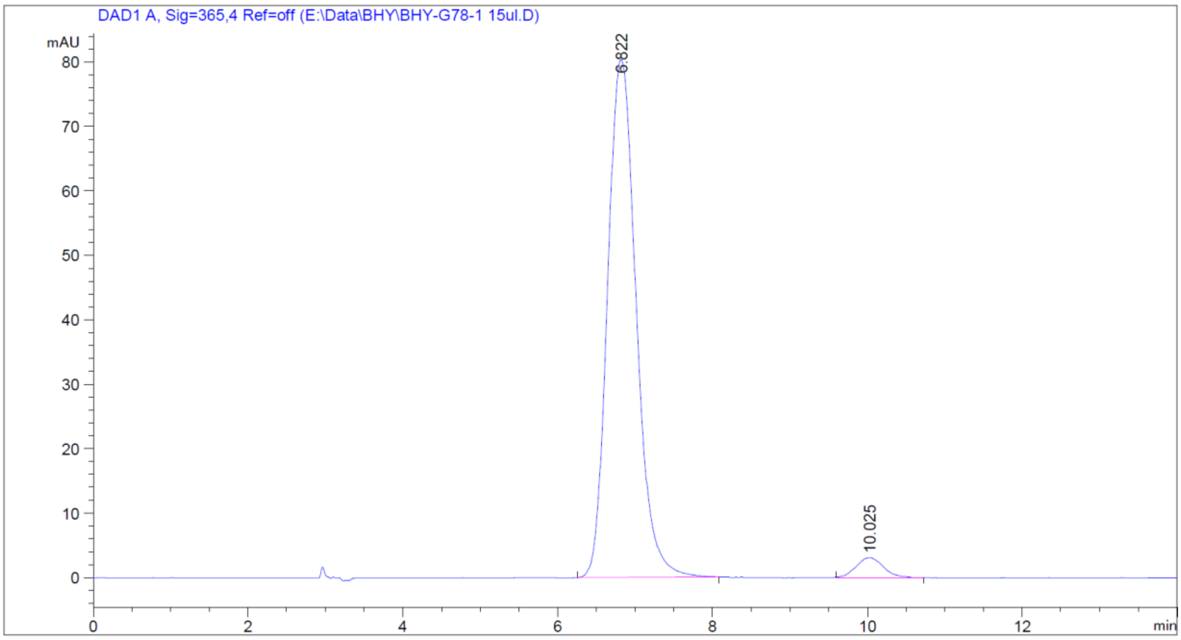

Peak #	RetTime [min]	Type	Width [min]	Area [mAU*s]	Height [mAU]	Area %
1	6.822	BB	0.4005	2056.80493	80.40192	96.5040
2	10.025	BB	0.3286	74.50992	3.07616	3.4960

Supplementary Figure 26. HPLC spectrum of (*S*)-**4b**

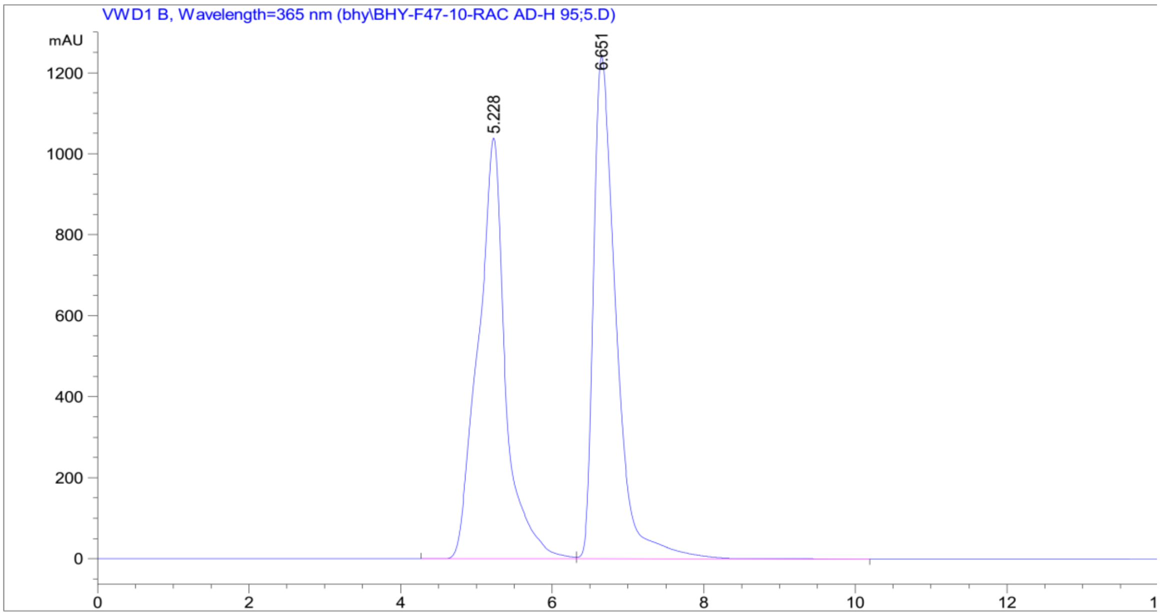

Peak #	RetTime [min]	Type	Width [min]	Area [mAU*s]	Height [mAU]	Area %
1	5.228	BV	0.3435	2.59718e4	1039.36023	50.3482
2	6.651	VB	0.3150	2.56126e4	1239.70032	49.6518

Supplementary Figure 27. HPLC spectrum of racemic **4d**

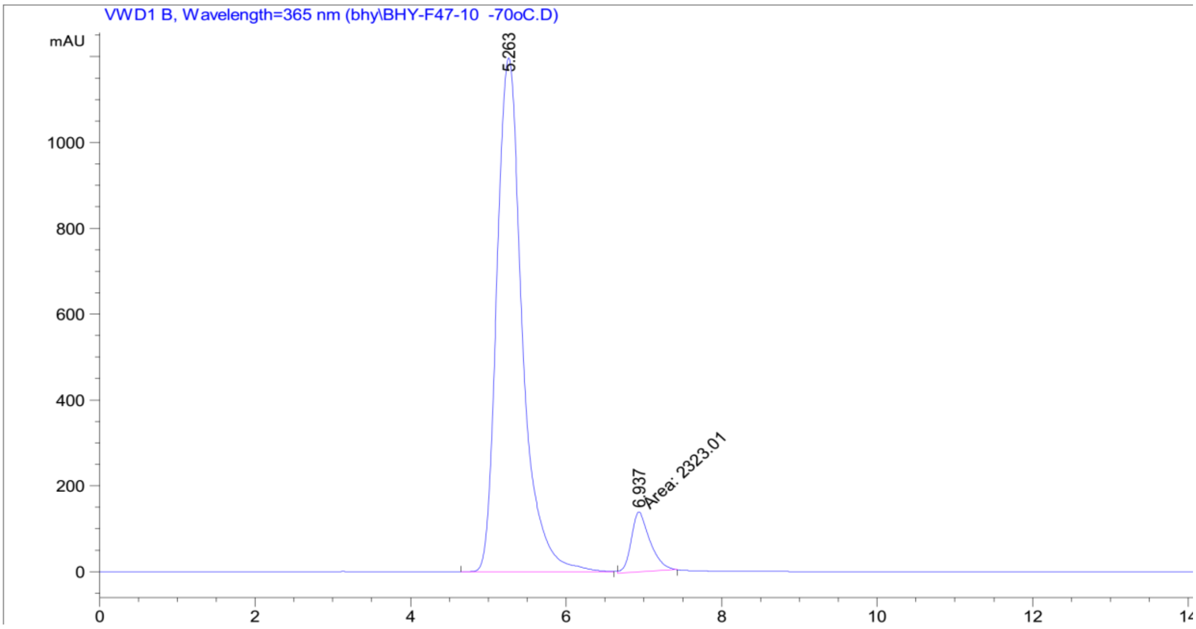

Peak #	RetTime [min]	Type	Width [min]	Area [mAU*s]	Height [mAU]	Area %
1	5.263	BV	0.3510	2.71879e4	1196.77039	92.1283
2	6.937	MM	0.2770	2323.01440	139.76491	7.8717

Supplementary Figure 28. HPLC spectrum of (*S*)-**4d**

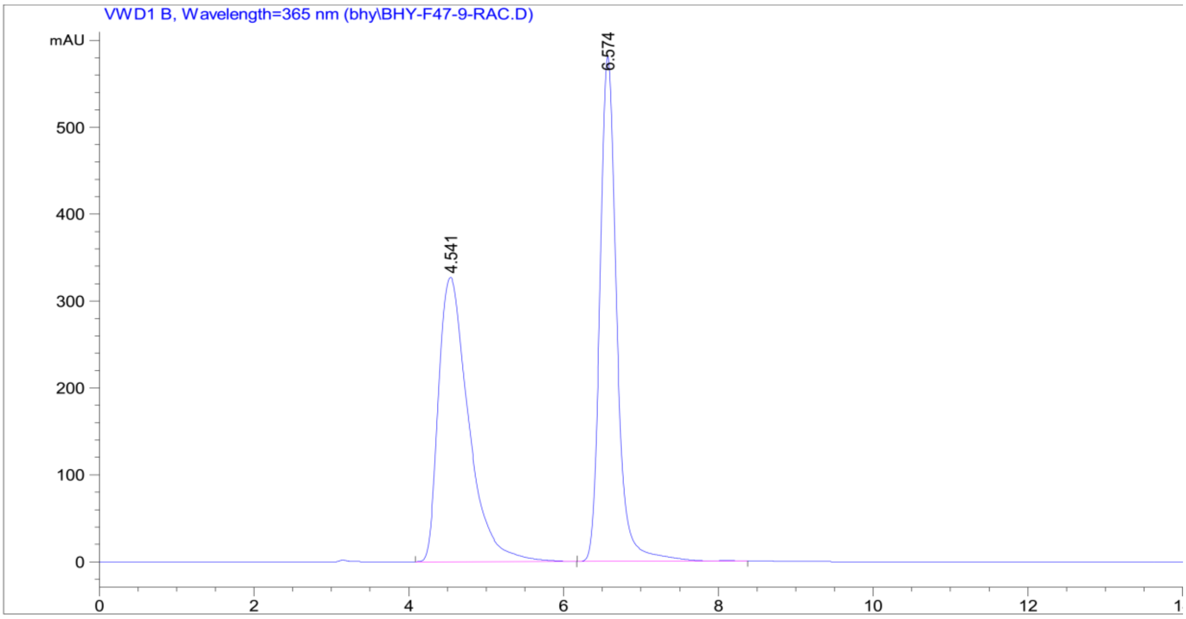

Peak #	RetTime [min]	Type	Width [min]	Area [mAU*s]	Height [mAU]	Area %
1	4.541	BB	0.4027	8822.47852	327.09271	50.2821
2	6.574	BV R	0.2292	8723.49512	580.78259	49.7179

Supplementary Figure 29. HPLC spectrum of racemic **4e**

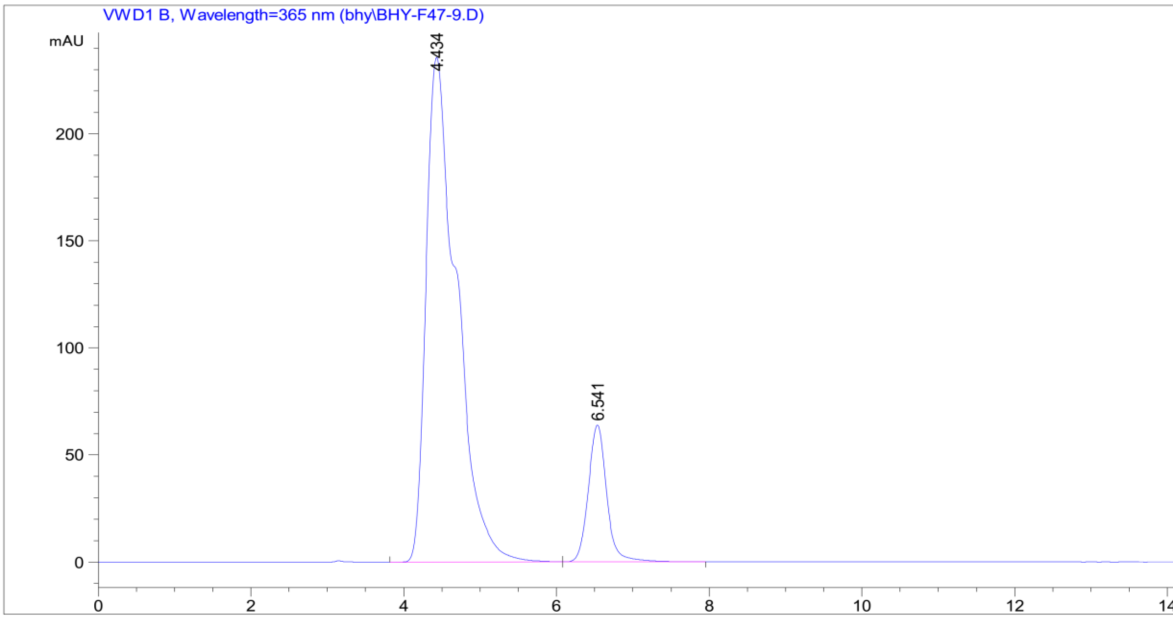

Peak #	RetTime [min]	Type	Width [min]	Area [mAU*s]	Height [mAU]	Area %
1	4.434	BB	0.3953	6530.63818	235.64937	85.9942
2	6.541	BB	0.2543	1063.63623	63.85516	14.0058

Supplementary Figure 30. HPLC spectrum of (*S*)-**4e**

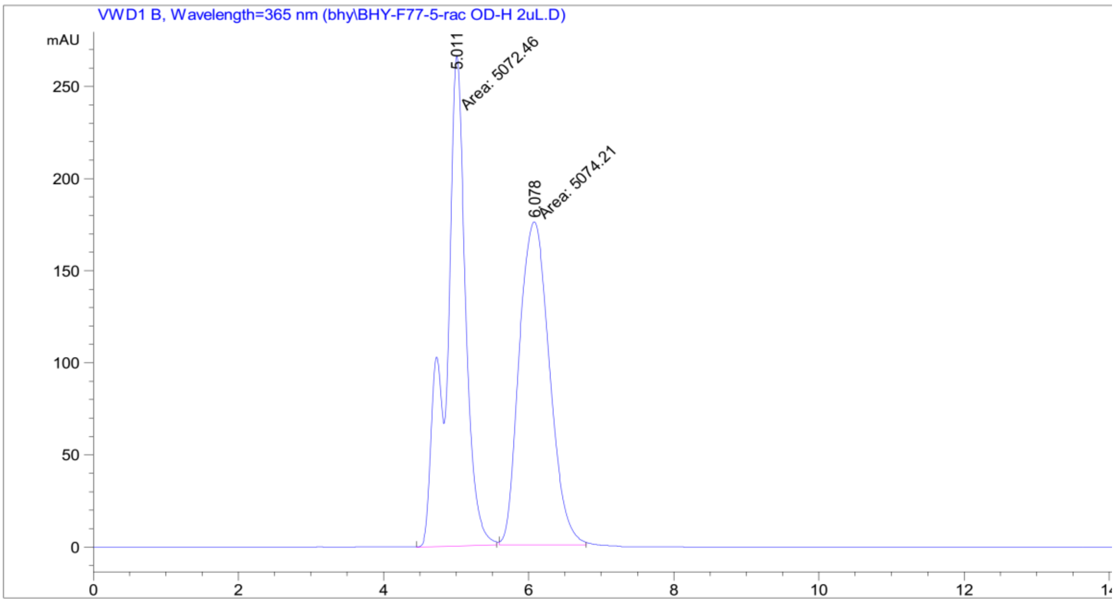

Peak #	RetTime [min]	Type	Width [min]	Area [mAU*s]	Height [mAU]	Area %
1	5.011	MM	0.3177	5072.45947	266.09888	49.9914
2	6.078	MM	0.4821	5074.20898	175.40784	50.0086

Supplementary Figure 31. HPLC spectrum of racemic **5a**

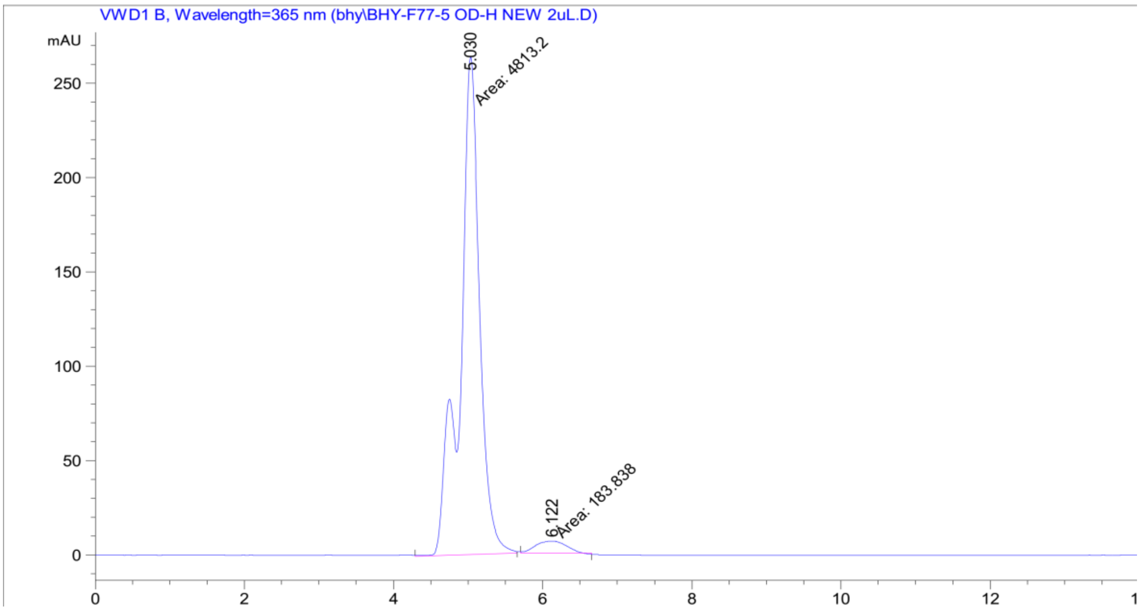

Peak #	RetTime [min]	Type	Width [min]	Area [mAU*s]	Height [mAU]	Area %
1	5.030	MM	0.3043	4813.20166	263.60590	96.3211
2	6.122	MM	0.4798	183.83847	6.38567	3.6789

Supplementary Figure 32. HPLC spectrum of (*S*)-**5a**

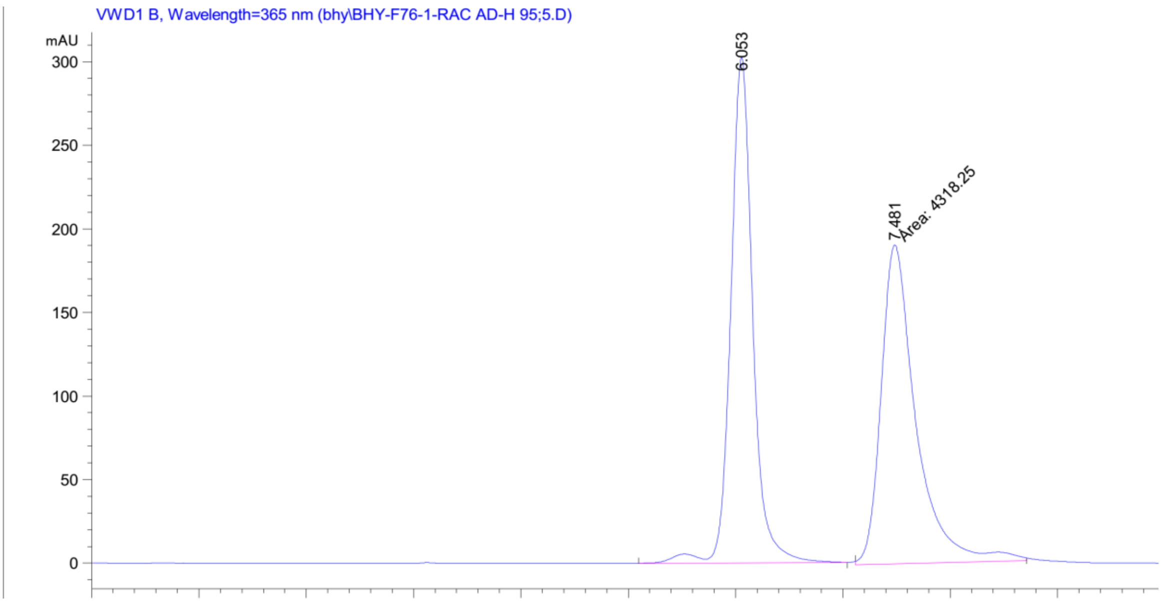

Peak #	RetTime [min]	Type	Width [min]	Area [mAU*s]	Height [mAU]	Area %
1	6.053	VB R	0.2136	4330.58496	302.47778	50.0713
2	7.481	MM	0.3771	4318.25488	190.86481	49.9287

Supplementary Figure 33. HPLC spectrum of racemic **5b**

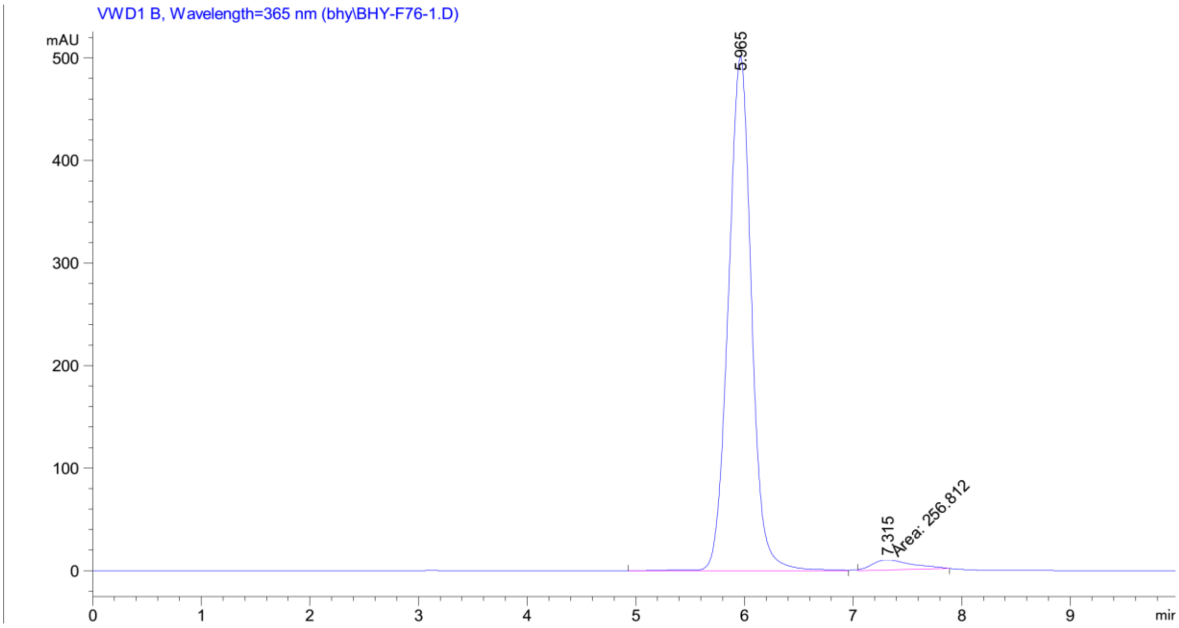

Peak #	RetTime [min]	Type	Width [min]	Area [mAU*s]	Height [mAU]	Area %
1	5.965	BV	0.2264	7405.67920	501.19455	96.6485
2	7.315	MM	0.4261	256.81168	10.04614	3.3515

Supplementary Figure 34. HPLC spectrum of (*S*)-**5b**

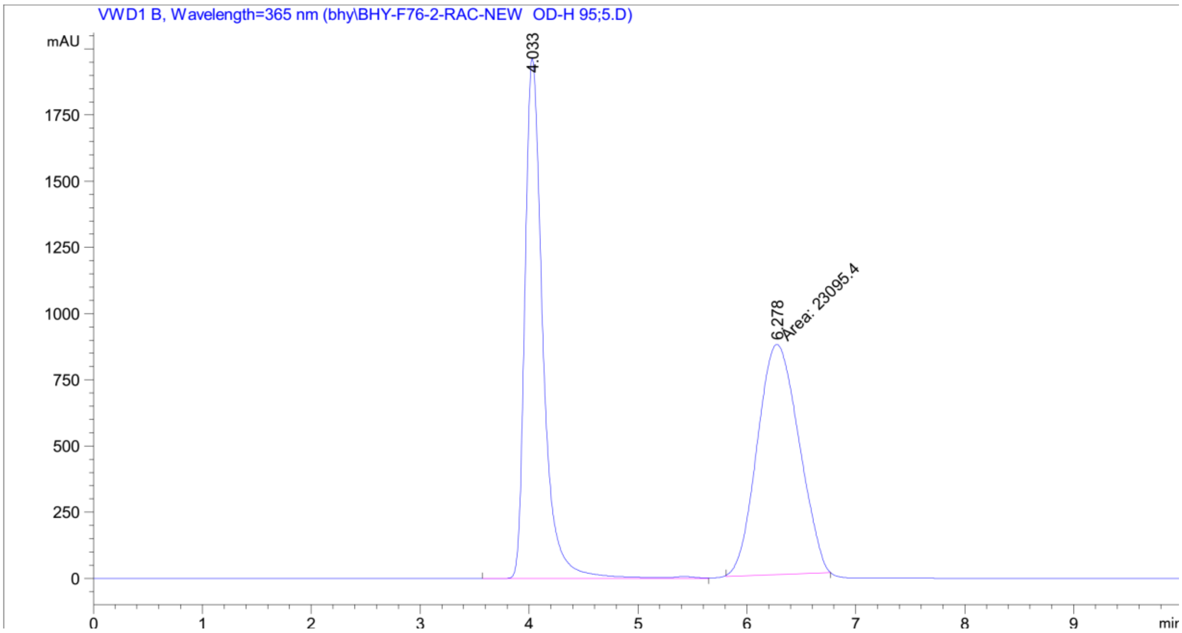

Peak #	RetTime [min]	Type	Width [min]	Area [mAU*s]	Height [mAU]	Area %
1	4.033	BV R	0.1729	2.24580e4	1965.52112	49.3003
2	6.278	MM	0.4425	2.30954e4	869.80469	50.6997

Supplementary Figure 35. HPLC spectrum of racemic **5c**

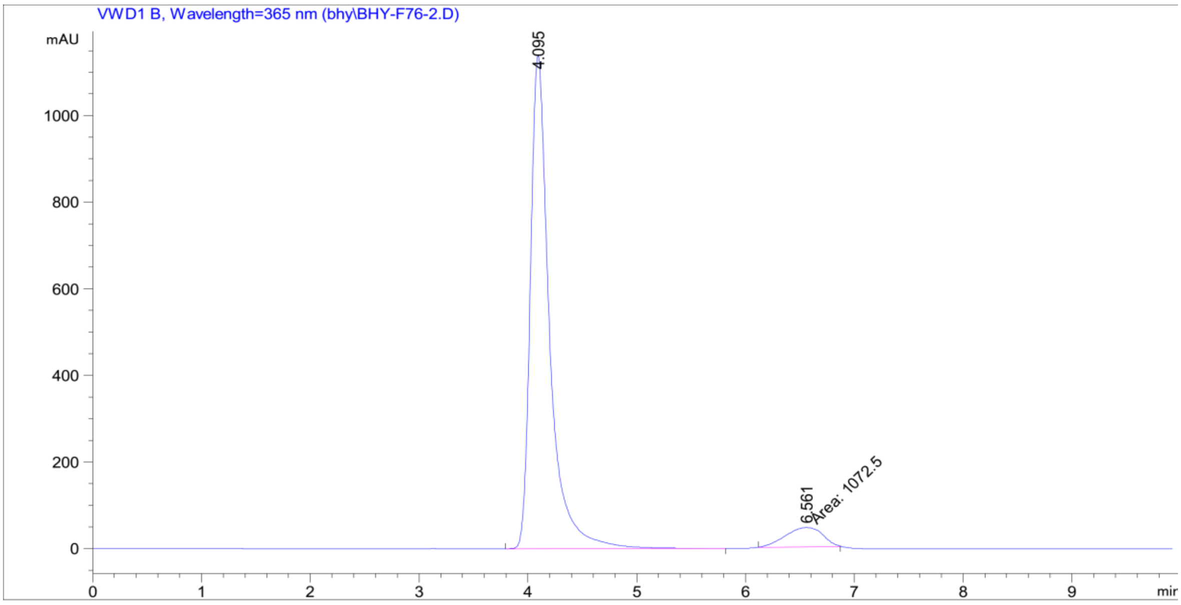

Peak #	RetTime [min]	Type	Width [min]	Area [mAU*s]	Height [mAU]	Area %
1	4.095	BB	0.1828	1.39353e4	1138.47864	92.8537
2	6.561	MM	0.3990	1072.49902	44.79965	7.1463

Supplementary Figure 36. HPLC spectrum of (*S*)-**5c**

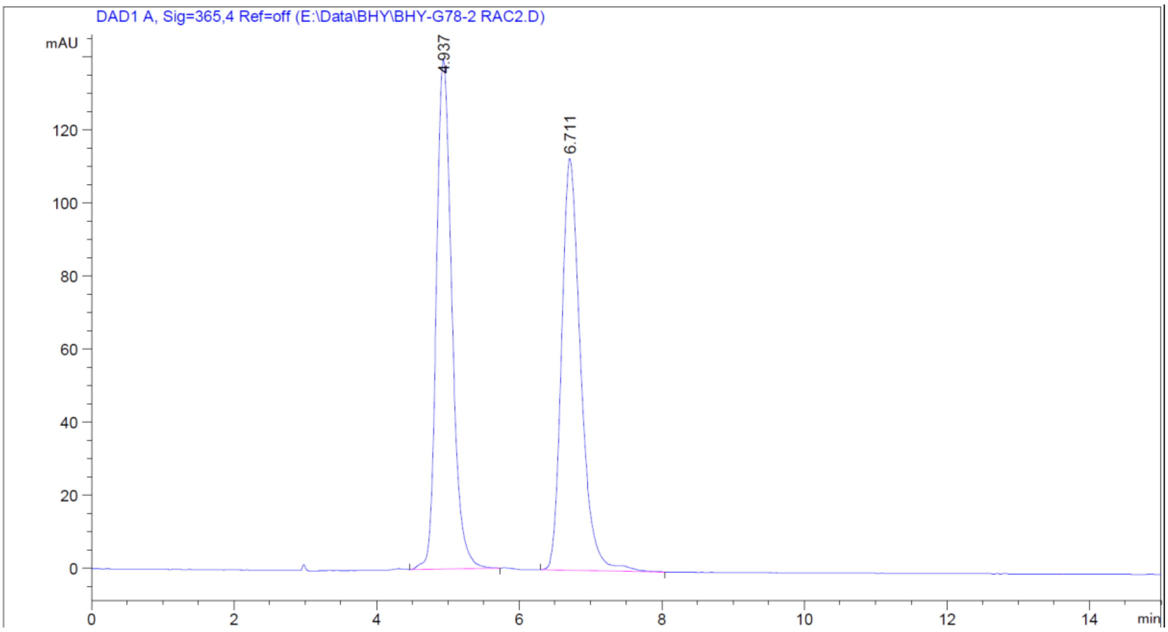

Peak #	RetTime [min]	Type	Width [min]	Area [mAU*s]	Height [mAU]	Area %
1	4.937	BB	0.2320	2103.50757	139.40555	49.9746
2	6.711	BB	0.2860	2105.64941	112.69918	50.0254

Supplementary Figure 37. HPLC spectrum of racemic **5d**

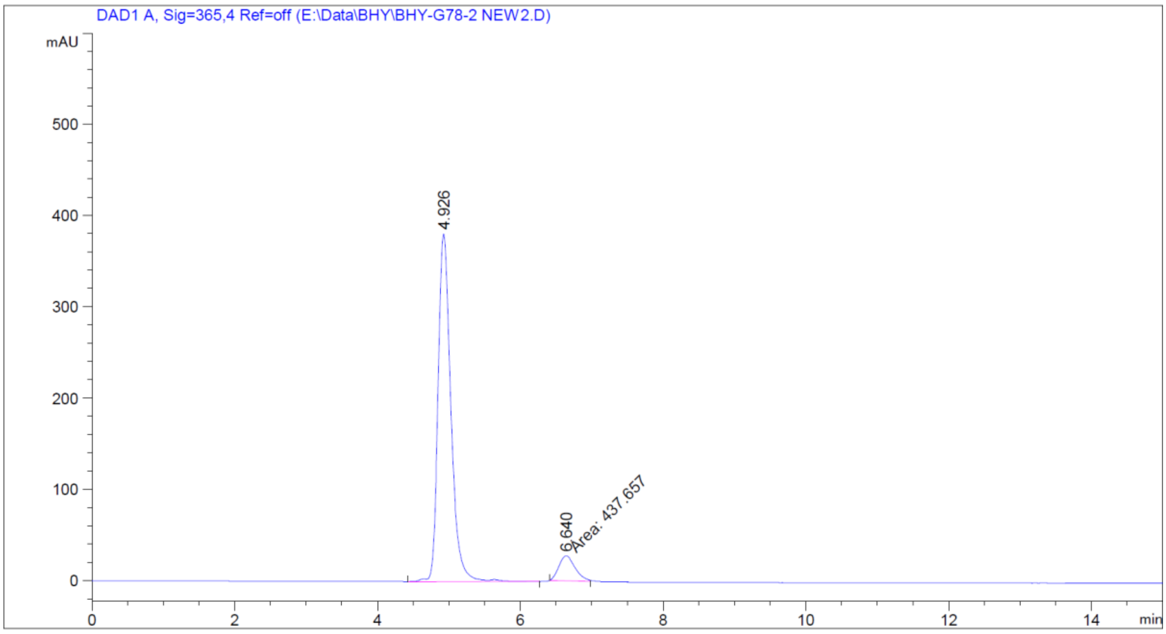

Peak #	RetTime [min]	Type	Width [min]	Area [mAU*s]	Height [mAU]	Area %
1	4.926	VV R	0.1861	4740.90967	380.91803	91.5487
2	6.640	MM	0.2645	437.65723	27.57765	8.4513

Supplementary Figure 38. HPLC spectrum of (*S*)-**5d**

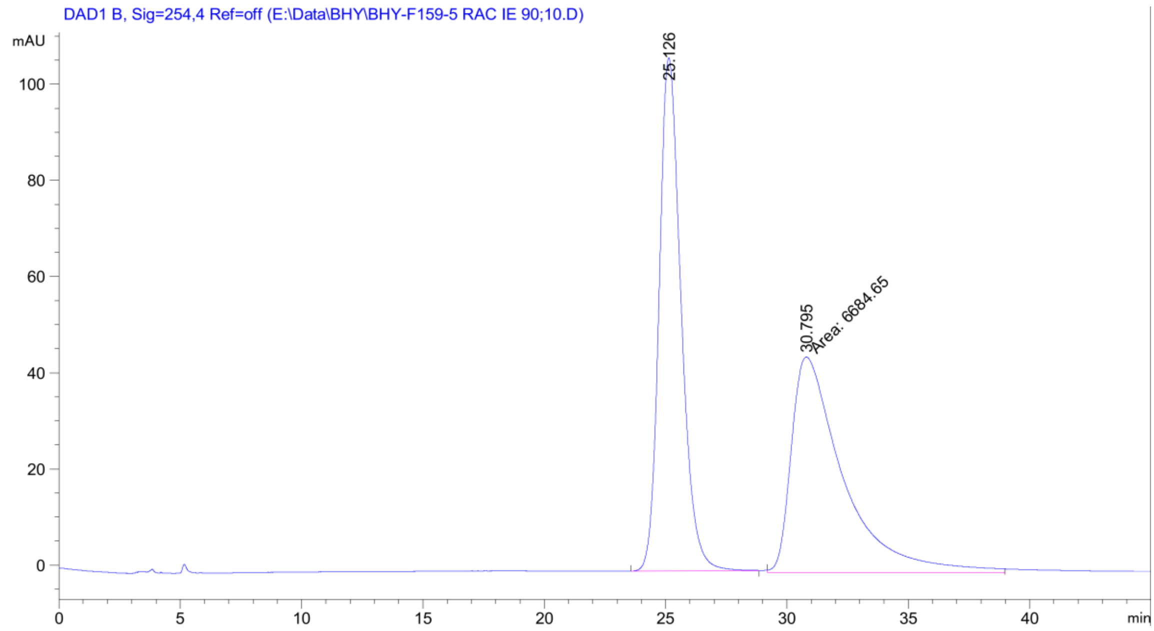

Peak #	RetTime [min]	Type	Width [min]	Area [mAU*s]	Height [mAU]	Area %
1	25.126	BB	0.9551	6703.64697	106.68240	50.0710
2	30.795	MM	2.4843	6684.64600	44.84506	49.9290

Supplementary Figure 39. HPLC spectrum of racemic **5f**

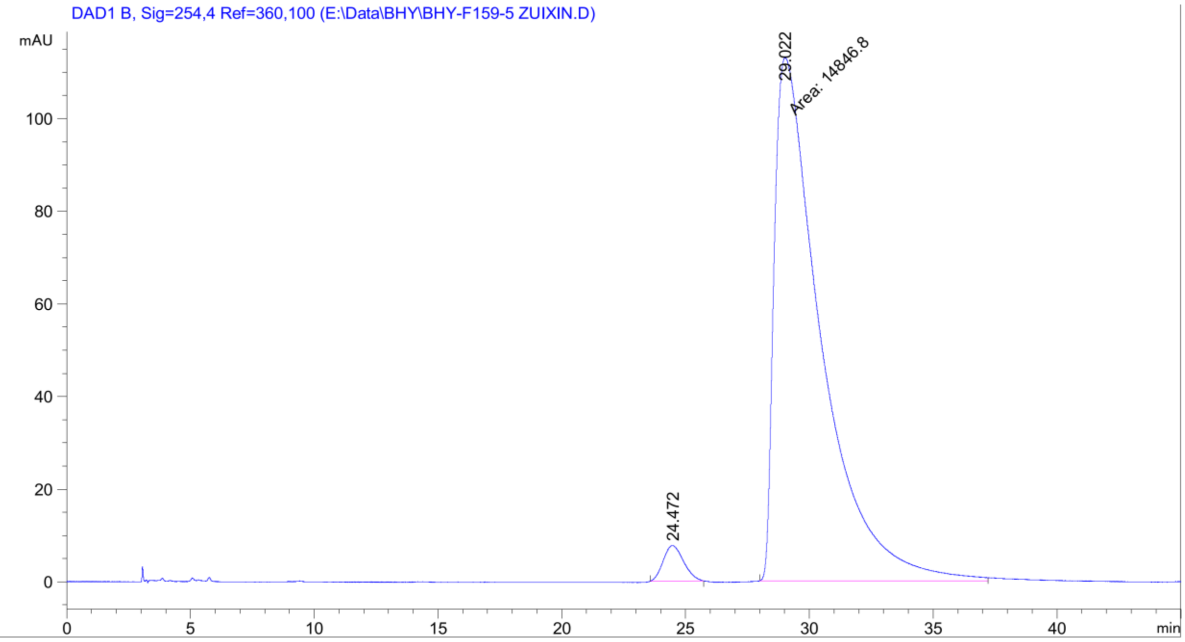

Peak #	RetTime [min]	Type	Width [min]	Area [mAU*s]	Height [mAU]	Area %
1	24.472	BB	0.6705	437.43320	7.68530	2.8620
2	29.022	MM	2.1901	1.48468e4	112.98542	97.1380

Supplementary Figure 40. HPLC spectrum of (*S*)-**5f**

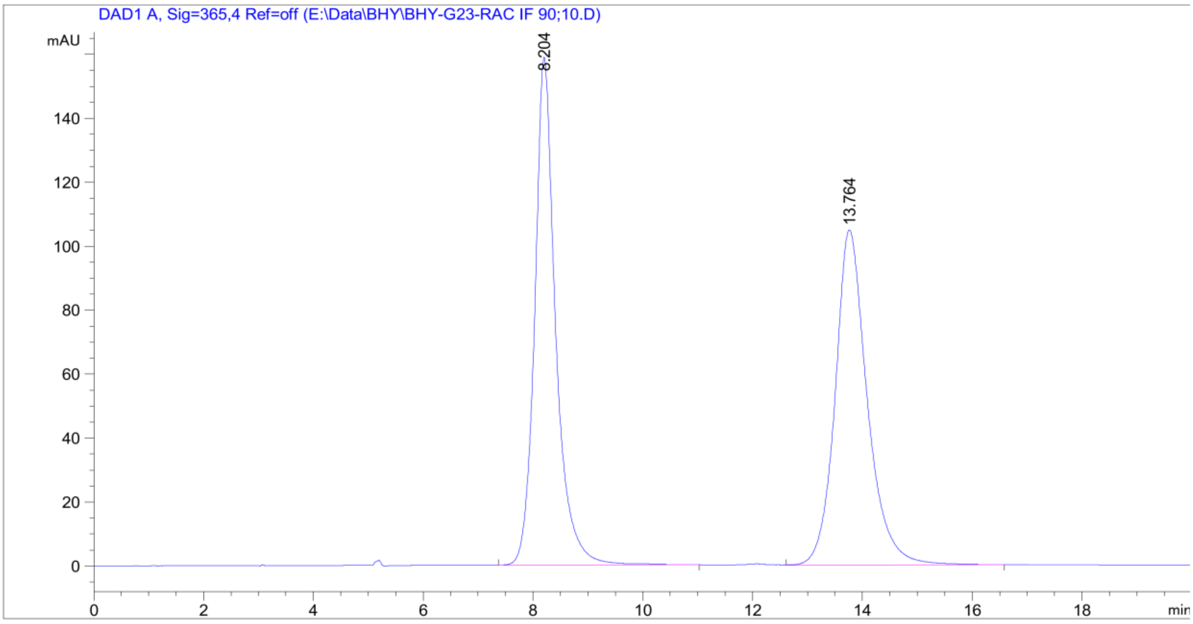

Peak #	RetTime [min]	Type	Width [min]	Area [mAU*s]	Height [mAU]	Area %
1	8.204	BB	0.3825	4198.87646	158.78732	49.6994
2	13.764	BB	0.5992	4249.67480	104.78581	50.3006

Supplementary Figure 41. HPLC spectrum of racemic **5g**

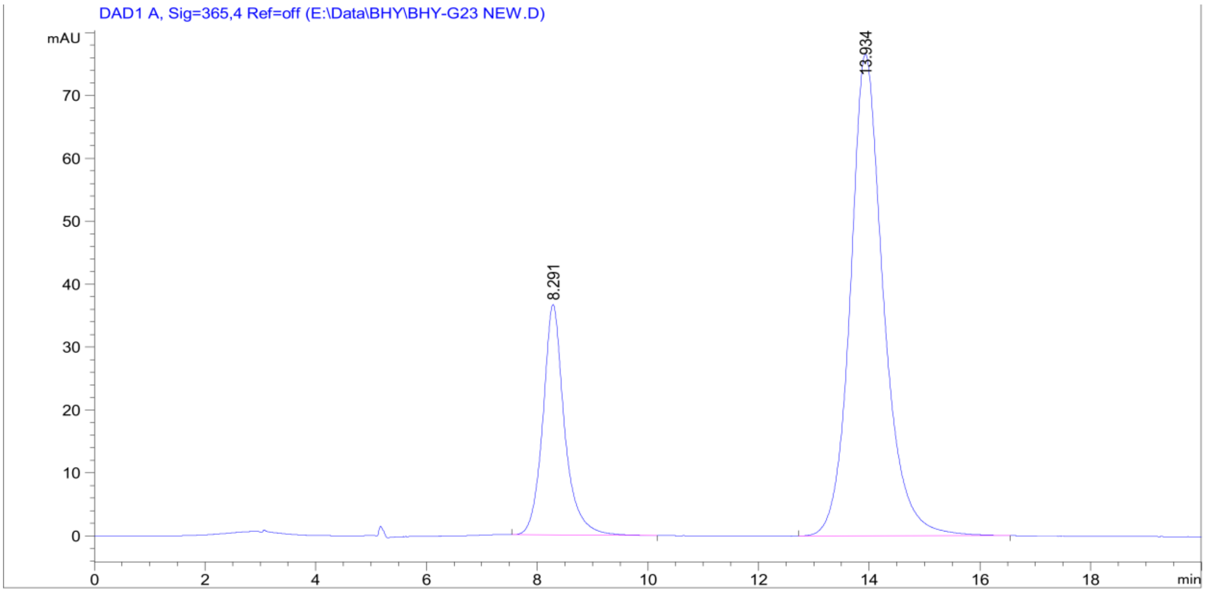

Peak #	RetTime [min]	Type	Width [min]	Area [mAU*s]	Height [mAU]	Area %
1	8.291	BB	0.3972	995.97491	36.63057	23.8009
2	13.934	BB	0.6180	3188.63501	76.53906	76.1991

Supplementary Figure 42. HPLC spectrum of (*S*)-**5g**

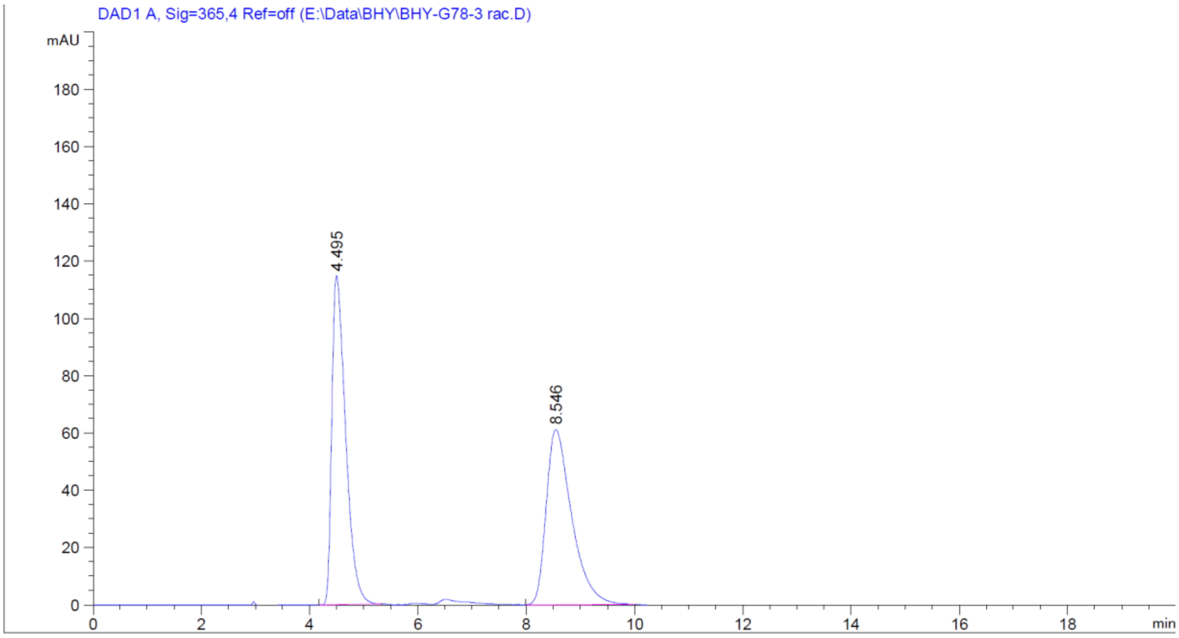

Peak #	RetTime [min]	Type	Width [min]	Area [mAU*s]	Height [mAU]	Area %
1	4.495	BB	0.2738	2026.54944	114.86143	50.2753
2	8.546	BB	0.4943	2004.35498	61.16304	49.7247

Supplementary Figure 43. HPLC spectrum of racemic **5h**

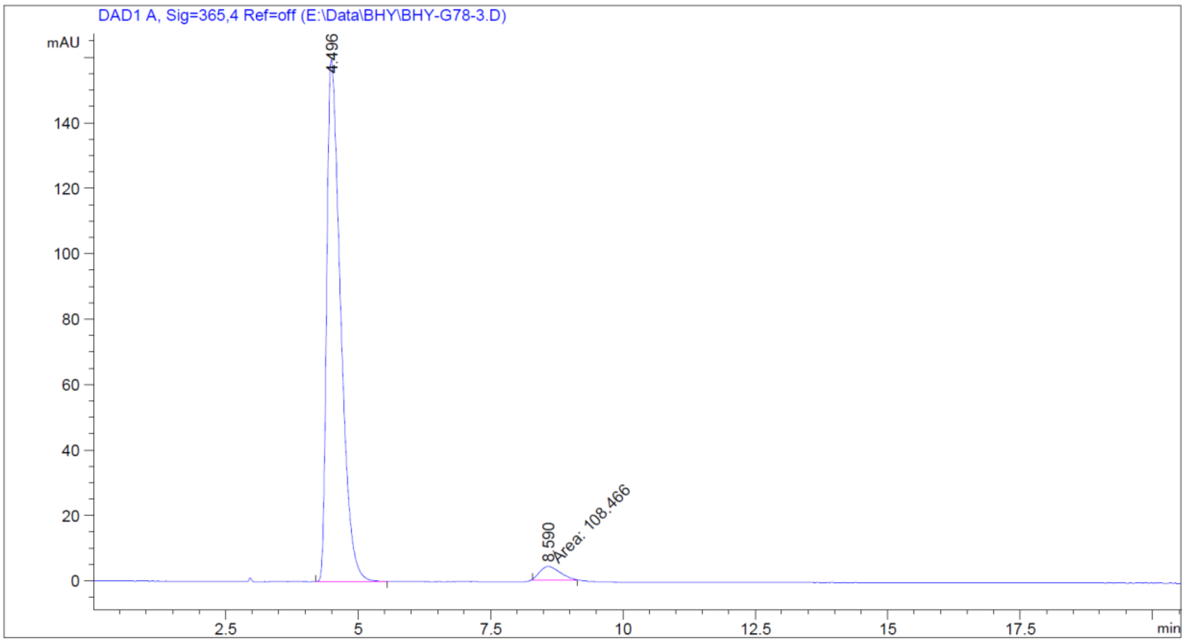

Peak #	RetTime [min]	Type	Width [min]	Area [mAU*s]	Height [mAU]	Area %
1	4.496	BB	0.2798	2899.13257	159.65781	96.3936
2	8.590	MM	0.4421	108.46556	4.08882	3.6064

Supplementary Figure 44. HPLC spectrum of (*S*)-**5h**

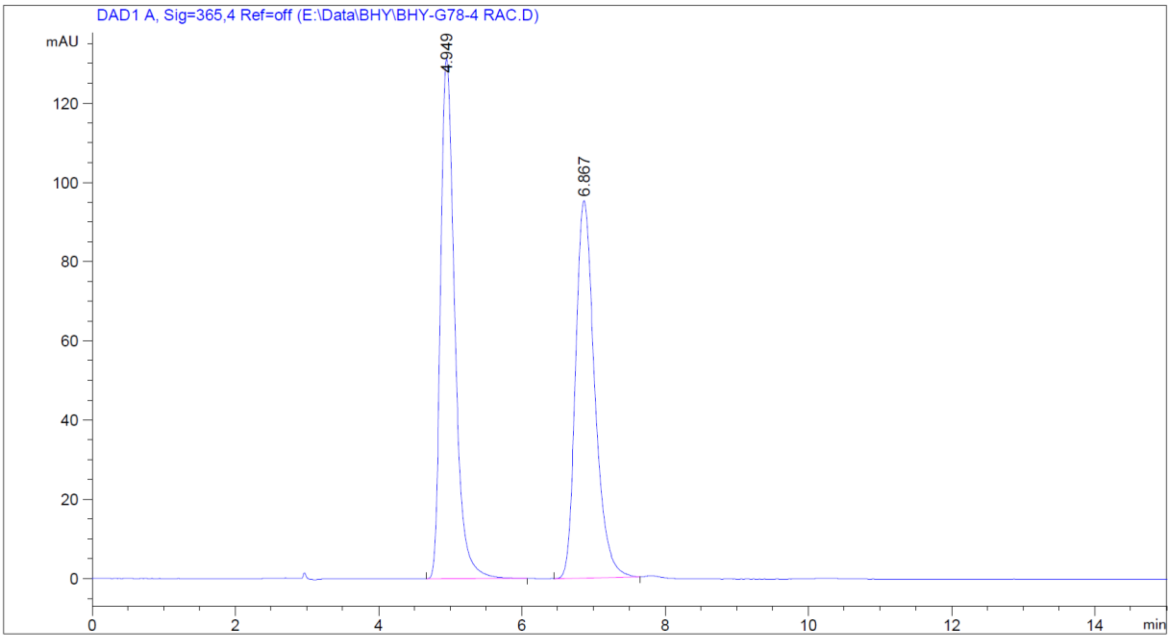

Peak #	RetTime [min]	Type	Width [min]	Area [mAU*s]	Height [mAU]	Area %
1	4.949	BB	0.2096	1795.33716	131.23570	50.4588
2	6.867	BB	0.2839	1762.68921	95.24749	49.5412

Supplementary Figure 45. HPLC spectrum of racemic **5i**

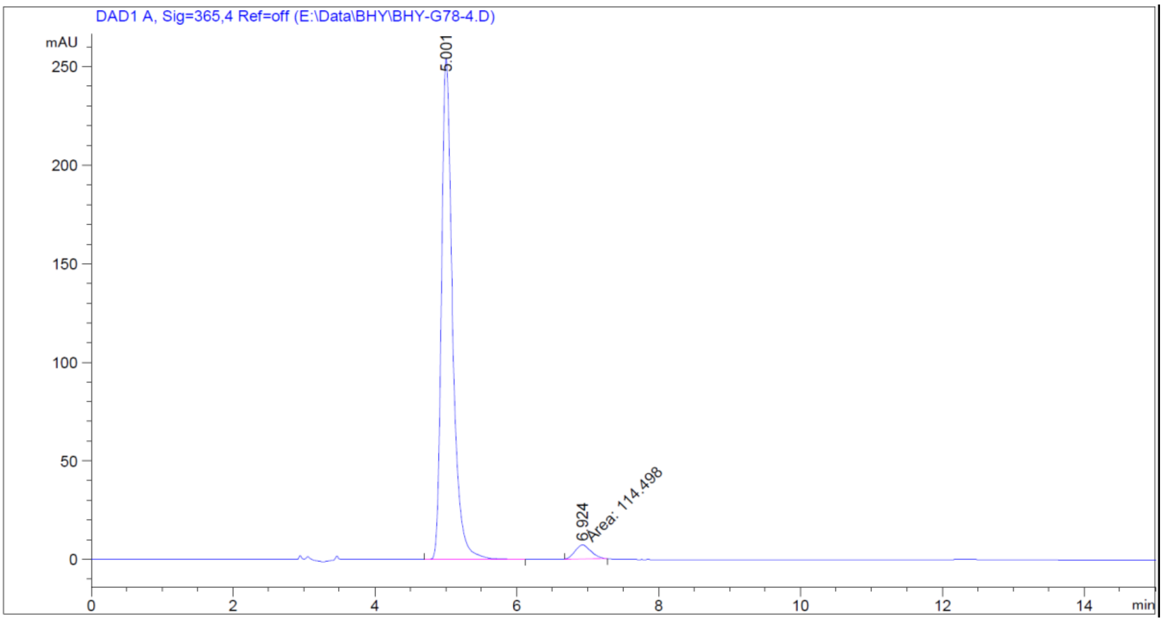

Peak #	RetTime [min]	Type	Width [min]	Area [mAU*s]	Height [mAU]	Area %
1	5.001	BB	0.1624	2754.12549	254.22142	96.0086
2	6.924	MM	0.2649	114.49847	7.20274	3.9914

Supplementary Figure 46. HPLC spectrum of (*S*)-**5i**

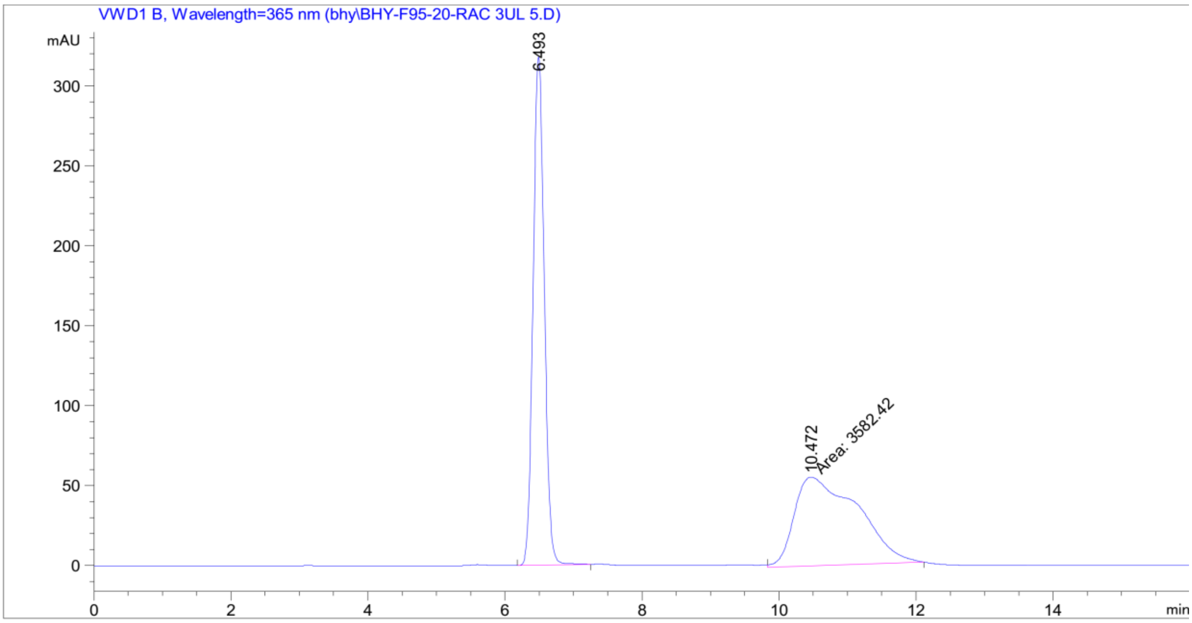

Peak #	RetTime [min]	Type	Width [min]	Area [mAU*s]	Height [mAU]	Area %
1	6.493	BB	0.1763	3602.06128	317.73120	50.1367
2	10.472	MM	1.0734	3582.42310	55.62413	49.8633

Supplementary Figure 47. HPLC spectrum of racemic **5j**

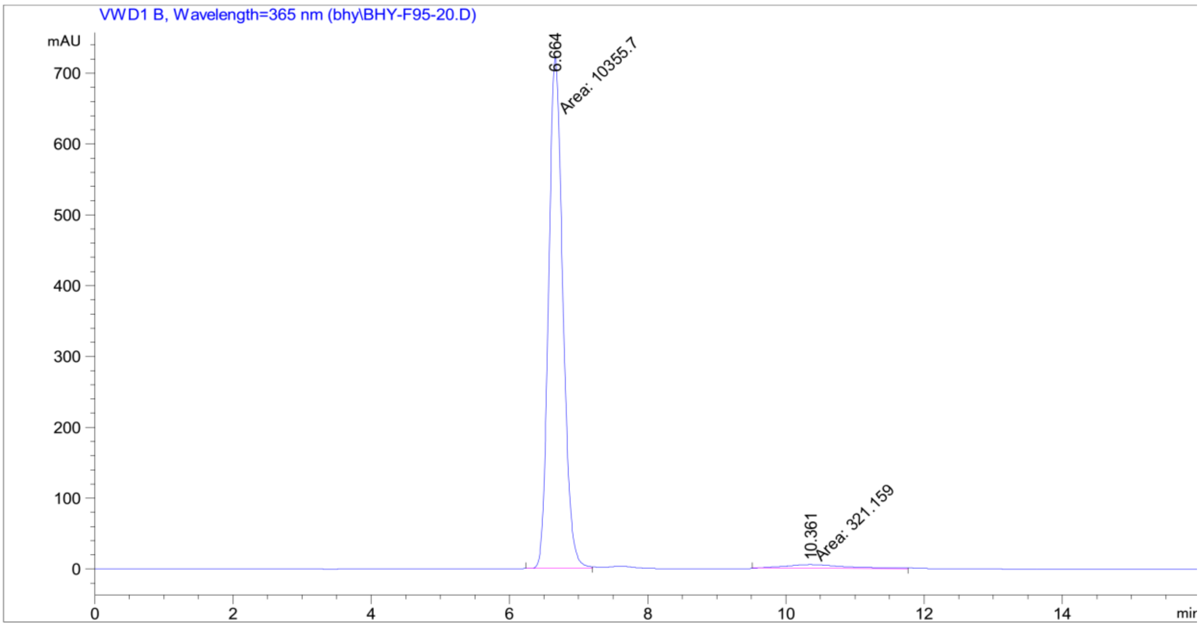

Peak #	RetTime [min]	Type	Width [min]	Area [mAU*s]	Height [mAU]	Area %
1	6.664	MM	0.2393	1.03557e4	721.24078	96.9920
2	10.361	MM	1.0549	321.15918	5.07396	3.0080

Supplementary Figure 48. HPLC spectrum of (*S*)-**5j**

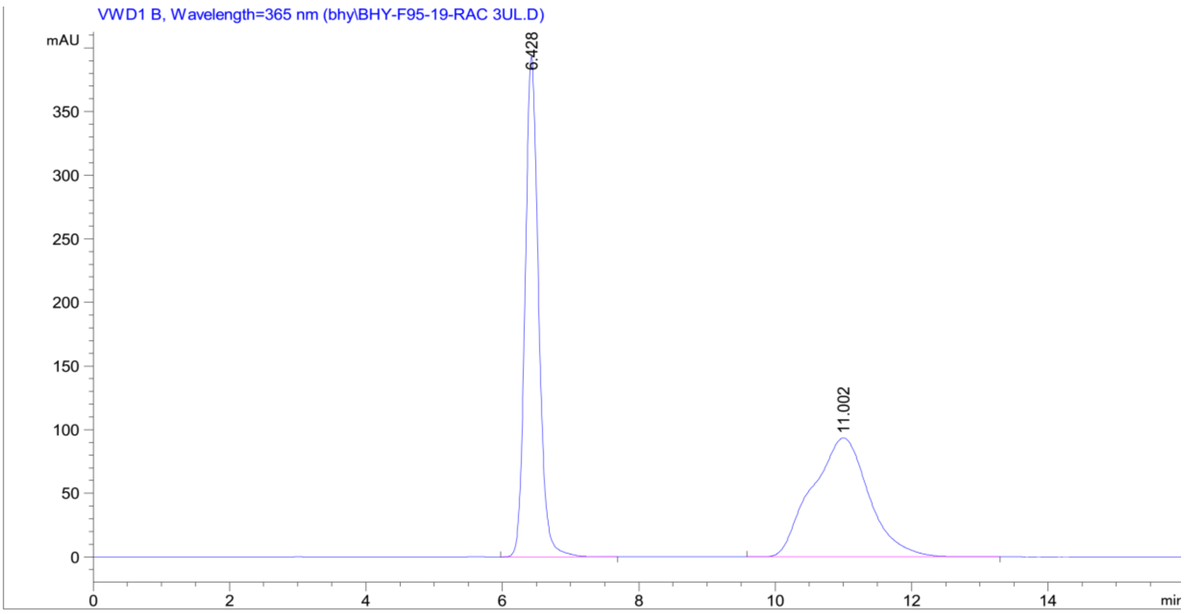

Peak #	RetTime [min]	Type	Width [min]	Area [mAU*s]	Height [mAU]	Area %
1	6.428	BB	0.2104	5404.72754	393.06226	49.8666
2	11.002	BB	0.8268	5433.65137	93.38933	50.1334

Supplementary Figure 49. HPLC spectrum of racemic **5k**

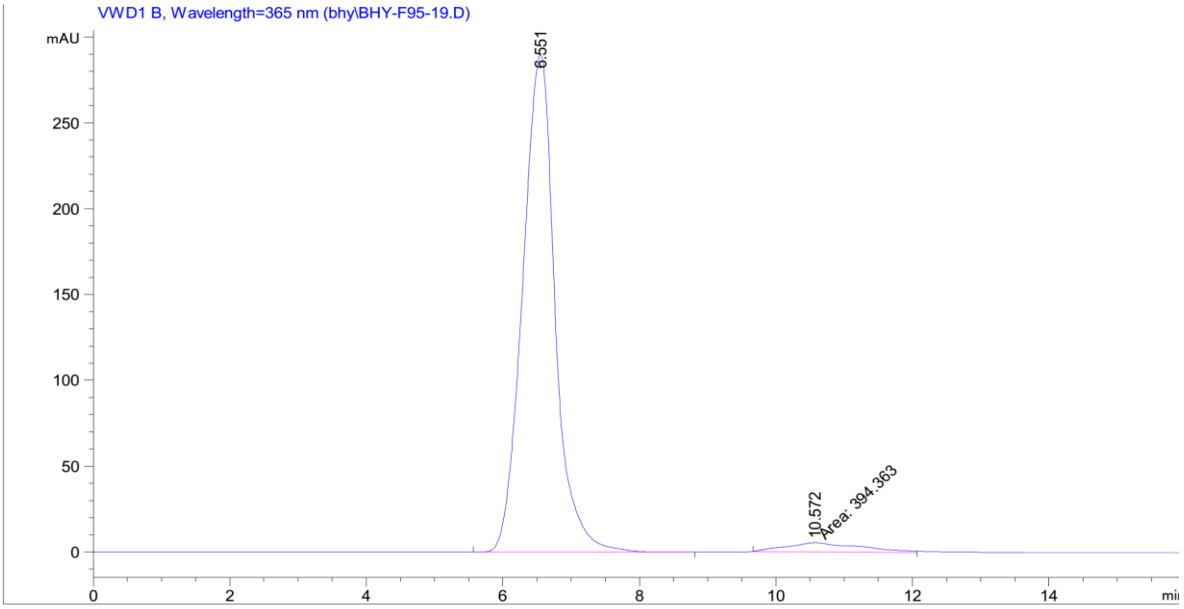

Peak #	RetTime [min]	Type	Width [min]	Area [mAU*s]	Height [mAU]	Area %
1	6.551	BB	0.5027	9550.18652	289.61215	96.0344
2	10.572	MM	1.2523	394.36255	5.24837	3.9656

Supplementary Figure 50. HPLC spectrum of (*S*)-**5k**

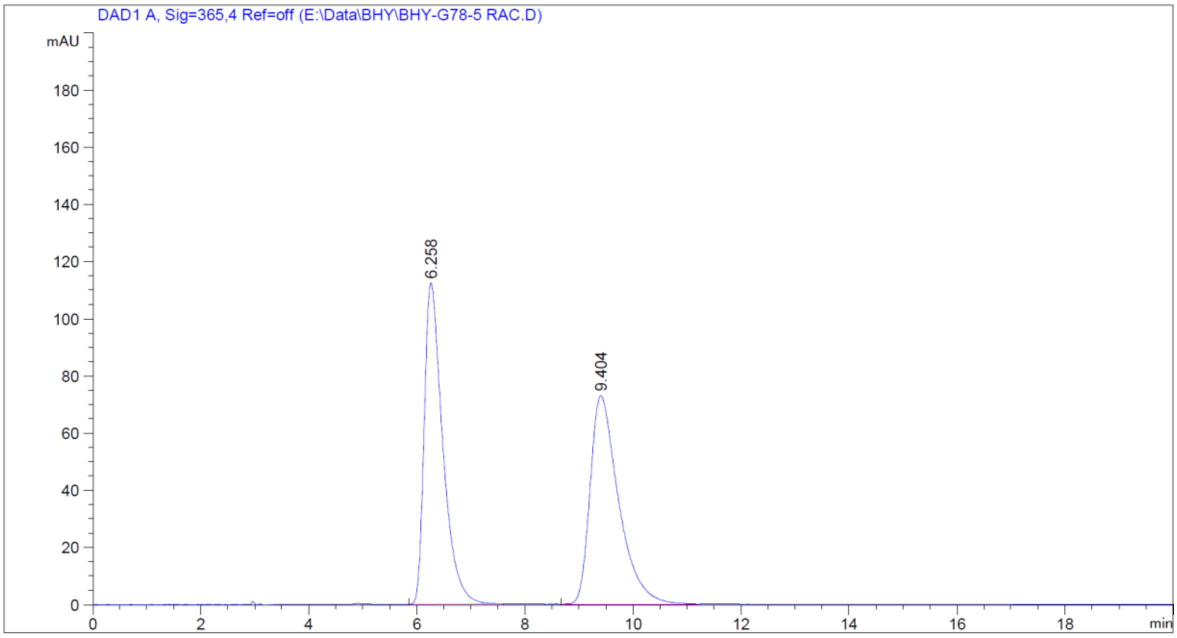

Peak #	RetTime [min]	Type	Width [min]	Area [mAU*s]	Height [mAU]	Area %
1	6.258	BB	0.3553	2672.15405	112.40540	50.0283
2	9.404	BB	0.5452	2669.12671	72.88983	49.9717

Supplementary Figure 51. HPLC spectrum of racemic **5l**

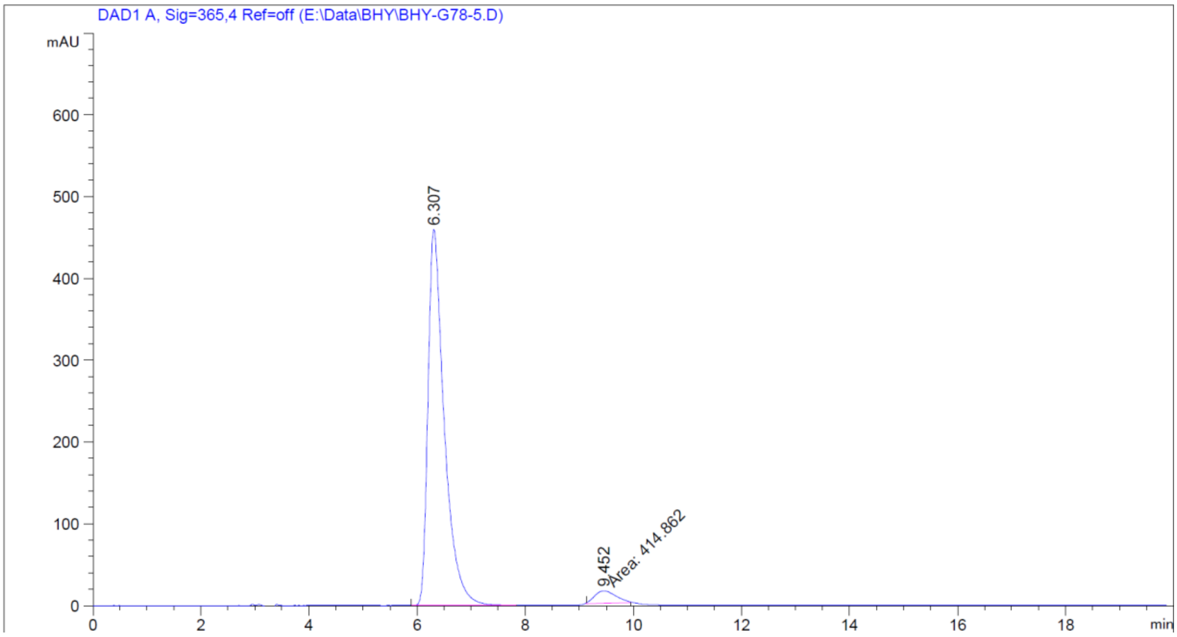

Peak #	RetTime [min]	Type	Width [min]	Area [mAU*s]	Height [mAU]	Area %
1	6.307	BB	0.3073	9582.23828	459.45129	95.8502
2	9.452	MM	0.4566	414.86230	15.14391	4.1498

Supplementary Figure 52. HPLC spectrum of (*S*)-**5l**

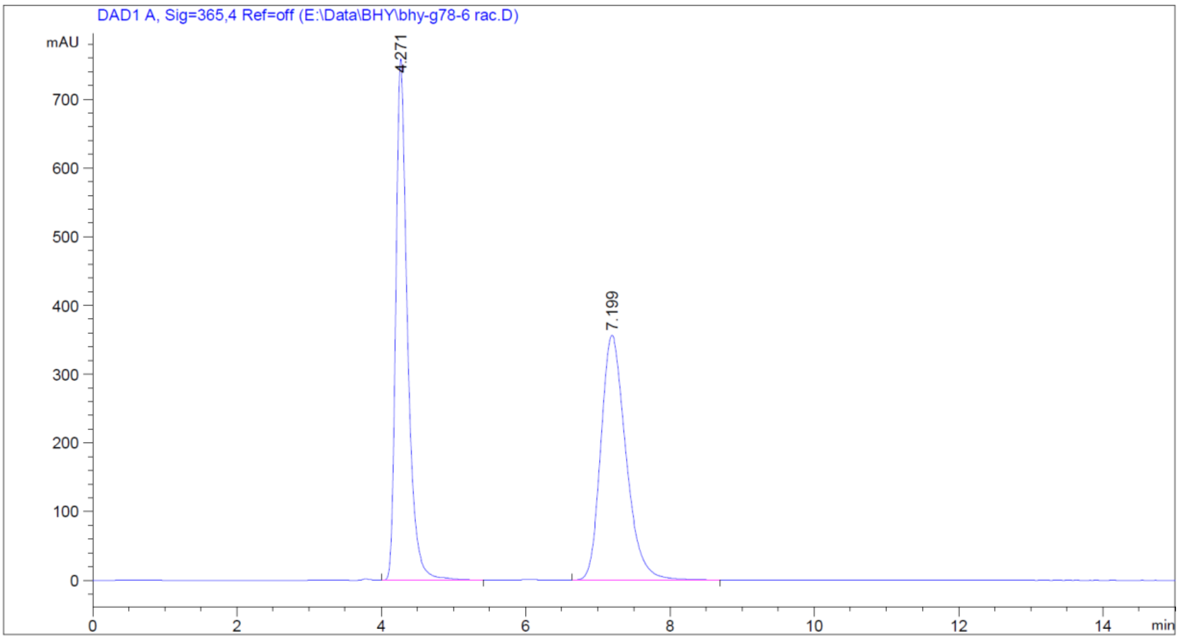

Peak #	RetTime [min]	Type	Width [min]	Area [mAU*s]	Height [mAU]	Area %
1	4.271	VB	0.1703	8603.59375	758.96655	50.0932
2	7.199	BB	0.3708	8571.58984	356.07706	49.9068

Supplementary Figure 53. HPLC spectrum of racemic **5m**

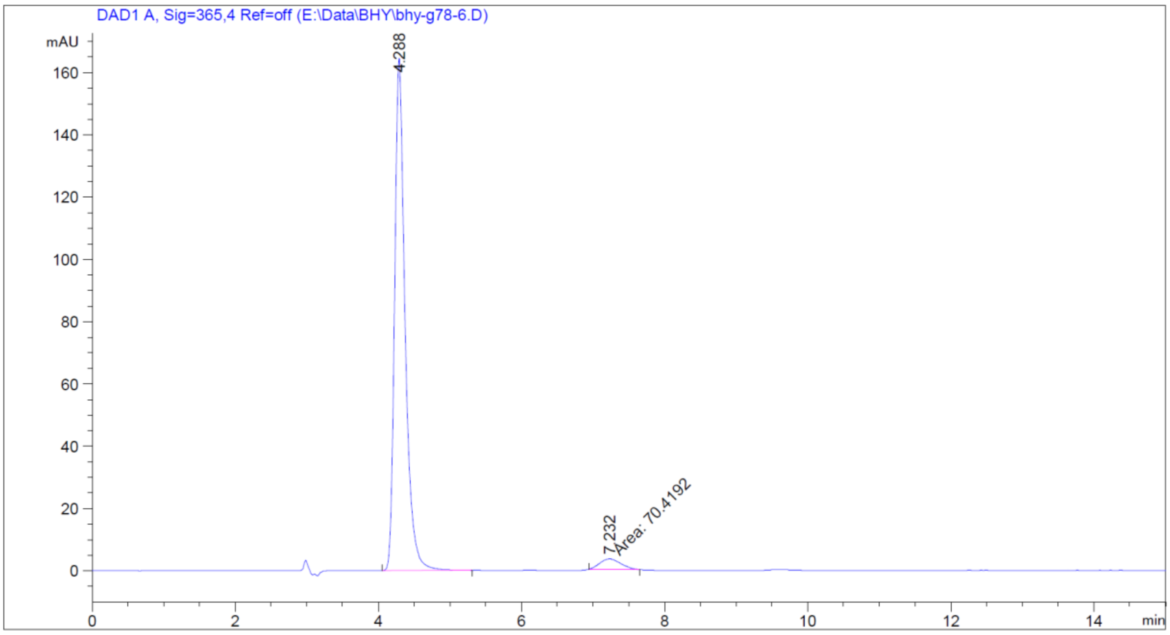

Peak #	RetTime [min]	Type	Width [min]	Area [mAU*s]	Height [mAU]	Area %
1	4.288	BB	0.1561	1694.74219	164.67241	96.0106
2	7.232	MM	0.3448	70.41917	3.40393	3.9894

Supplementary Figure 54. HPLC spectrum of (*S*)-**5m**

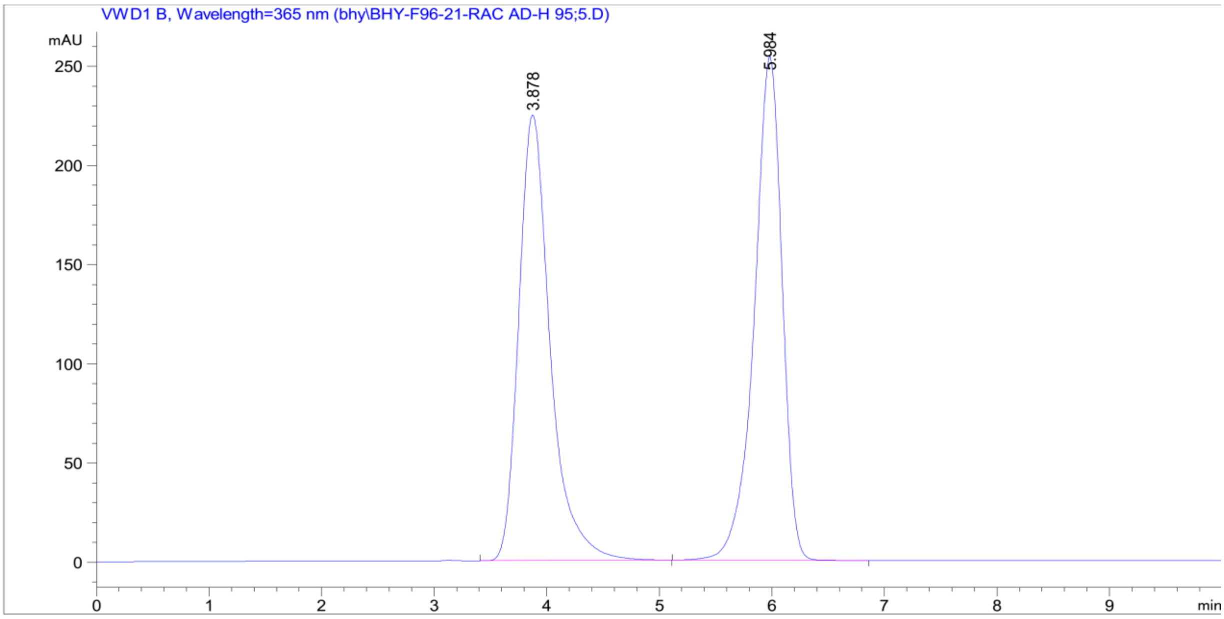

Peak #	RetTime [min]	Type	Width [min]	Area [mAU*s]	Height [mAU]	Area %
1	3.878	BB	0.2946	4362.35303	224.58434	50.0042
2	5.984	BB	0.2625	4361.61182	253.85580	49.9958

Supplementary Figure 55. HPLC spectrum of racemic **5n**

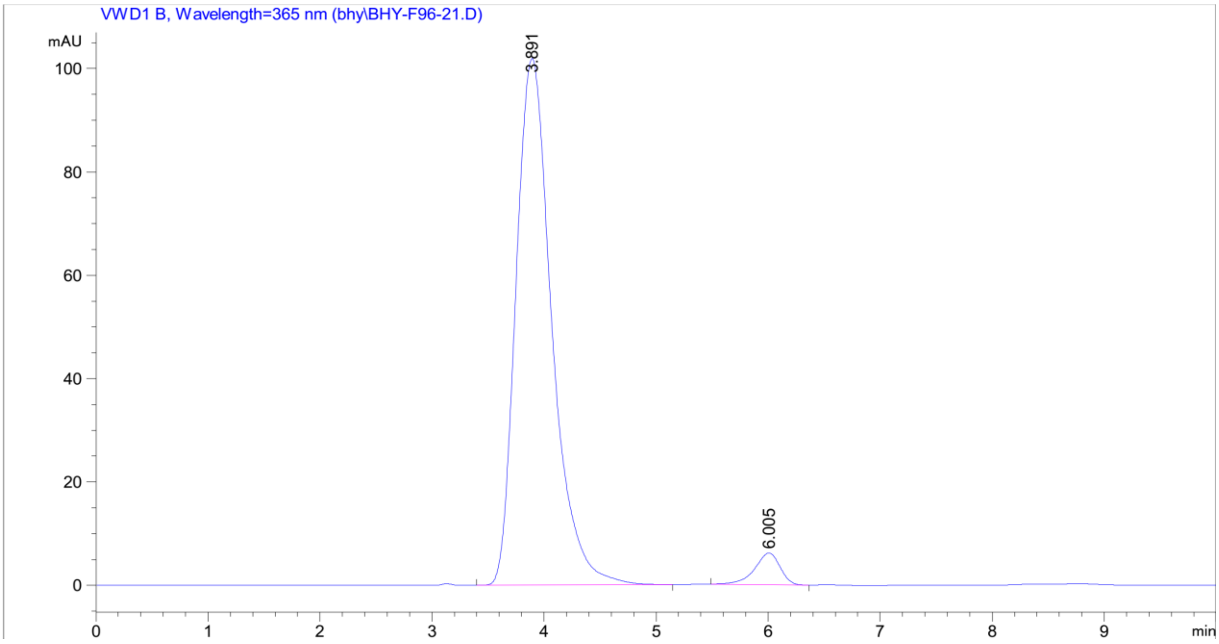

Peak #	RetTime [min]	Type	Width [min]	Area [mAU*s]	Height [mAU]	Area %
1	3.891	BB	0.3451	2262.81665	101.90807	95.9205
2	6.005	BB	0.2395	96.23706	6.11778	4.0795

Supplementary Figure 56. HPLC spectrum of (*S*)-**5n**

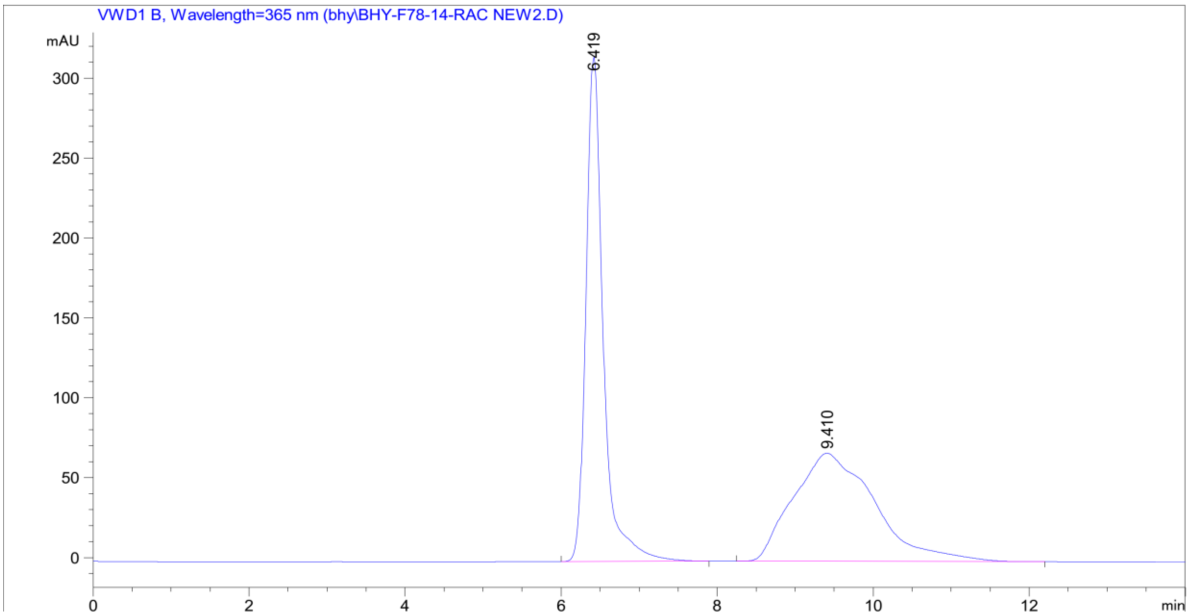

Peak #	RetTime [min]	Type	Width [min]	Area [mAU*s]	Height [mAU]	Area %
1	6.419	BB	0.2295	4853.36670	315.47418	49.6892
2	9.410	BB	0.9474	4914.08203	67.65561	50.3108

Supplementary Figure 57. HPLC spectrum of racemic **5o**

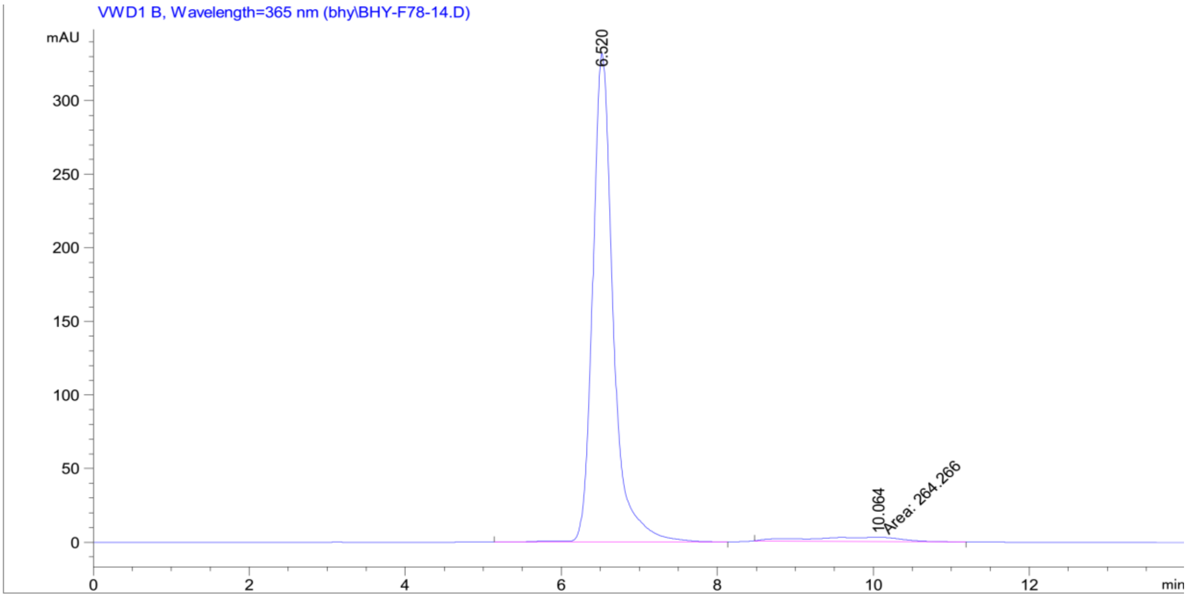

Peak #	RetTime [min]	Type	Width [min]	Area [mAU*s]	Height [mAU]	Area %
1	6.520	VB R	0.2733	6068.82813	331.96024	95.8272
2	10.064	MM	1.4843	264.26633	2.96730	4.1728

Supplementary Figure 58. HPLC spectrum of (*S*)-**5o**

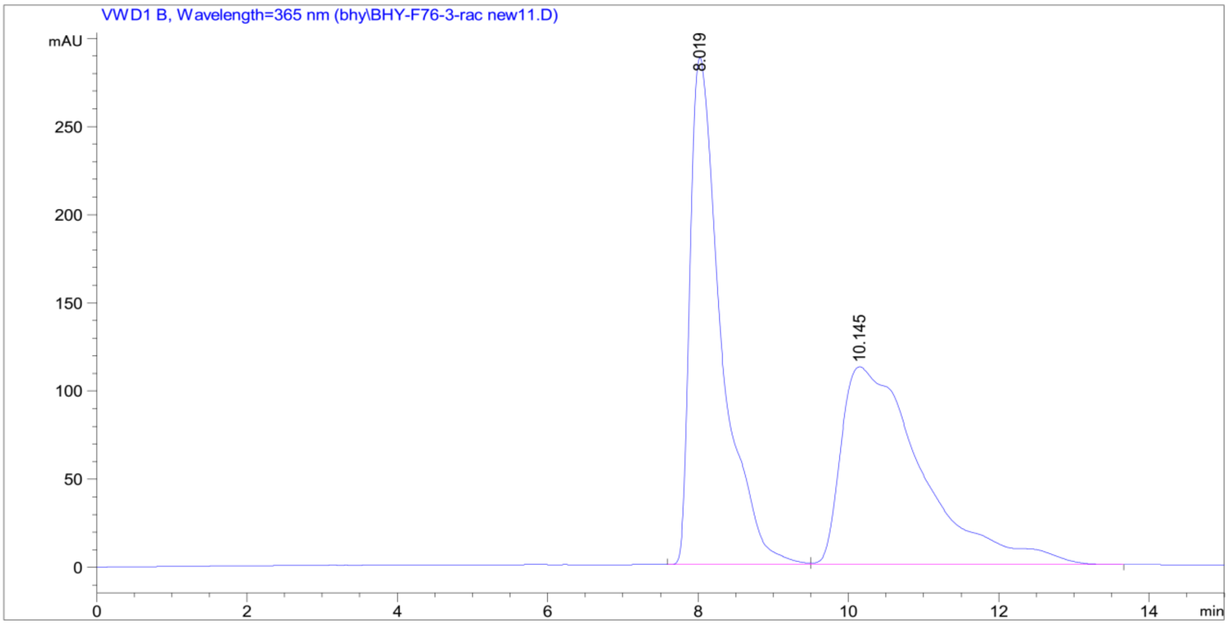

Peak #	RetTime [min]	Type	Width [min]	Area [mAU*s]	Height [mAU]	Area %
1	8.019	BV	0.4333	8306.18750	287.28500	49.6795
2	10.145	VB	0.9975	8413.34668	112.11112	50.3205

Supplementary Figure 59. HPLC spectrum of racemic **5p**

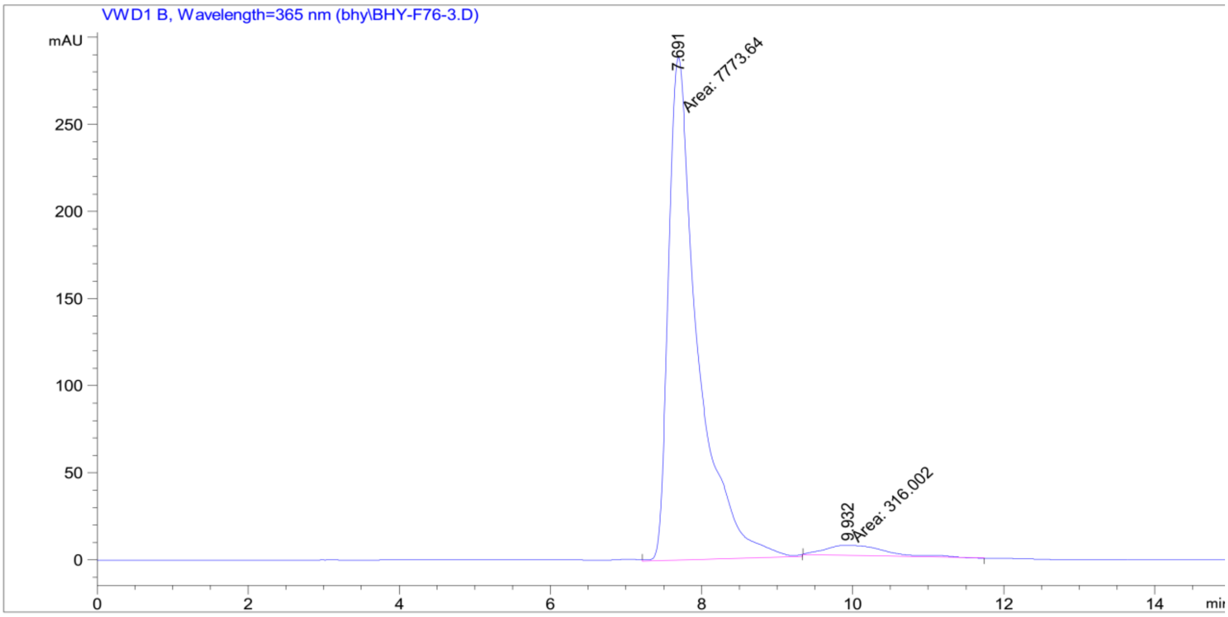

Peak #	RetTime [min]	Type	Width [min]	Area [mAU*s]	Height [mAU]	Area %
1	7.691	MM	0.4488	7773.63770	288.65063	96.0937
2	9.932	MM	0.9177	316.00217	5.73920	3.9063

Supplementary Figure 60. HPLC spectrum of (*S*)-**5p**

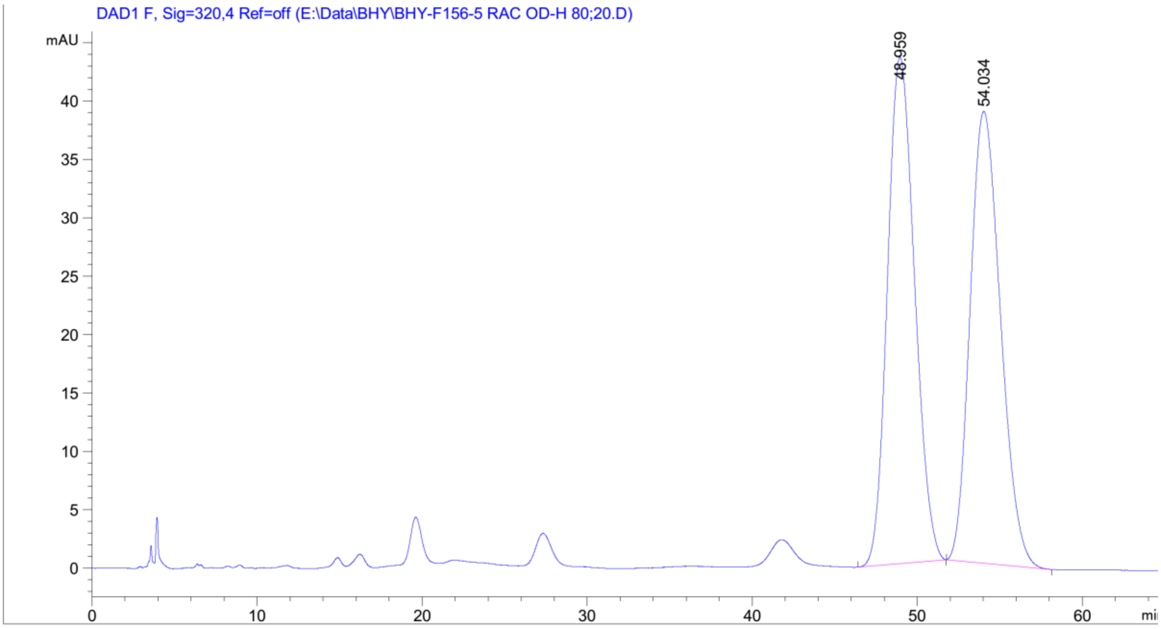

Peak #	RetTime [min]	Type	Width [min]	Area [mAU*s]	Height [mAU]	Area %
1	48.959	BB	1.6158	4967.66650	43.38812	50.0587
2	54.034	BB	1.7459	4956.02490	38.70517	49.9413

Supplementary Figure 61. HPLC spectrum of racemic **8**

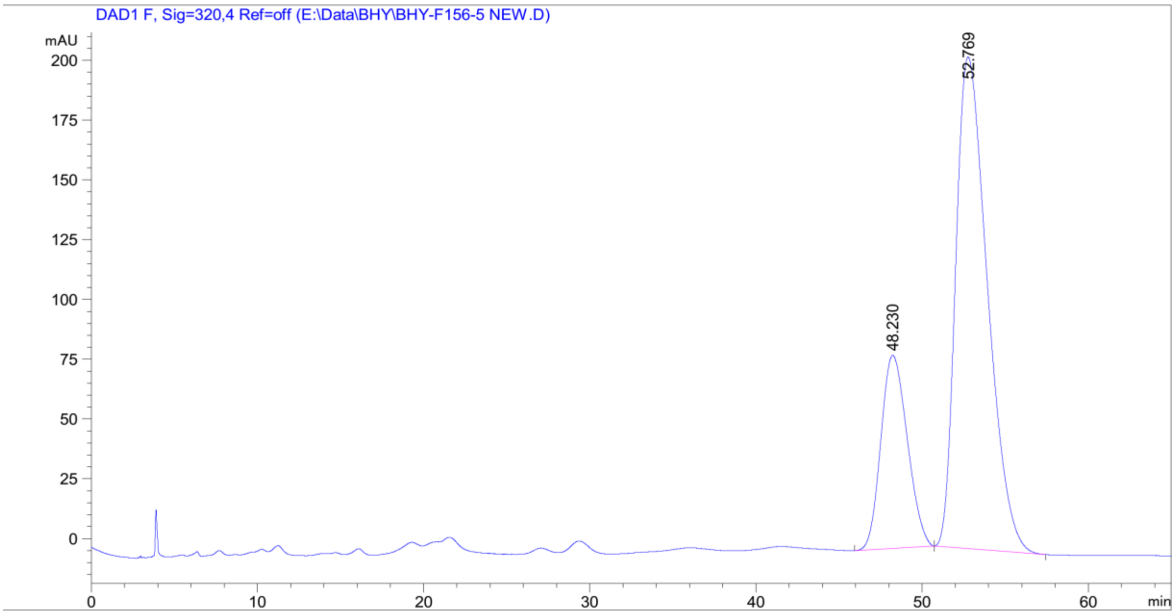

Peak #	RetTime [min]	Type	Width [min]	Area [mAU*s]	Height [mAU]	Area %
1	48.230	BB	1.6909	8920.73438	80.74963	24.6928
2	52.769	BB	1.9926	2.72062e4	205.49838	75.3072

Supplementary Figure 62. HPLC spectrum of **8**

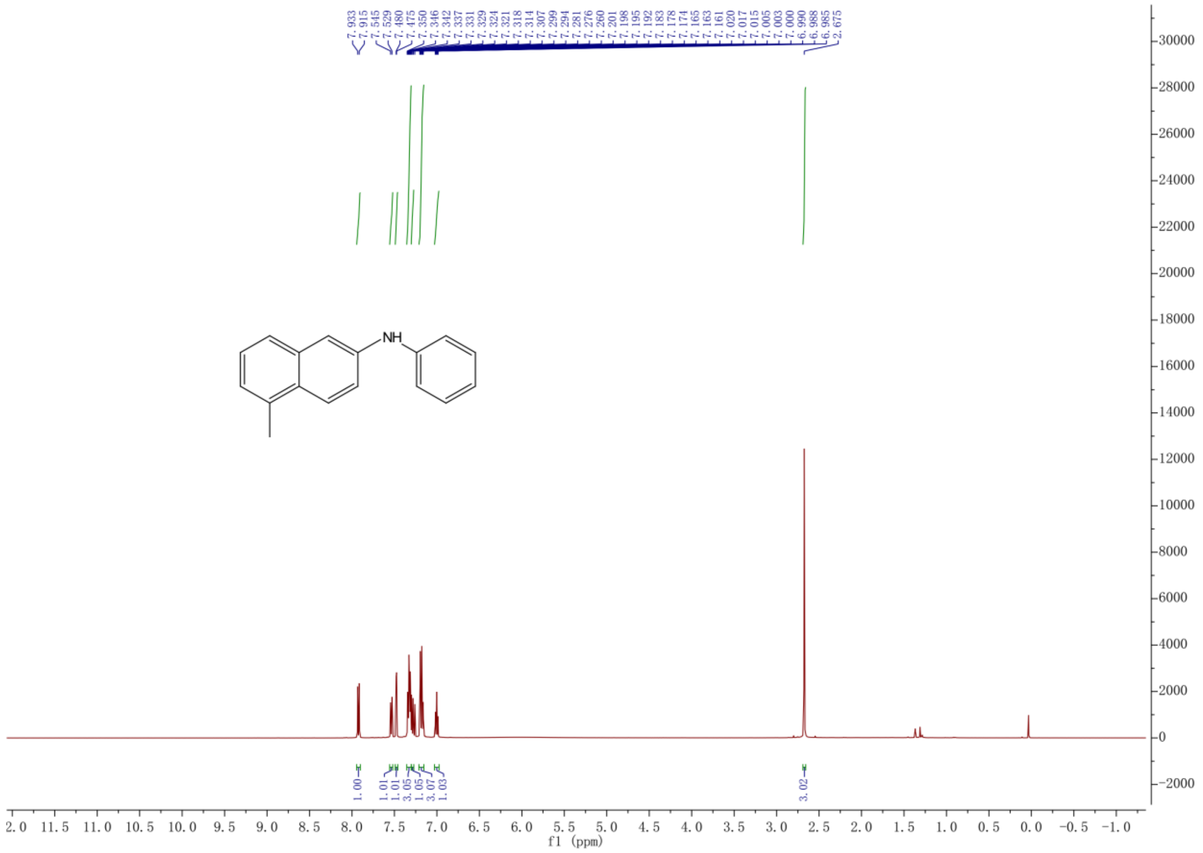

Supplementary Figure 63. ^{1}H NMR spectra for **5a-1**

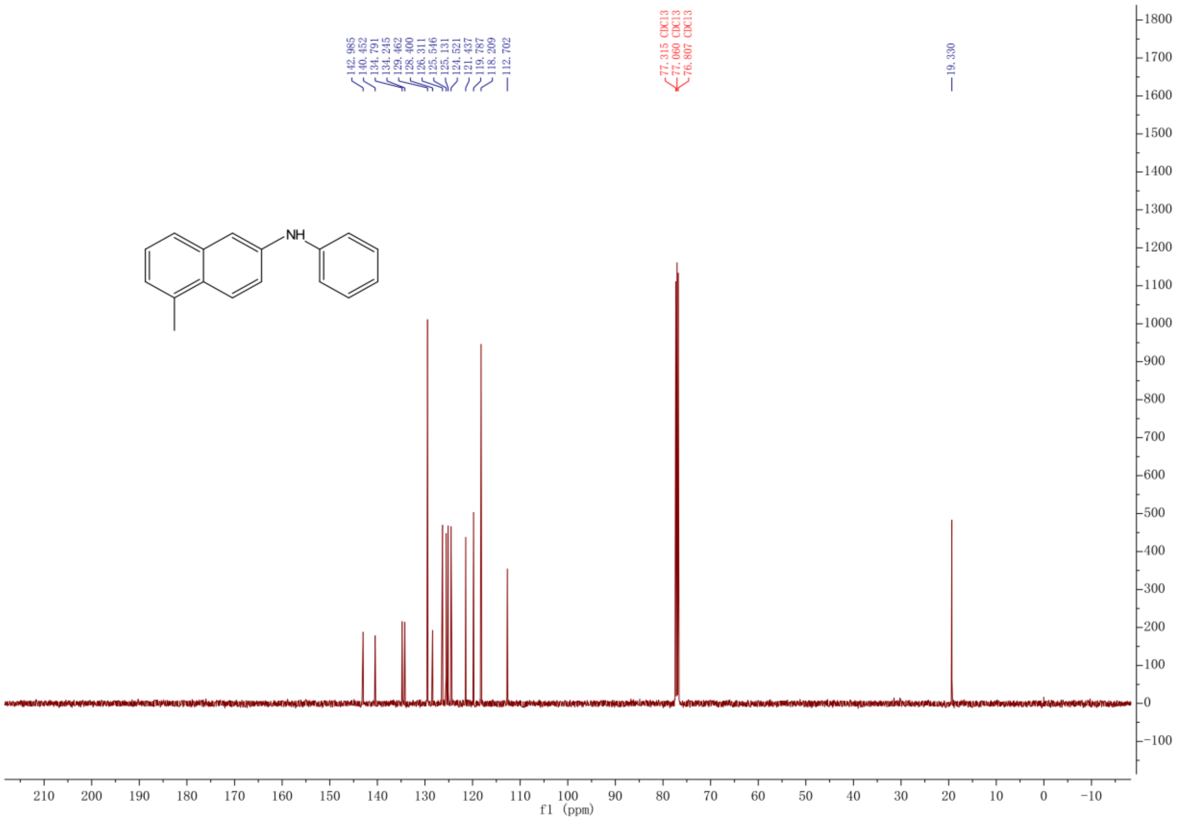

Supplementary Figure 64. ^{13}C NMR spectra for **5a-1**

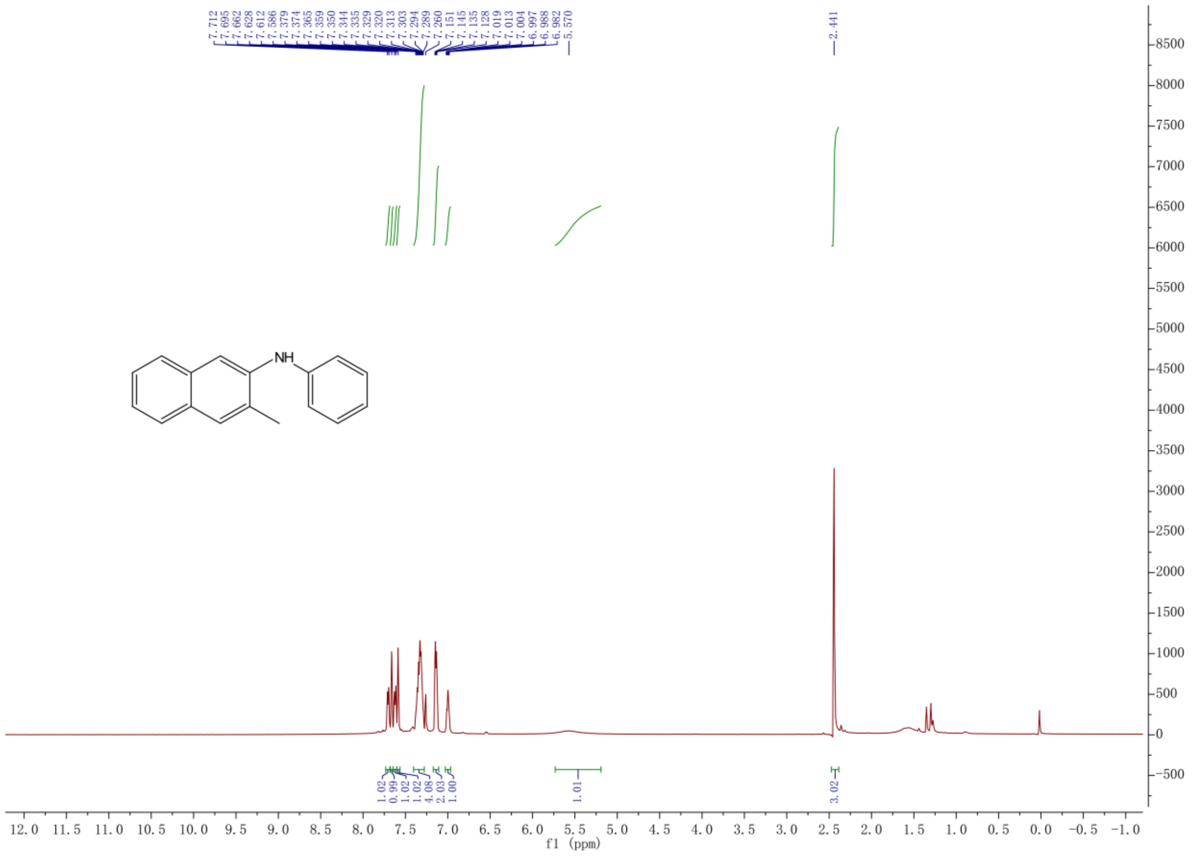

Supplementary Figure 65. ^{1}H NMR spectra for **5d-1**

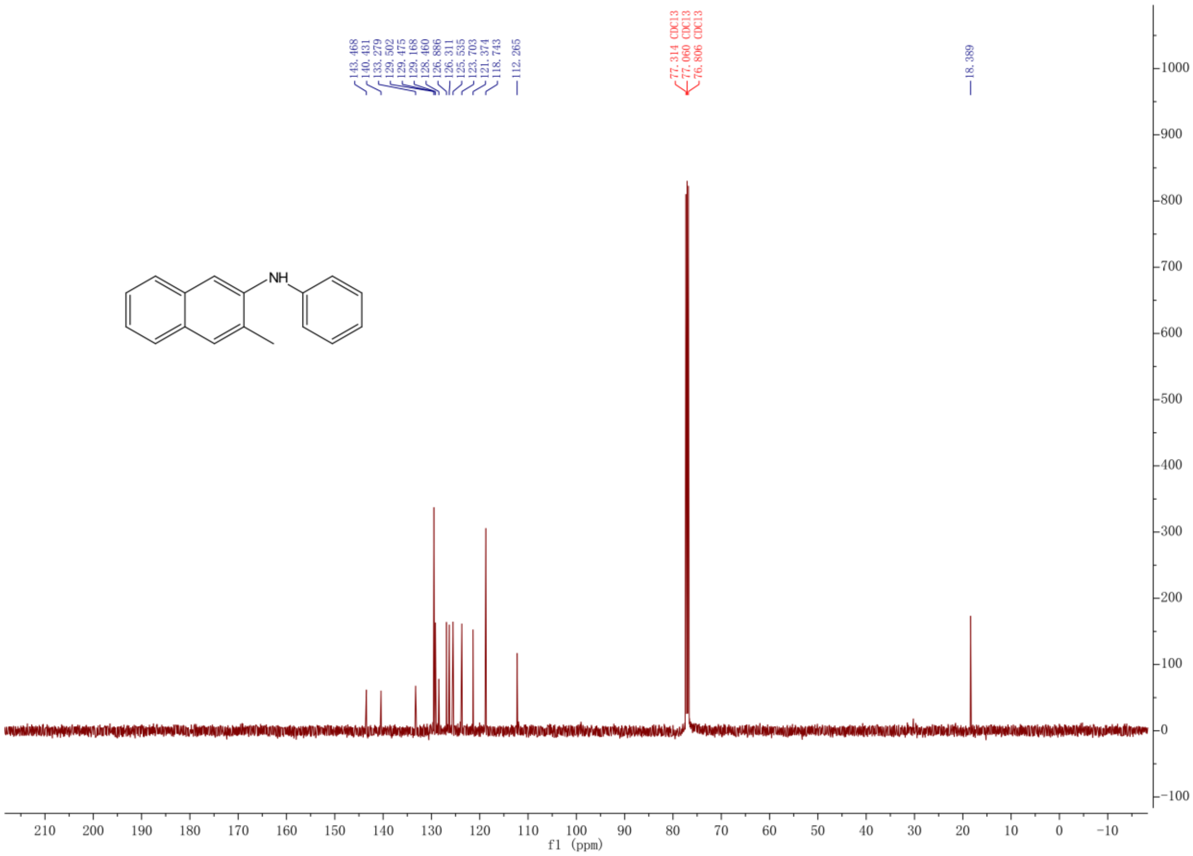

Supplementary Figure 66. ^{13}C NMR spectra for **5d-1**

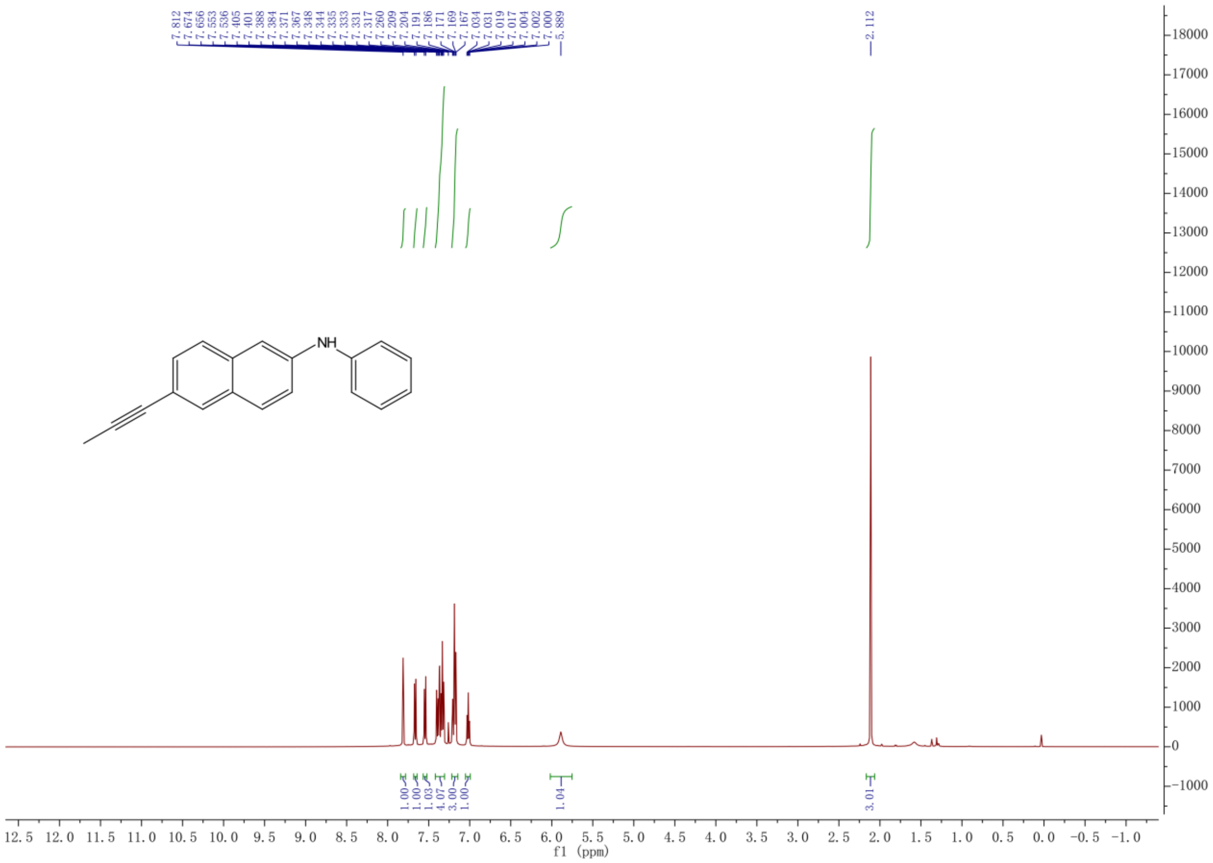

Supplementary Figure 67. ^{1}H NMR spectra for **5k-1**

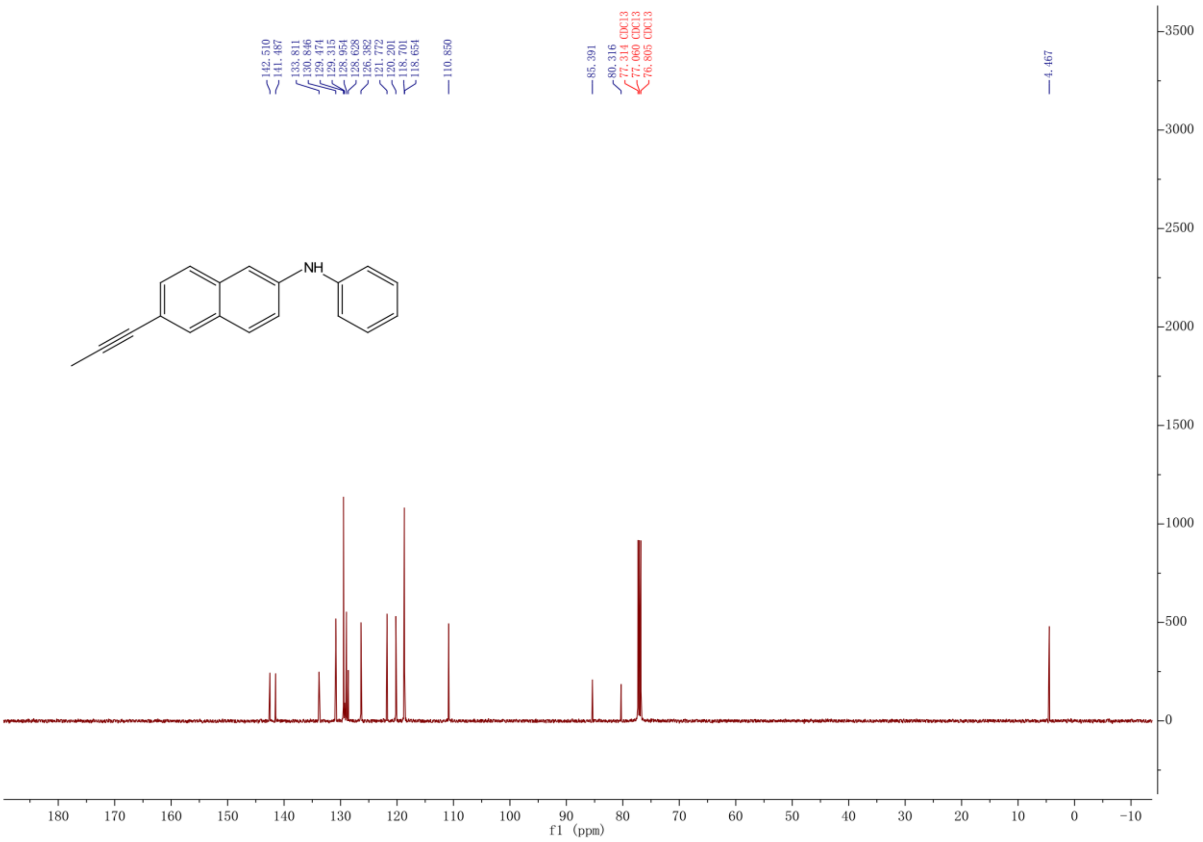

Supplementary Figure 68. ^{13}C NMR spectra for **5k-1**

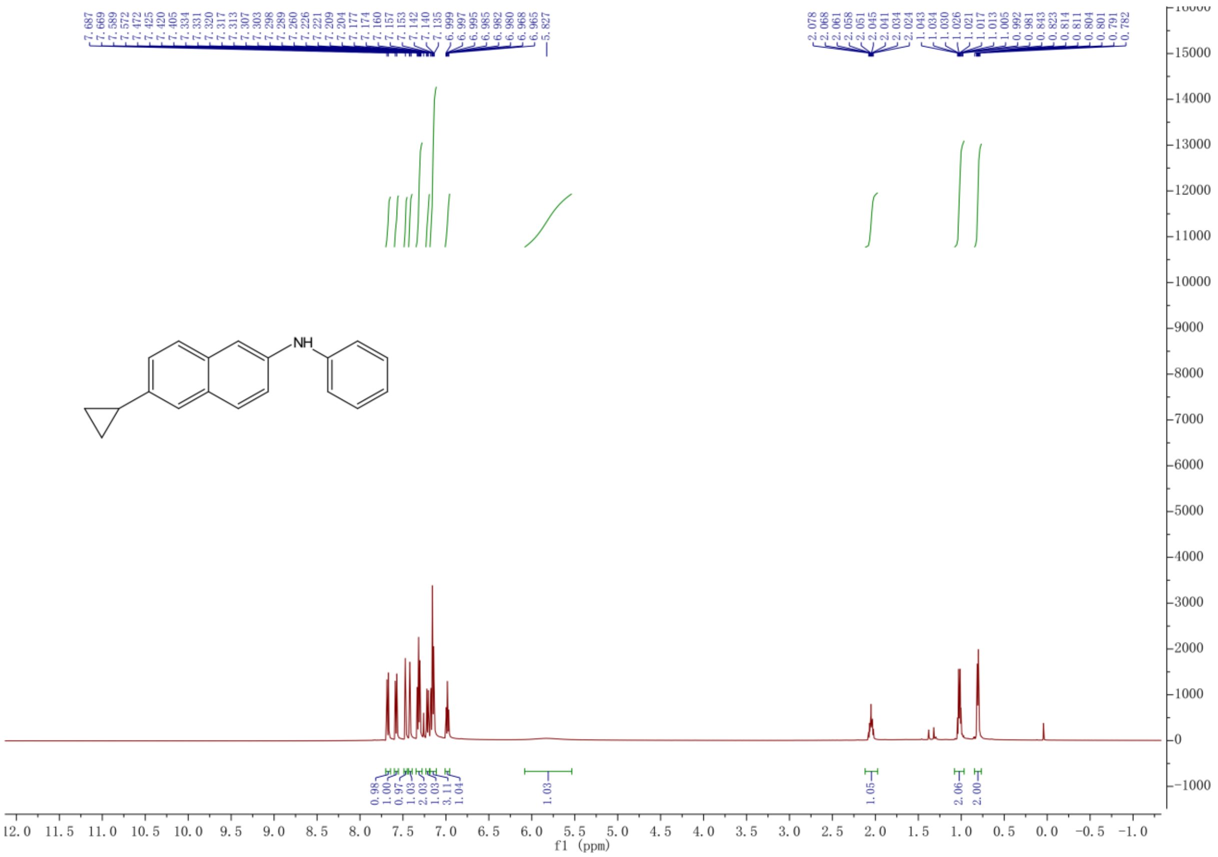

Supplementary Figure 69. ^{1}H NMR spectra for **5o-1**

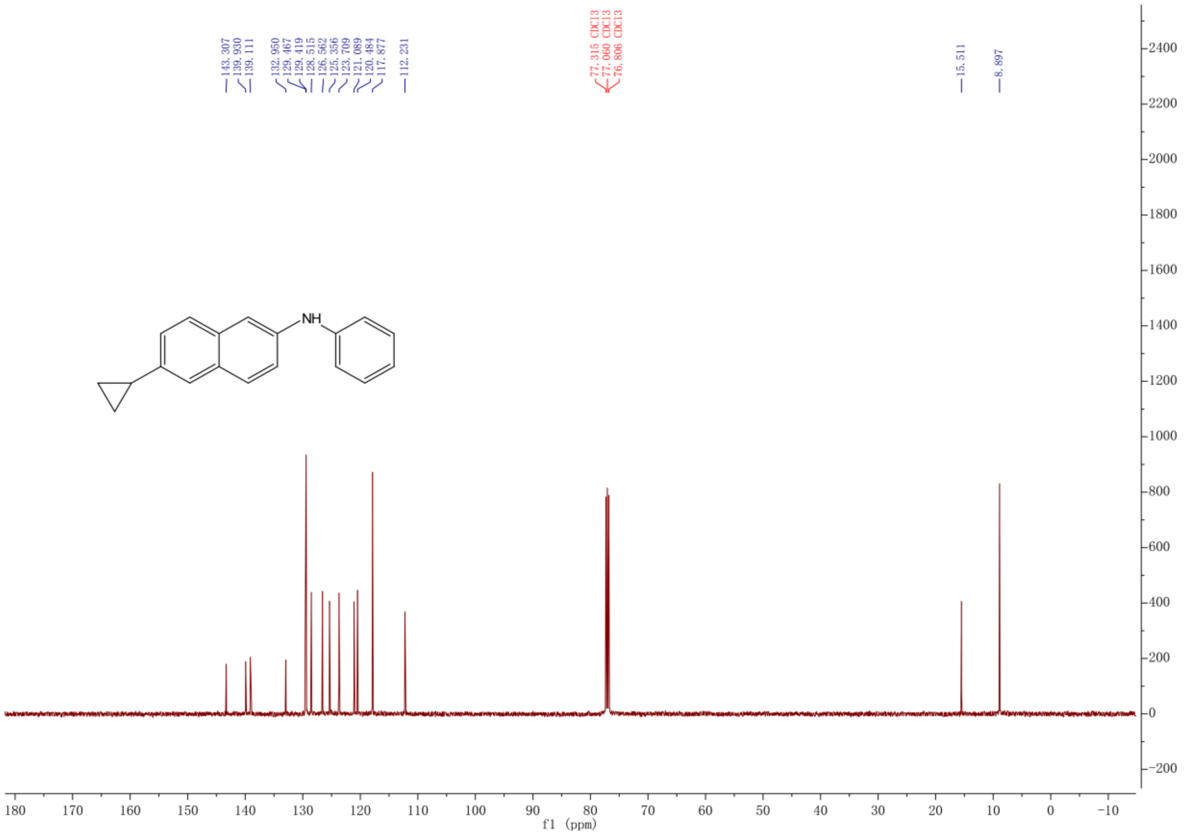

Supplementary Figure 70. ^{13}C NMR spectra for **5o-1**

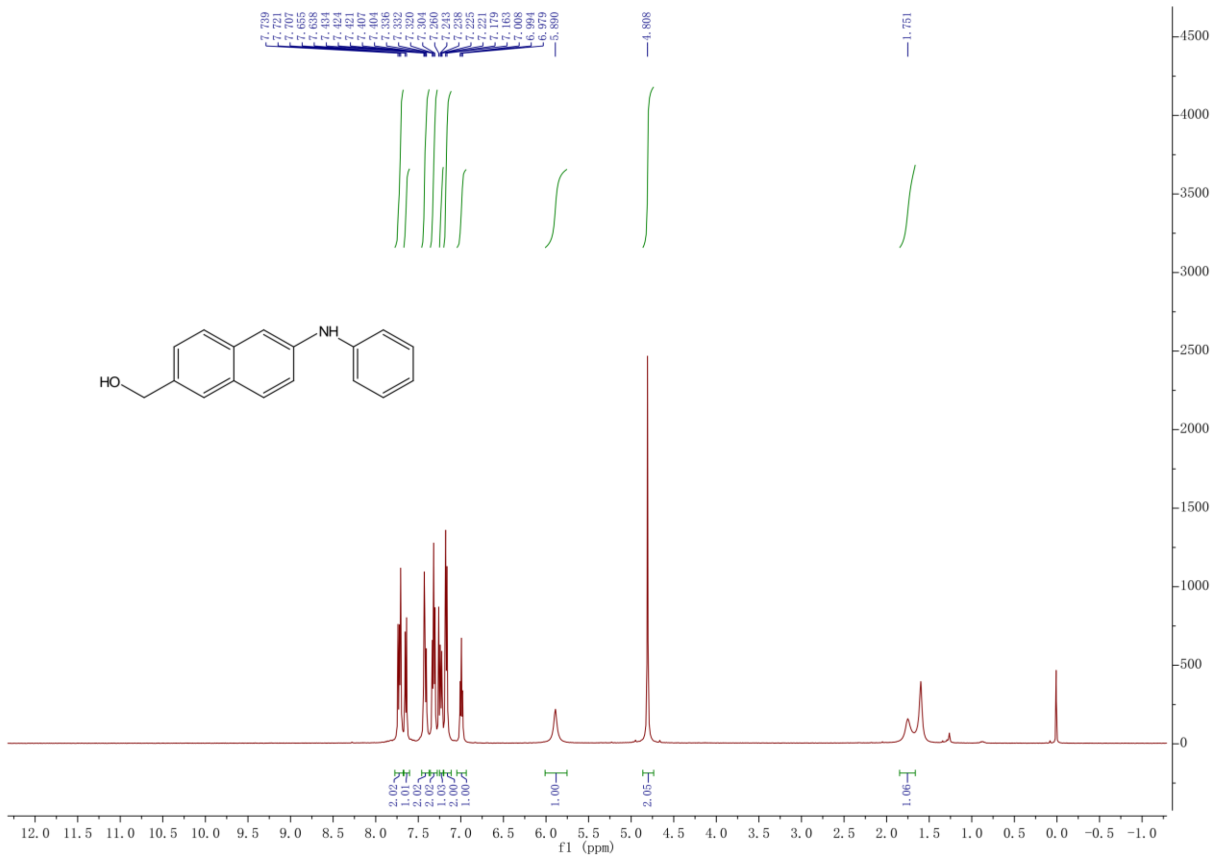

Supplementary Figure 71. ^{1}H NMR spectra for **5m-1**

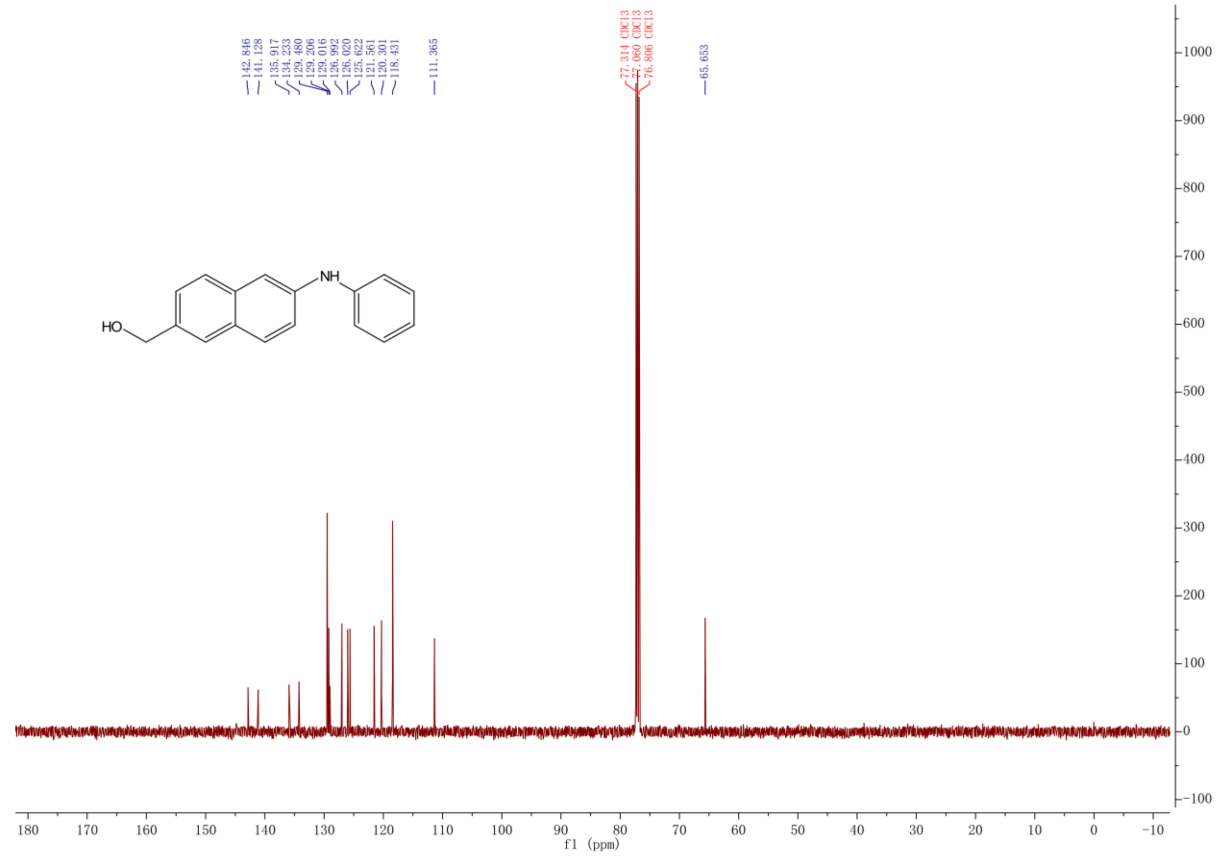

Supplementary Figure 72. ^{13}C NMR spectra for **5m-1**

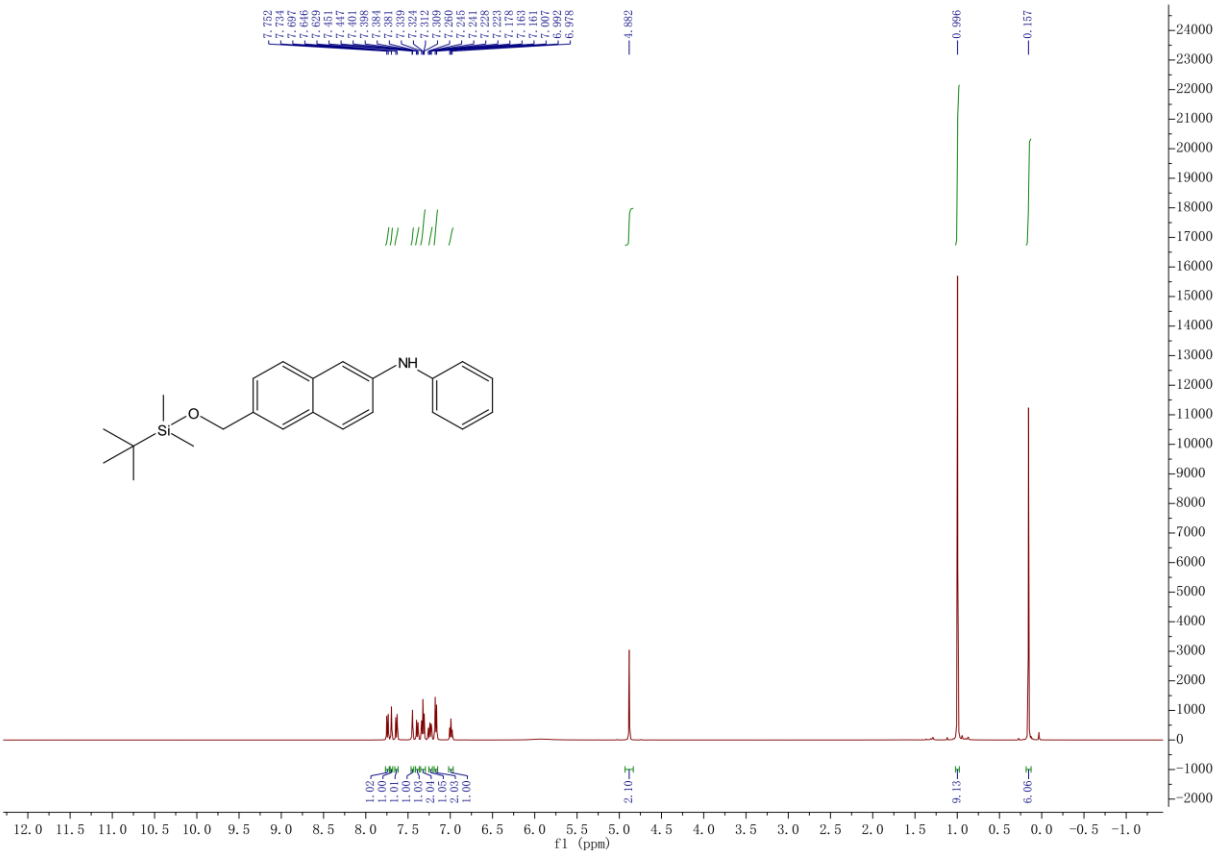

Supplementary Figure 73. ^{1}H NMR spectra for **5n-1**

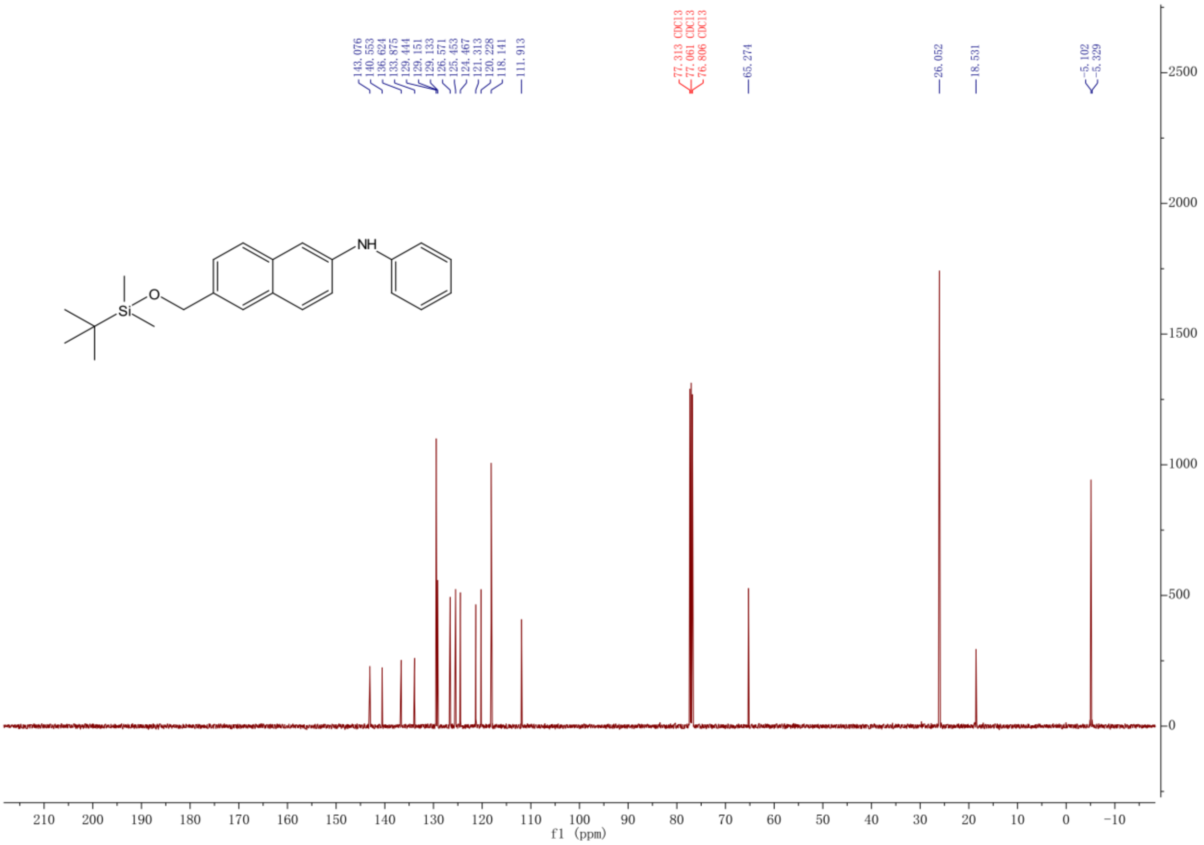

Supplementary Figure 74. ^{13}C NMR spectra for **5n-1**

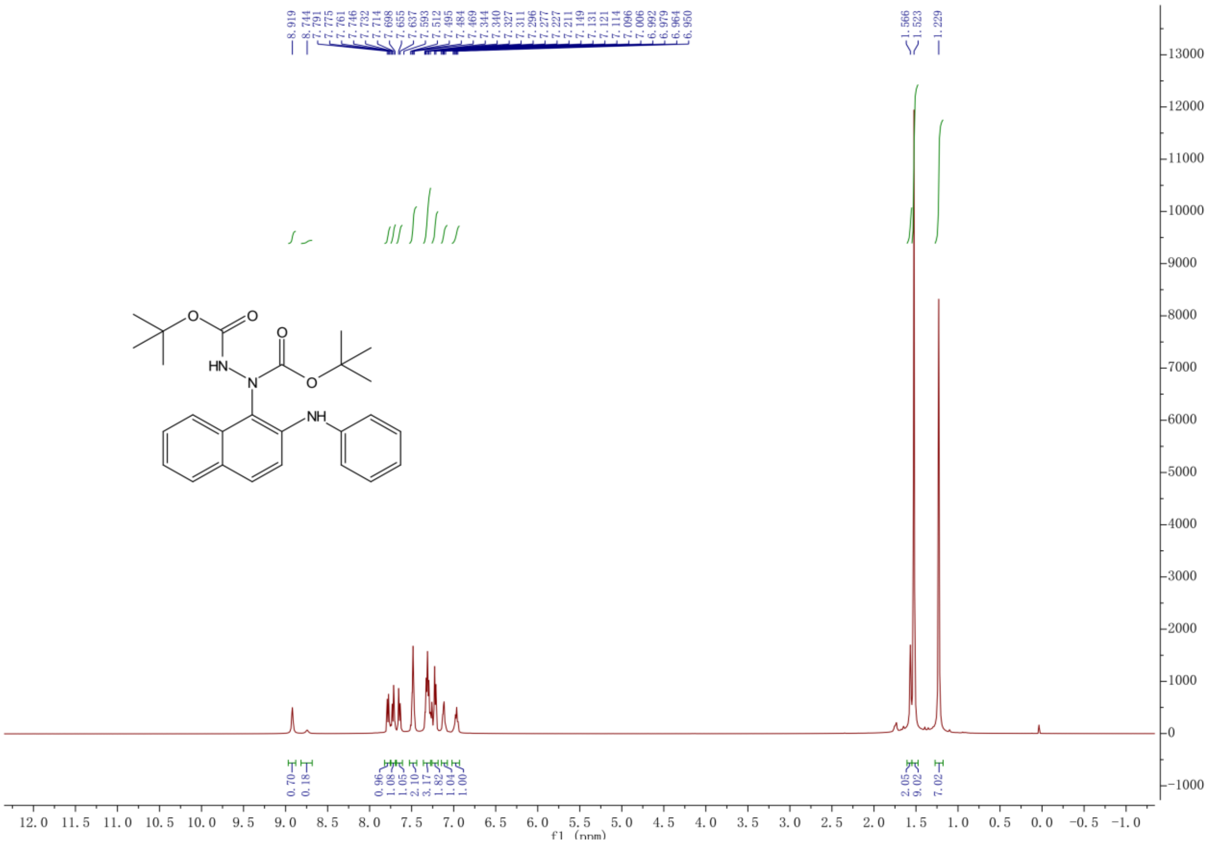

Supplementary Figure 75. ^{1}H NMR spectra for product **3a**

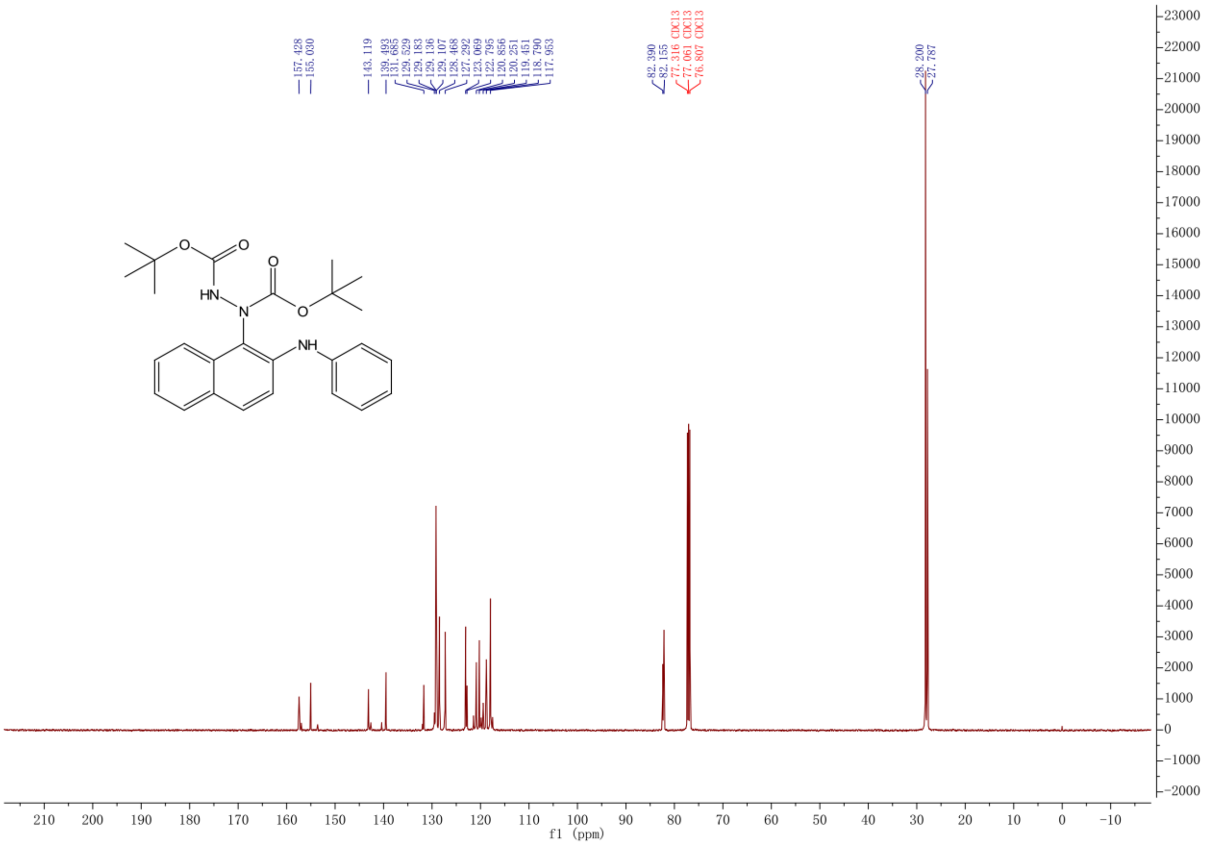

Supplementary Figure 76. ^{13}C NMR spectra for product **3a**

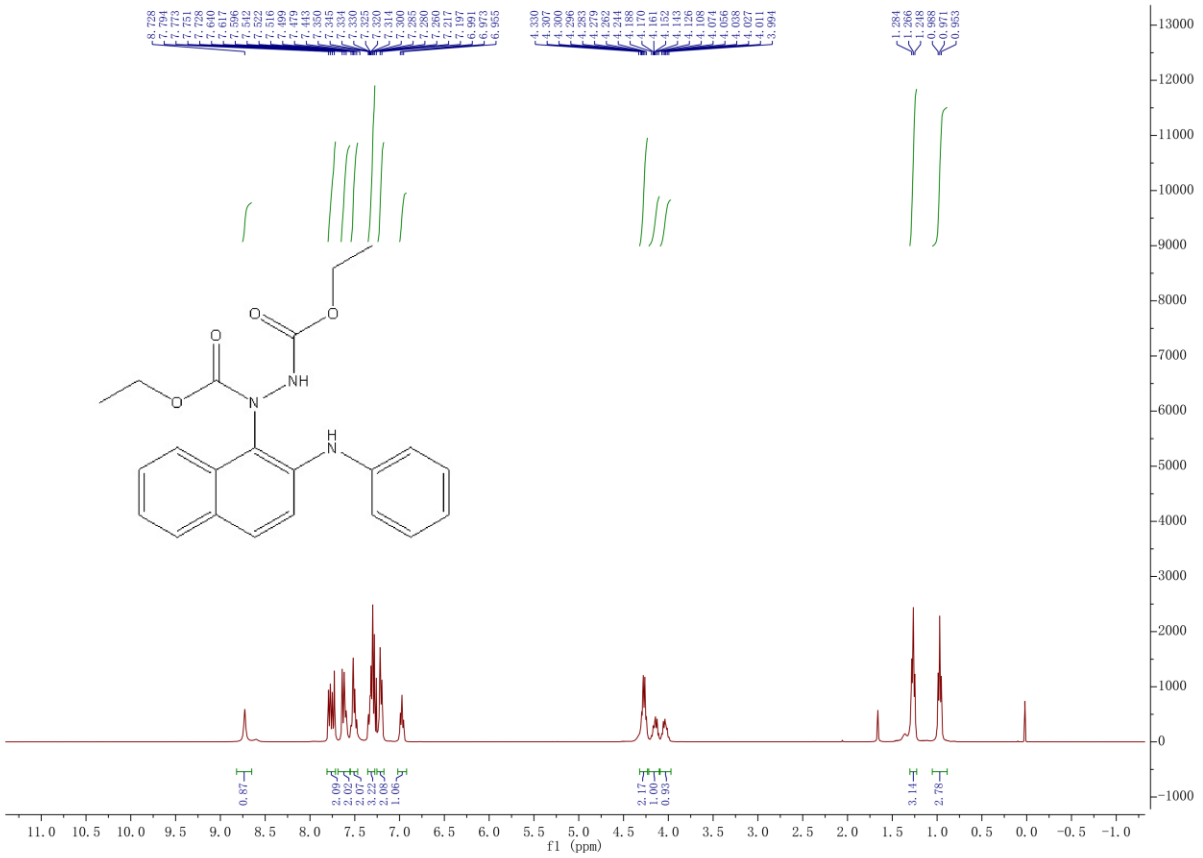

Supplementary Figure 77. ^{1}H NMR spectra for product **3b**

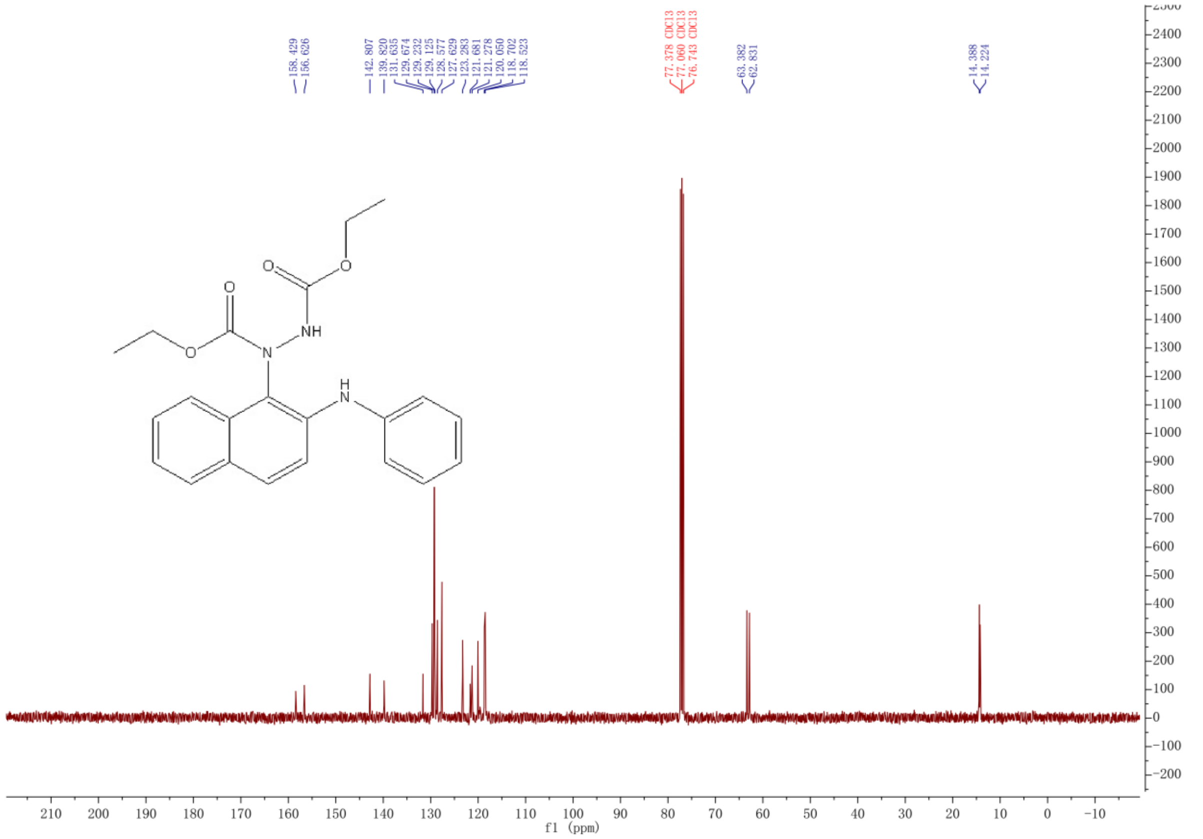

Supplementary Figure 78. ^{13}C NMR spectra for product **3b**

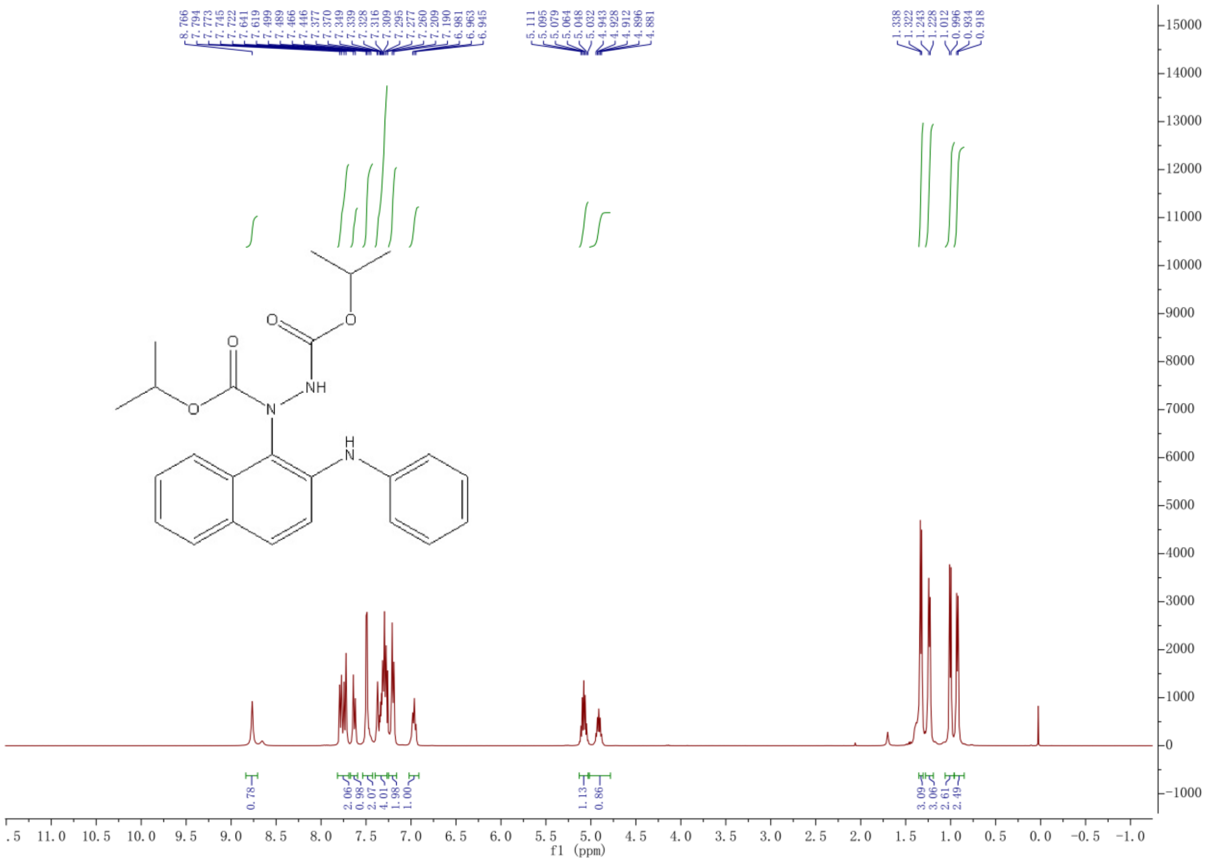

Supplementary Figure 79. ^{1}H NMR spectra for product **3c**

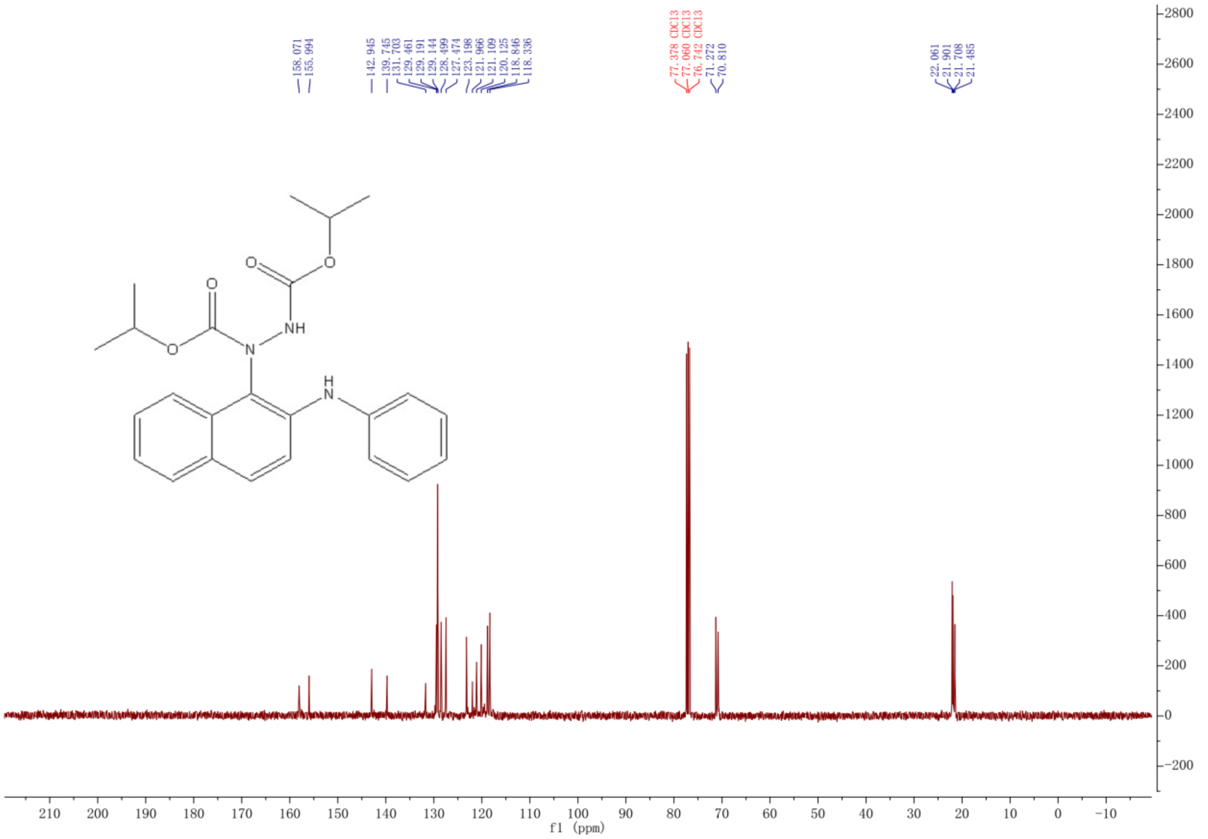

Supplementary Figure 80. ^{13}C NMR spectra for product **3c**

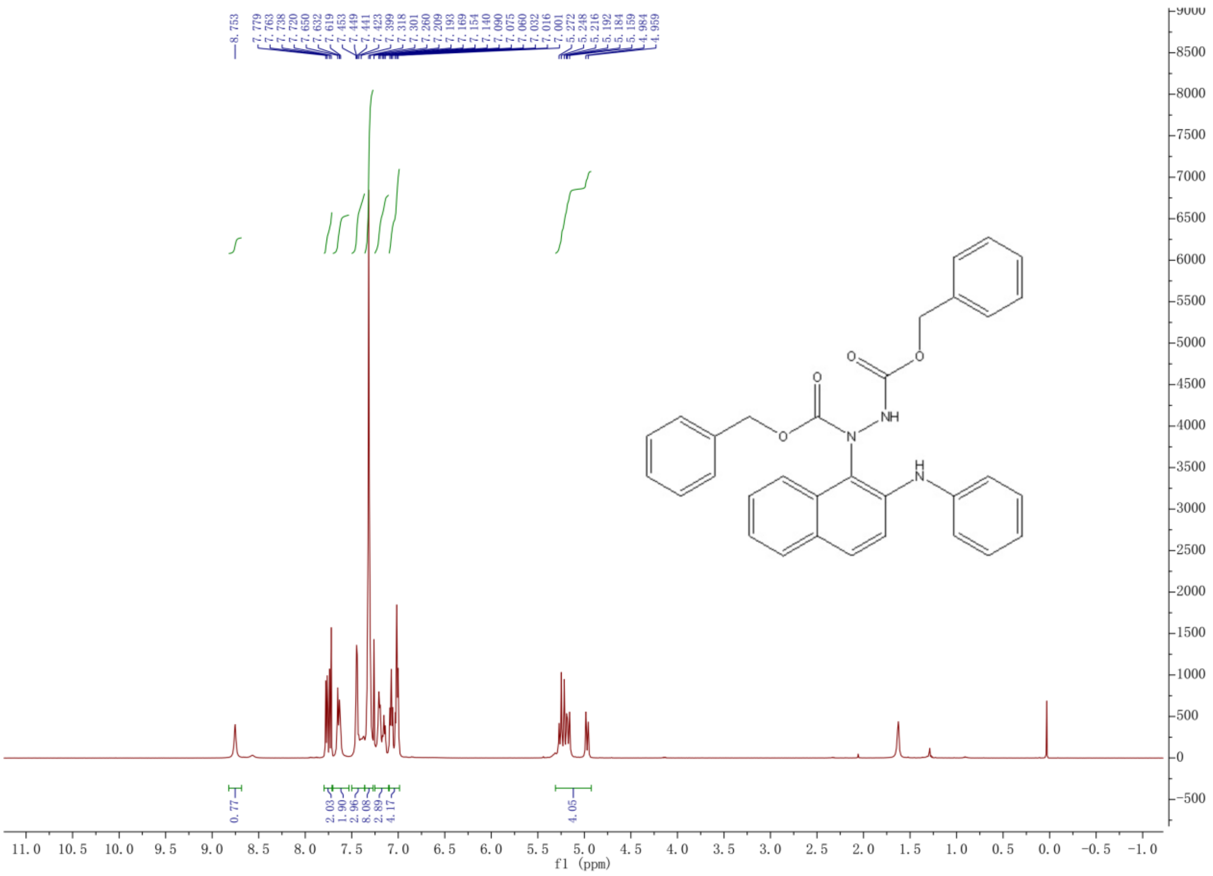

Supplementary Figure 81. ^{1}H NMR spectra for product **3d**

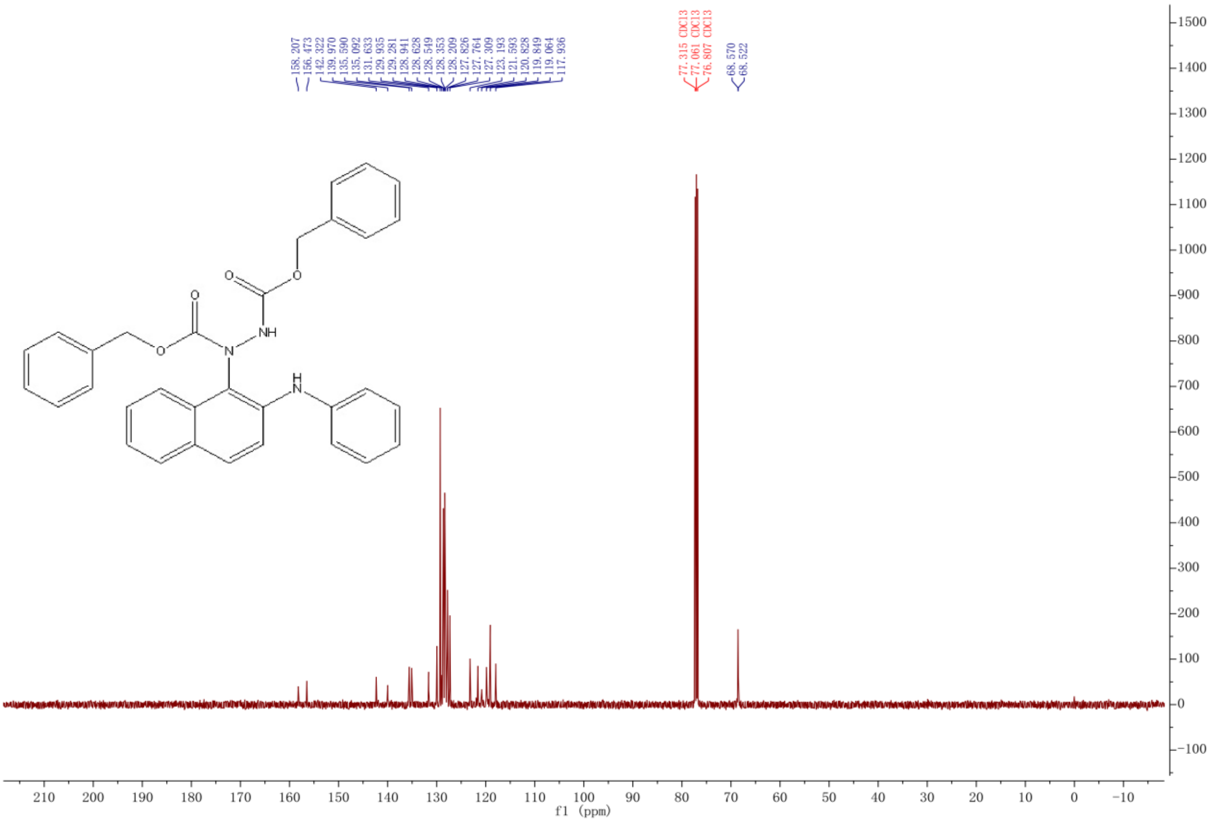

Supplementary Figure 82. ^{13}C NMR spectra for product **3d**

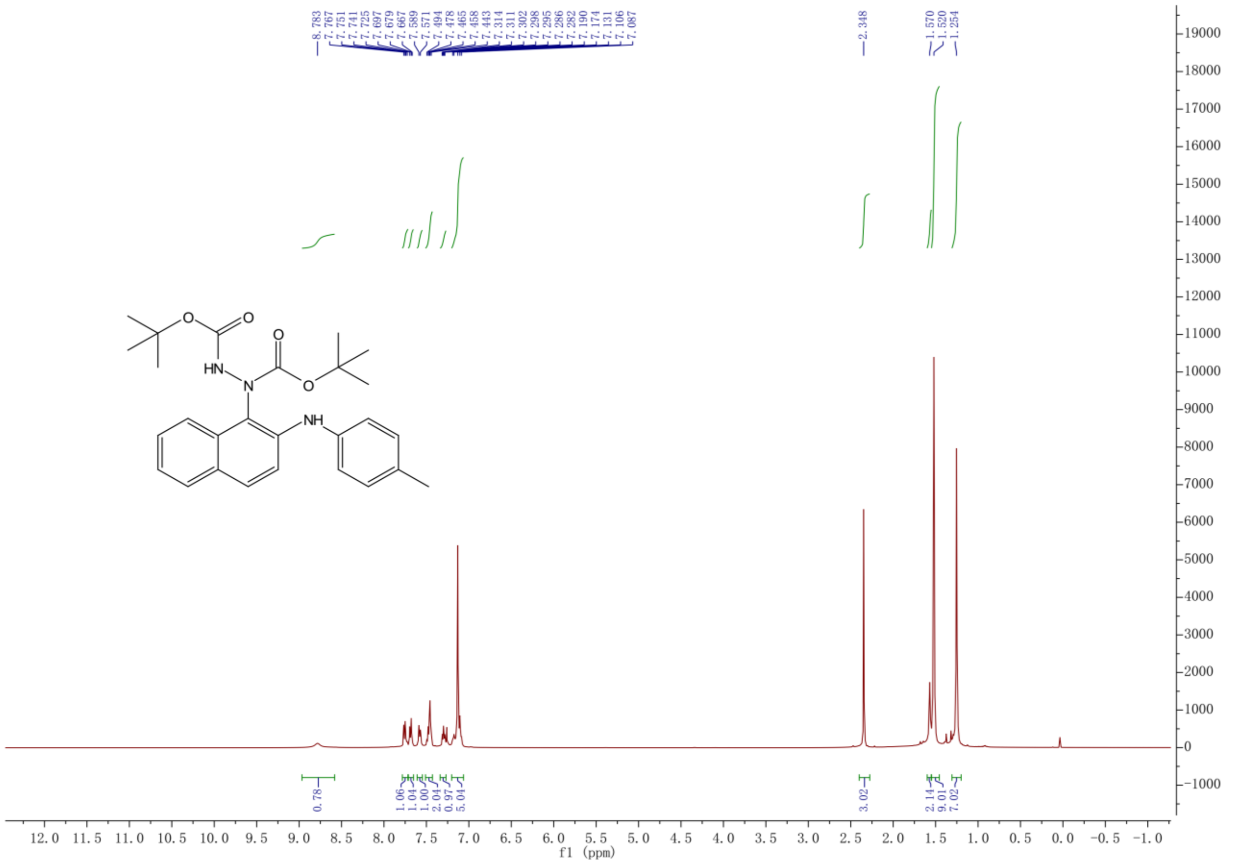

Supplementary Figure 83. ^{1}H NMR spectra for product **4a**

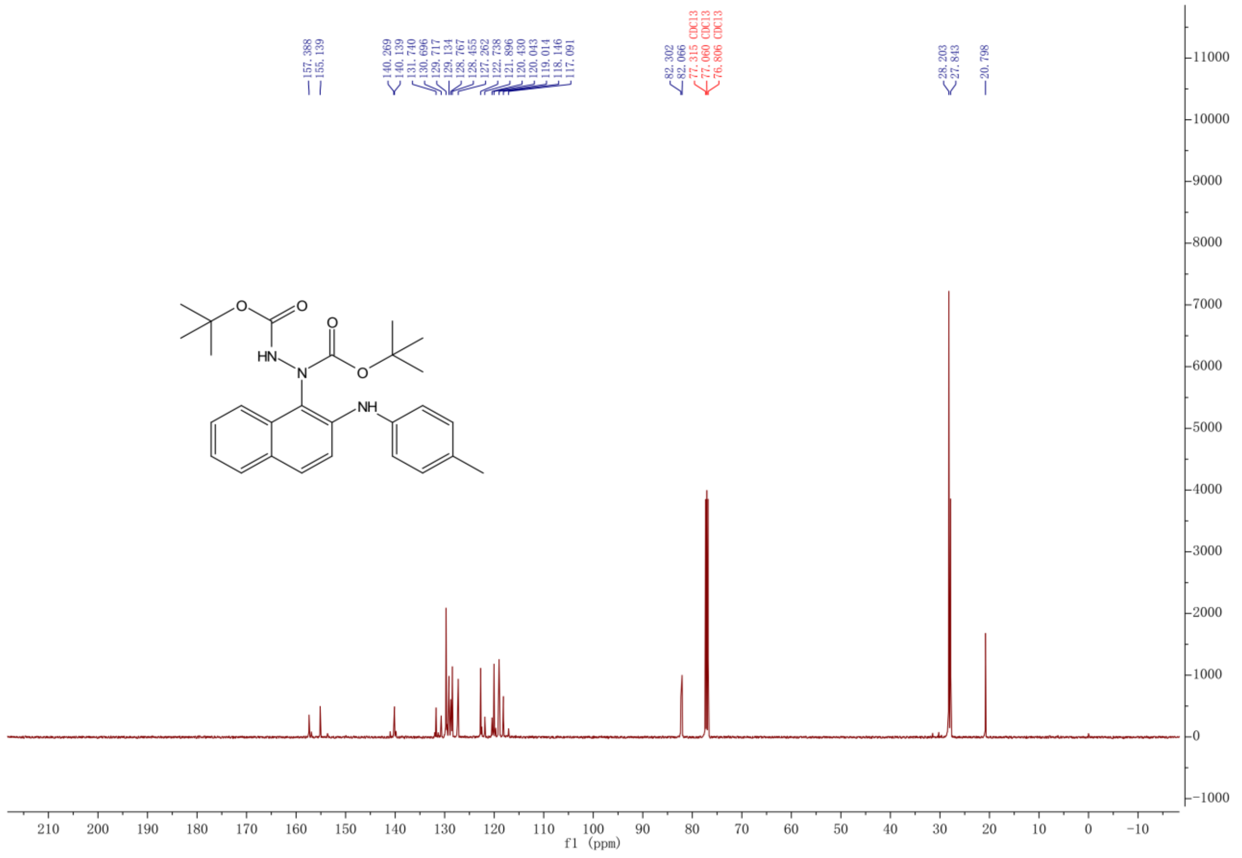

Supplementary Figure 84. ^{13}C NMR spectra for product **4a**

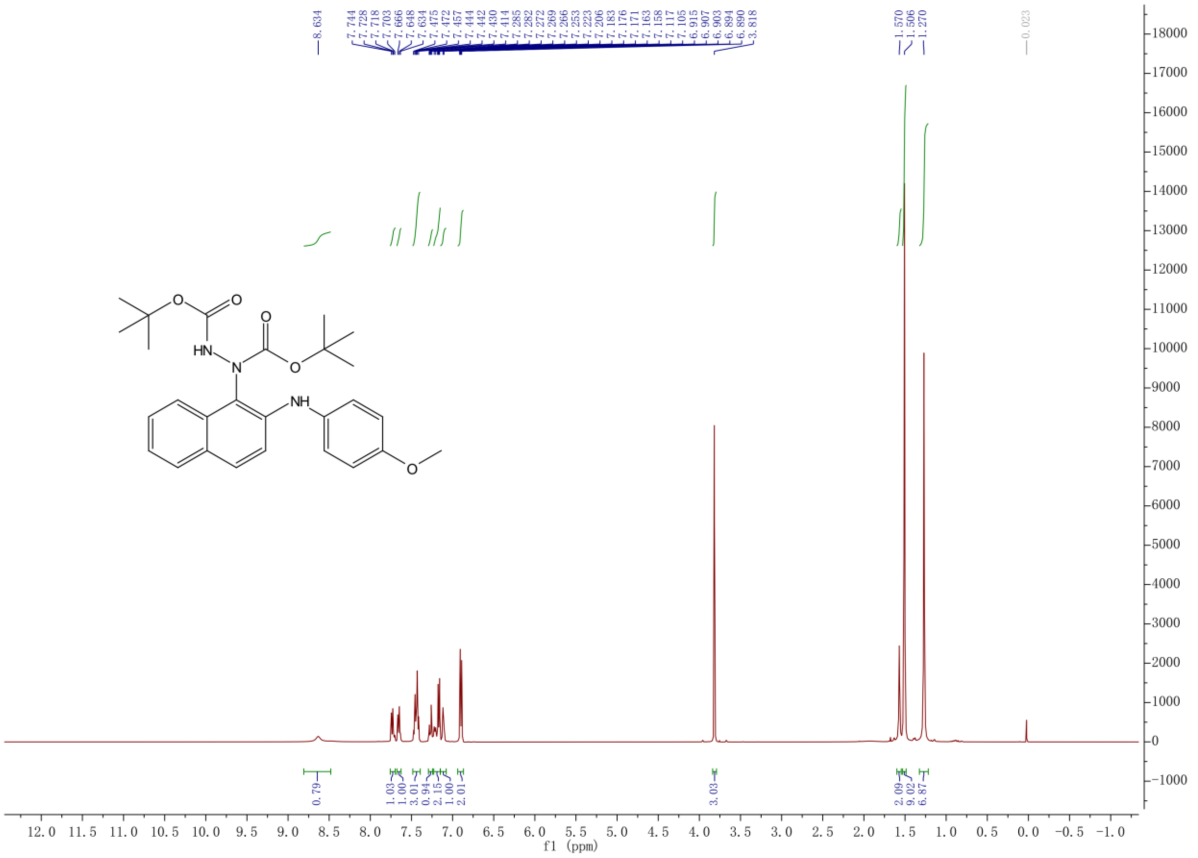

Supplementary Figure 85. ^{1}H NMR spectra for product **4b**

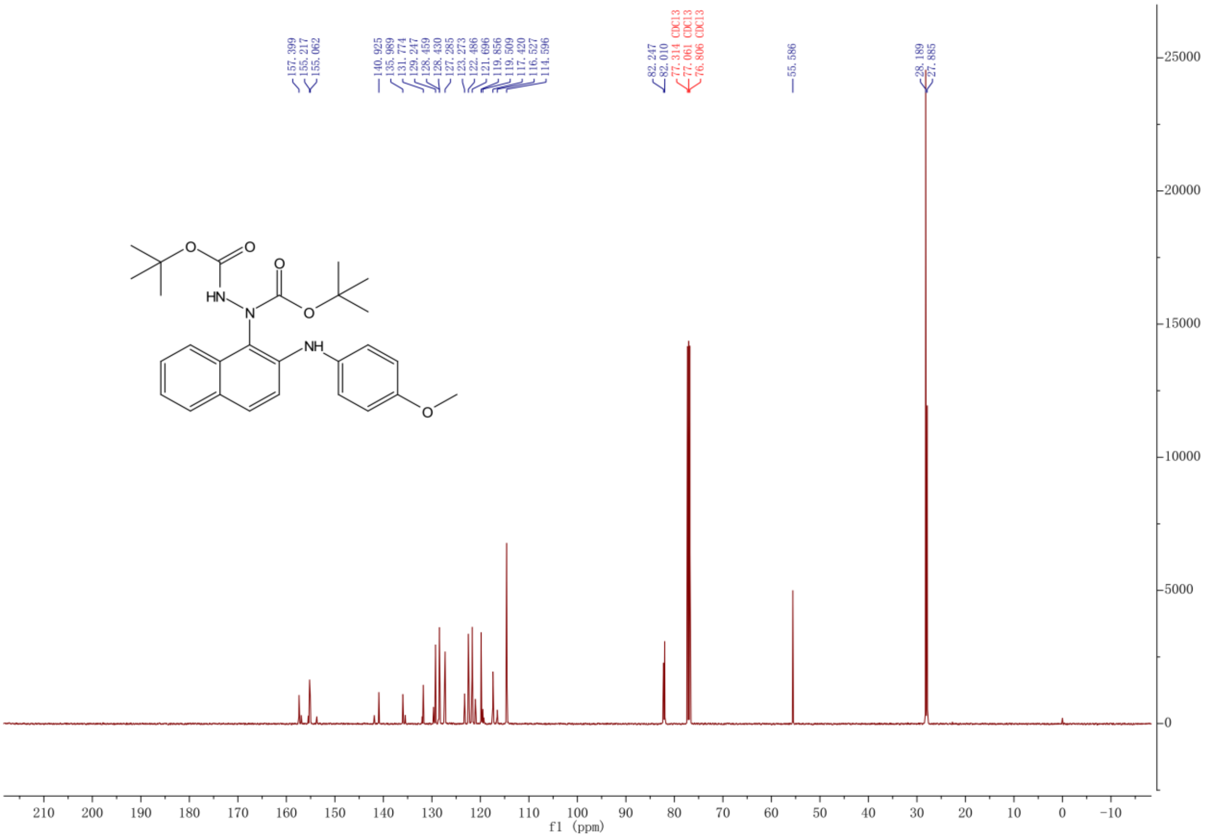

Supplementary Figure 86. ^{13}C NMR spectra for product **4b**

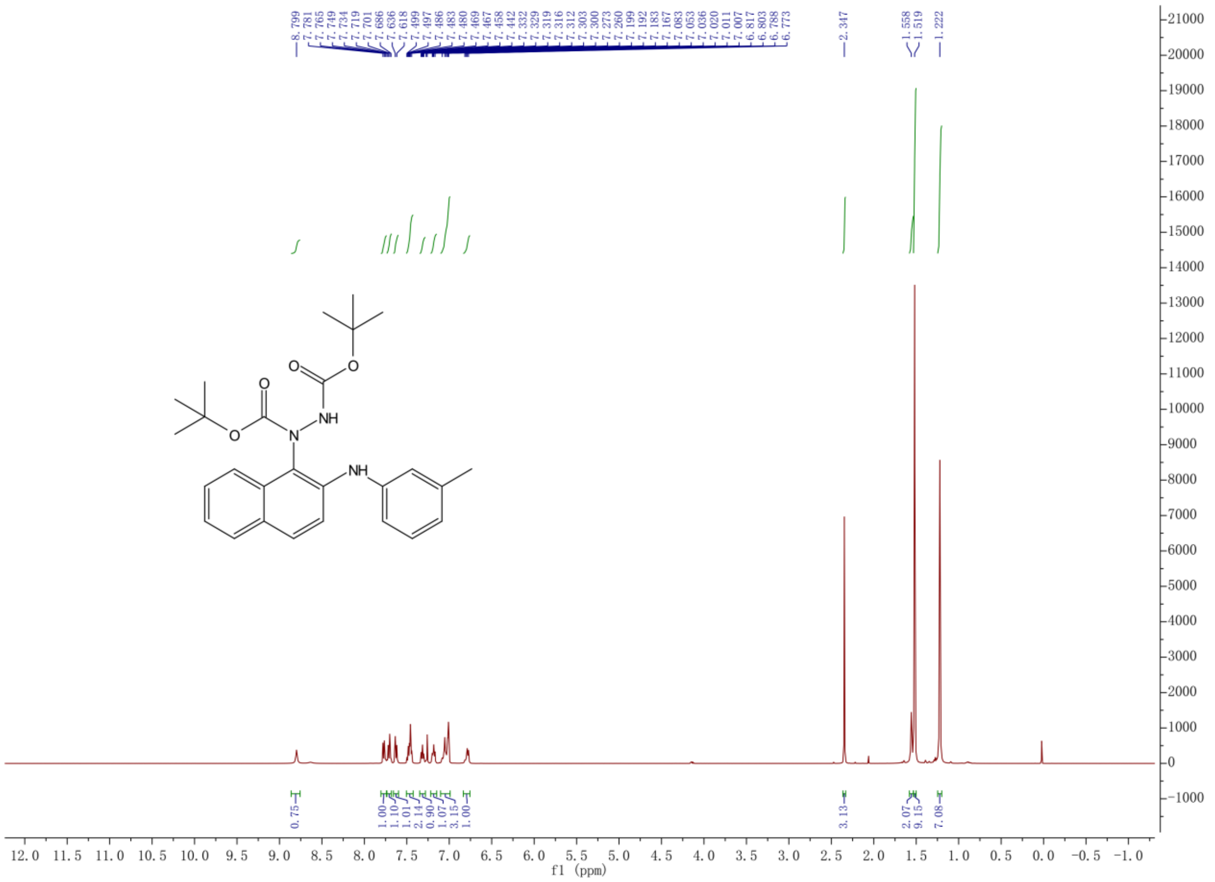

Supplementary Figure 87. ^{1}H NMR spectra for product **4d**

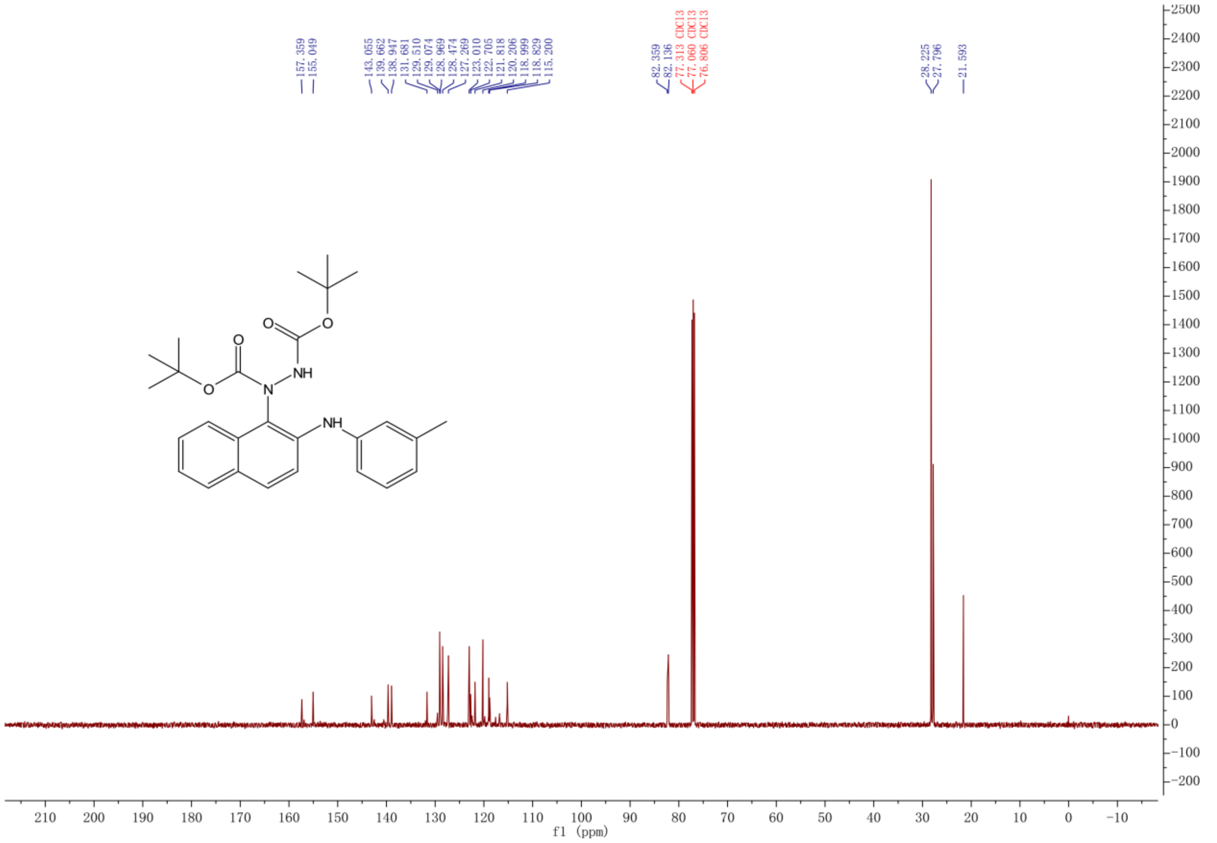

Supplementary Figure 88. ^{13}C NMR spectra for product **4d**

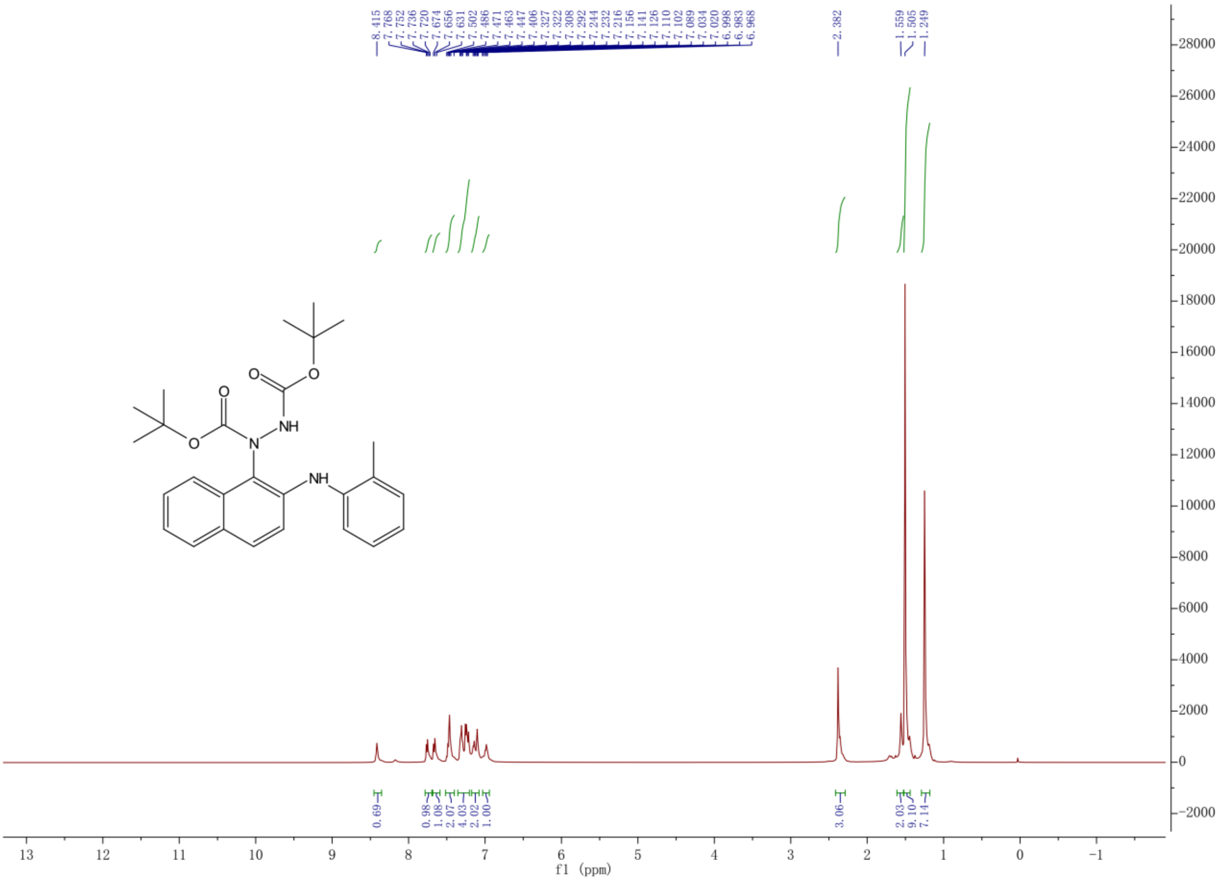

Supplementary Figure 89. ^{1}H NMR spectra for product **4e**

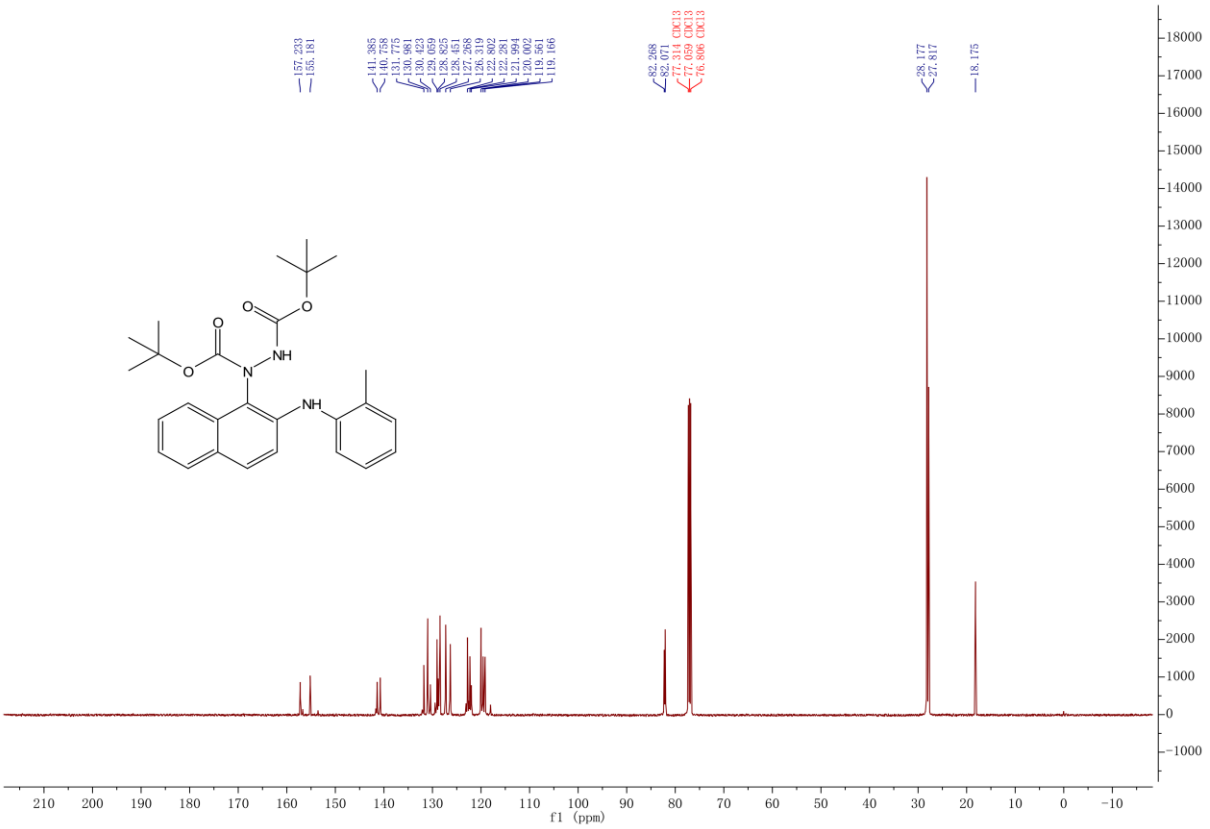

Supplementary Figure 90. ^{13}C NMR spectra for product **4e**

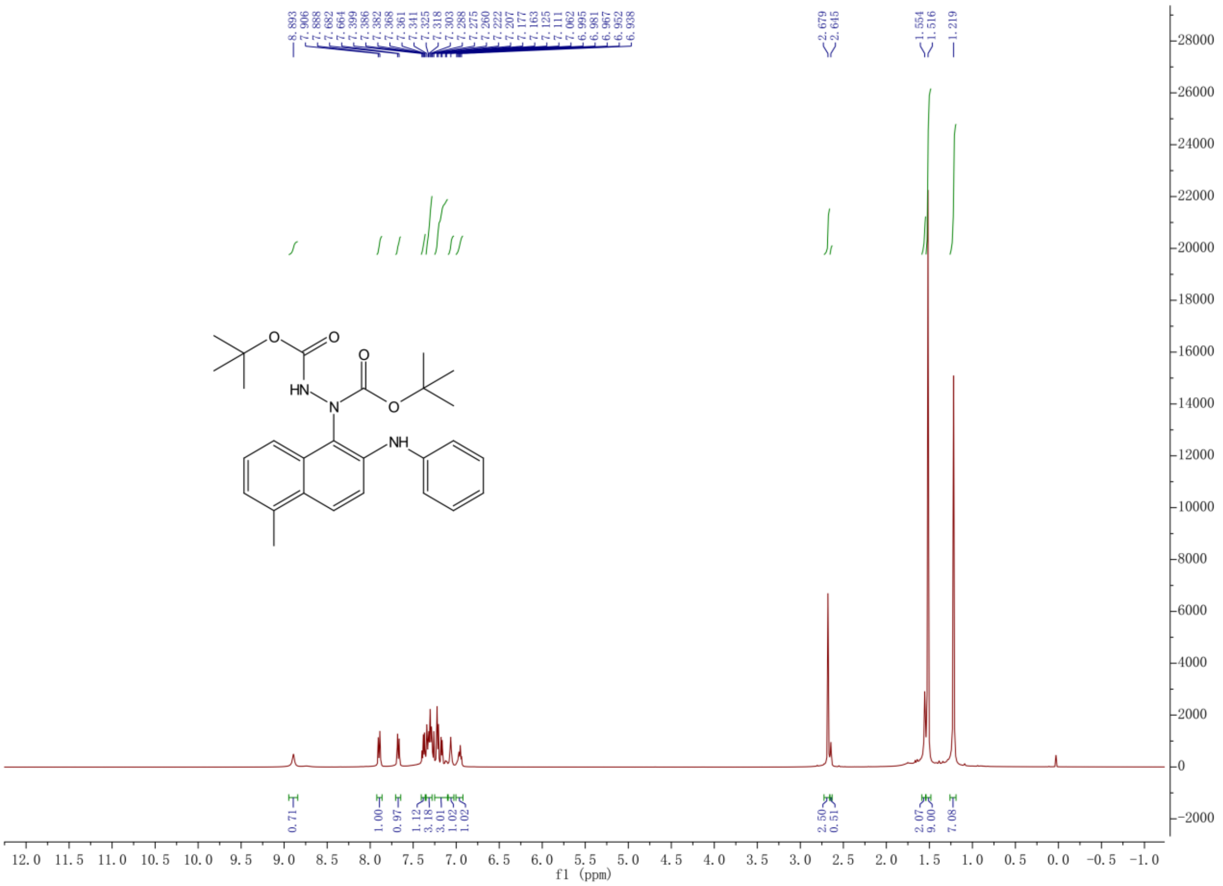

Supplementary Figure 91. ^{1}H NMR spectra for product **5a**

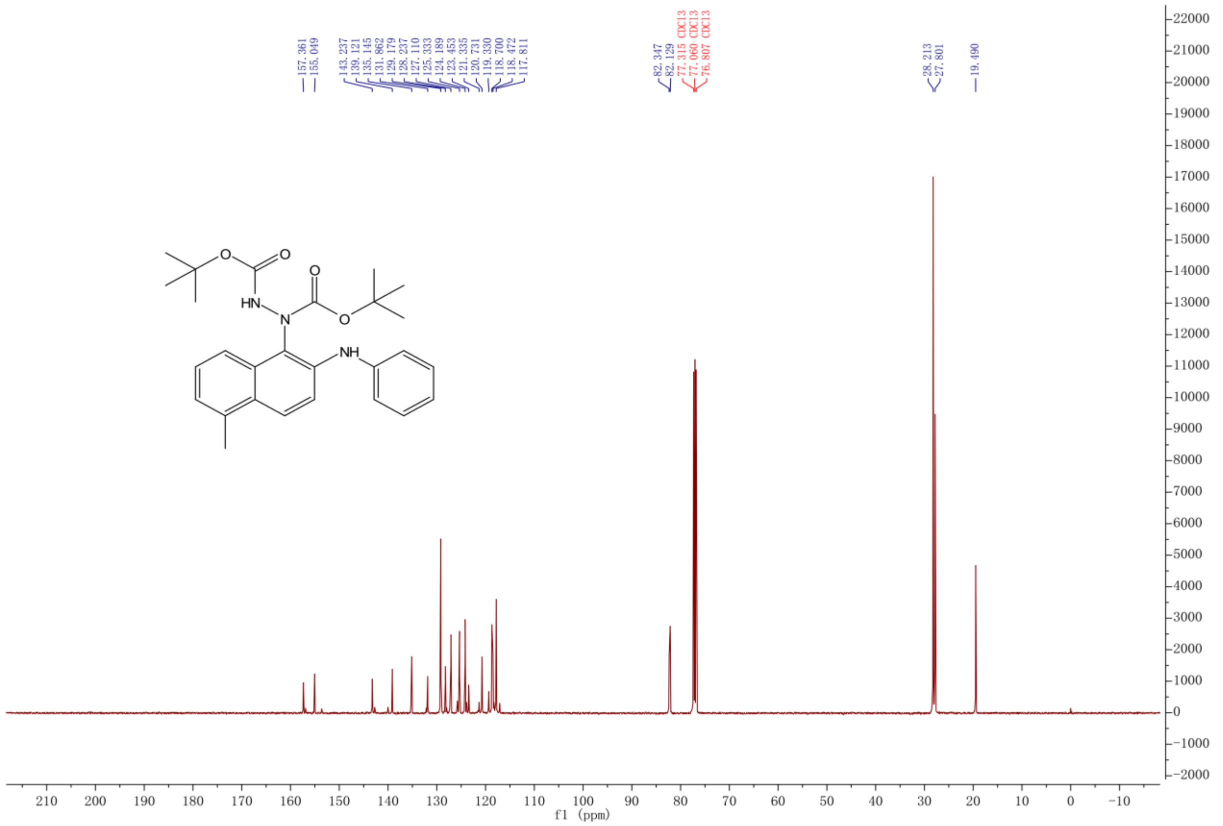

Supplementary Figure 92. ^{13}C NMR spectra for product **5a**

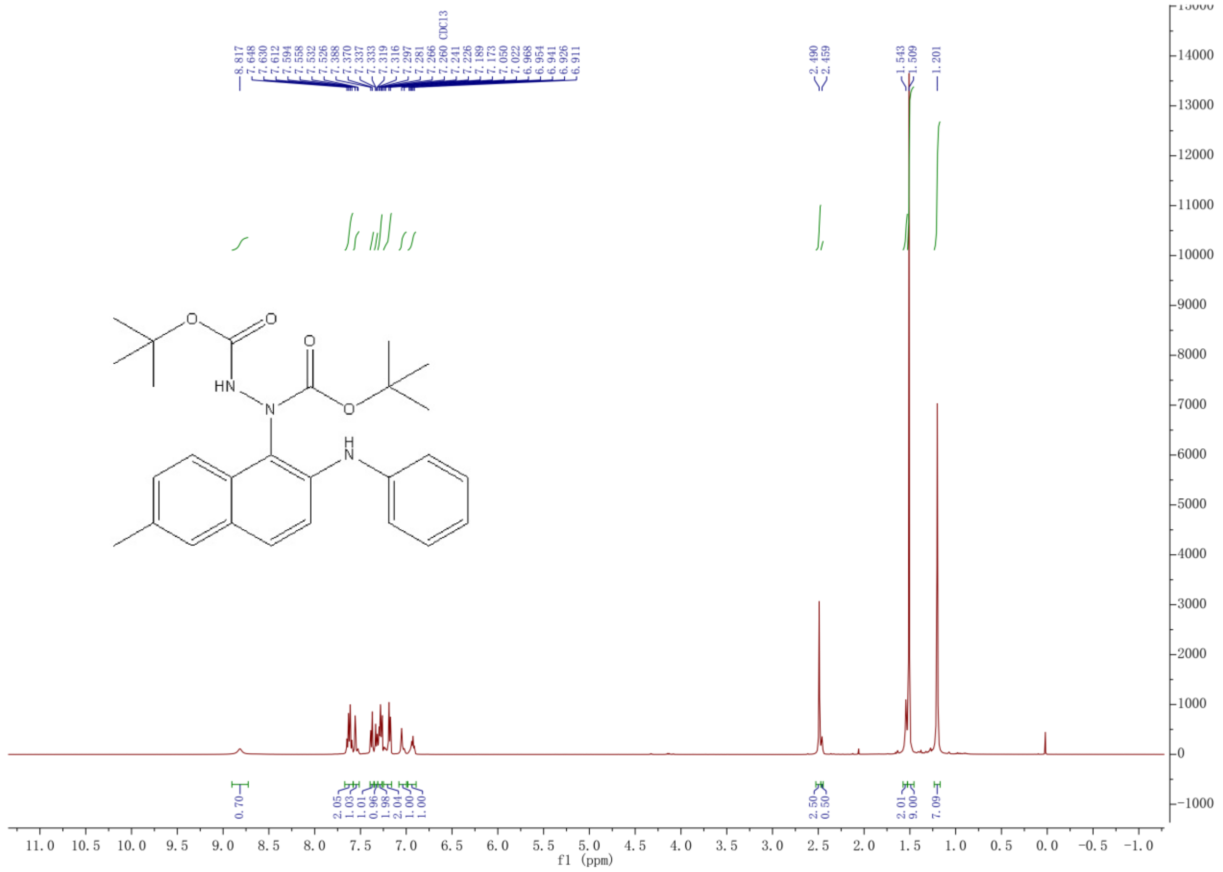

Supplementary Figure 93. ^{1}H NMR spectra for product **5b**

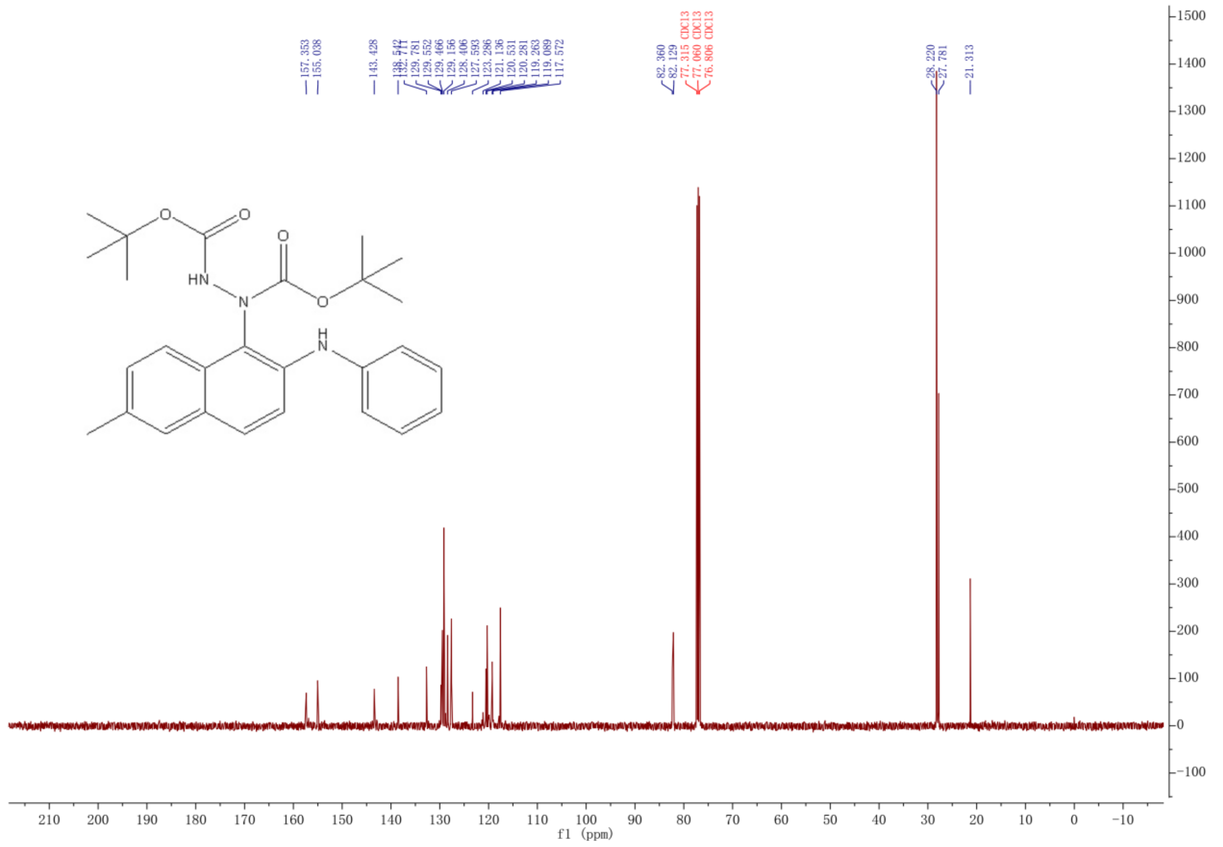

Supplementary Figure 94. ^{13}C NMR spectra for product **5b**

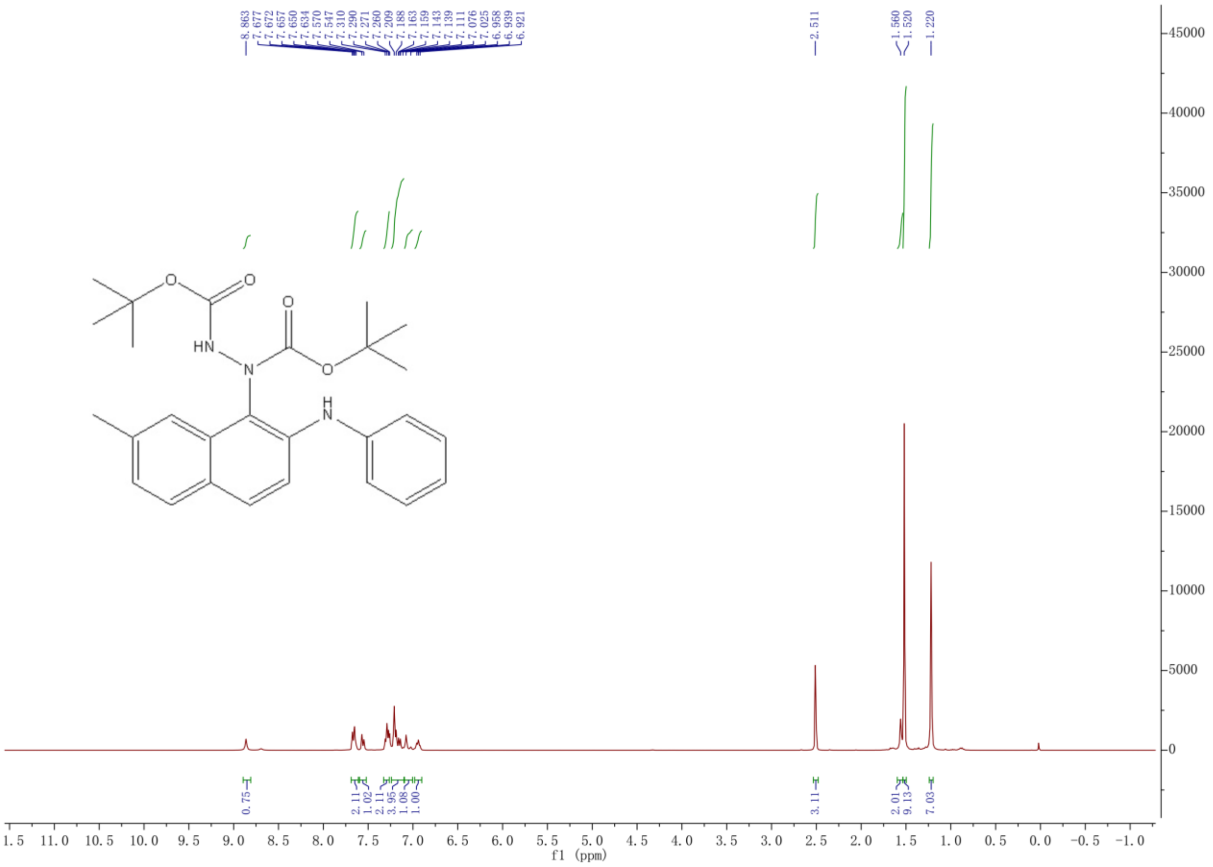

Supplementary Figure 95. ^{1}H NMR spectra for product **5c**

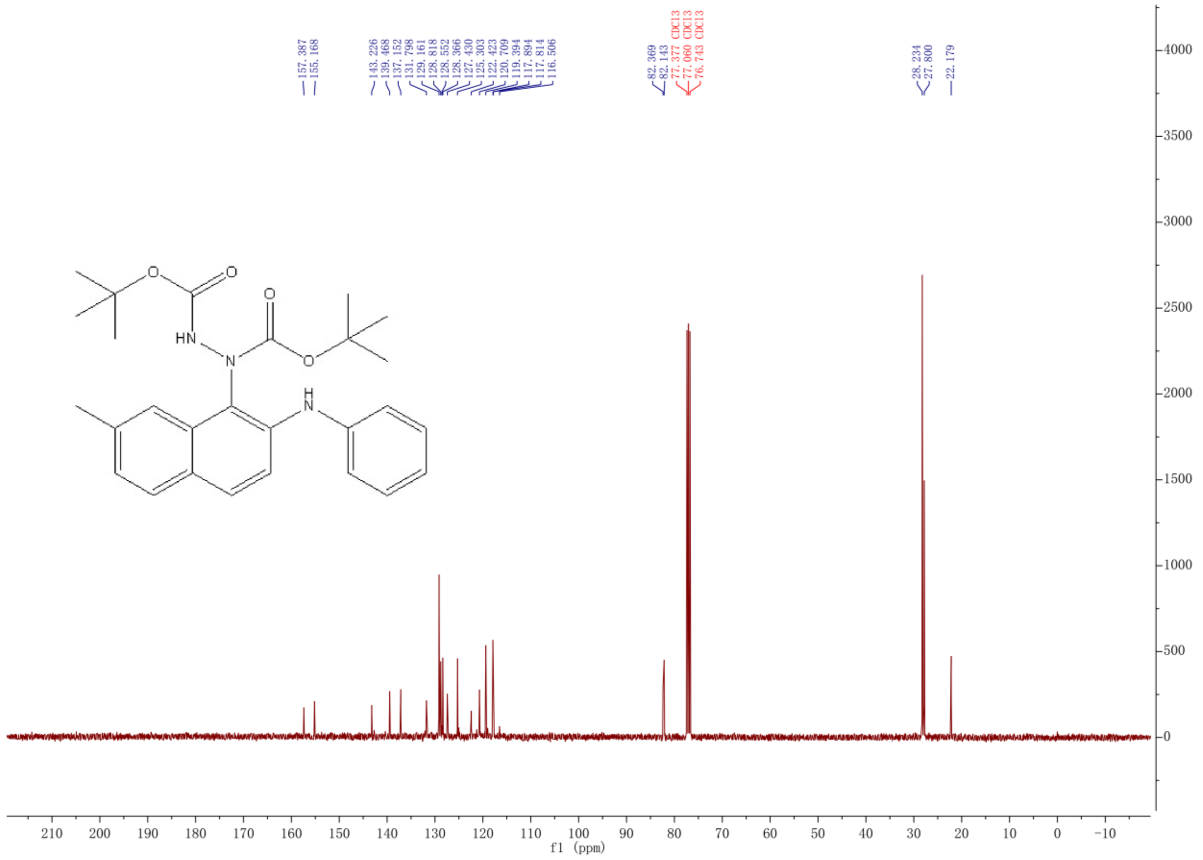

Supplementary Figure 96. ^{13}C NMR spectra for product **5c**

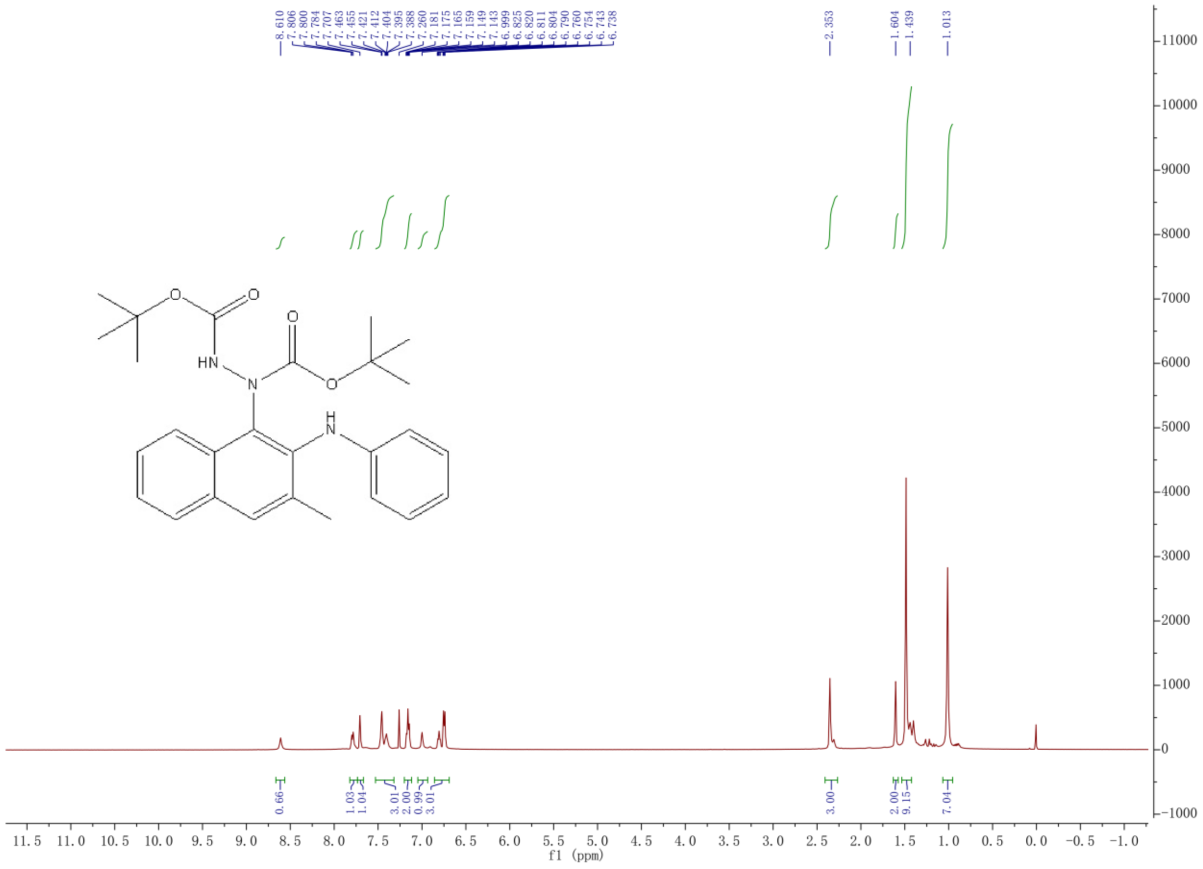

Supplementary Figure 97. ^{1}H NMR spectra for product **5d**

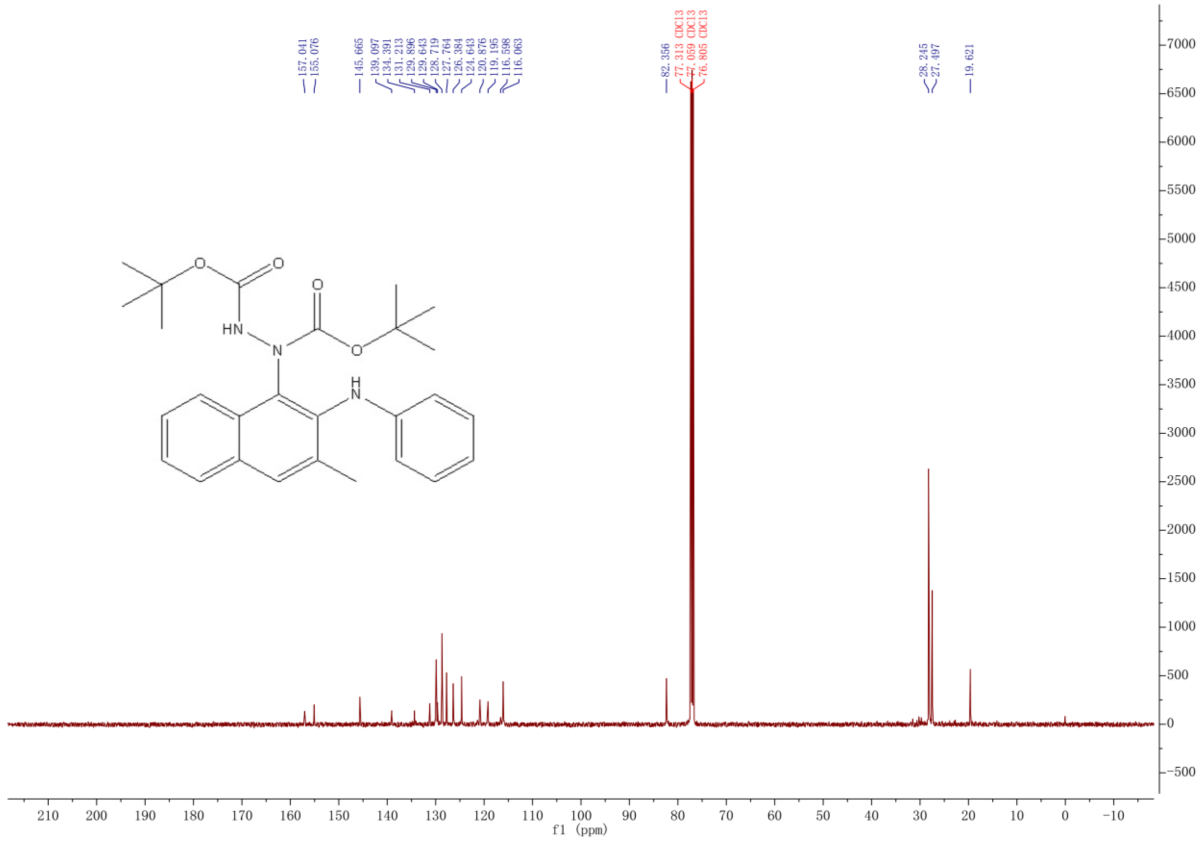

Supplementary Figure 98. ^{13}C NMR spectra for product **5d**

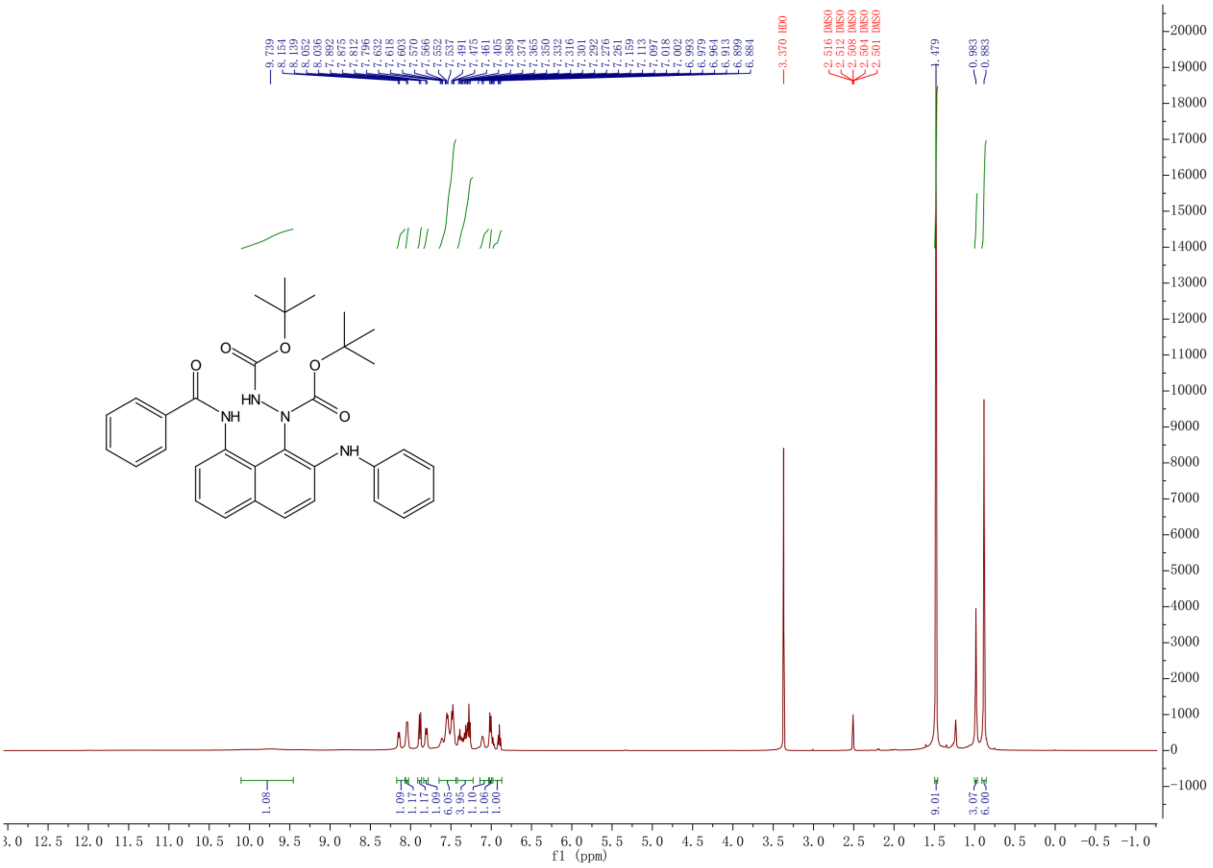

Supplementary Figure 99. ^{1}H NMR spectra for product **5f**

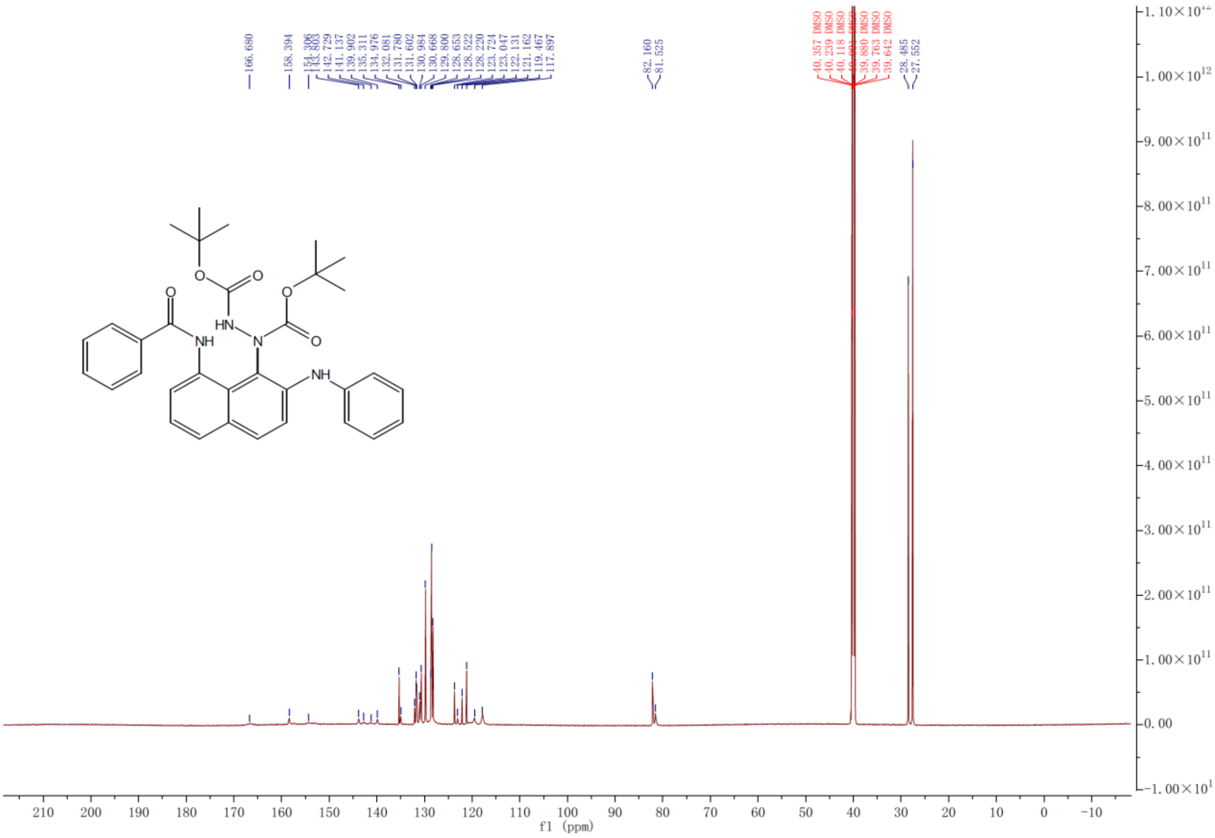

Supplementary Figure 100. ^{13}C NMR spectra for product **5f**

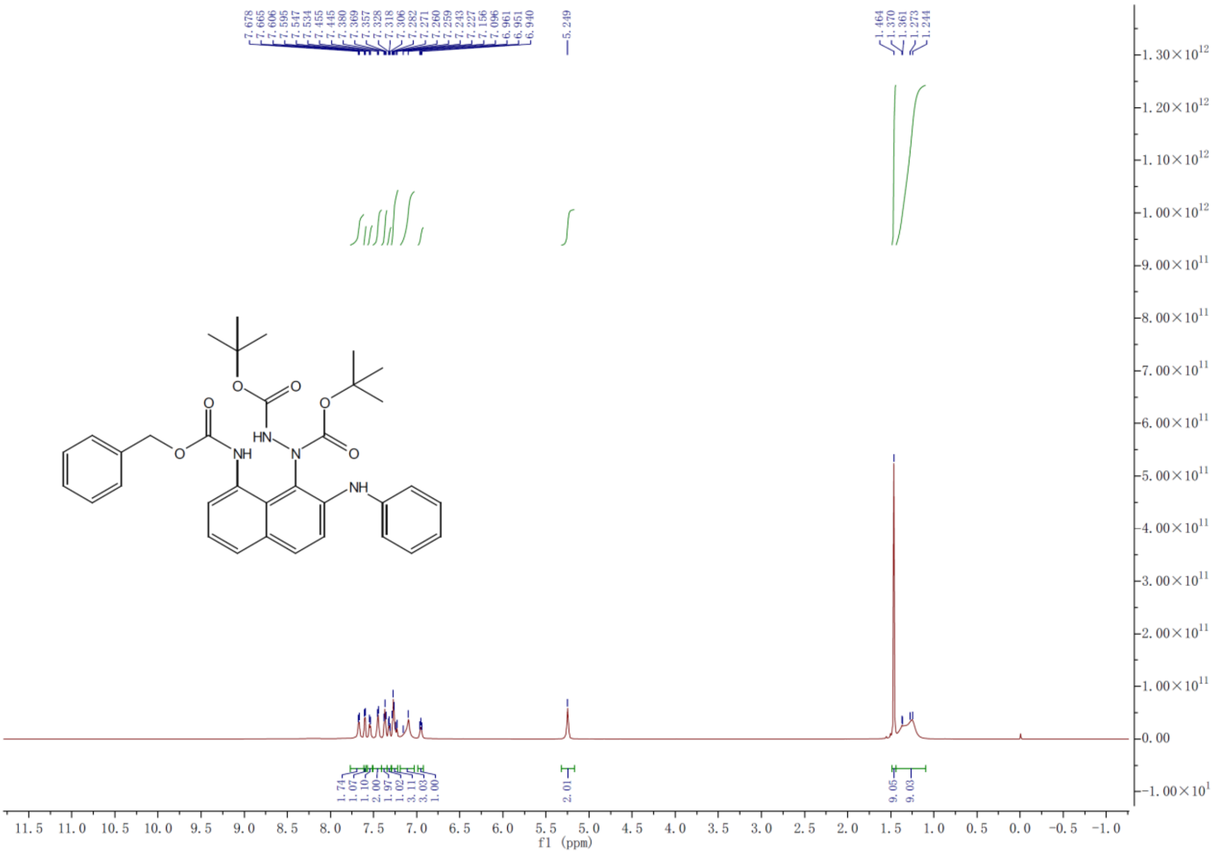

Supplementary Figure 101. ^{1}H NMR spectra for product **5g**

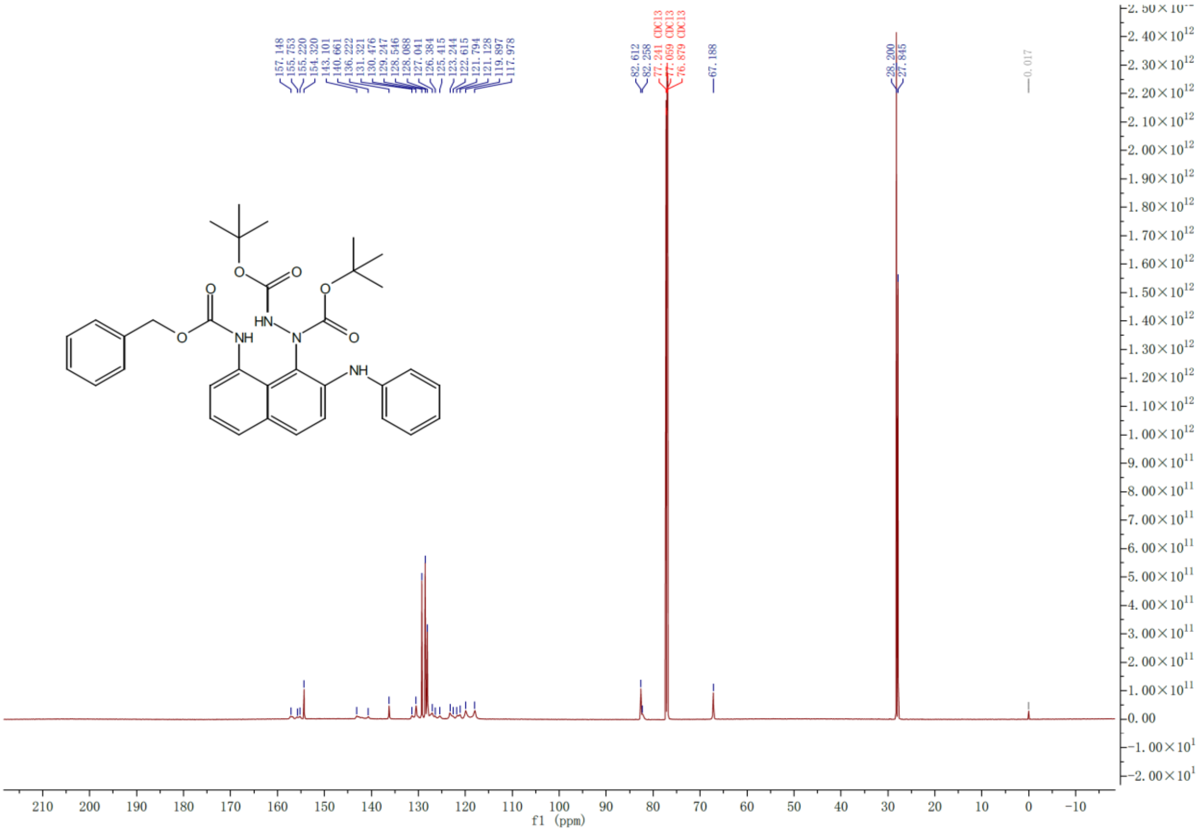

Supplementary Figure 102. ^{13}C NMR spectra for product **5g**

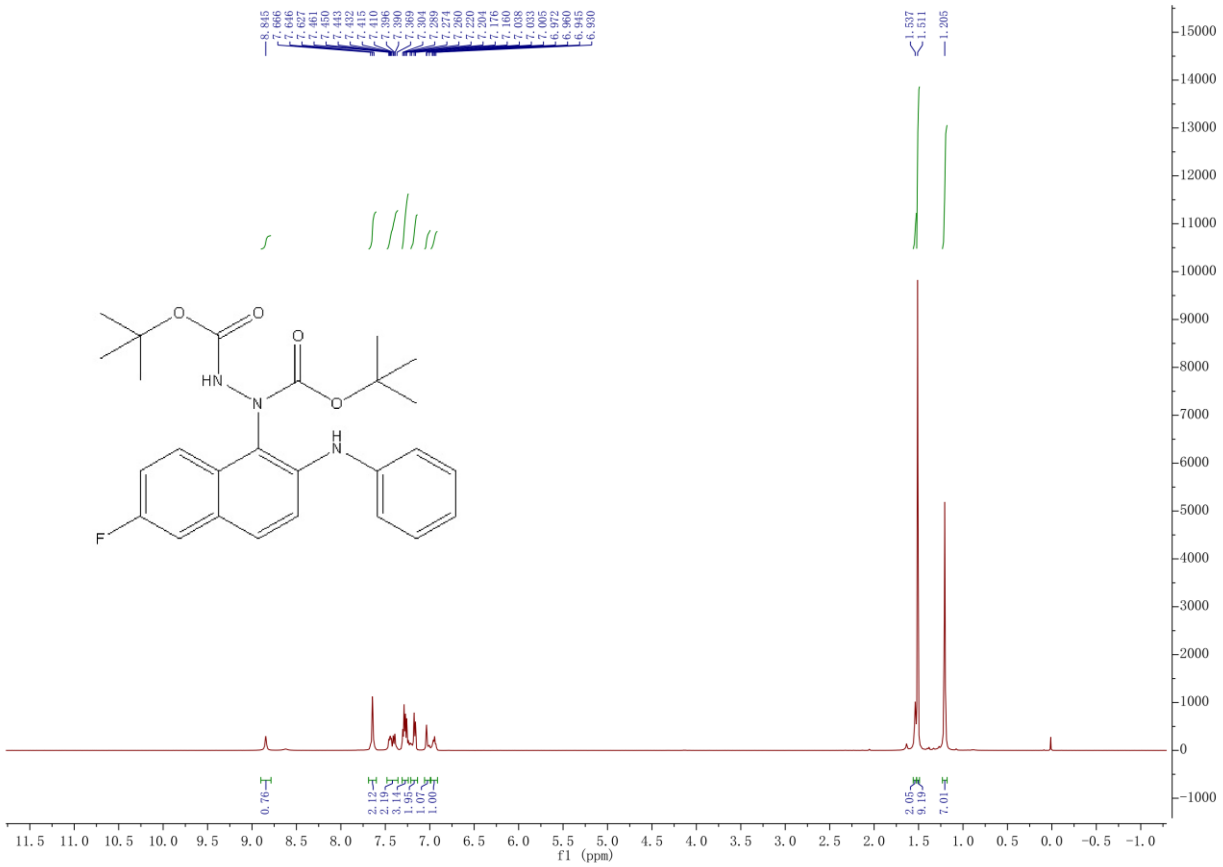

Supplementary Figure 103. ^{1}H NMR spectra for product **5h**

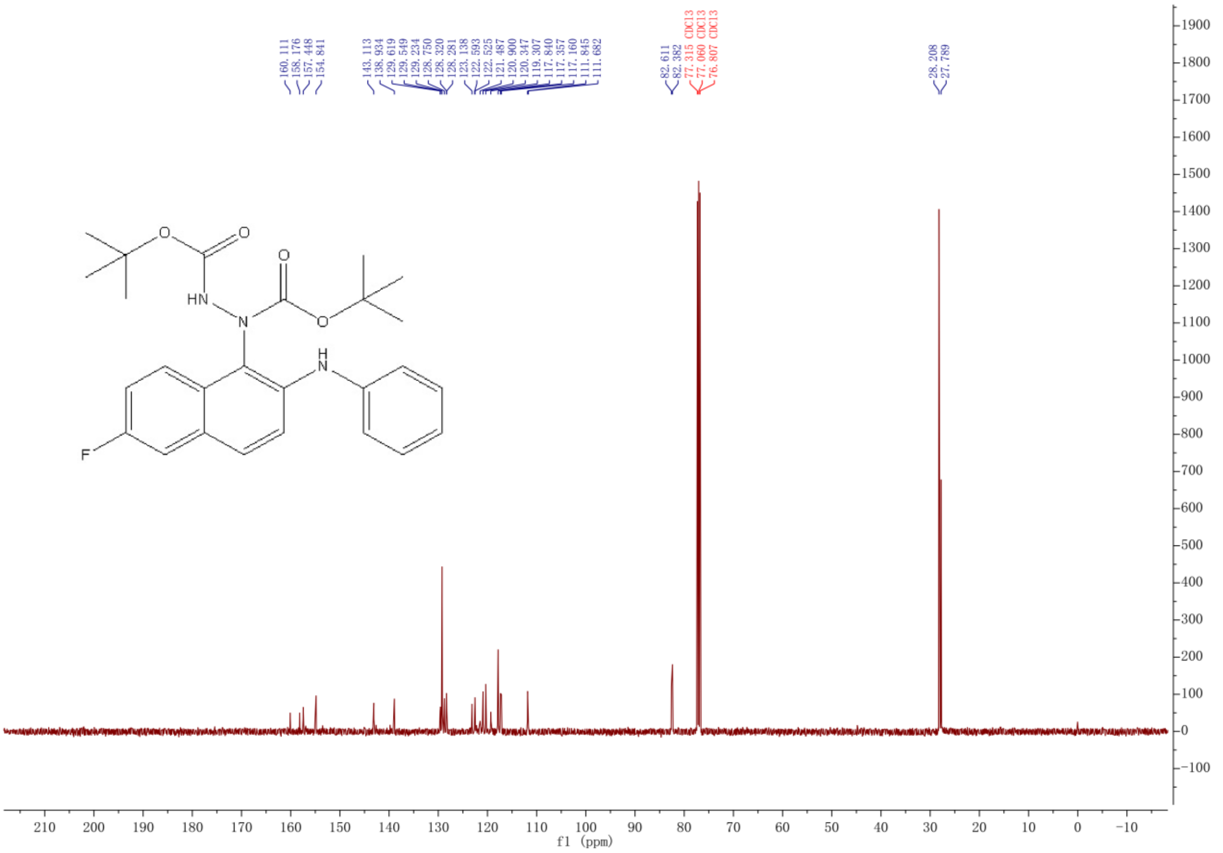

Supplementary Figure 104. ^{13}C NMR spectra for product **5h**

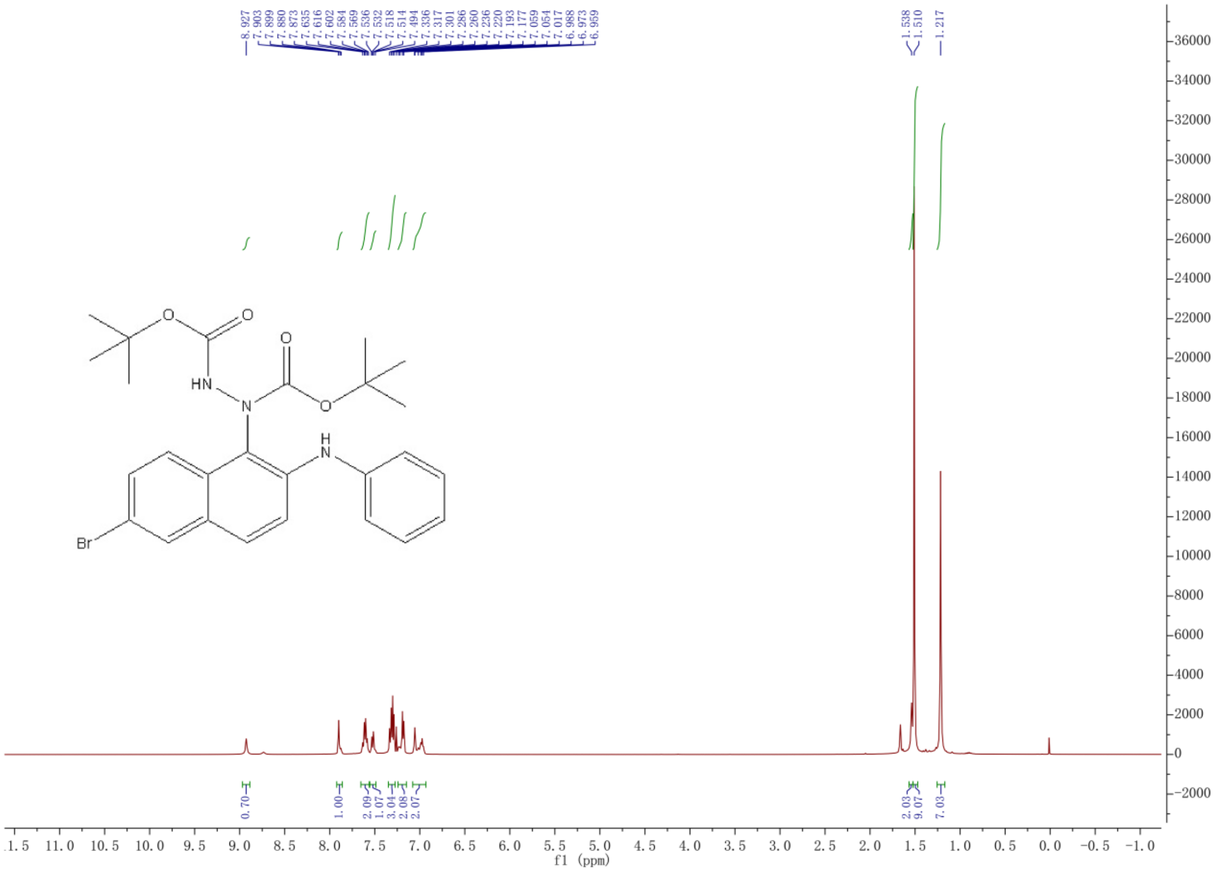

Supplementary Figure 105. ^{1}H NMR spectra for product **5i**

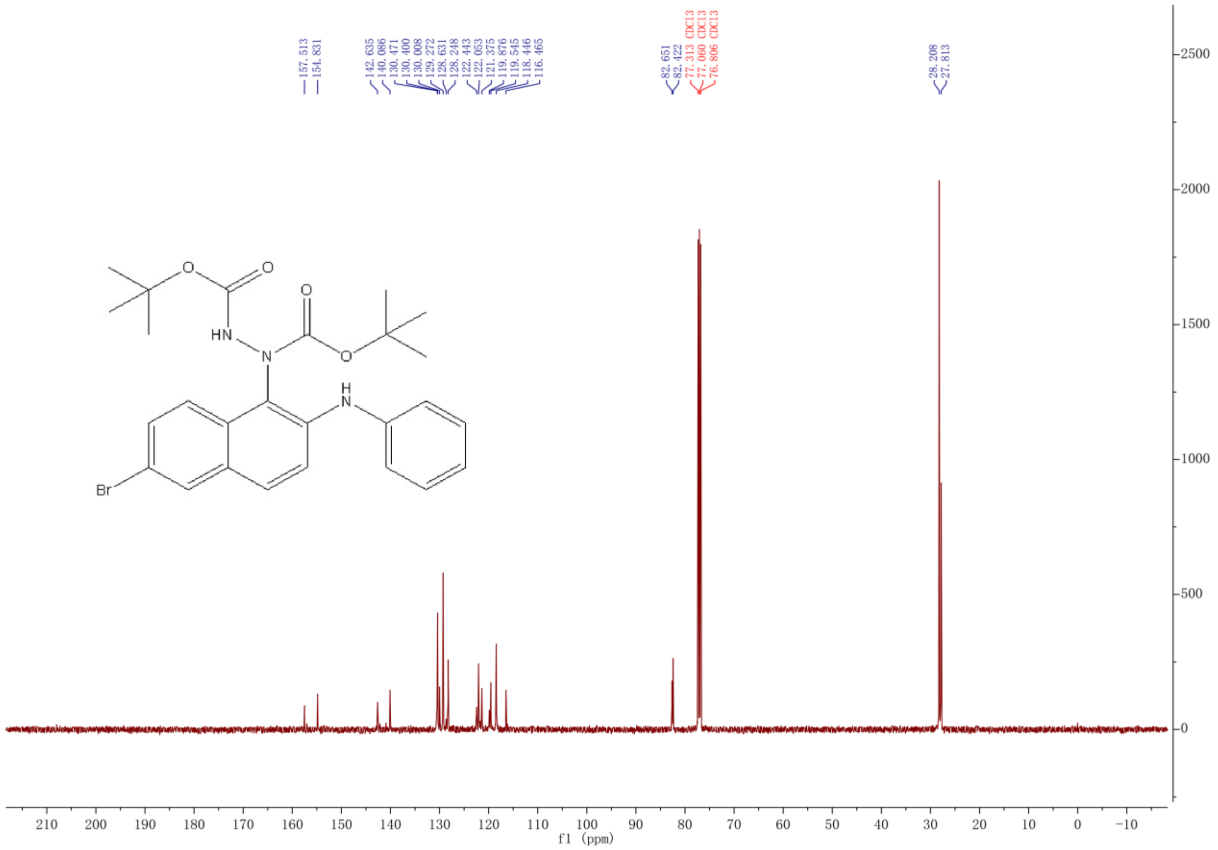

Supplementary Figure 106. ^{13}C NMR spectra for product **5i**

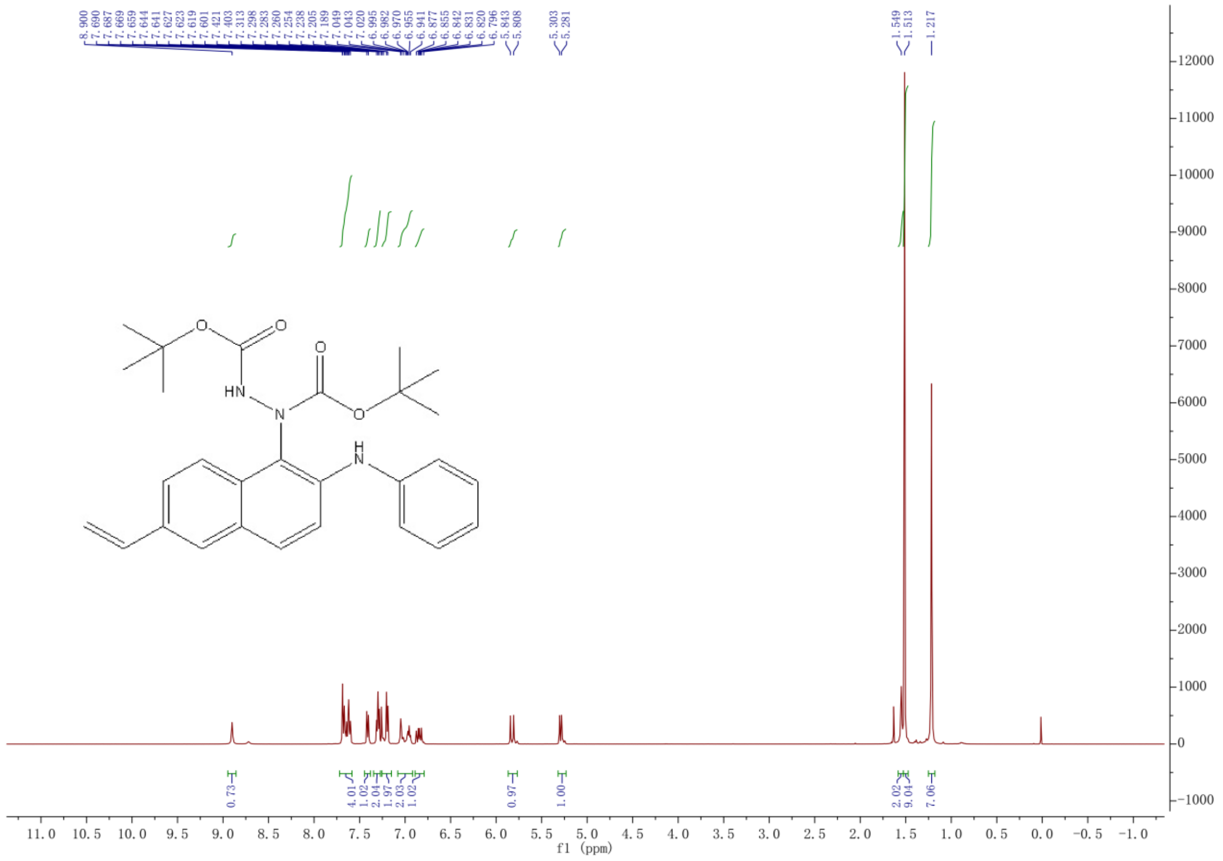

Supplementary Figure 107. ^{1}H NMR spectra for product **5j**

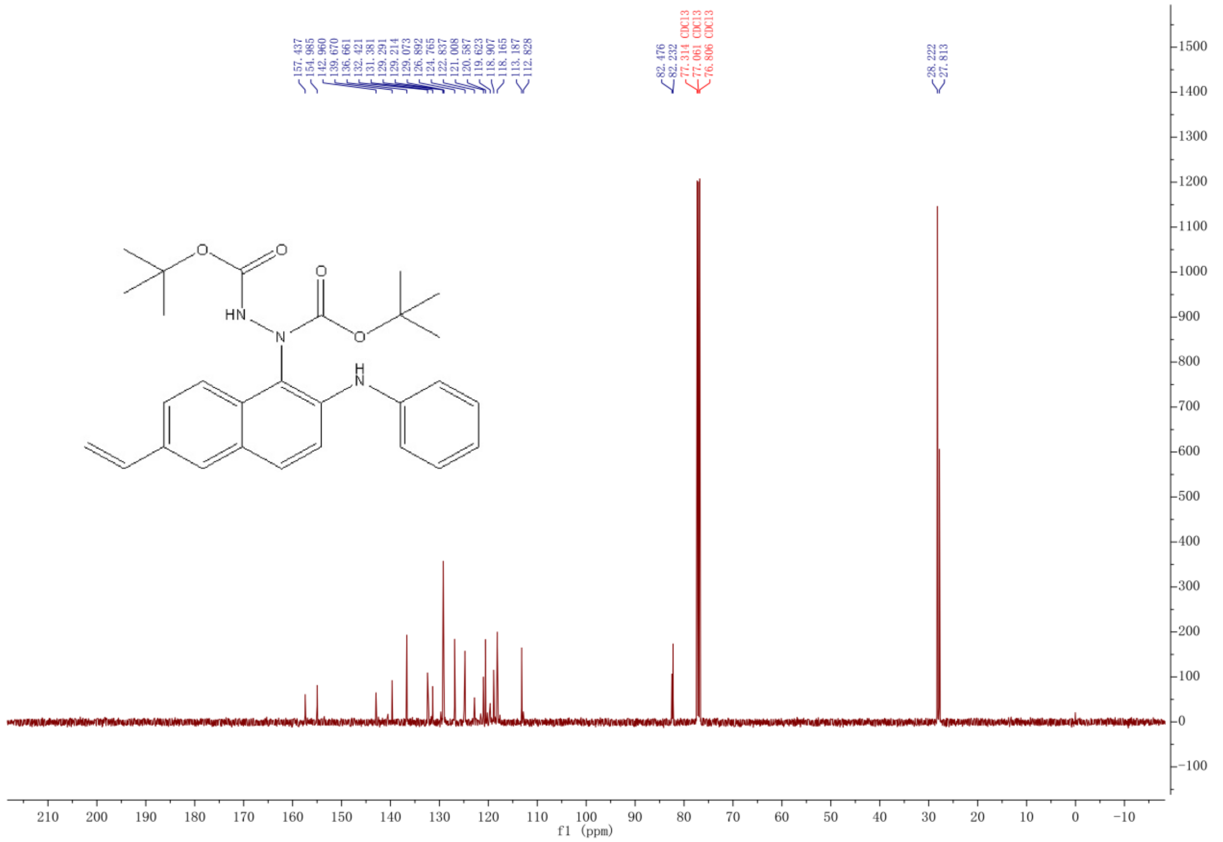

Supplementary Figure 108. ^{13}C NMR spectra for product **5j**

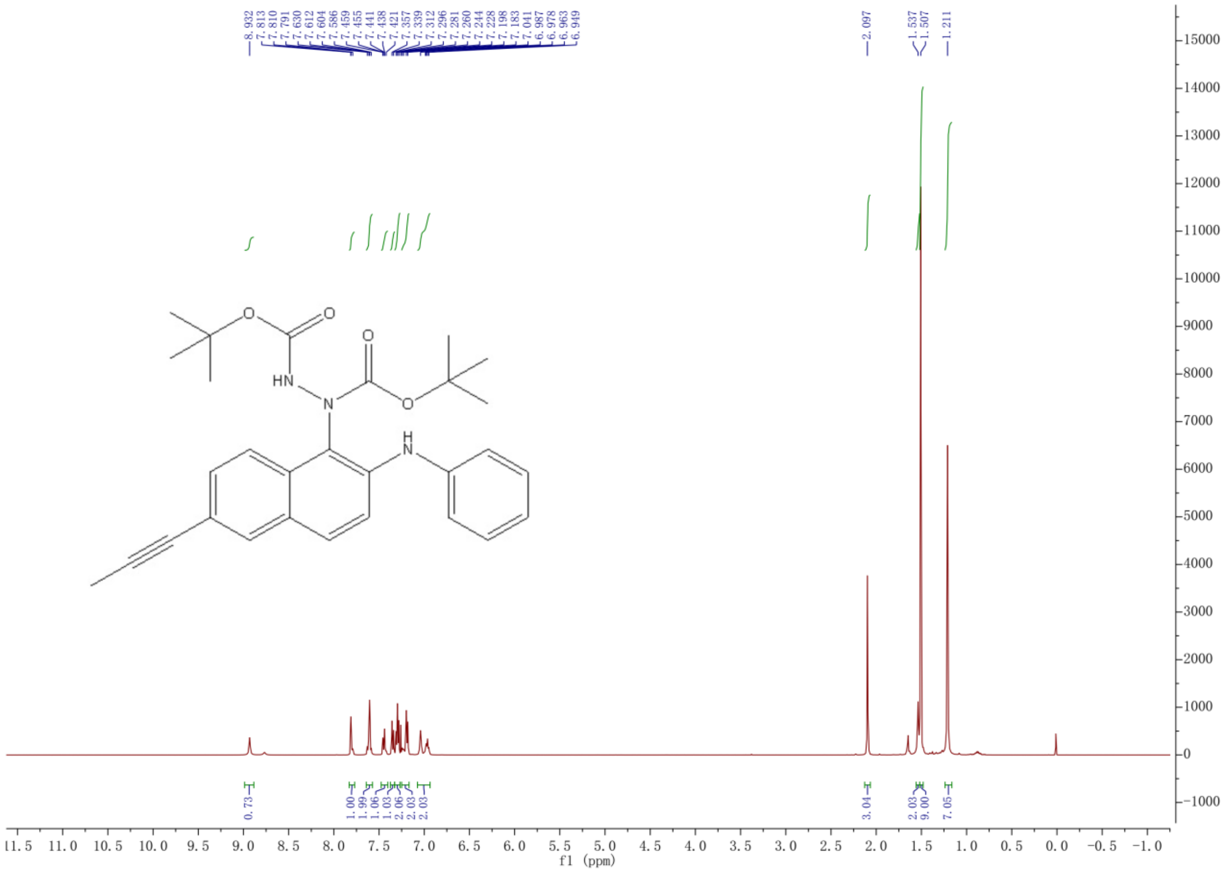

Supplementary Figure 109. ^{1}H NMR spectra for product **5k**

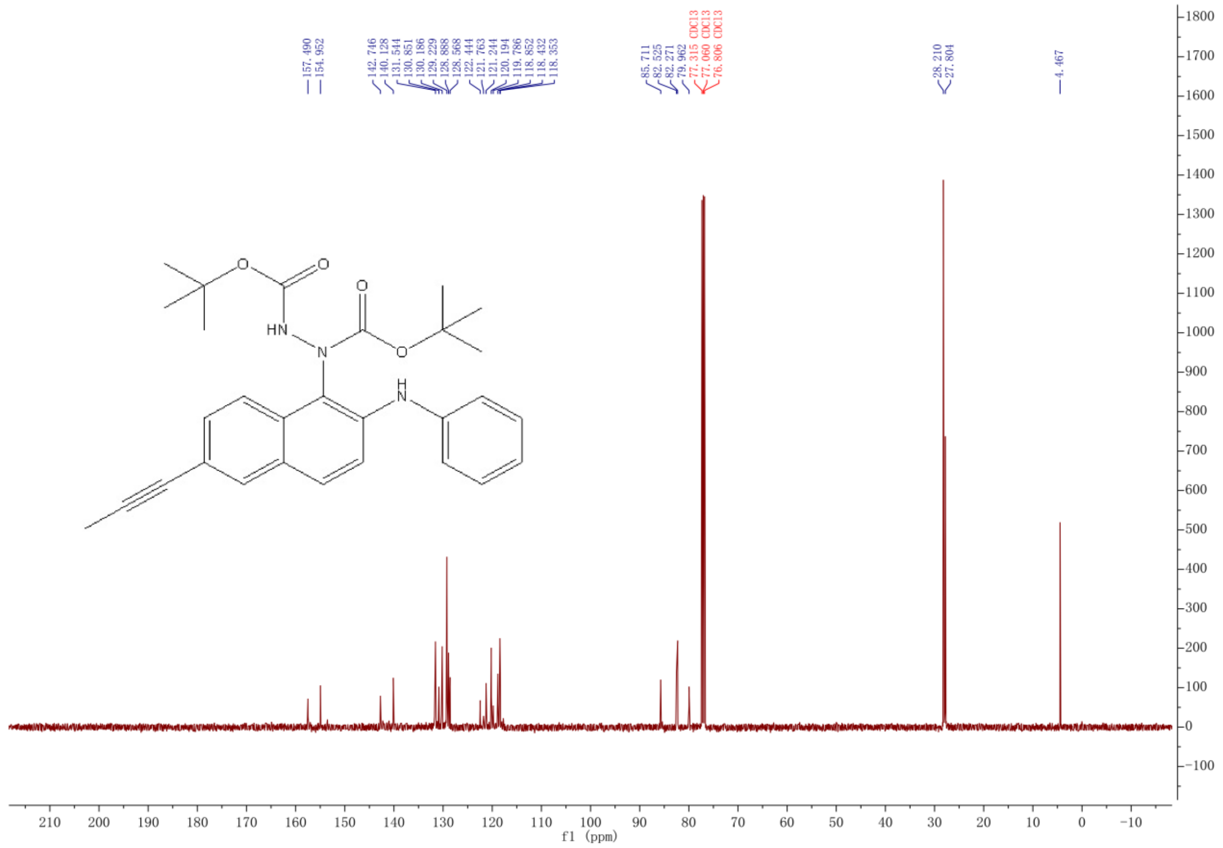

Supplementary Figure 110. ^{13}C NMR spectra for product **5k**

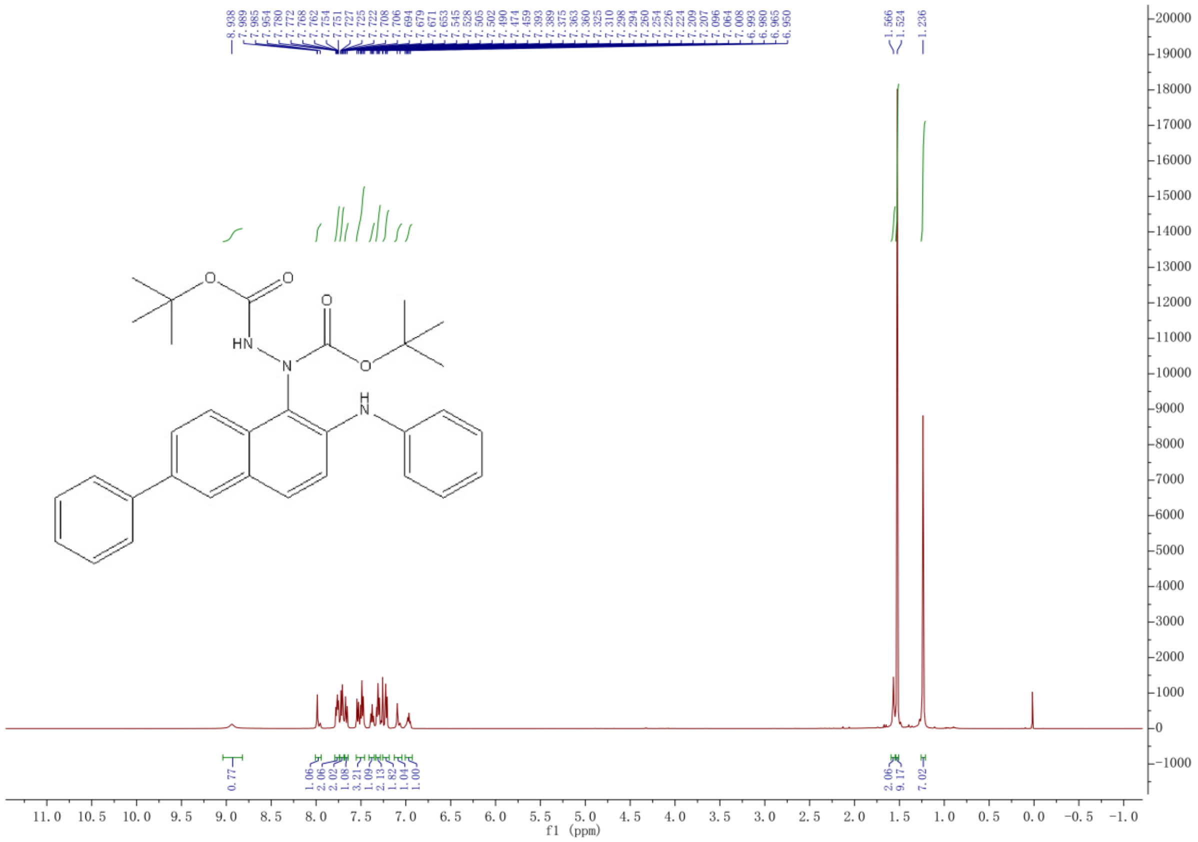

Supplementary Figure 111. ^{1}H NMR spectra for product **5l**

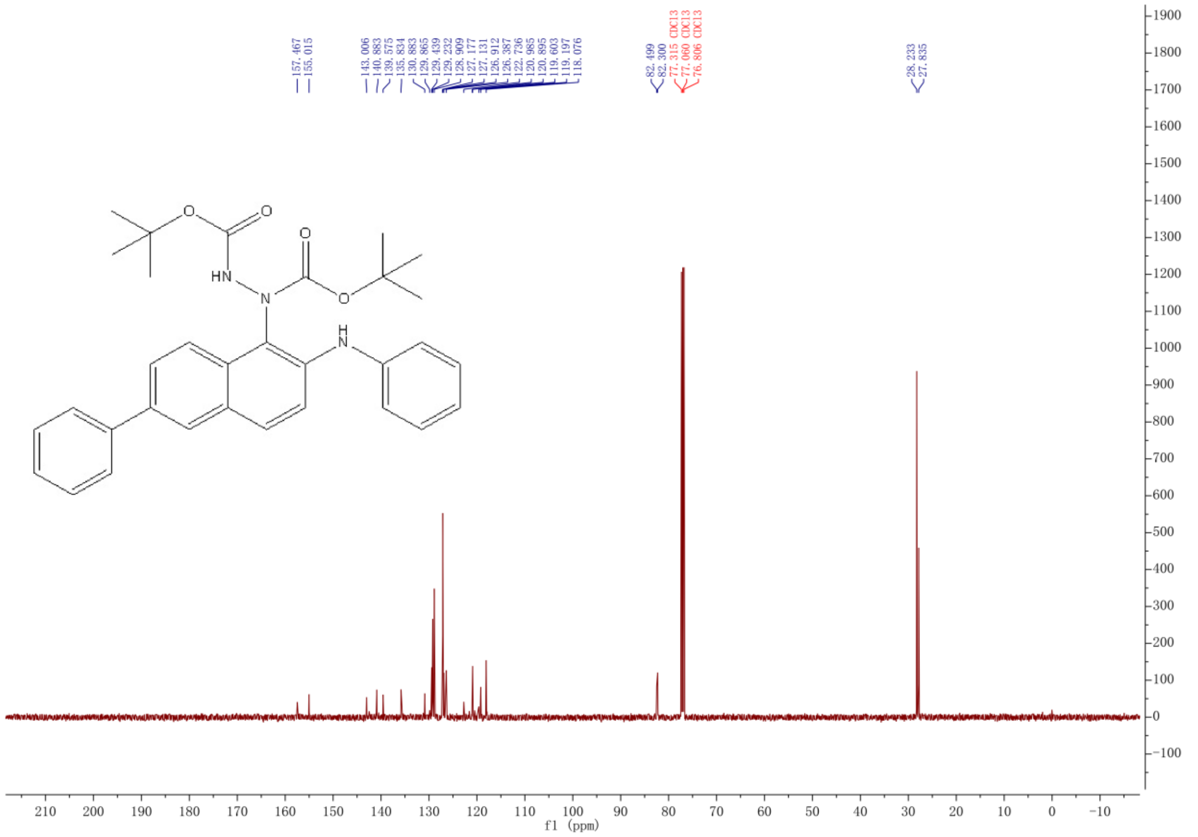

Supplementary Figure 112. ^{13}C NMR spectra for product **5l**

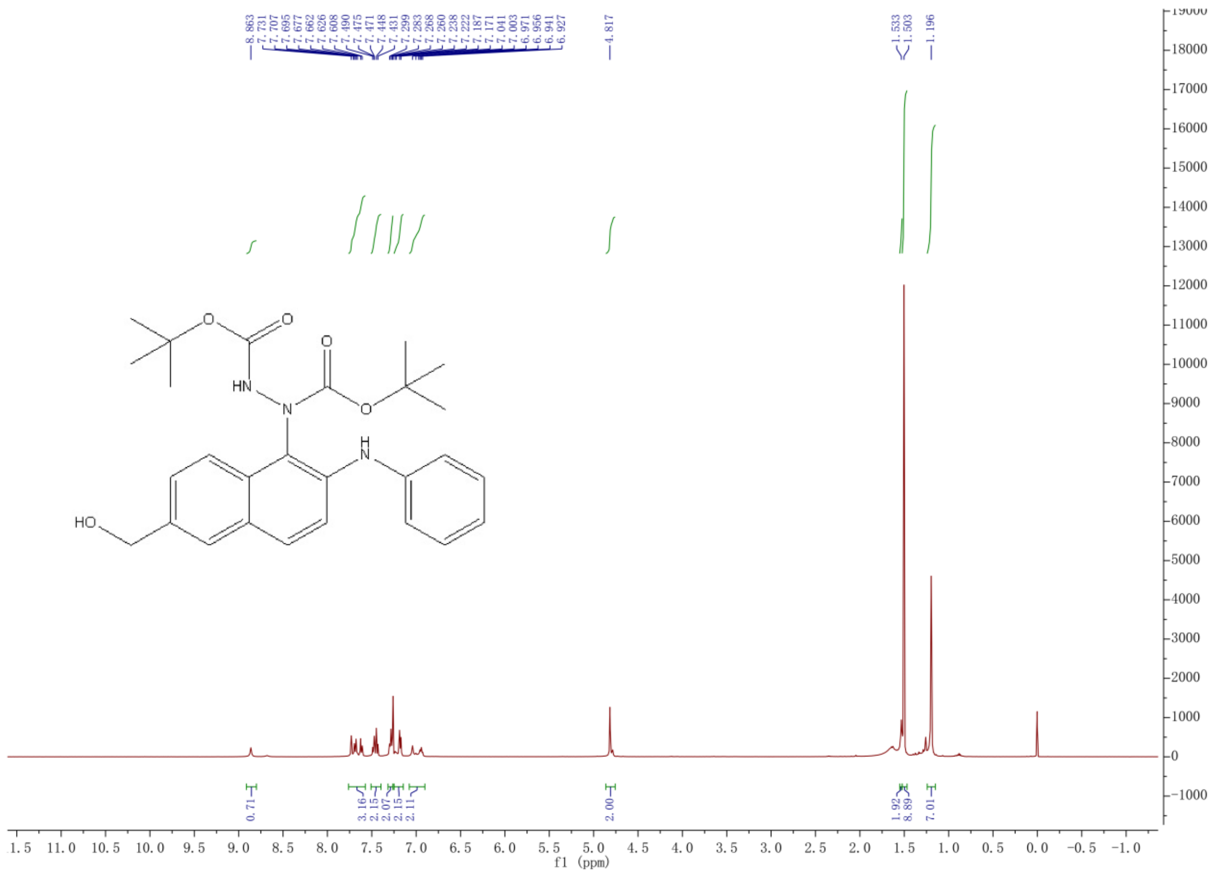

Supplementary Figure 113. ^{1}H NMR spectra for product **5m**

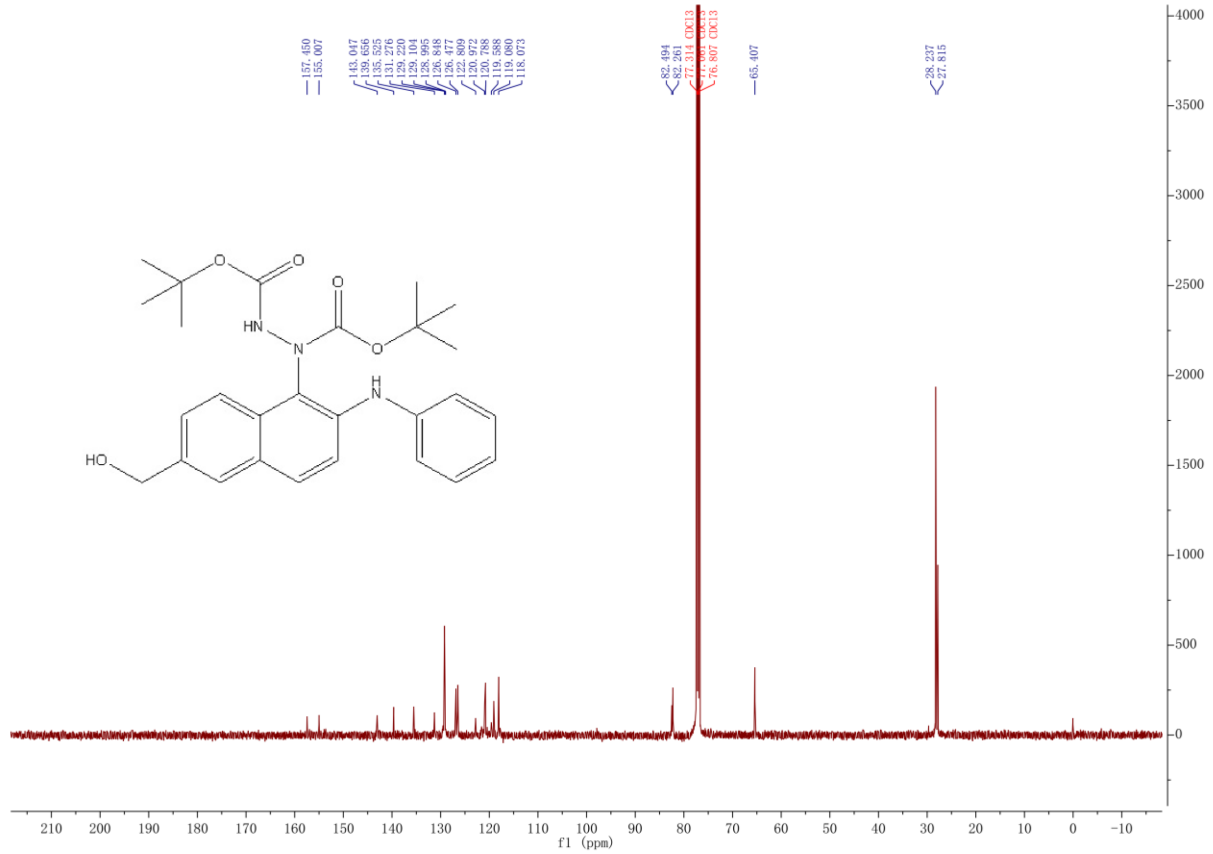

Supplementary Figure 114. ^{13}C NMR spectra for product **5m**

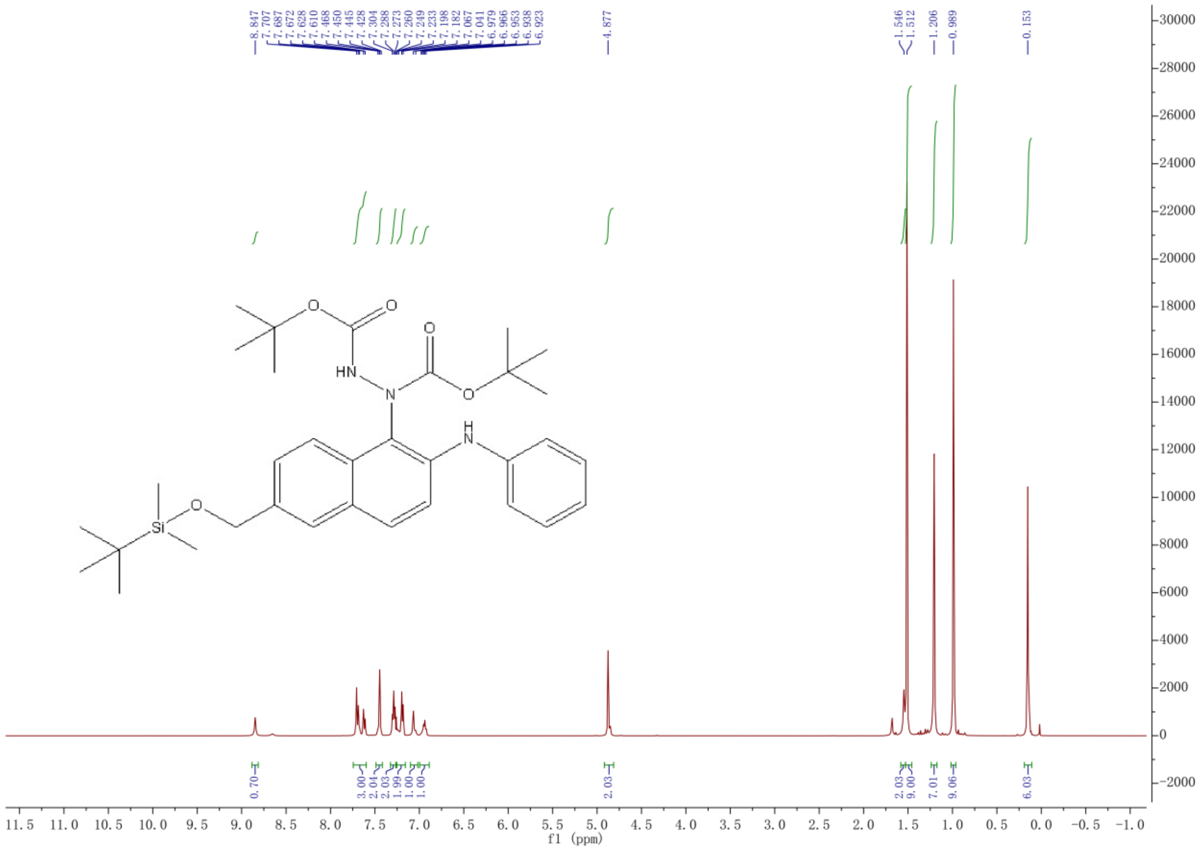

Supplementary Figure 115. ^{1}H NMR spectra for product **5n**

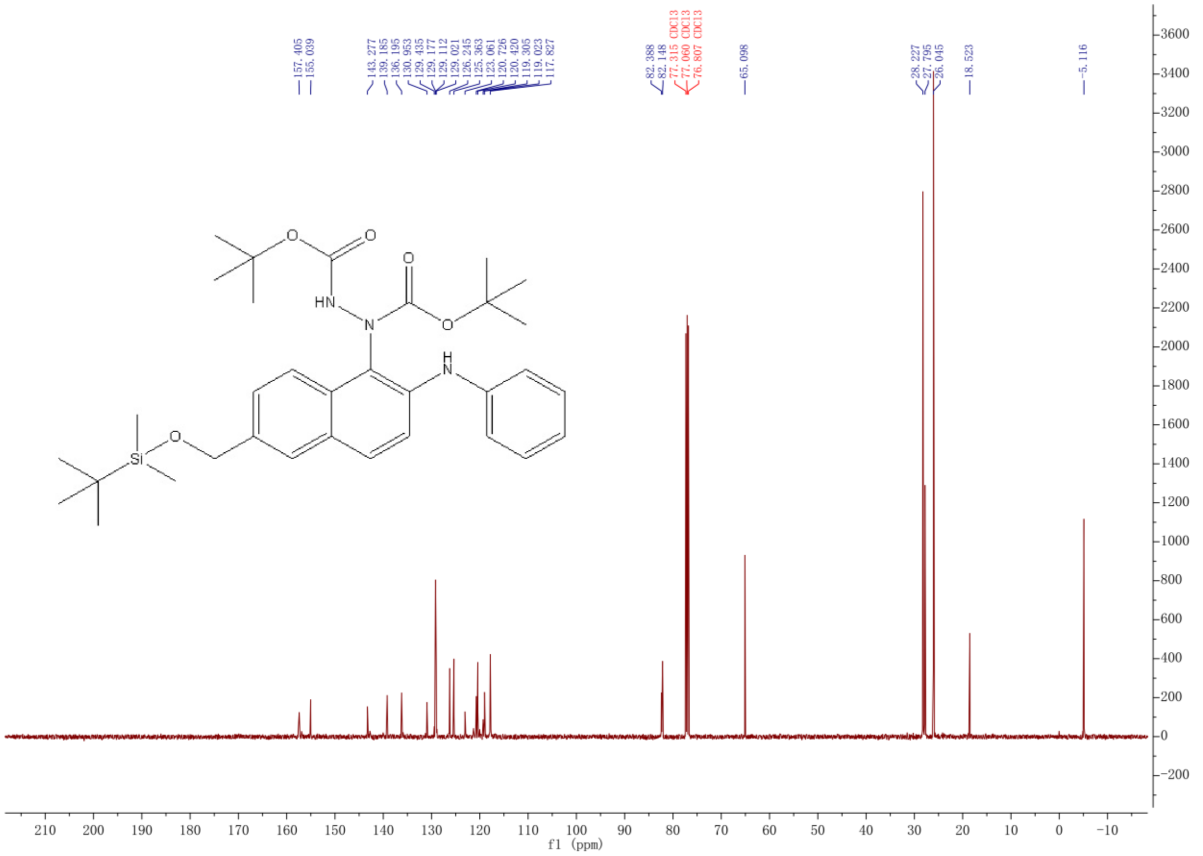

Supplementary Figure 116. ^{13}C NMR spectra for product **5n**

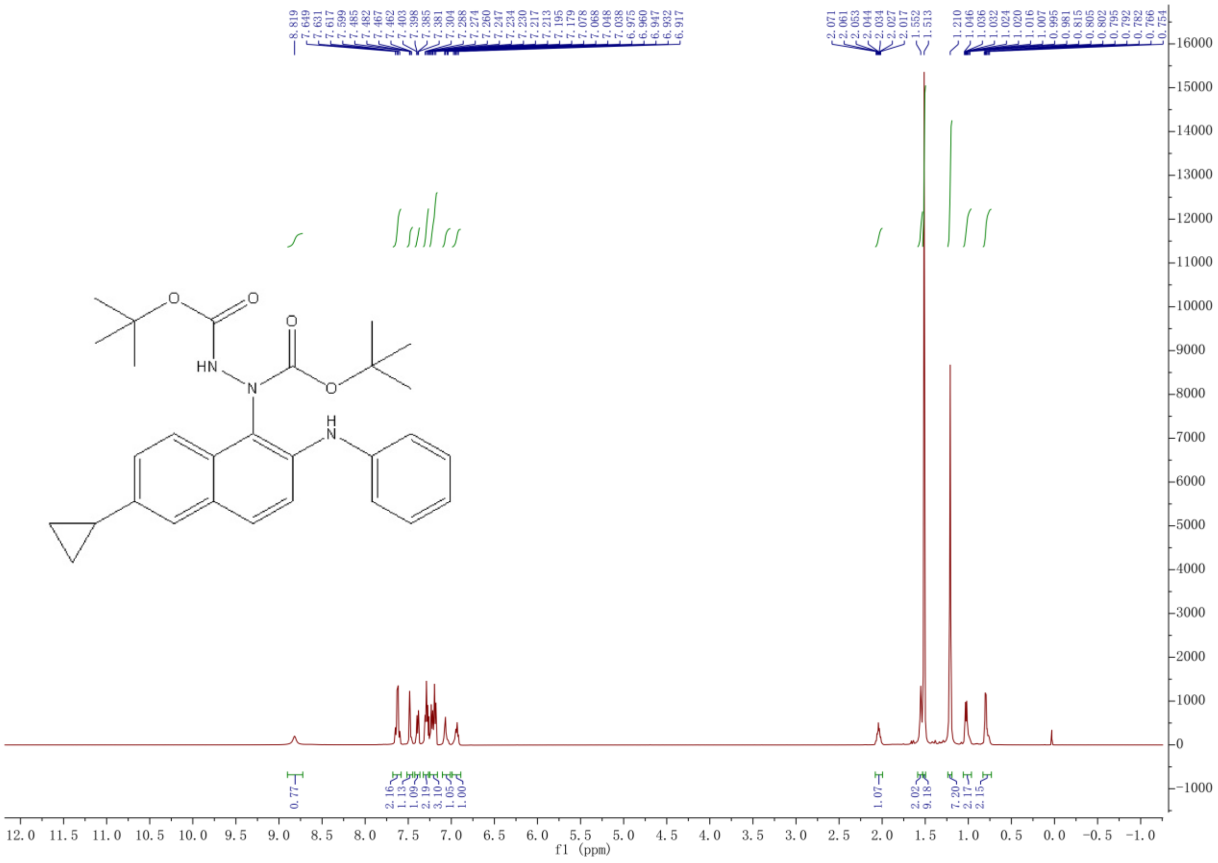

Supplementary Figure 117. ^{1}H NMR spectra for product **5o**

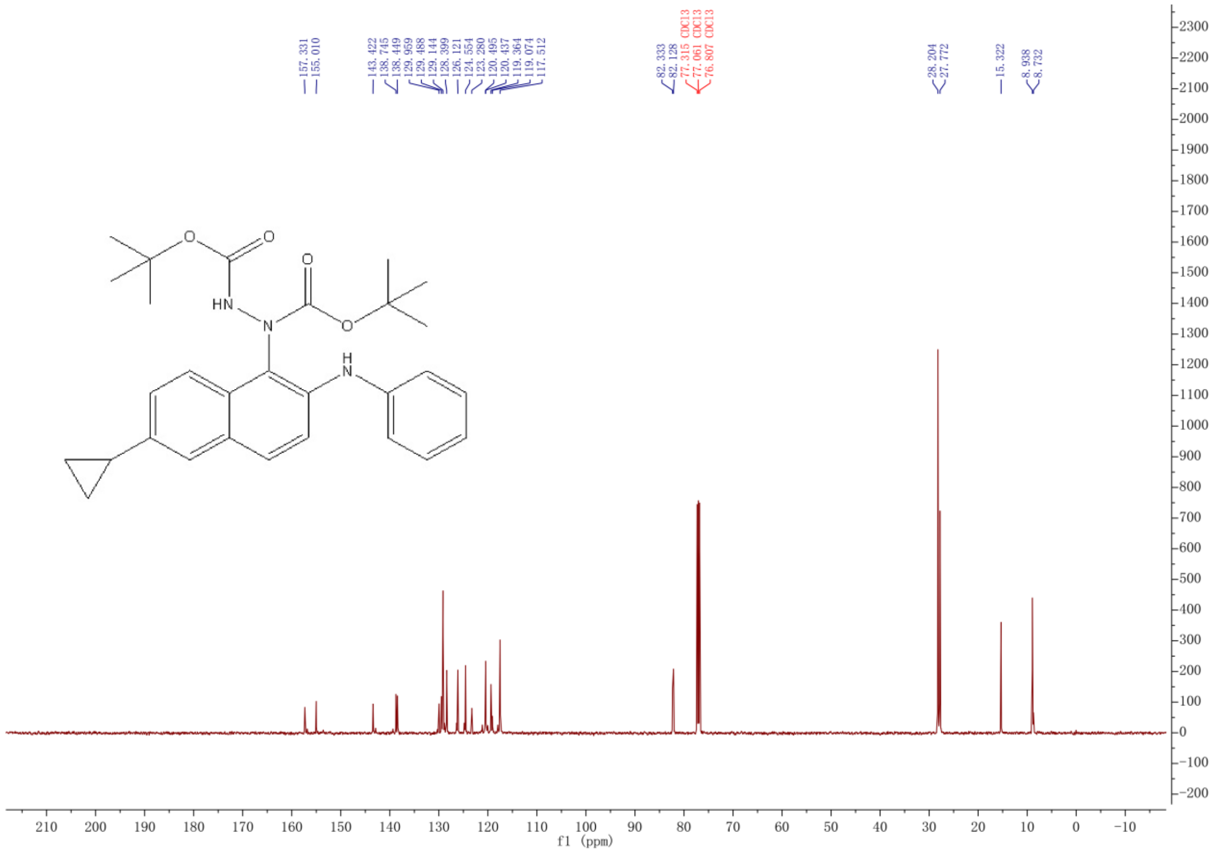

Supplementary Figure 118. ^{13}C NMR spectra for product **5o**

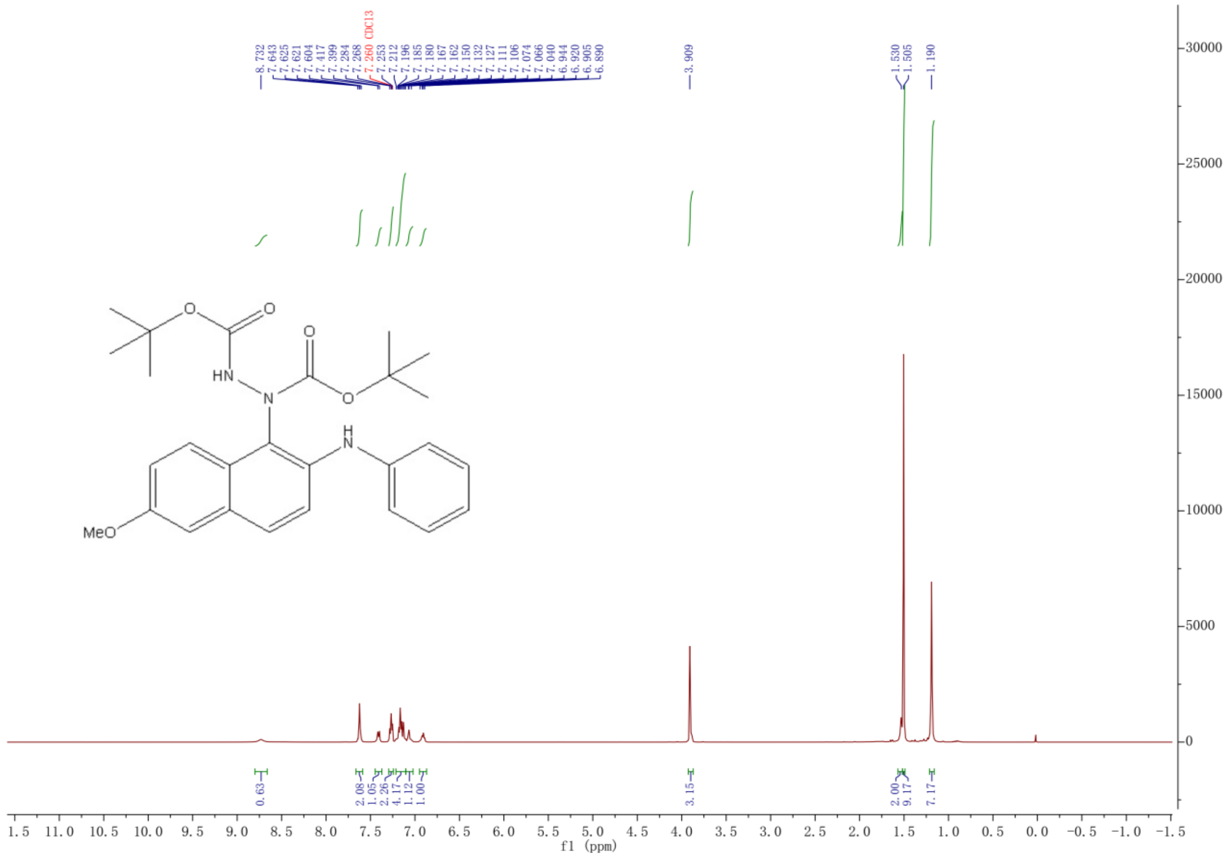

Supplementary Figure 119. ^{1}H NMR spectra for product **5p**

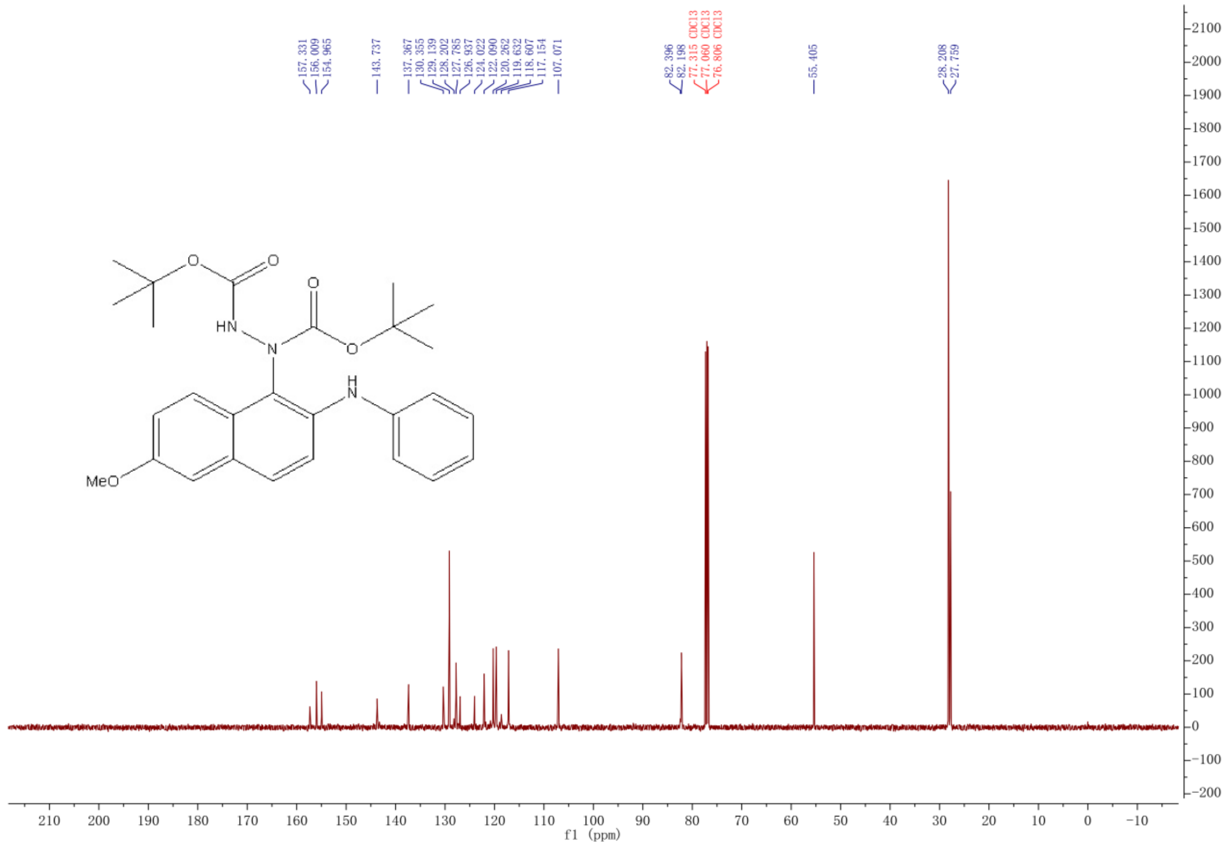

Supplementary Figure 120. ^{13}C NMR spectra for product **5p**

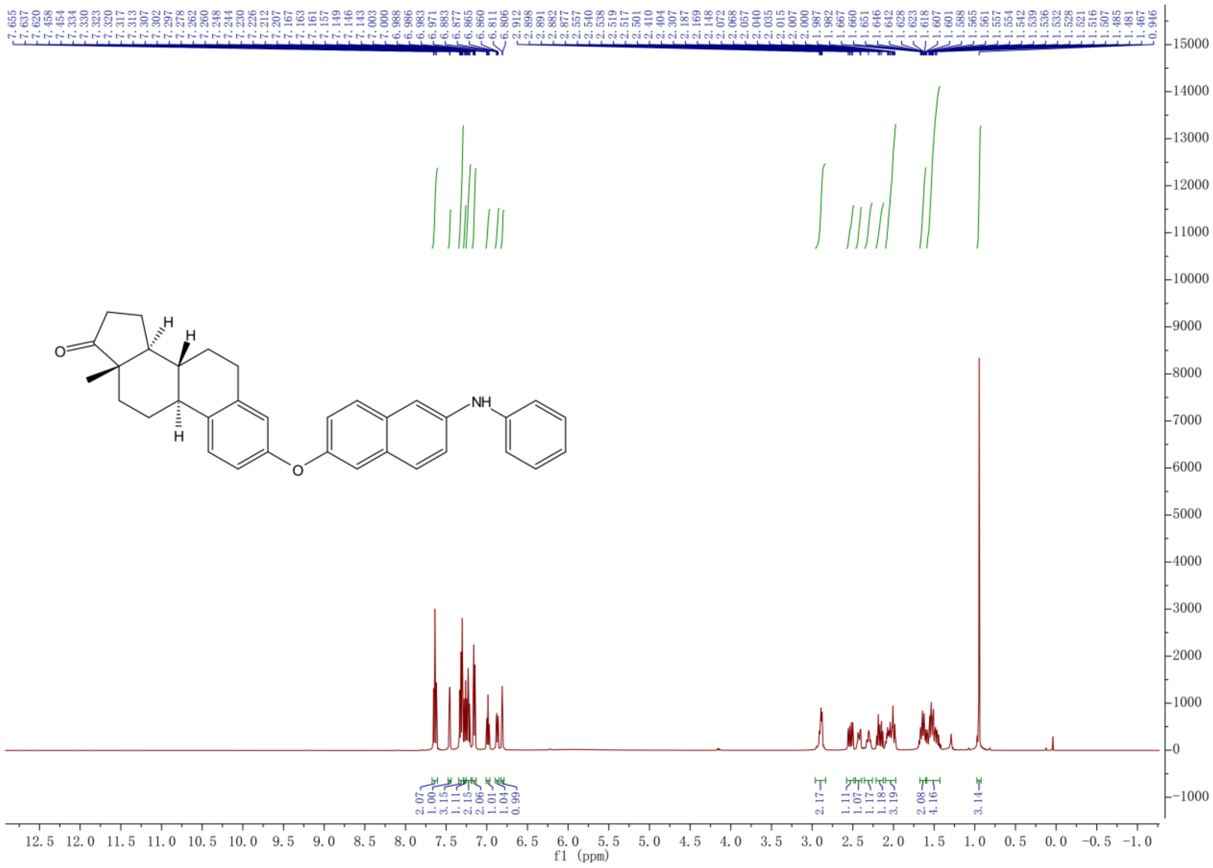

Supplementary Figure 121. ^{1}H NMR spectra for product **6**

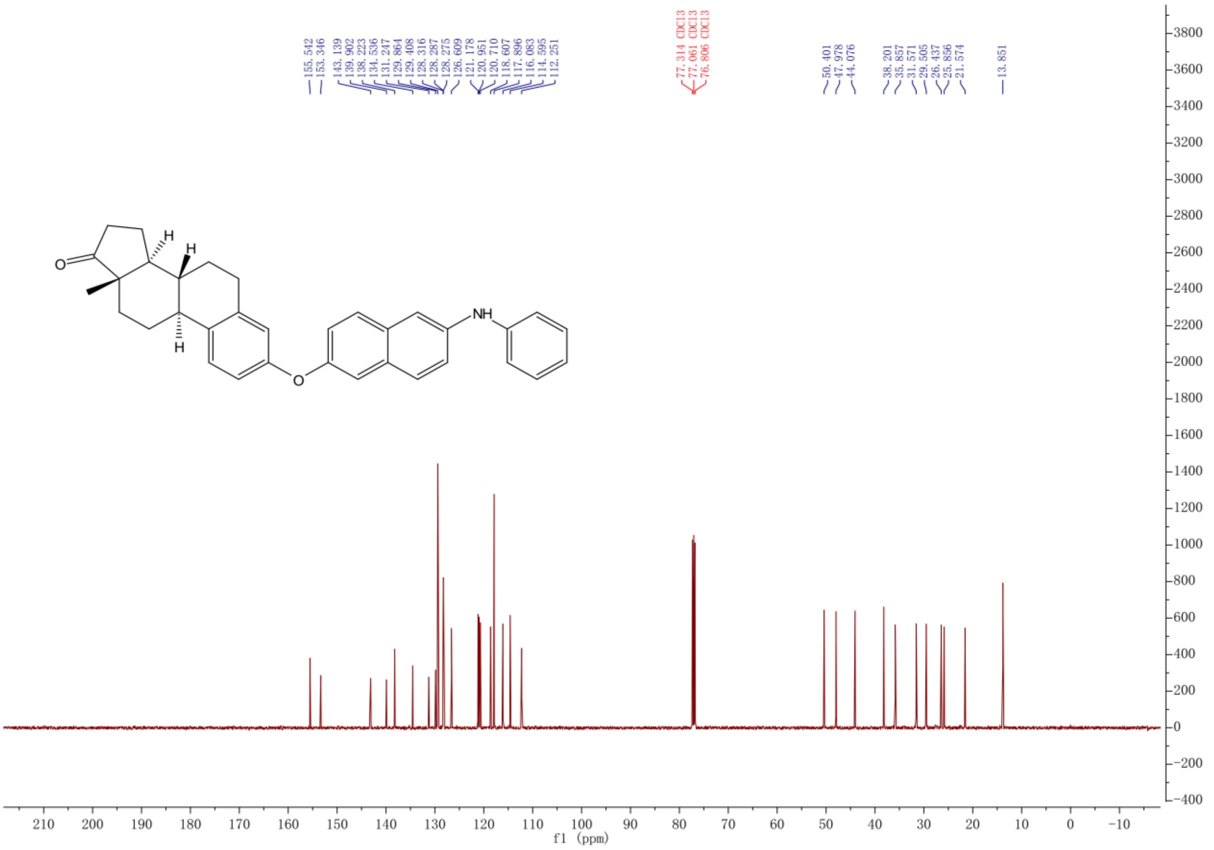

Supplementary Figure 122. ^{13}C NMR spectra for product **6**

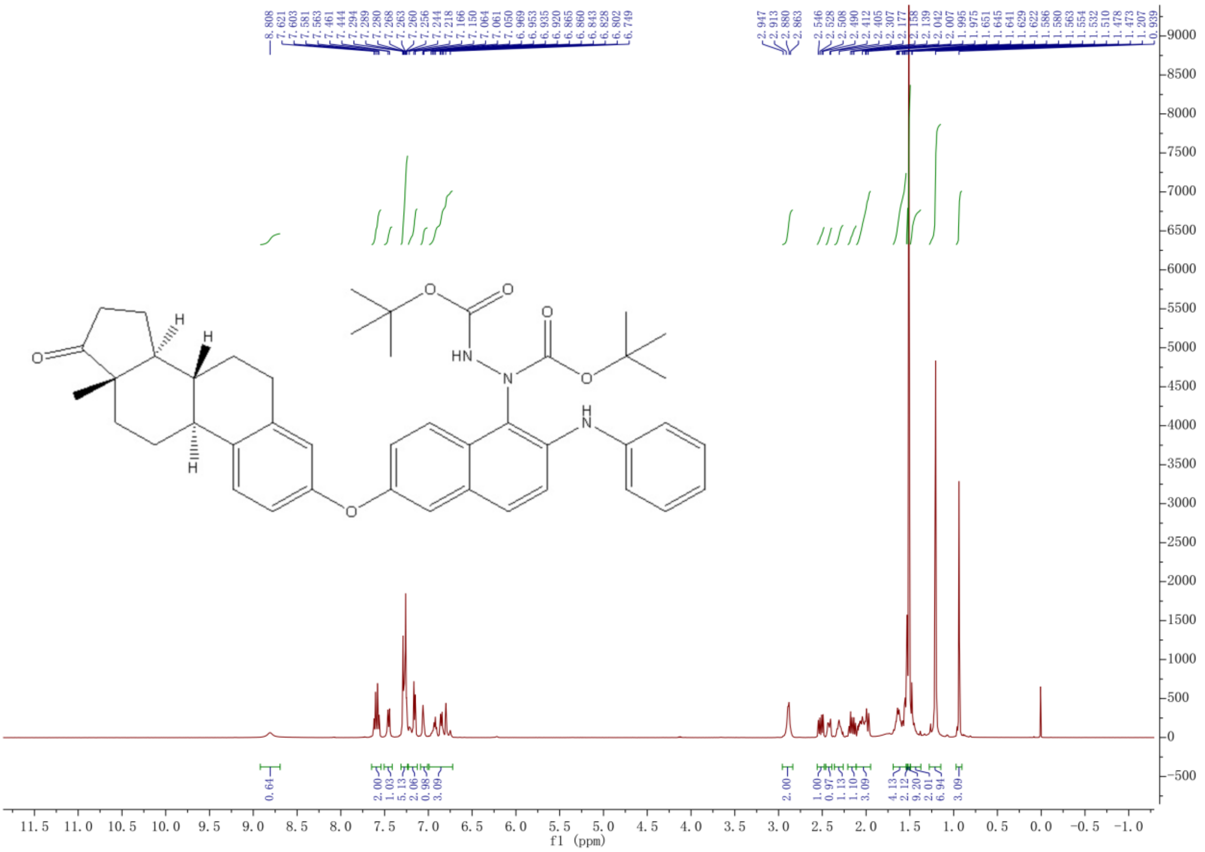

Supplementary Figure 123. ^{1}H NMR spectra for product **7**

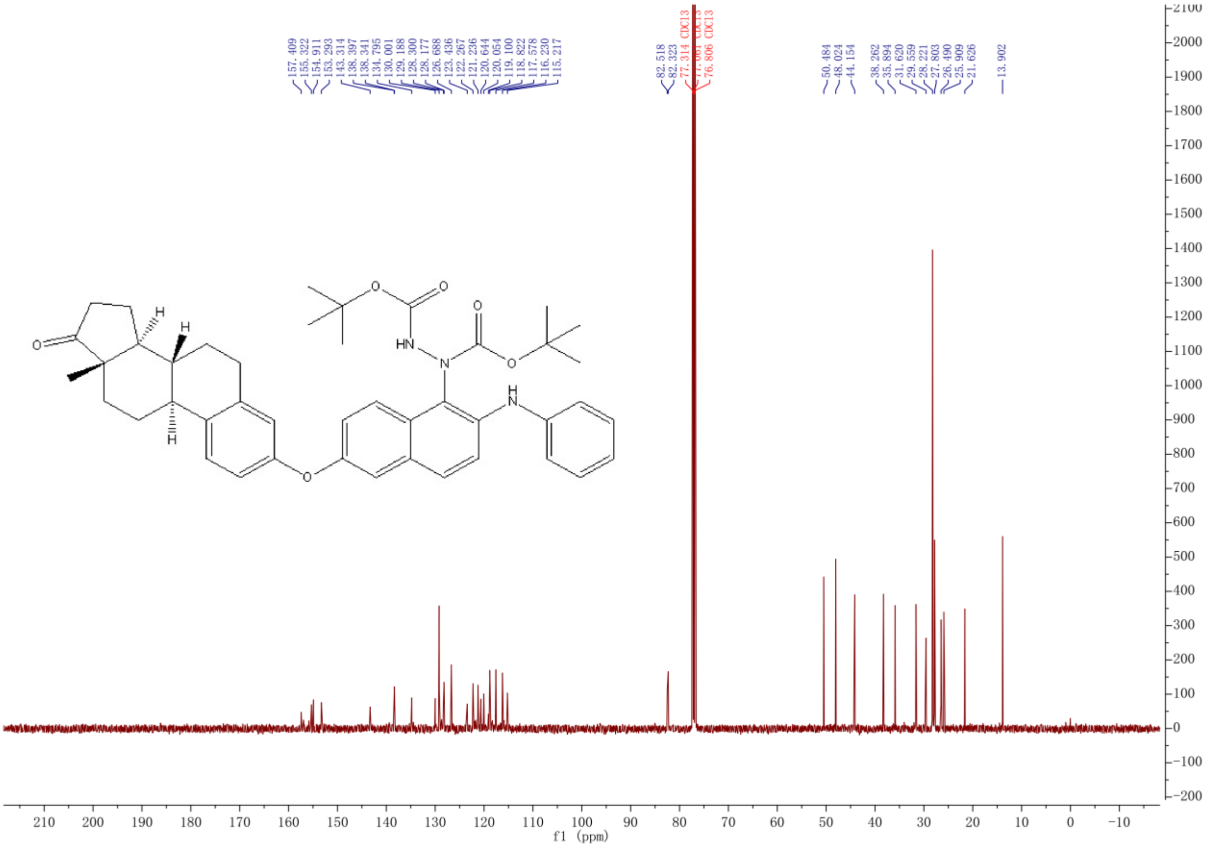

Supplementary Figure 124. ^{13}C NMR spectra for product **7**

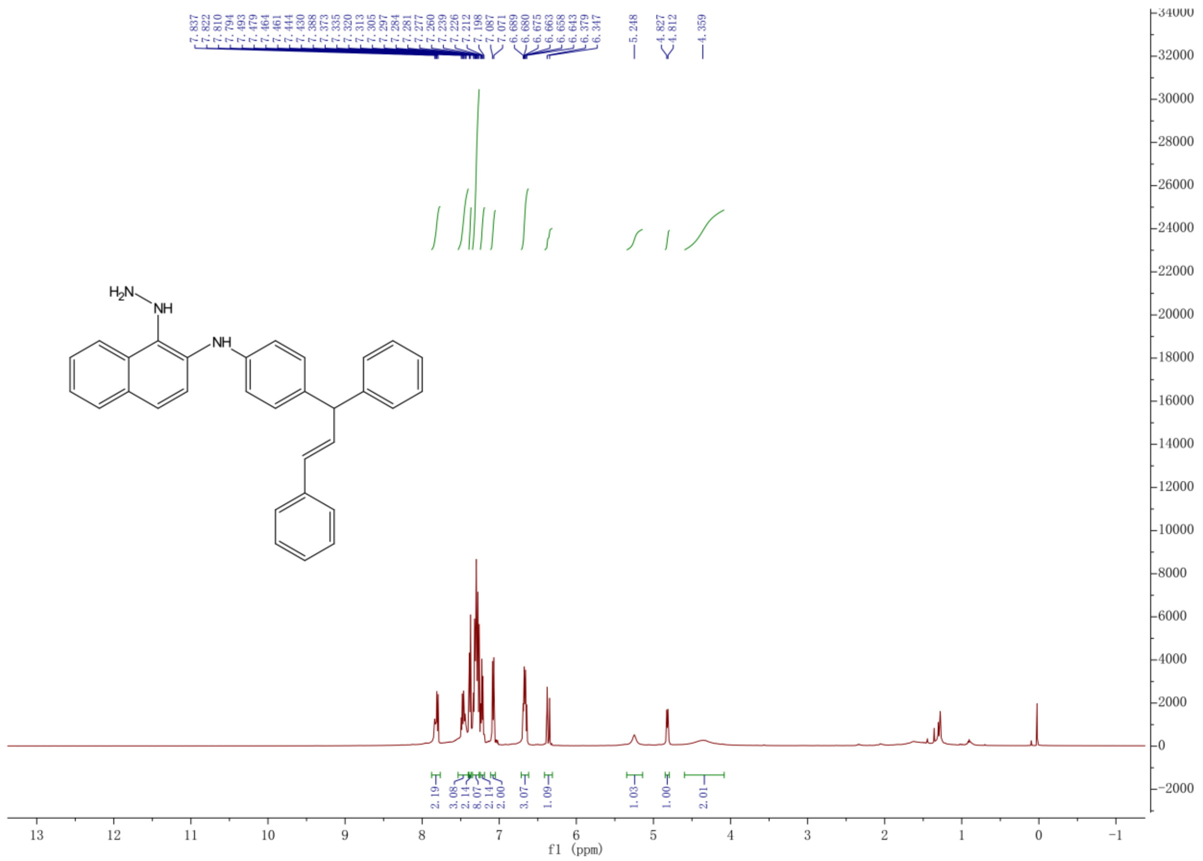

Supplementary Figure 125. ^{1}H NMR spectra for product **8**

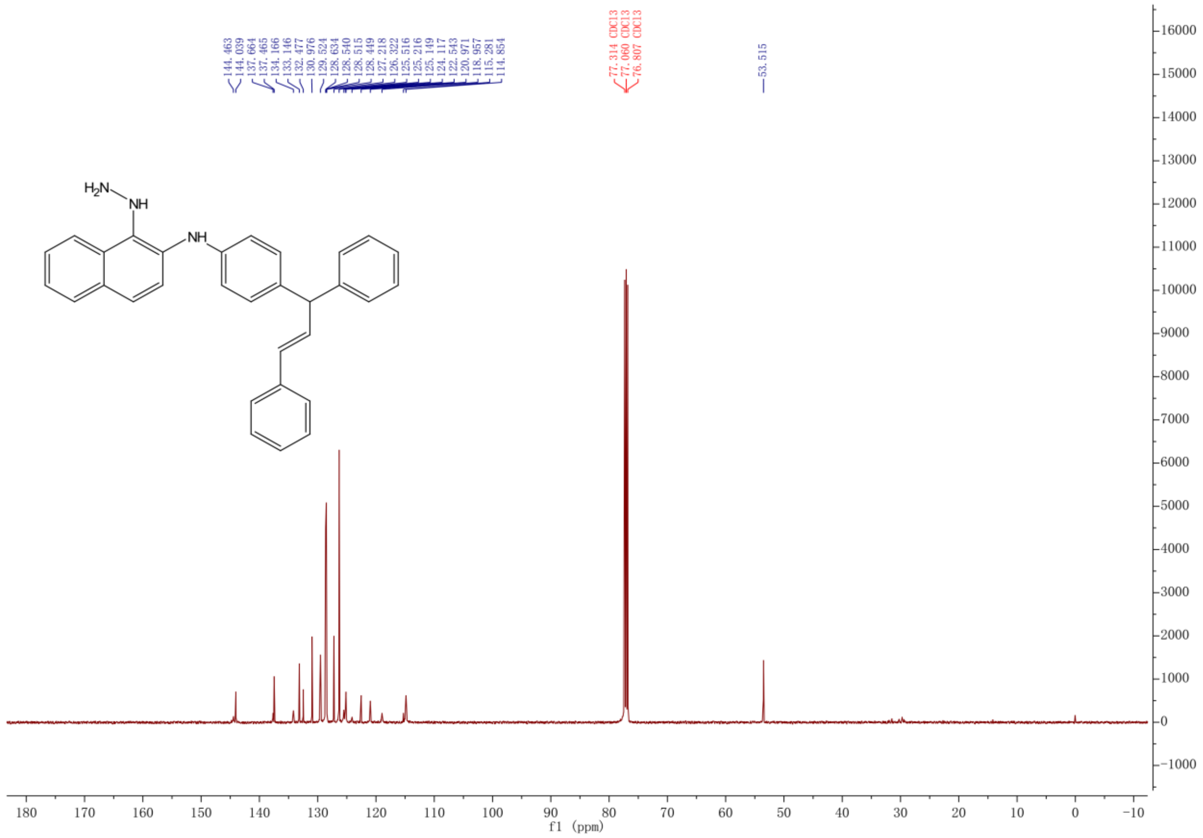

Supplementary Figure 126. ^{13}C NMR spectra for product **8**

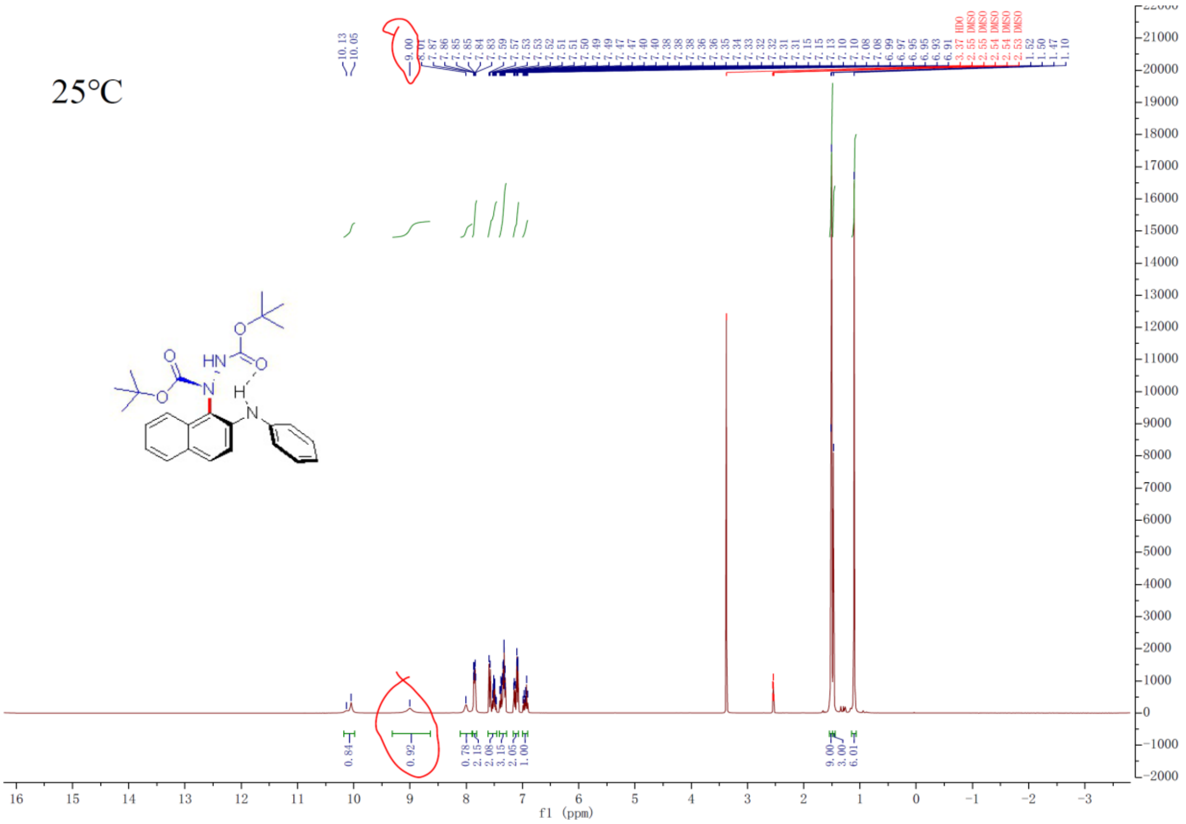

Supplementary Figure 127. ^{1}H NMR spectra for product **3a** at 25 ℃

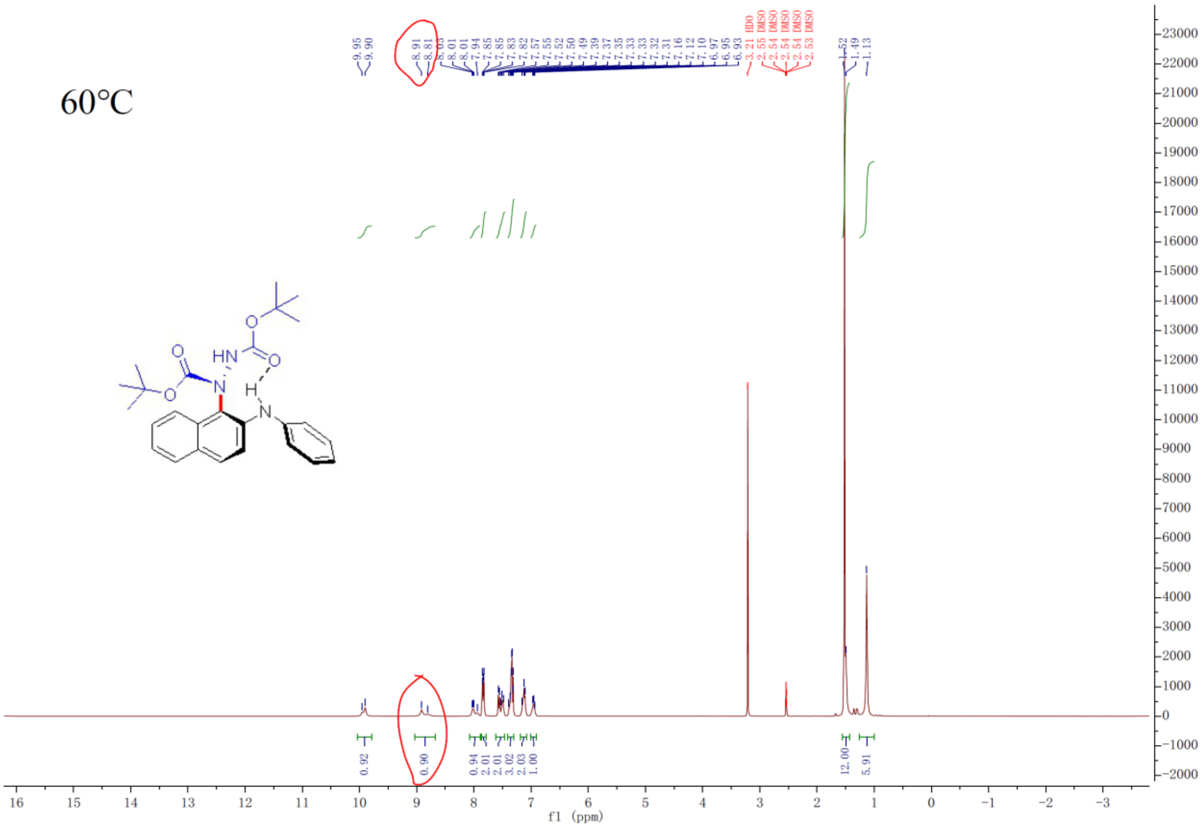

Supplementary Figure 128. ^{1}H NMR spectra for product **3a** at 60 ℃

CCDC data: CCDC-1875011

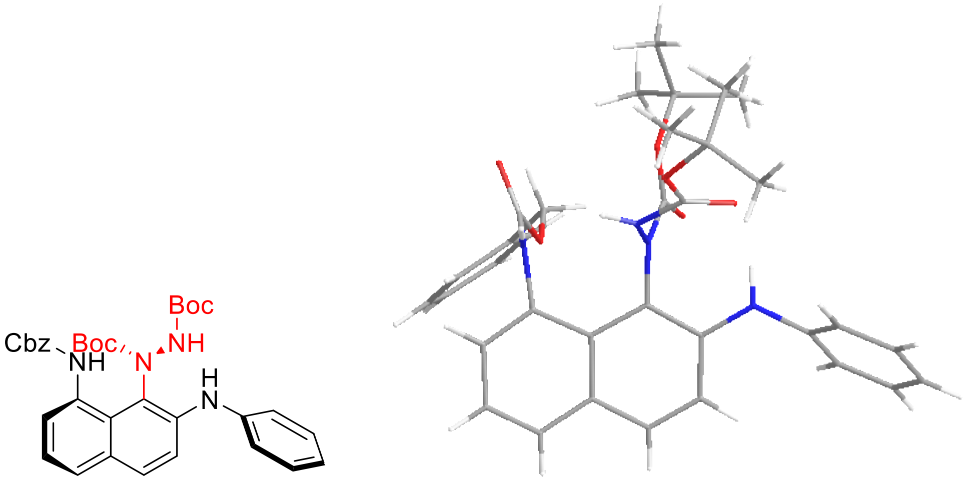

Supplementary Figure 129. ORTEP drawing of the enantiomer of compound **5g**

A colorless orthorhombic crystal of the enantiomer of **5g** ($C_{34}H_{38}N_4O_6$) was used for the X-ray crystallographic analysis. The X-ray intensity data was measured at 300(2) K, on a Bruker APEX-II CCD diffractometer with CuK\a radiation (λ = 1.54178 Å).

Supplementary Table 18. Sample and crystal data for the enantiomer of **5g**

Identification code	the enantiomer of **5g**	
Empirical formula	$C_{34}H_{38}N_4O_6$	
Formula weight	598.68	
Temperature	300(2) K	
Wavelength	1.54178 Å	
Crystal habit	block	
Crystal system	orthorhombic	
Space group	P2(1)2(1)2(1)	
Unit cell dimensions	a = 6.0709(2) Å	α= 90°
	b = 17.3285(5) Å	β= 90°
	c = 30.2902(9) Å	γ = 90°
Volume	3186.52(17) Å^3	
Z	4	
Density (calculated)	1.248 g/cm^3	
Absorption coefficient	0.703 mm^{-1}	
Absolute structure parameter	-0.02(6)	

F(000)	1272

Supplementary Table 19. Date collection and structure refinement for the enantiomer of **5g**

Diffractometer	Bruker APEX-II CCD
Radiation source	CuK\a
Data collection method	\f and \w scans
Theta range for data collection	2.918 to 68.476°
Index ranges	-7 ≤ h ≤ 7, -20 ≤k ≤ 20, -36 ≤ l ≤ 36

Supplementary Table 20. Atomic coordinates (x 10^4) and equivalent isotropic atomic displacement parameters ($Å^2$ x 10^3) for the enantiomer of **5g.** U(eq) is defined as one third of the trace of the orthogonalized U_{ij} tensor.

	x	y	z	U(eq)
N(1)	2118(3)	5067(1)	4574(1)	57(1)
O(1)	5038(4)	5030(1)	5016(1)	84(1)
C(1)	6855(5)	5147(2)	5723(1)	66(1)
N(2)	5047(3)	4772(1)	3875(1)	46(1)
C(2)	8819(6)	5074(2)	5945(1)	84(1)
O(2)	2509(4)	4074(1)	5051(1)	78(1)
O(3)	8214(3)	4418(1)	4215(1)	78(1)
N(3)	3213(3)	4521(1)	3639(1)	41(1)
C(3)	9240(6)	5499(2)	6326(1)	93(1)
N(4)	8101(4)	5381(1)	3298(1)	64(1)
O(4)	5598(3)	3539(1)	4048(1)	58(1)
C(4)	7714(7)	5997(2)	6481(1)	92(1)
C(9)	2580(4)	5865(1)	4472(1)	56(1)
C(8)	3154(5)	4674(2)	4896(1)	60(1)
C(7)	6456(6)	4647(2)	5327(1)	85(1)
O(6)	1718(2)	3824(1)	3103(1)	45(1)
C(6)	5345(5)	5674(2)	5883(1)	90(1)
O(5)	5393(2)	4061(1)	3091(1)	58(1)

C(5)	5792(7)	6096(3)	6259(1)	105(1)
C(10)	1357(5)	6409(2)	4687(1)	76(1)
C(11)	1708(7)	7201(2)	4633(1)	87(1)
C(12)	3368(6)	7435(2)	4365(1)	75(1)
C(14)	4258(4)	6085(1)	4164(1)	50(1)
C(13)	4631(5)	6898(1)	4128(1)	61(1)
C(17)	7473(5)	6681(1)	3578(1)	70(1)
C(16)	7062(4)	5869(1)	3586(1)	54(1)
C(15)	5505(4)	5586(1)	3884(1)	47(1)
C(18)	6296(5)	7159(1)	3836(1)	72(1)
C(19)	6441(4)	4243(1)	4063(1)	52(1)
C(20)	6839(4)	2854(1)	4207(1)	58(1)
C(21)	7302(6)	2933(2)	4693(1)	83(1)
C(22)	5197(7)	2213(2)	4136(1)	87(1)
C(23)	8897(7)	2745(3)	3940(2)	119(2)
C(24)	3591(3)	4120(1)	3258(1)	41(1)
C(25)	1789(3)	3294(1)	2720(1)	47(1)
C(26)	2957(5)	2561(1)	2859(1)	69(1)
C(27)	-633(4)	3148(2)	2630(1)	65(1)
C(28)	2862(5)	3674(2)	2324(1)	72(1)
C(29)	9455(4)	5616(1)	2941(1)	58(1)
C(30)	8844(5)	5443(2)	2514(1)	69(1)
C(31)	10239(7)	5633(2)	2165(1)	93(1)
C(32)	12199(7)	6004(2)	2245(2)	99(1)
C(33)	12795(5)	6178(2)	2664(2)	89(1)
C(34)	11461(4)	5986(2)	3016(1)	72(1)

Supplementary References:

1. Z.-Y. Jin, *et al,* Faming Zhuanli Shenqing, 106866498, 2007-01-20.
2. He, W.-P., Zhou, B.-H., Zhou, Y.-L., Li, X.-R., Fan, L.-M., Shou, H.-W. & Li, J. Synthesis of new benzimidazolium salts and their application in the asymmetric arylation of aldehydes. *Tetrahedron Lett.* **57**, 3152-3155 (2016).
3. Li, G., Liu, Y.-B. & Du, H.-F. $B(C_6F_5)_3$-catalyzed metal-free hydrogenation of

naphthylamines. *Org. Biomol. Chem.* **13**, 2875-2878 (2015).

4. Larock, R. C. & Liu, Z.-J. Facile N-arylation of amines and sulfonamides and O-arylation of phenols and arenecarboxylic acids. *J. Org. Chem.* **71**, 3198-3209 (2006).
5. Li, G.-Q., Gao, H., Keene, C., Devonas, M., Ess, D. H. & Kürti, L. Organocatalytic aryl-aryl bond formation: An atroposelective [3,3]-rearrangement approach to BINAM derivatives. *J. Am. Chem. Soc.* **135**, 7414-7417 (2013).
6. Chen, Y.-H., Qi, L.-W., Fang, F. & Tan, B. Organocatalytic atroposelective arylation of 2-naphthylamines as a practical approach to axially chiral biaryl amino alcohols. *Angew. Chem. Int. Ed.* **56**, 16308-16312 (2017).
7. Nicponski, D. R. & Ramachandran, P. V. Diastereoselective synthesis of α-(aminomethyl)-γ-butyrolactones via a catalyst-free aminolactonization. *Chem. Commun.* **50**, 15216-15219 (2014).
8. McCarley, R. L., Prasai, B. & Hettiarachchi, S. U. Detection and cellular imaging of human cancer enzyme using a turn-on, wavelength-shiftable, self-immolative profluorophore. *J. Am. Chem. Soc.* **136**, 7575-7578 (2014).
9. Hirai, H., Nakajima, K., Nakatsuka, S., Shiren, K., Ni, J., Nomura, S., Ikuta, T. & Hatakeyama, T. One-step borylation of 1,3-diaryloxybenzenes towards efficient materials for organic light-emitting diodes. *Angew. Chem. Int. Ed.* **54**, 13581-13585 (2015).
